# An Intramolecular Reaction between Pyrroles and Alkynes Leads to Pyrrole Dearomatization under Cooperative Actions of a Gold Catalyst and Isoxazole Cocatalysts

**DOI:** 10.1021/acs.orglett.4c02601

**Published:** 2024-08-19

**Authors:** Satish Bhausaheb Dawange, Rai-Shung Liu

**Affiliations:** Department of Chemistry, National Tsing-Hua University, Hsinchu 30013, Taiwan (ROC)

## Abstract

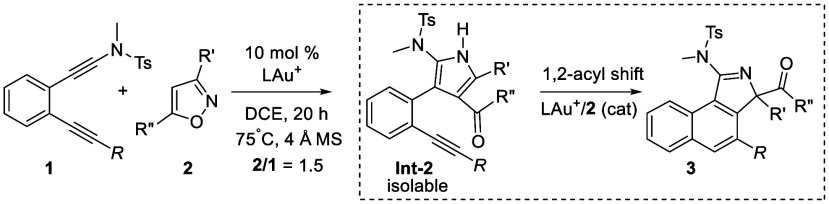

The gold-catalyzed one-pot synthesis of 3*H*-benzo[*e*]isoindoles (**3**) from a mixture of isoxazole (**2**) and diynamides (**1**) is described. This tandem catalysis involves two separate steps: (i) initial synthesis of 2-(3-pyrrolyl)-1-alkynylbenzenes and (ii) a novel alkyne/pyrrole coupling reaction through pyrrole dearomatization. Our control experiments reveal the cooperative action of the gold catalyst and isoxazole cocatalyst to enable the novel alkyne/pyrrole coupling leading to a 1,2-acyl shift.

In organic synthesis, gold-catalyzed reactions have become a powerful tool for constructing many carbo- or heterocyclic frameworks.^1^ Gold-catalyzed cycloisomerizations of enynes represent some of the most valuable reactions.^2^Scheme 1, eq 1 shows one notable enyne cycloisomerization reported by Hashmi,^3^ who used an alkyne and substituted furans to afford substituted phenol derivatives in either inter- or intramolecular systems. This catalytic phenol synthesis is postulated to involve cyclopropylgold carbene **Int-1** as the reaction intermediate (Scheme 1, eq 1). Unfortunately, such an aromatization/rearrangement sequence fails to work with other heteroaromatic compounds, including pyrroles or indoles, which afforded single addition products instead (eq 2).^4^ We sought new chemoselectivity for catalytic coupling reactions between pyrroles and alkynes. This work reports catalytic cascade reactions involving an intramolecular reaction between substituted pyrroles and alkynes as in intermediate **Int-2** (eq 3), further affording 2,2-disubstituted-2*H*-pyrroles (**3**). This tandem catalysis involves two separate steps using one gold catalyst in a one-pot operation. The first step involves the catalytic formation of 2-alkynylphenyl-3-pyrroles (**Int-2**) from a mixture of diynamides (**1**) and isoxazoles (**2**), and the final step ends with catalytic cycloisomerizations between alkynes and their tethered substituted pyrroles, affording the observed 3*H*-benzo[*e*]isoindoles (**3**). In the second step, an intriguing 1,2-acyl shift occurs to induce pyrrole dearomatization. The key intermediates (**Int-2**) can be isolated efficiently, and according to our mechanistic analysis their pyrrole/alkyne reactions require the cooperative action of a gold catalyst and an isoxazole cocatalyst.

**Scheme 1:**
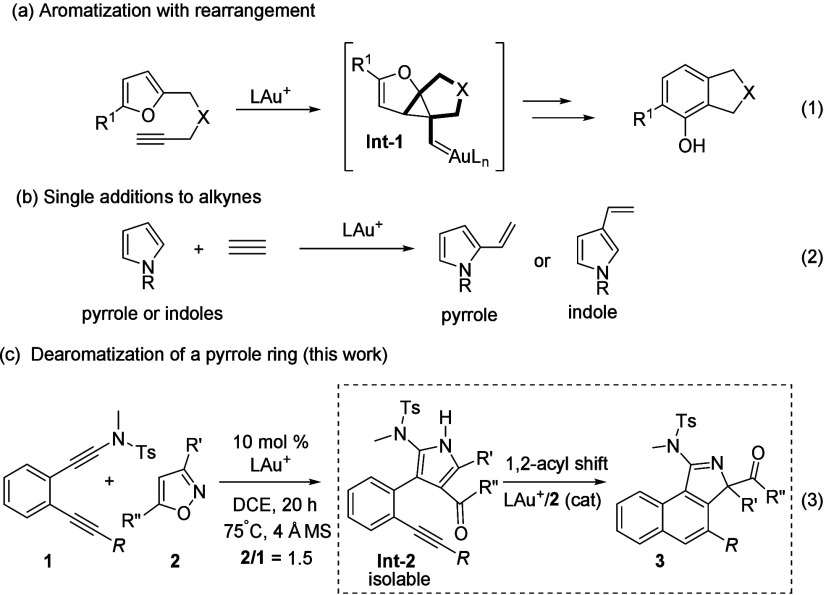
Reactions between Alkynes and Furans or Pyrroles

Catalyst screening was performed using various gold catalysts to optimize the product yields. Our initial test between diynamide (**1a**) and isoxazole (**2a**) (**2a**/**1a** = 1.5) with IPrAuCl/AgNTf_2_ in hot DCE (75 °C) delivered the desired 3*H*-benzo[*e*]isoindole **3a** in 28% yield over 8 h and 58% yield over 24 h (Table 1, entries 1 and 2). With the alteration of the ligands (L) in the LAuCl/AgNTf_2_ catalysts (L = IPr, P(*t*-Bu)_2_(*o*-biphenyl), P(OPh)_3_, P(2,4-*t*-Bu_2_C_6_H_3_O)_3_; 10 mol %, entries 3–6), reaction yields of 55–68% were obtained for our target **3a**, with the PPh_3_ ligand being the most efficient. Changing the silver salts in PPh_3_AuCl/AgX (X = SbF_6_, OTf) gave our target **3a** in 41% and 47% yields, respectively (entries 7 and 8), demonstrating the effects of counteranions. As a counteranion, NTf_2_ appears to be more durable in prolonged heating conditions.^5^ Ph_3_PAuCl/NaBARF (BARF = (C_6_F_5_)_4_B) afforded product **3a** in only 7% yield. AgNTf_2_ alone appeared to be ineffective (entries 9 and 10). The use of PPh_3_AuCl/AgNTf_2_ in various solvents gave compound **3a** in the following yields: 42% for toluene and 47% for MeCN. The molecular structure of compound **3a** was inferred from an X-ray diffraction study of its relative **3d**,^6^ revealing a naphthalene ring fused with a 2-methyl-2-acetyl-2*H*-pyrrole ring. This structure discloses an interesting pyrrole dearomatization, which is accompanied by a 1,2-acyl shift; herein, the loss of energy is compensated for by the formation of a new benzene ring.

**Table 1:**
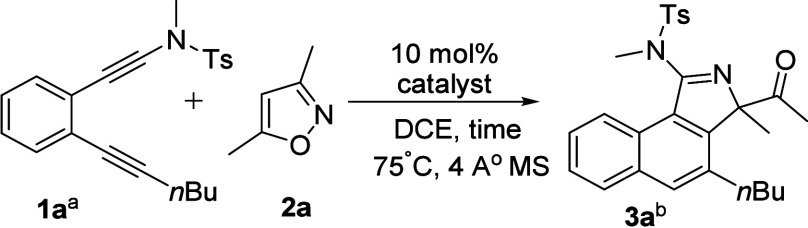
Cascade Cyclizations over Various Catalysts

entry	catalyst (10 mol %)	solvent	time (h)	yield^b^(**3a**)
1	IPrPAuClAgNTf_2_	DCE	8	28
2	IPrPAuCl/AgNTf_2_	DCE	24	58
**3**	**PPh**_**3**_**AuCl/AgNTf**_**2**_	**DCE**	**20**	**68**
4	LAuCl/AgNTf_2_	DCE	22	62
5	(PhO)_3_PAuCl/AgNTf_2_	DCE	23	55
6	L′AuCl/AgNTf2	DCE	24	60
7	PPh_3_AuCl/AgSbF_6_	DCE	20	41
8	PPh_3_AuCl/AgOTf	DCE	20	47
9	PPh_3_AuCl/NaBARF	DCE	24	07
10	AgNTf_2_	DCE	36	00
11	PPh_3_AuCl/AgNTf_2_	toluene	17	42
12	PPh_3_AuCl/AgNTf_2_	MeCN	20	47

a [**1a**] = 0.13 mmol. L = (*t*-Bu)_2_P(*o*-biphenyl), L′ = P(2,4-*t*-Bu)_2_C_6_H_3_O)_3_.

b Product yields were obtained after purification from a silica column. IPr = 1,3-bis(diisopropylphenyl)-imidazole-2-ylidene, DCE = 1,2-dichloroethane, MeCN = acetonitrile.

The diynamide scope was assessed using substrates **1** and isoxazole **2a** under the optimized conditions in Table 1 (entry 3), and the results are summarized in Scheme 2. Notably, two atropisomers can be detected for large sulfonamides (R^1^ = *n*-butyl, *i*-propyl, benzyl), rendering the conformation inflexible around the nitrogen center. We synthesized various diynamides **1b**–**1d** bearing various tosylamides (R^1^ = *n-*Bu, *i-*Pr, Bn; R^2^ = tosyl). Their catalytic cyclizations with 3,5-dimethylisoxazole **2a** afforded the corresponding products **3b**–**3d** as two aptroisomers that were not separable by a silica column; their respective ratios were 1.2:1, 2.0:1, and 2.5:1 with combined yields in the 51–53% range. Further, we prepared diynamide **1e** having R^1^ = Me and R^2^ = Ms, producing the desired product **3e** as a single isomer, albeit in 33% yield. We examined the reactions on additional diynamides **1f** and **1g** with R^1^ = *n-*Bu and Bn, respectively, bearing different mesylamides (R^2^ = Ms), and the resulting products **3f** and **3g** were produced as two inseparable atropisomers in ratios of 2.2:1 and 2.3:1. We next tested the reactions on alkyl-containing internal alkynes (R^3^ = *i-*Pr, *n-*Pr, *n-*hexyl, Me, Cy) as in diynamides **1h–1l**, and their corresponding products **3h**–**3l** were obtained in 45–60% yields. Small MeNT groups avoid forming atropisomers. We also tested the substituents of the central benzene cores. We prepared diynamides **1m** and **1n** bearing C(4) substituents (R^4^ = Me, Cl), further affording compounds **3m** and **3n** in 56% and 47% yields, respectively. For the analogous C(5) substituents (R^5^ = Cl, Br) as in substrates **1o** and **1p**, compounds of the same type (**3o** and **3p**) were obtained 52% and 30% yields, respectively. For diynamide **1q** bearing a phenylethyne group (R^3^ = Ph), the resulting product is contaminated with an impurity that cannot be removed by a silica column or crystallization.

**Scheme 2:**
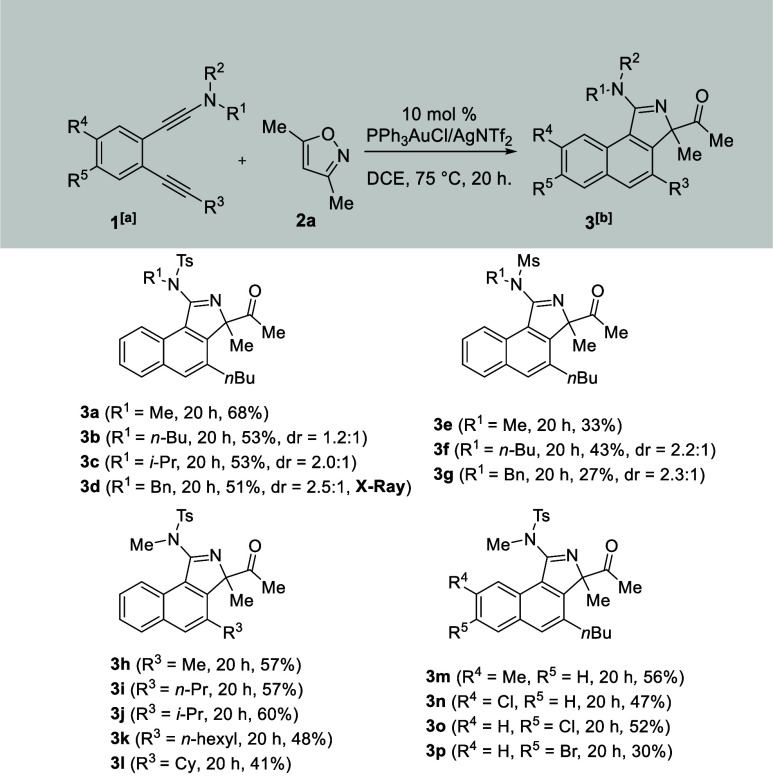
Substrate Scope for Various Diynamides [**1a**] = 0.13 mmol. Product yields are reported after separation from a silica column. DCE = 1,2-dichloroethane.

The scope of 3,5-disubstituted isoxazoles has been studied, and the results are provided in Scheme 3. Notably, isoxazoles monosubstituted at either the C(3) or C(5) position (X or Y = H) are inapplicable substrates. Dialkyl-substituted isoxazoles performed better, possibly due to their better nucleophilicity. We prepared various 3-methylisoxazoles (Y = Me) containing different C(5)-alkyl substituents **2b**–**2e** (X = Et, *i*-Pr, *n*-Bu, *i*-Bu), and their corresponding products were obtained in low yields (ca. 27–31% yields). This trend is reasonable because the resulting products **3** are produced from the N-attack of isoxazoles at the C(1)-ynamide carbon, whereas these long alkyl groups will increase oxygen’s nucleophilicity to facilitate the O-attack of the isoxazole. However, byproducts from the O-attack were absent here because such an attack only occurs with electron-deficient propiolates. We also tested the reactions on isoxazoles **2f** and **2g** bearing large alkyl groups (Y = Pr and Et) at C(5), but the resulting products were obtained in 26% and 46% yields, respectively. We believe that a long alkyl at C(5) as in isoxazoles **2f** and **2g** impedes not only the attack of the isoxazole on the gold π-alkyne complex but also the 1,2-acyl shift in the second step. In the second step, highly hindered tertiary carbon N–**C**Y(COX) is present on the C(2)-pyrrole ring of products **2f** and **2g**. These unfavorable factors on large X or Y substituents of isoxazoles are expected to give a limited scope of applicable isoxazoles.

**Scheme 3:**
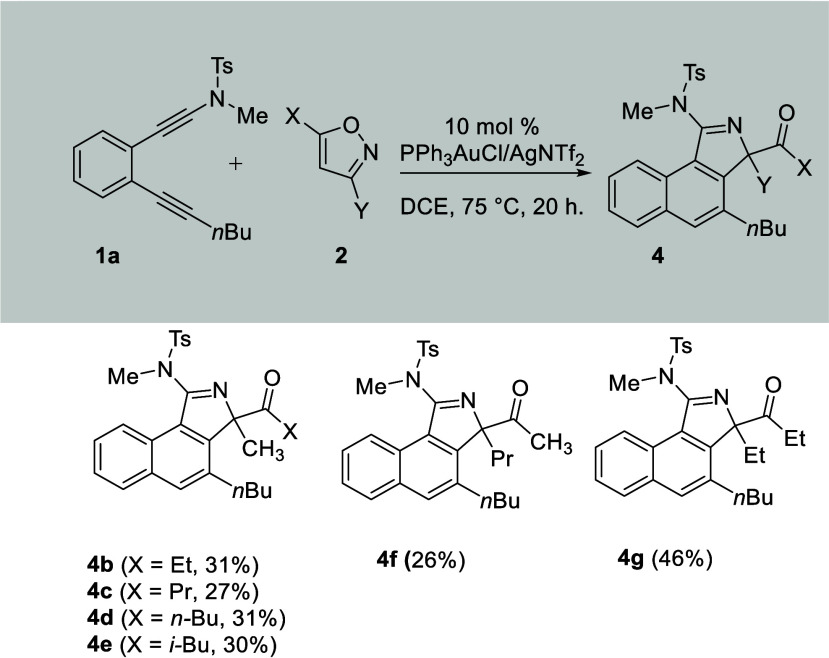
Substrate Scope for Various Isoxazoles [**1a**] = 0.13 mmol. Product yields are reported after separation from a silica column. DCE = 1,2-dichloroethane.

To perform the reaction on a large scale, we used diynamide **1a** on a 1.0 g (2.74 mmol) scale. The reaction produced 0.78 g (1.68 mmol) of compound **3a**, corresponding to a 61% yield. For species **3a**, removal of the tosyl group was achieved with HOTf (1.0 equiv) in DCM, affording compound **5a** in 32% yield (Scheme 4, eq 4). The molecular structure of this new derivative was confirmed by an X-ray diffraction study.^6^ Shown in eq 5 are two control experiments to identify the reaction intermediates. We performed these gold-catalyzed reactions with two diynamides (**1a** and **1j**) at room temperature; we hoped that these mild conditions could isolate tractable intermediates. After workup, two new products (**6a** and **6b**) were isolated in 72% and 66% yields, respectively. The molecular structure of species **6b** was verified by X-ray diffraction.^6^ In subsequent pyrrole/alkyne reactions using PPh_3_AuCl/AgNTf_2_ (10 mol %) in hot DCE (20 h), both species **6a** (R = *n*-butyl) and **6b** (R = *i*-Pr), to our astonishment, only led to 65–76% recovery (eq 5). In a separate experiment (eq 6), we are sure that species **6a** is the intermediate in this two-step catalysis; herein, we note that isoxazole **2b** still remains under the initial condition **2a/1a** = 1.5. Therefore, we tested the reaction on intermediate **6a** with a mixture of the gold catalyst (10 mol %) and isoxazole (20 mol %), smoothly delivering product **3a** in 74% yield (eq 7). This outcome confirms the cocatalyst role of isoxazole, which cooperates catalytically with the gold catalyst in this new pyrrole/alkyne coupling reaction.

**Scheme 4:**
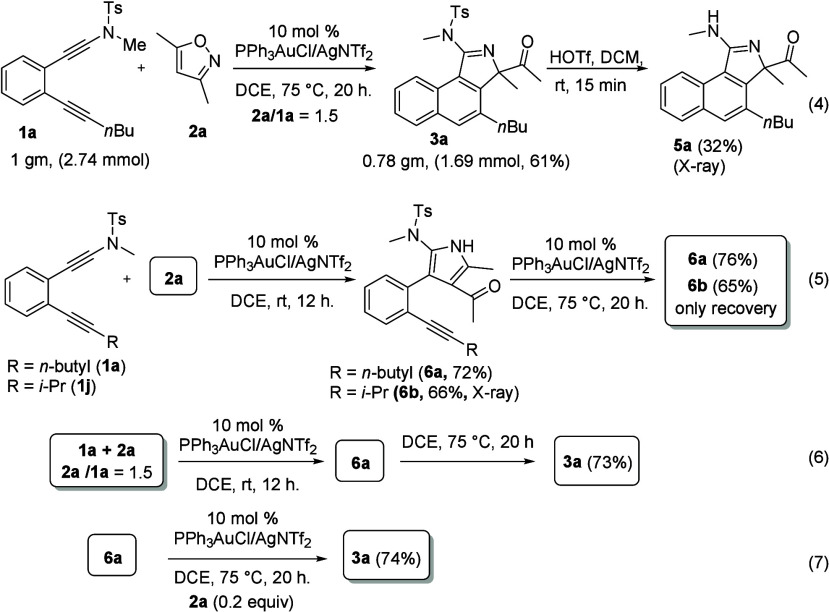
Control Experiments

To gain insight into the mechanism, we performed a crossover experiment involving a 1:1 mixture of intermediates **6a** and **6c** bearing two different sulfonamides and acyl groups. As shown in Scheme 5, we did not obtain any crossover product apart from expected products **3r** and **3a**. This information reveals that the 1,2-acyl migration proceeds in an intramolecular fashion.

**Scheme 5:**
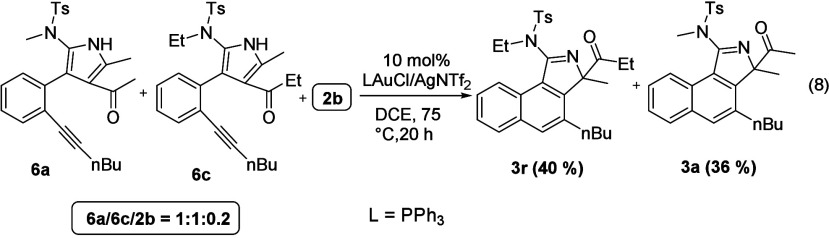
Crossover Experiment

Scheme 6 shows a plausible mechanism for the two separate steps. For the initial diynamide **1a**, its gold π-alkyne complex preferably reacts with isoxazole **2a** at the C(1)-ynamide carbon because it is highly electron-rich. This regioselectivity is expected to afford our isolable pyrrole intermediate **6a** based on an early report from Ye et al.^7^ A subsequent intramolecular cyclization of pyrrole intermediate **6a** is only operable at high temperatures in the presence of both the gold catalyst and the isoxazole cocatalyst. A pyrrole addition at the gold π-alkyne in intermediate **6a** is expected to form species **B** bearing a vinylgold substituent and also an iminium center. This electronic feature activates an intramolecular 1,2-acyl migration^8^ to form gold carbene **C**, which is an ideal precursor for the formation of naphthylgold species **D** after a protonation reaction. A subsequent proto-deauration of this final intermediate **D** delivers the observed product **3a**. In the second step, the loss of energy for pyrrole dearomatization is compensated for by the energy released in the formation of a new benzene. According to our control experiments in Scheme 4, eqs 7 and 8, the presence of isoxazole **2a** is required to enable the pyrrole/alkyne coupling reaction. Deprotonation and proto-deauration for intermediates **C** and **D** require a proton shuttle such as isoxazole (**2a**) to complete this process.

**Scheme 6:**
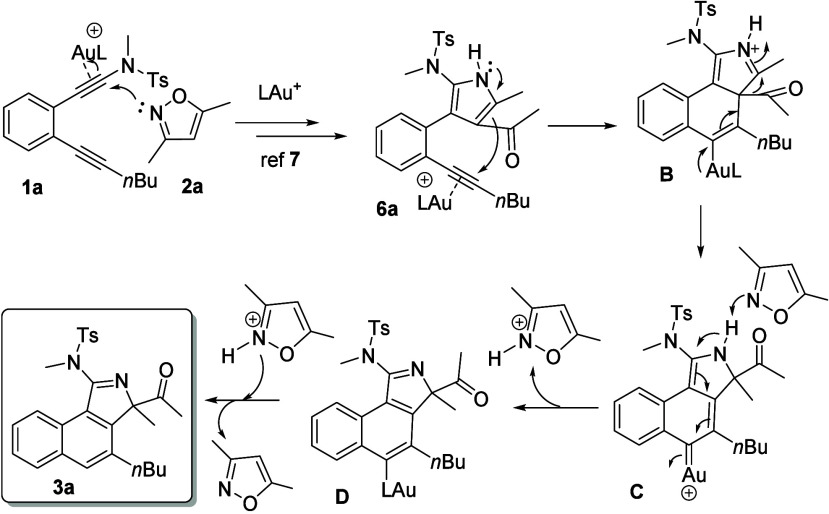
A Postulated Reaction Mechanism

The ultimate goal of this catalysis is to explore the chemoselectivity of a new reaction between pyrroles and alkynes, which are reported to exclusively give alkyne addition products exclusively. This work reports the gold-catalyzed synthesis of 3*H*-benzo[*e*]isoindole (**3**) from a mixture of isoxazoles (**2**) and diynamides (**1**).^9^ This one-pot catalysis involves the initial formation of isolable 2-(3-pyrroly)-1-alkynylbenzenes, followed by catalytic coupling of pyrrole/alkyne functionalities. Herein, new chemoselectivity has been achieved with a pyrrole dearomatization accompanied by a 1,2-acyl shift.^7^ Our control experiments reveal that this atypical alkyne/pyrrole coupling is enabled by the cooperative action of a gold catalyst and an isoxazole cocatalyst. Efforts to realize this reaction in the intermolecular system are under investigation.

## Data Availability

The data underlying this study are available in the published article and its online Supporting Information.

## Supporting Information Available

Experimental procedures, characterization data, crystallography data, and ^1^H NMR and ^13^C NMR spectra for representative compounds (PDF)
