# Supplementary material for: An Intramolecular Reaction between Pyrroles and Alkynes Leads to Pyrrole Dearomatization under Cooperative Actions of a Gold Catalyst and Isoxazole Cocatalysts
Source: Org Lett. 2024 Aug 19;26(34):7181–5. doi: 10.1021/acs.orglett.4c02601 (PMC11372827; doi:10.1021/acs.orglett.4c02601)
Supplement: Supplementary file 1 — ol4c02601_si_001.pdf [file ol4c02601_si_001.pdf]

Supporting Information

An Intramolecular Reaction between Pyrroles and Alkynes leads to a Pyrrole Dearomatization under Cooperative Actions of Gold Catalyst and Isoxazole Cocatalysts.

Satish Bhausaheb Dawange and Rai-Shung Liu*

Department of Chemistry, National Tsing-Hua University, Hsinchu 30013, Taiwan, ROC-----
e-mail: rsliu@mx.nthu.edu.tw

Content:

(1) Representative synthetic procedures: --S2

(2) General procedure for synthesis of substrates: --S2

(3) Standard procedure for catalytic operations: --S3

(4) Synthetic procedures for chemical functionalization: -------------------------------------S5

(5) References: ---S6

(6) Spectral data of new compounds: --S6

(7) X-ray crystallographic data for 3d, 5a and 6b: --S28

(8) ^{1}H and ^{13}C spectra of key compounds: --S33

Experimental Section.

(1) General methods:

Unless otherwise noted, all the preparation of the substrates was performed in oven dried glassware under nitrogen atmosphere with freshly distilled solvents. Catalytic reactions were performed under nitrogen atmosphere. Toluene and DCE were distilled from CaH_2 under nitrogen. THF was distilled from Na metal under nitrogen.Commercial reagents were used without further purification unless otherwise indicated. ^{1}H NMR and ^{13}C NMR spectra were recorded on Varian 700 MHz, Varian 500 MHz and Bruker 400 MHz Spectrometers using chloroform-d ($CDCl_3$: δ_H = 7.24 ppm, δ_C = 77.00 as a solvent and Me_4Si as an internal standard. Chemical Shift (δ) and Spin-Spin coupling constant (*J*). The following abbreviations were used to show the multiplicities: s: singlet, bs: broad singlet, d: doublet, t: triplet, q: quadruplet, dd: doublet of doublet, tt: triplet of triplet, qt: quadruplet of triplet, tq: triplet of quadruplet, m: multiplet. High-resolution mass spectral analysis (HRMS) data were measured on JMS-T100LP4G (JEOL) mass spectrometer or a TOF mass analyzer equipped with the ESI source, JEOL Model: JMS-T200GC AccuTOFGCx equipped with FD (field desorption) source and Magnetic Sector Mass Analyzer (MStation) equipped with the EI source. Melting points were recorded on BUCHI melting point B-540. All heating reactions were carried out with an oil bath as a heat source. Reactions were magnetically stirred and monitored by thin layer chromatography on 0.25 mm E. Merck silica gel plate (60f - 254) using UV light as visualizing agents. Single-crystal X-ray diffraction intensity data were collected on a Bruker X8 APEX diffractometer equipped with a CCD area detector and Mo Kα radiation (λ = 0.71073 Å) at 100 K; all data calculations were performed by using the PC version of the APEX2 program package. All the substrates were prepared according to the literature procedures which are described below.

(2) General procedure for synthesis of substrates:

(a) Synthesis of **N-((2-(hex-1-yn-1-yl)phenyl)ethynyl)-N,4-dimethylbenzenesulfonamide (1a):**

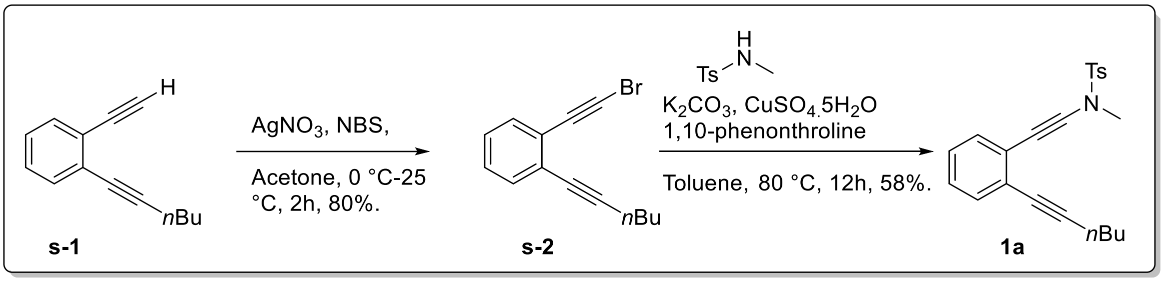

Compound **1-ethynyl-2-(hex-1-yn-1-yl)benzene (s-1)** synthesized according to the literature procedure;[1]

(**i**) Synthesis of **1-(bromoethynyl)-2-(hex-1-yn-1-yl)benzene (s-2);** To a stirring solution of NBS (4.8 gm, 27.16 mmol) and $AgNO_3$ (419 mg, 2.47 mmol) in dry acetone (50 mL) was slowly added **s-1** (4.5 gm, 24.69 mmol) at 0 °C under nitrogen; the resulting mixture was slowly

warmed to room temperature over 2 hrs. Acetone was evaporated and pentane (40 mL) was added to this mixture before further stirring for 10 min at room temperature. The solution was filtered and the filtrate was concentrated to afford **(s-2)** (3 gm, 22.91 mmol, 80%) as dark brown oil.

(**ii**) Synthesis of **N-((2-(hex-1-yn-1-yl)phenyl)ethynyl)-N,4-dimethylbenzenesulfonamide (1a);** To a sealed tube was added 4-methyl-N-phenylbenzenesulfonamide (2.2 gm, 9.16 mmol), $CuSO_4 \cdot 5H_2O$ (190 mg, 0.76 mmol), 1,10-phenanthroline (274 mg, 1.52 mmol) and K_2CO_3 (2.6 gm, 19.0 mmol) and this mixture was subsequently treated with toluene (20 mL) and **s-2** (1.1 gm, 7.63 mmol). The resulting mixture was heated in oil bath at 80 °C for 12 h before cooling to the room temperature. The solution was filtered through a small celite bed and concentrated. Purification of the crude residues was conducted with a silica flash column with ethyl acetate/hexane (20:80) as the eluent to afford desired **N-((2-(hex-1-yn-1-yl)phenyl)ethynyl)-N,4-dimethylbenzenesulfonamide (1a)** (0.9 gm, 2.46 mmol, 58%) as a brown liquid. Other Substrates **1b-1r** were synthesized according to the above procedure

(b) Synthesis of Isoxazole

(i) 3,5-Dimethylisoxazole (2a) was commercially ordered from the TCI chemical industry

(ii) Substrates **2b-2g (3,5-disubstituted Isoxazoles)** were synthesized according to the reported literature procedure; [2]

(3) Standard procedures for catalytic operations;

(a) Standard procedures for Gold catalyzed reactions;

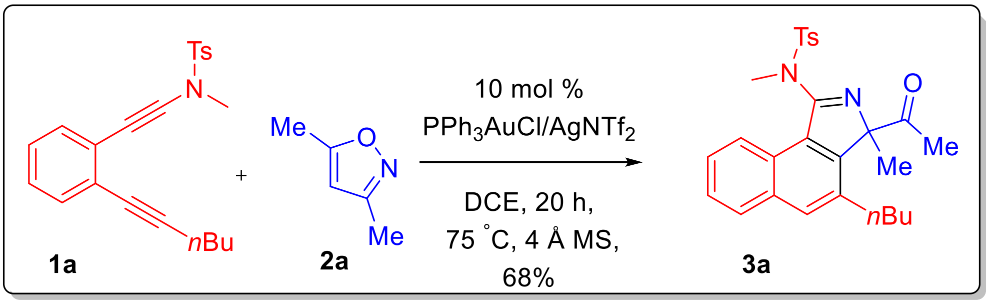

A suspension of LAuCl (L= PPh_3) (6.77 mg, 0.013 mmol) and $AgNTf_2$ (5.31 mg, 0.013 mmol) in dry DCE (1.0 mL) containing 4 Å molecular sieves, was fitted with an N_2 balloon and the mixture was stirred at 25 °C for 5 min. To this mixture was added a dry DCE (2.0 mL) solution of N-((2-(hex-1-yn-1-yl)phenyl)ethynyl)-N,4-dimethylbenzenesulfonamide **(1a)** (50 mg, 0.136 mmol) and 3,5-dimethylisoxazole **(2a)** (19.93 mg, 0.205 mmol) at 25 °C; the resulting mixture was stirred at 75 °C for 20 hours. TLC analysis indicated complete consumption of starting material and formed a polar spot on TLC. The solution was filtered over a short celite bed and evaporated under reduced pressure. The residue was purified on a silica gel column chromatography using ethyl acetate/hexane (10:90) as an eluent to give compound N-(3-acetyl-

4-butyl-3-methyl-3H-benzo[e]isoindol-1-yl)-N,4-dimethylbenzenesulfonamide (**3a)** as White solid (43 mg, 0.054 mmol, 68%).

(b) Catalytic procedure for synthesis of compound **N-(3-acetyl-4-butyl-3-methyl-3H-benzo[e]isoindol-1-yl)-N,4-dimethylbenzenesulfonamide** (**3a**) without isolating intermediate; (For eq. 6 from the manuscript)

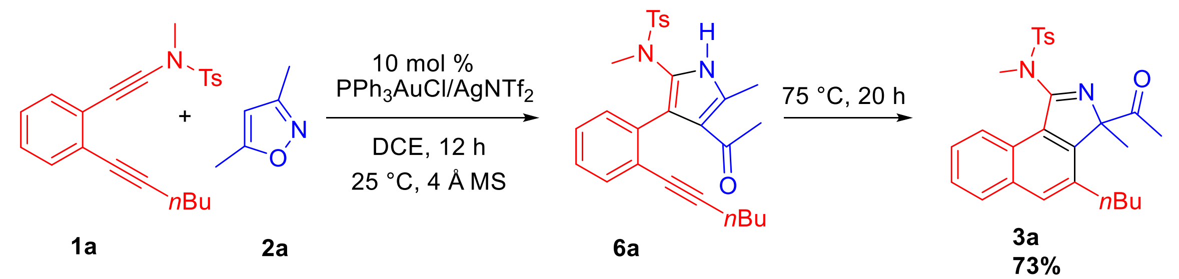

Synthesis of N-(3-acetyl-4-butyl-3-methyl-3H-benzo[e]isoindol-1-yl)-N,4-dimethylbenzenesulfonamide **(3a);** A suspension of LAuCl (L= PPh_3) (6.77 mg, 0.013 mmol) and $AgNTf_2$ (5.31 mg, 0.013 mmol) in dry DCE (1.0 mL) containing 4 Å molecular sieves, was fitted with an N_2 balloon and the mixture was stirred at 25 °C for 5 min. To this mixture was added a dry DCE (2.0 mL) solution of N-((2-(hex-1-yn-1-yl)phenyl)ethynyl)-N,4-dimethylbenzenesulfonamide **(1a)** (50 mg, 0.136 mmol) and 3,5-dimethylisoxazole **(2a)** (19.93 mg, 0.205 mmol) at 25 °C; the resulting mixture was stirred at 25 °C for 12 hours. TLC analysis indicated complete consumption of starting material. Further without isolating intermediate **(6a)** resulting reaction mixture was stirred at 75 °C in oil bath for 20 hours. TLC analysis indicated complete consumption of intermediate **(6a)**. The solution was filtered over a short celite bed and evaporated under reduced pressure. The residue was purified on a silica gel column using ethyl acetate/hexane (10:90) as the eluent to give compound N-(3-acetyl-4-butyl-3-methyl-3H-benzo[e]isoindol-1-yl)-N,4-dimethylbenzenesulfonamide **(3a)** (46 mg, 0.099 mmol, 73%)**.**

(c) Catalytic procedure for synthesis of compound **N-(3-acetyl-4-butyl-3-methyl-3H-benzo[e]isoindol-1-yl)-N,4-dimethylbenzenesulfonamide** (**3a**) by using intermediate (**6a**); (for eq. 7 from the manuscript)

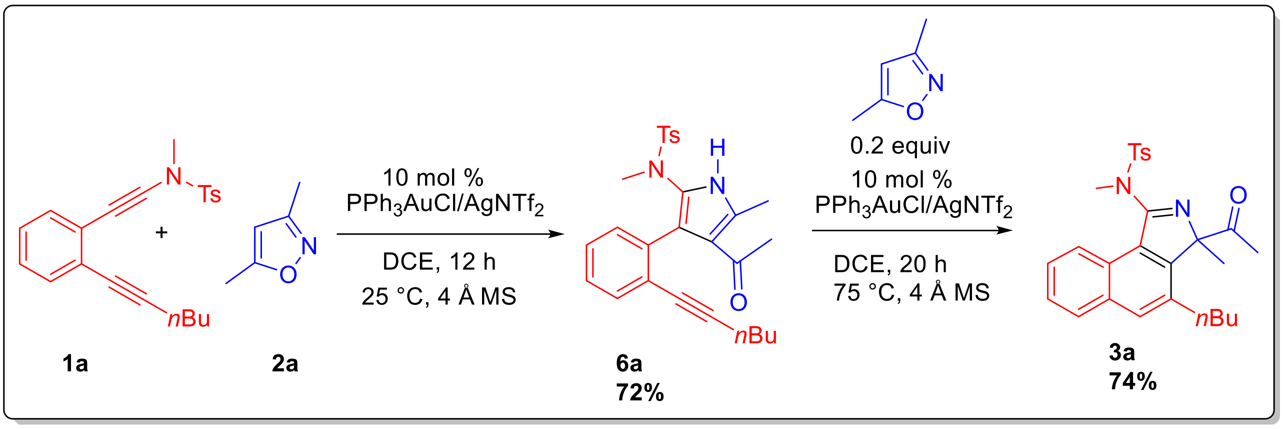

Synthesis of **N-(4-acetyl-3-(2-(hex-1-yn-1-yl)phenyl)-5-methyl-1H-pyrrol-2-yl)-N,4-dimethylbenzenesulfonamide (6a);** A suspension of LAuCl (L= PPh_3) (6.77 mg, 0.013 mmol) and $AgNTf_2$ (5.31 mg, 0.013 mmol) in dry DCE (1.0 mL) containing 4 Å molecular sieves, was fitted with an N_2 balloon and the mixture was stirred at 25 °C for 5 min. To this mixture was added a dry DCE (2.0 mL) solution of N-((2-(hex-1-yn-1-yl)phenyl)ethynyl)-N,4-dimethylbenzenesulfonamide **(1a)** (50 mg, 0.136 mmol) and 3,5-dimethylisoxazole **(2a)** (19.93 mg, 0.205 mmol) at 25 °C; the resulting mixture was stirred at 25 °C for 12 hours. TLC analysis indicated complete consumption of starting material. The solution was filtered over a short celite bed and evaporated under reduced pressure. The residue was purified on a silica gel column using ethyl acetate/hexane (10:90) as the eluent to give compound N-(4-acetyl-3-(2-(hex-1-yn-1-yl)phenyl)-5-methyl-1H-pyrrol-2-yl)-N,4-dimethylbenzenesulfonamide **(6a)** (54 mg, 0.116 mmol, 72%)**.** Compound **6b** and **6c** also prepared by the same method.

Synthesis of **N-(3-acetyl-4-butyl-3-methyl-3H-benzo[e]isoindol-1-yl)-N,4-dimethylbenzenesulfonamide (3a)**; A suspension of LAuCl (L= PPh_3) (3.06 mg, 0.006 mmol) and $AgNTf_2$ (2.52 mg, 0.006 mmol) in dry DCE (1.0 mL) containing 4 Å molecular sieves, was fitted with an N_2 balloon and the mixture was stirred at 25 °C for 5 min. To this mixture was added a dry DCE (2.0 mL) solution of N-(4-acetyl-3-(2-(hex-1-yn-1-yl)phenyl)-5-methyl-1H-pyrrol-2-yl)-N,4-dimethylbenzenesulfonamide **(6a)** (30 mg, 0.064 mmol) and 3,5-dimethylisoxazole **(2a)** (1.2 mg, 0.012 mmol) at 25 °C; the resulting mixture was stirred at 75 °C for 20 hours. TLC analysis indicated complete consumption of intermediate. The solution was filtered over a short celite bed and evaporated under reduced pressure. The residue was purified on a silica gel column using ethyl acetate/hexane (10:90) as the eluent to give compound N-(3-acetyl-4-butyl-3-methyl-3H-benzo[e]isoindol-1-yl)-N,4-dimethylbenzenesulfonamide **(3a)** (22 mg, 0.116 mmol, 74%)**.**

(4) Synthetic procedures for chemical functionalization;

(a) Synthesis of **1-(4-butyl-3-methyl-1-(methylamino)-3H-benzo[e]isoindol-3-yl)ethanone (5a);**

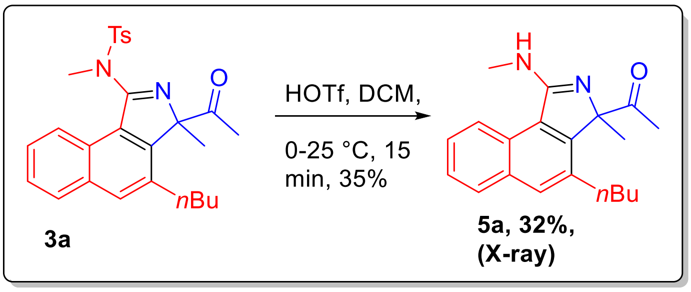

To a solution of **3a** (30 mg, 0.064 mmol) in dry DCM (2.0 mL) at 0 °C was added HOTf (0.05 mL, 0.064 mmol), the mixture was stirred at 25 °C for 15 min, confirmed the completion of reaction using TLC. The reaction mixture was quenched with saturated sodium bicarbonate solution, and the aqueous layer was extracted by dichloromethane three times. DCM was evaporated under reduced pressure and the residue was purified through a silica gel column chromatography with MeOH/DCM (10/90) to yield compound **5a** (6.5 mg, 0.022 mmol, 32 %) as a white solid.

(5) References

(1) Ye, L.; Wang, Y.; Aue, D. H.; Zhang, L. Experimental and Computational Evidence for Gold Vinylidenes: Generation from Terminal Alkynes via a Bifurcation Pathway and Facile C-H Insertions. *J. Am. Chem. Soc.* **2012**, *134*, 31–34.

(2) (a) Perez, J. D.; Wunderlin, D. A. Flash Vacuum Pyrolysis of Some 4-Nitroisoxazoles. *J. Org. Chem.* **1987**, *52*, 3637. (b) Tonder, J. E., Hansen, J. B., Begtrup, M., Pettersson, I., Rimvall, K., Christensen, B., Ehrbar, U., and Olesen, P. H. Improving the Nicotinic Pharmacophore with a Series of (Isoxazole)methylene-1-azacyclic Compounds: Synthesis, Structure−Activity Relationship, and Molecular Modeling. *J. Med. Chem.* **1999,** *42*, 4970-4980. (c) Sahani, R. L.; Liu, R.-S. Development of Gold-Catalyzed [4 + 1] and [2 + 2+1]/[4 + 2] Annulations Between Propiolate Derivatives and Isoxazoles. *Angew. Chem., Int. Ed.* **2017**, *56*, 1026−1030.

(6) Spectral data of key compounds;

Spectral data of N-((2-(hex-1-yn-1-yl)phenyl)ethynyl)-N,4-dimethylbenzenesulfonamide (1a);

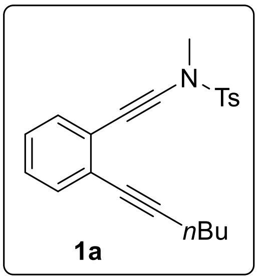

The compound was purified on a silica gel column using ethyl acetate/hexane: (20: 80) as the eluent; Brown oil (0.6 g, 1.64 mmol, 42%); ^{1}H NMR (400 MHz, $CDCl_3$): δ 7.87 (d, *J* = 8 Hz, 2H), 7.28-7.36 (m, 4H), 7.13-7.16 (m, 2H), 3.14 (s, 3H), 2.41 (t, *J* = 4 Hz, 5H), 1.50-1.68 (m, 2H), 1.40-1.45 (m, 2H), and 0.88 (t, *J* = 8 Hz, 3H); ^{13}C NMR (100 MHz, $CDCl_3$): δ 144.6, 133.2, 131.7, 130.7, 129.6, 127.7, 127, 125.5, 125, 94.4, 87.3, 79.1, 68.4, 39.3, 30.6, 21.9, 21.5, 19.2, 13.5; HRMS (ESI-TOF) m/z: [M+Na]+ calcd. For $C_{22}H_{23}NNaO_2S$: 388.1347 found 388.1349.

Spectral data of N-butyl-N-((2-(hex-1-yn-1-yl)phenyl)ethynyl)-4-methylbenzenesulfonamide (1b);

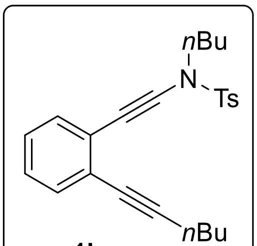

1b

The compound was purified on a silica gel column using ethyl acetate/hexane: (20: 80) as the eluent; Brown oil (0.4 g, 0.98 mmol, 25%); ^{1}H NMR (400 MHz, $CDCl_3$): δ 7.87 (d, *J* = 8 Hz, 2H), 7.29-7.36 (m, 4H), 7.12-7.15 (m, 2H), 3.39 (t, *J* = 4 Hz, 2H), 2.39 (t, *J* = 4 Hz, 5H), 1.71 (t, *J* = 8 Hz, 2H), 1.54 (t, *J* = 8 Hz, 2H), 1.34-1.45 (m, 4H), and 0.89 (q, *J* = 8 Hz, 6H); ^{13}C NMR (100 MHz, $CDCl_3$): δ 144.5, 134.7, 132, 130.9, 129.7, 127.6, 127.2, 127, 125.5, 125.4, 94.3, 85.9, 79.4, 70.1, 51.4, 30.8, 29.8, 22.1, 21.6, 19.5, 19.3, 13.6, 13.6; HRMS (ESI-TOF) m/z: [M]+ calcd. For $C_{25}H_{29}NO_2S$: 408.1997 found 408.2001.

Spectral data of N-((2-(hex-1-yn-1-yl)phenyl)ethynyl)-N-isopropyl-4-methylbenzenesulfonamide (1c);

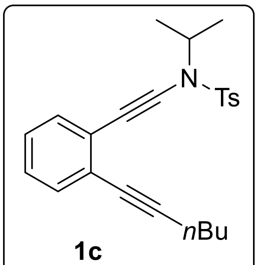

1c

The compound was purified on a silica gel column using ethyl acetate/hexane: (20:80) as the eluent; Brown oil (0.3 g, 0.762 mmol, 19%); ^{1}H NMR (400 MHz, $CDCl_3$): δ 7.86, (d, *J* = 8 Hz, 2H), 7.27-7.36 (m, 4H), 7.10-7.17 (m, 2H), 4.20-4.27 (m, 1H), 2.36 (q, *J* = 4 Hz, 5H), 1.49-

1.56 (m, 2H), 1.36-1.45 (m, 2H), 1.17 (d, J = 4 Hz, 6H) and 0.89 (t, J = 4 Hz, 3H); ^{13}C NMR (100 MHz, $CDCl_3$): δ 144.6, 136.1, 132, 130.8, 129.8, 129.8, 127.4, 127.2, 126.9, 125.7, 125.2, 94.2, 83, 79.6, 72.2, 52.7, 30.7, 22.1, 21.6, 20.6, 19.4, 13.6; HRMS (ESI-TOF) m/z: [M]+ calcd. For $C_{24}H_{27}NO_2S$: 394.1840 found 394.1843.

Spectral data of N-benzyl-N-((2-(hex-1-yn-1-yl)phenyl)ethynyl)-4-methylbenzenesulfonamide(1d);

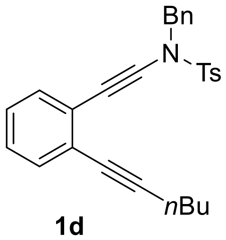

1d

The compound was purified on a silica gel column using ethyl acetate/hexane: (20: 80) as the eluent; Brown oil (500 mg, 1.13 mmol, 29%); ^{1}H NMR (400 MHz, $CDCl_3$): δ 7.85 (d, J = 8 Hz, 2H), 7.36-7.41 (m, 3H), 7.26-7.30 (m, 5H), 7.19 (d, J = 4 Hz, 1H), 7.12-7.14 (q, J = 4 Hz, 2H), 4.63 (s, 2H), 2.39 (t, J = 4 Hz, 5H), 1.57 (q, J = 4 Hz, 2H), 1.44 (q, J = 4 Hz, 2H) and 0.91 (t, J = 8 Hz, 3H); ^{13}C NMR (100 MHz, $CDCl_3$): δ 144.3, 134.6, 134.3, 131.7, 130.6, 129.4, 128.5, 128.2, 128, 127.4, 126.9, 126.8, 125.2, 125, 94.3, 86.2, 79.1, 70.4, 55.8, 30.5, 29.5, 21.9, 21.4, 19.1, 13.4; HRMS (ESI-TOF) m/z: [M]+ calcd. For $C_{28}H_{27}NNaO_2S$: 464.1660 found 464.1662.

Spectral data of N-((2-(hex-1-yn-1-yl)phenyl)ethynyl)-N-methylmethanesulfonamide (1e);

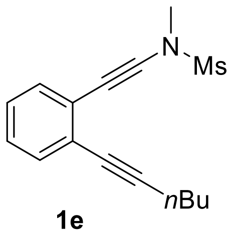

1e

The compound was purified on a silica gel column using ethyl acetate/hexane: (20: 80) as the eluent; Brown oil (200 mg, 0.691 mmol, 18%); ^{1}H NMR (400 MHz, $CDCl_3$): δ 7.32-7.37 (m, 2H), 7.15-7.18 (m, 2H), 3.28 (s, 3H), 3.13 (s, 3H), 2.43 (t, J = 8 Hz, 2H), 1.55-1.62 (m, 2H),1.44-1.51 (m, 2H) and 0.92 (t, J = 8 Hz, 3H); ^{13}C NMR (100 MHz, $CDCl_3$): δ 131.8, 130.9,

127.3, 127.1, 125.8, 124.6, 94.4, 86.3, 79.2, 68.9, 39.1, 36.5, 30.7, 21.9, 19.2, 13.5; HRMS (ESI-TOF) m/z: [M+Na]+ calcd. For $C_{16}H_{19}NNaO_2S$: 312.1034 found 312.1033.

Spectral data of N-butyl-N-((2-(hex-1-yn-1-yl)phenyl)ethynyl)methanesulfonamide (1f);

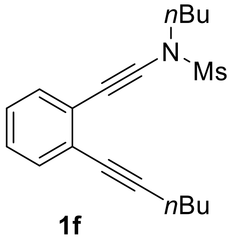

1f

The compound was purified on a silica gel column using ethyl acetate/hexane: (20: 80) as the eluent; Brown oil (0.2 g, 0.60 mmol, 16%); ^{1}H NMR (400 MHz, $CDCl_3$): δ 7.32-7.37 (m, 2H), 7.15-7.18 (m, 2H), 3.54 (t, *J* = 4 Hz, 2H), 3.14 (s, 3H), 2.41 (t, *J* = 4 Hz, 2H), 1.78-1.81 (m, 2H), 1.54-1.78 (m, 2H), 1.40-1.48 (m, 4H), and 0.95 (t, *J* = 4 Hz, 6H); ^{13}C NMR (100 MHz, $CDCl_3$): δ 132, 131.1, 127.3, 127.2, 125.7, 124.9, 94.3, 85, 79.4, 70.5, 51.4, 38, 30.8, 30.2, 22.1, 19.5, 19.3, 13.6, 13.6; HRMS (ESI-TOF) m/z: [M+Na]+ calcd. For $C_{19}H_{25}NNaO_2S$: 354.1503 found 354.1503.

Spectral data of N-benzyl-N-((2-(hex-1-yn-1-yl)phenyl)ethynyl)methanesulfonamide (1g);

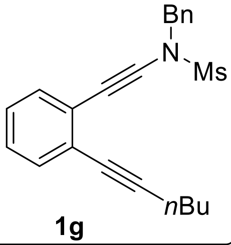

1g

The compound was purified on a silica gel column using ethyl acetate/hexane: (20: 80) as the eluent; Brown oil (0.4 g, 1.09 mmol, 28%); ^{1}H NMR (400 MHz, $CDCl_3$): δ 7.54, (d, *J* = 4 Hz, 2H), 7.35-7.39 (m, 4H), 7.30 (q, *J* = 4 Hz, 1H), 7.16 (q, *J* = 4 Hz, 2H), 4.73 (s, 2H), 2.91 (s, 3H), 2.39 (t, *J* = 4 Hz, 2H), 1.55 (q, *J* = 8 Hz, 2H), 1.45 (q, *J* = 8 Hz, 2H) and 0.92 (t, *J* = 4 Hz, 3H); ^{13}C NMR (100 MHz, $CDCl_3$): δ 134.7, 132, 131, 128.9, 128.8, 128.7, 127.4, 127.2, 125.8, 124.9, 94.5, 85.7, 79.5, 71, 56.1, 38.8, 30.8, 22.1, 19.3, 13.7; HRMS (ESI-TOF) m/z: [M+Na]+ calcd. For $C_{22}H_{23}NNaO_2S$: 388.1347 found 388.1345.

Spectral data of N,4-dimethyl-N-((2-(prop-1-yn-1-yl)phenyl)ethynyl)benzenesulfonamide (1h);

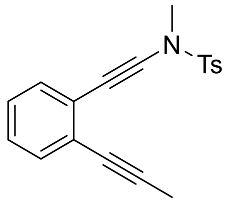

1h

The compound was purified on a silica gel column using ethyl acetate/hexane: (20: 80) as the eluent; Brown oil (0.5 g, 1.55 mmol, 33%); ^{1}H NMR (400 MHz, $CDCl_3$): δ 7.88, (d, *J* = 8 Hz, 2H), 7.33 (d, *J* = 8 Hz, 3H), 7.26-7.28 (m, 1H), 7.13-7.15 (m, 2H), 3.14 (s, 3H), 2.41 (s, 3H), and 1.62 (s, 3H); ^{13}C NMR (100 MHz, $CDCl_3$): δ 144.7, 133.4, 131.7, 130.5, 129.8, 127.8, 127.2, 127, 125.5, 125.3, 90, 87.5, 78.4, 68.6, 39.4, 29.7, 21.6; HRMS (ESI-TOF) m/z: [M+Na]+ calcd. For $C_{19}H_{17}NNaO_2S$: 346.0877 found 346.0880.

Spectral data of N,4-dimethyl-N-((2-(pent-1-yn-1-yl)phenyl)ethynyl)benzenesulfonamide (1i);

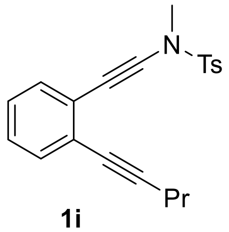

1i

The compound was purified on a silica gel column using ethyl acetate/hexane: (20: 80) as the eluent; Brown oil (1 g, 2.85mmol, 47%); ^{1}H NMR (400 MHz, $CDCl_3$): δ 7.86 (d, *J* = 24 Hz, 2H), 7.28-7.36 (m, 4H), 7.13-7.15 (m, 2H), 3.13 (s, 3H), 2.38 (t, *J* = 8 Hz, 5H), 1.55 (q, *J* = 8 Hz, 2H), and 0.98 (t, *J* = 8 Hz, 3H); ^{13}C NMR (100 MHz, $CDCl_3$): δ 144.6, 133.1, 131.6, 130.7, 129.6, 127.5, 126.9, 125.4, 125, 94.1, 87.2, 79.2, 68.3, 60.1, 39.2, 21.9, 21.3, 13.9, 13.3; HRMS (ESI-TOF) m/z: [M+Na]+ calcd. For $C_{21}H_{21}NNaO_2S$: 374.1190 found 374.1190.

Spectral data of N,4-dimethyl-N-((2-(3-methylbut-1-yn-1-yl)phenyl)ethynyl)benzenesulfonamide (1j);

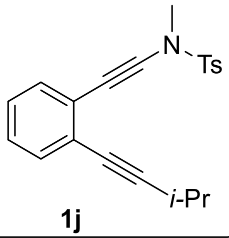

1j

The compound was purified on a silica gel column using ethyl acetate/hexane: (20: 80) as the eluent; Brown oil (0.6 g, 1.71 mmol, 42%); ^{1}H NMR (400 MHz, $CDCl_3$): δ 7.86 (d, J = 8 Hz, 2H), 7.29-7.35 (m, 4H), 7.13-7.15 (m, 2H), 3.14 (s, 3H), 2.73-2.80 (m, 1H), 2.40 (q, 3H), 1.21 (s, 3H) and 1.19 (s, 3H); ^{13}C NMR (100 MHz, $CDCl_3$): δ 144.6, 133.1, 131.7, 130.8, 129.6, 127.6, 127.1, 127, 125.4, 124.9, 99.6, 87.2, 78.3, 68.3, 39.3, 29.5, 22.8, 21.4, 21.1; HRMS (ESI-TOF) m/z: [M+Na]+ calcd. For $C_{21}H_{21}NNaO_2S$: 374.1192 found 374.1190.

Spectral data of N,4-dimethyl-N-((2-(3-oxooct-1-yn-1-yl)phenyl)ethynyl)benzenesulfonamide (1k);

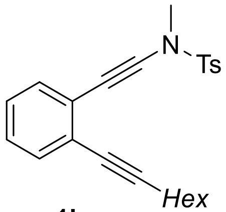

1k

The compound was purified on a silica gel column using ethyl acetate/hexane: (20: 80) as the eluent; Brown oil (1 g, 2.55 mmol, 48%); ^{1}H NMR (400 MHz, $CDCl_3$): δ 7.88 (t, J = 8 Hz, 2H), 7.28-7.36 (m, 4H), 7.13-7.15 (m, 2H), 3.14 (s, 3H), 2.40 (t, J = 8 Hz, 5H), 1.55 (t, J = 8 Hz, 2H), 1.40 (q, J = 8 Hz, 2H), 1.24-1.30 (m, 4H), and 0.85 (q, J = 4 Hz, 3H); ^{13}C NMR (100 MHz, $CDCl_3$): δ 144.6, 133.3, 131.7, 130.6, 129.6, 127.7, 127, 126.9, 125.5, 125.1, 94.5, 87.3, 79.1, 68.4, 39.3, 31.2, 28.6, 28.5, 22.4, 21.5, 19.5, 13.9; HRMS (ESI-TOF) m/z: [M+Na]+ calcd. For $C_{24}H_{27}NNaO_2S$: 416.1660 found 416.1656.

Spectral data of N-((2-(cyclohexylethynyl)phenyl)ethynyl)-N,4-dimethylbenzenesulfonamide (1l);

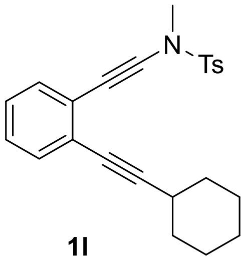

1l

The compound was purified on a silica gel column using ethyl acetate/hexane: (20: 80) as the eluent; Brown oil (0.4 g, 1.02 mmol, 29%); ^{1}H NMR (400 MHz, $CDCl_3$): δ 7.85 (d, J = 8 Hz, 2H), 7.30-7.35 (m, 4H), 7.12-7.15 (m, 2H), 3.13 (s, 3H), 2.57 (t, J = 4 Hz, 1H), 2.39 (s, 3H), 1.74-1.80 (m, 2H), 1.70 (q, J = 4 Hz, 2H), 1.43-1.51 (m, 3H) and 1.26 (d, J = 8 Hz, 3H); ^{13}C NMR (100 MHz, $CDCl_3$): δ 144.8, 133.3, 131.9, 131.2, 129.8, 127.8, 127.3, 127.1, 125.8,

125.1, 98.4, 87.3, 79.3, 68.4, 39.4, 32.6, 29.8, 25.9, 24.8, 21.6; HRMS (ESI-TOF) m/z: [M+Na]+ calcd. For $C_{24}H_{25}NNaO_2S$: 414.1503 found 414.1501.

Spectral data of N-((2-(hex-1-yn-1-yl)-5-methylphenyl)ethynyl)-N,4-dimethylbenzenesulfonamide(1m);

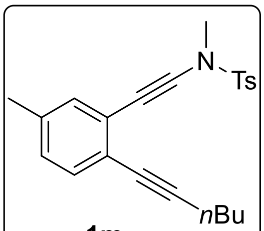

1m

The compound was purified on a silica gel column using ethyl acetate/hexane: (20: 80) as the eluent; Brown oil (0.5 g, 1.32 mmol, 36%); ^{1}H NMR (400 MHz, $CDCl_3$): δ 7.87 (d, *J* = 8 Hz, 2H), 7.33 (d, *J* = 8 Hz, 2H), 7.23 (d, *J* = 8 Hz, 1H), 7.12 (s, 1H), 6.95 (d, *J* = 4 Hz, 1H), 3.13 (s, 3H), 2.42 (s, 3H), 2.38 (t, *J* = 8 Hz, 2H), 2.26 (s, 3H), 1.49-1.53 (m, 2H), 1.38-1.43 (m, 2H) and 0.87 (t, *J* = 8 Hz, 3H); ^{13}C NMR (100 MHz, $CDCl_3$): δ 144.6, 137.1, 133.4, 131.7, 131.4, 129.7, 128.1, 127.8, 124.9, 122.7, 93.5, 87, 79.2, 68.6, 39.4, 30.8, 22, 21.6, 21.1, 19.3, 13.6; HRMS (ESI-TOF) m/z: [M+Na]+ calcd. For $C_{23}H_{25}NNaO_2S$: 402.1503 found 402.1502.

Spectral data of N-((5-chloro-2-(hex-1-yn-1-yl)phenyl)ethynyl)-N,4-dimethylbenzenesulfonamide(1n);

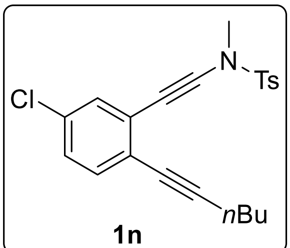

1n

The compound was purified on a silica gel column using ethyl acetate/hexane: (20: 80) as the eluent; Brown oil (0.4 g, 1.0 mmol, 29%); ^{1}H NMR (400 MHz, $CDCl_3$): δ 7.85 (q, *J* = 8 Hz, 2H), 7.34 (d, *J* = 8 Hz, 2H), 7.24 (t, *J* = 4 Hz, 2H), 7.09 (q, *J* = 4 Hz, 1H), 3.13 (s, 3H), 2.38-2.46 (m, 5H), 1.49-1.57 (m, 2H), 1.37-1.46 (m, 2H), 0.87 (t, *J* = 8 Hz, 3H); ^{13}C NMR (100 MHz, $CDCl_3$): δ 144.9, 133.3, 132.9, 132.8, 130.3, 129.8, 127.7, 127.3, 126.8, 123.9, 95.5, 88.6, 78.3, 69.8, 39.3, 30.6, 22, 21.6, 19.3, 13.6; HRMS (ESI-TOF) m/z: [M+Na]+ calcd. For $C_{22}H_{22}ClNNaO_2S$: 422.0957 found 422.0953.

Spectral data of N-((4-chloro-2-(hex-1-yn-1-yl)phenyl)ethynyl)-N,4-

dimethylbenzenesulfonamide (1o);

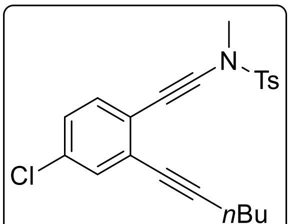

1o

The compound was purified on a silica gel column using ethyl acetate/hexane: (20: 80) as the eluent; Brown oil (0.6 g, 1.50 mmol, 44%); ^{1}H NMR (400 MHz, $CDCl_3$): δ 7.85 (q, *J* = 8 Hz, 2H), 7.32-7.35 (m, 3H), 7.19-7.24 (m, 1H), 7.10-7.13 (m, 1H), 3.13 (t, *J* = 4 Hz, 3H), 2.38-2.42 (m, 5H), 1.49-1.61 (m, 2H), 1.39-1.46 (m, 2H), and 0.86-0.90 (m, 3H); ^{13}C NMR (100 MHz, $CDCl_3$): δ 144.8, 133.4, 132.7, 131.8, 131.6, 129.8, 127.8, 127.5, 127.1, 123.7, 96, 88.2, 78.2, 67.8, 39.3, 30.6, 22, 21.6, 19.2, 13.5; HRMS (ESI-TOF) m/z: [M+Na]+ calcd. For $C_{22}H_{22}ClNNaO_2S$: 422.0957 found 422.0952.

Spectral data of N-((4-bromo-2-(hex-1-yn-1-yl)phenyl)ethynyl)-N,4-dimethylbenzenesulfonamide (1p);

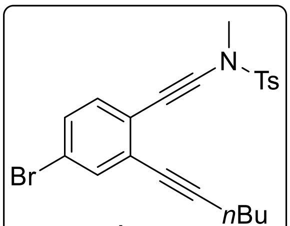

1p

The compound was purified on a silica gel column using ethyl acetate/hexane: (20: 80) as the eluent; Brown oil (0.6 g, 1.35 mmol, 45%); ^{1}H NMR (500 MHz, $CDCl_3$): δ 7.87 (d, *J* = 10 Hz, 2H), 7.51 (d, *J* = 2 Hz, 1H), 7.35 (d, *J* = 10 Hz, 2H), 7.28 (dd, *J* = 10 Hz, 1H), 7.15 (d, *J* = 10 Hz, 1H), 3.15 (s, 3H), 2.42 (t, *J* = 10 Hz, 5H), 1.53 (q, *J* = 10 Hz, 2H), 1.42 (q, *J* = 5 Hz, 2H) and 0.9 (t, *J* = 5 Hz, 3H); ^{13}C NMR (100 MHz, $CDCl_3$): δ 159.6, 139.2, 134.1, 133.9, 129.1, 116.9, 116.5, 114.2, 114, 95.4, 80.7, 79, 76.3, 55.3, 30.7, 29.7, 22, 19.3, 13.7; HRMS (ESI-TOF) m/z: [M+Na]+ calcd. For $C_{22}H_{22}BrNNaO_2S$: 466.0452 found 466.0456.

Spectral data of N-ethyl-N-((2-(hex-1-yn-1-yl)phenyl)ethynyl)-4-methylbenzenesulfonamide (1r);

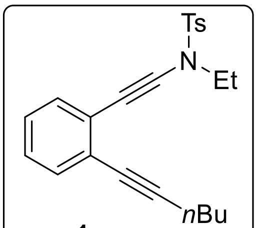

1r

The compound was purified on a silica gel column using ethyl acetate/hexane: (20: 80) as the eluent; Brown oil (0.8 g, 2.11 mmol, 55%); ^{1}H NMR (400 MHz, $CDCl_3$): δ 7.86 (t, *J* = 8 Hz, 2H), 7.30-7.36 (m, 4H), 7.13-7.18 (m, 2H), 3.46 (q, *J* = 8 Hz, 2H), 2.38 (q, *J* = 8 Hz, 5H), 1.49-1.54 (m, 2H), 1.38-1.44 (m, 2H), 1.28 (t, *J* = 8 Hz, 3H) and 0.90 (t, *J* = 8 Hz, 3H); ^{13}C NMR (100 MHz, $CDCl_3$): δ 144.5, 134.9, 131.9, 130.8, 129.7, 127.6, 127.1, 127.0, 125.5, 125.4, 94.4, 85.5, 79.3, 70.4, 46.8, 30.7, 22.0, 21.6, 19.3, 13.6, 13.1; HRMS (ESI-TOF) m/z: [M+Na]+ calcd. For $C_{23}H_{25}NNaO_2S$: 402.1503 found 402.1506.

Spectral data of Products;

Spectral data of N-(3-acetyl-4-butyl-3-methyl-3H-benzo[e]isoindol-1-yl)-N,4-dimethylbenzenesulfonamide (3a);

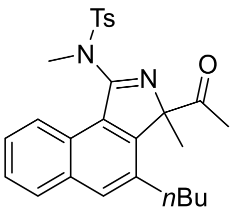

3a

The compound was purified on a silica gel column using ethyl acetate/hexane: (20: 80) as the eluent; MP 186-188 °C, White Solid, (43 mg, 0.09 mmol, 68%); ^{1}H NMR (400 MHz, $CDCl_3$): δ 8.99 (d, *J* = 0.3 Hz, 1H), 7.87 (t, *J* = 8 Hz, 4H), 7.65-7.69 (m, 1H), 7.53-7.57 (m, 2H), 7.33 (d, *J* = 8 Hz, 2H), 3.24 (d, *J* = 0.3 Hz, 3H), 2.67-2.82 (m, 2H), 2.44 (s, 3H) 1.58-1.66 (m, 4H), 1.54 (s, 2H) 1.42-1.48 (m, 3H) and 0.98 (t, *J* = 8 Hz, 3H); ^{13}C NMR (100 MHz, $CDCl_3$): δ 203, 168.8, 153.5, 144.4, 135.4, 134.6, 131.7, 131.1, 129.7, 129.5, 129.1, 127.9, 127.2, 126.6, 126.4, 123.7, 84.2, 37, 33.2, 30.6, 23.6, 22.6, 21.6, 17.9, 13.9; HRMS (ESI-TOF) m/z: [M]+ calcd. For $C_{27}H_{30}N_2O_3S$: 463.2055 found 463.2056.

Spectral data of N-(3-acetyl-4-butyl-3-methyl-3H-benzo[e]isoindol-1-yl)-N-butyl-4-methylbenzenesulfonamide (3b);

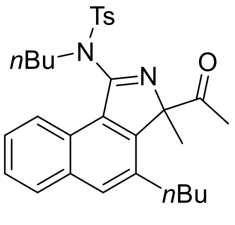

3b

The compound was purified on a silica gel column using ethyl acetate/hexane: (20: 80) as the eluent; Yellow Semi-Solid (33 mg, 0.06 mmol, 53%); ^{1}H NMR (700 MHz, CDCl$_3$): ^{1}H NMR for major isomer; δ 9.33 (d, *J* = 7 Hz, 1H), 7.85 (d, *J* = 7 Hz, 2H), 7.75 (t, *J* = 7 Hz, 1H), 7.67 (t, *J* = 7 Hz, 4H), 7.56 (t, *J* = 7 Hz, 1H), 7.35 (t, *J* = 7 Hz, 5H), 7.21 (d, *J* =7 Hz, 1H), 7.04 (t, *J* = 7 Hz, 2H), 7.01 (d, *J* = 7 Hz, 2H), 5.15 (d, *J* = 7 Hz, 1H), 4.47 (d, *J* = 7 Hz, 1H), 2.65 (d, *J* = 14 Hz, 2H), 2.57 (t, *J* = 7 Hz, 1H), 2.45 (s, 4H), 1.54-1.64 (m, 3H), 1.36-1.41 (m, 3H), 1.31 (s, 1H), 0.93 (t, *J* = 7 Hz, 4H) and 0.54 (s, 3H); ^{1}H NMR for minor isomer; δ 9.12 (d, *J* = 7 Hz, 1H), 7.81 (d, *J* = 7 Hz, 2H), 7.51 (t, *J* = 7 Hz, 1H), 5.0 (d, *J* = 7 Hz, 1H), 4.57 (d, *J* = 7 Hz, 1H) rest of the peaks are merged with major isomer peaks; ^{13}C NMR (175 MHz, CDCl$_3$): for major isomer; δ 203.1, 168.1, 153.2, 144.9, 135.2, 134.7, 134.7, 134.6, 134.4, 134.1, 133.1, 133, 132.4, 130.9, 130.8, 129.7, 129.7, 129.5, 129.3, 128.9, 128.5, 128.5, 128.1, 128.1, 127.8, 127.8, 127.7, 127.7, 126.9, 126.9, 126.5, 126.4, 126.2, 124.6, 123.8, 83.9, 54.2, 33.1, 30.5, 30.4, 24.3, 22.6, 22.6, 22.1, 21.7, 21.6, 17.7, 13.9, for minor isomer; δ 203.3, 167.7, 153.8, 144.5, 84, 54.6, 32.9, 18.1, rest of the peaks are merged with major isomer peaks; HRMS (ESI-TOF) m/z: [M]+ calcd. For $C_{30}H_{36}N_2O_3S$: 505.2524 found 505.2518.

Spectral data of N-(3-acetyl-4-butyl-3-methyl-3H-benzo[e]isoindol-1-yl)-N-isopropyl-4-methylbenzenesulfonamide (3c);

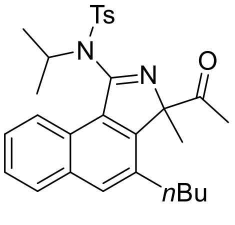

3c

The compound was purified on a silica gel column using ethyl acetate/hexane: (20: 80) as the eluent; Yellow Semi-solid (33 mg, 0.06 mmol, 53%); ^{1}H NMR (400 MHz, CDCl$_3$): ^{1}H NMR for major isomer; δ 9.15 (s, 1H), 7.94 (d, *J* = 8 Hz, 2H), 7.88 (d, *J* = 8 Hz, 1H), 7.82 (d, *J* = 8 Hz, 1H), 7.76 (s, 1H), 7.66-7.76 (m, 1H), 7.52-7.56 (m, 1H), 7.32 (t, *J* = 8 Hz, 3H), 4.41-4.48 (m, 1H), 2.71-2.80 (m, 3H), 2.43 (s, 5H), 1.76 (d, *J* = 4 Hz, 4H), 1.59-1.69 (m, 7H), 1.43-1.48

(m, 2H), 1.25 (d, J = 8 Hz, 3H), 1.15 (d, J = 8 Hz, 2H), and 1.03 (t, J = 8 Hz, 7H); for minor isomer; 4.59-4.65 (m, 1H), rest of the peaks are merged with major isomer peaks; ^{13}C NMR (100 MHz, $CDCl_3$): for major isomer; δ 203.5, 165.6, 153.4, 143.9, 138.2, 135.1, 135, 134.8, 134.7, 134.3, 133.7, 130.9, 130.9, 129.7, 129.4, 128.5, 128.2, 128, 127.2, 127.1, 126.6, 126.5, 126.4, 123.6, 123.4, 84.5, 52.8, 33.2, 33.1, 30.6, 30.6, 24.5, 24, 22.7, 21.9, 21.6, 21.6, 21, 18.3, 18, for minor isomer; δ 203.1, 166.3, 153.2, 143.8, 137.7, 84.7, 53.3, rest of the peaks are merged with major isomer peaks; HRMS (ESI-TOF) m/z: [M]+ calcd. For $C_{29}H_{34}N_2O_3S$: 491.2368 found 491.2361.

Spectral data of N-(3-acetyl-4-butyl-3-methyl-3H-benzo[e]isoindol-1-yl)-N-benzyl-4-methylbenzenesulfonamide (3d);

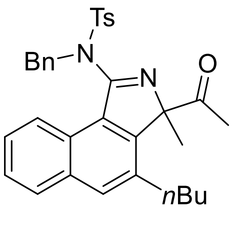

3d

The compound was purified on a silica gel column using ethyl acetate/hexane: (20: 80) as the eluent; MP 172-174 °C, Yellow Semi-solid (31 mg, 0.05 mmol, 51%); ^{1}H NMR (700 MHz, $CDCl_3$): ^{1}H NMR for major isomer; δ 9.15 (d, J = 7 Hz, 1H), 7.83-7.88 (m, 3H), 7.75 (t, J = 7 Hz, 2H), 7.65 (q, J = 14 Hz, 4H), 7.54 (t, J = 7 Hz, 1H), 7.30 (q, J = 7 Hz, 3H), 3.50-3.85 (m, 2H), 2.69-2.78 (m, 4H), 2.41 (s, 5H), 1.73 (s, 6H), 1.61-1.70 (m, 7H), 1.42-1.60 (m, 7H), 1.32-1.37 (m, 3H), 1.21 (q, J = 7 Hz, 2H), 0.95 (t, J = 7 Hz, 5H), 0.79 (t, J = 7 Hz, 3H) and 0.73 (t, J = 14 Hz, 3H); ^{1}H NMR for minor isomer; δ 9.12 (d, J = 7 Hz, 1H), 7.80 (d, J = 7 Hz, 1H), 7.51 (t, J = 7 Hz, 1H), 5.01 (d, J = 7 Hz, 1H), 4.57 (d, J = 14 Hz, 1H), rest of the peaks are merged with major isomer peaks; ^{13}C NMR (175 MHz, $CDCl_3$): for major isomer; δ 202.8, 168.9, 153, 144.4, 135.3, 135, 134.7, 134.6, 133.5, 133, 132.9, 131.1, 131, 129.6, 129.4, 128.7, 128.4, 127.9, 127.8, 127.2, 126.9, 126.6, 126.6, 126.5, 126.4, 124.2, 123.6, 84.2, 50.7, 33.2, 30.5, 30.5, 29.6, 24.2, 23.6, 22.6, 22.6, 21.6, 21.6, 20, 19.8, 17.9, 13.9, 13.5 ; for minor isomer; δ 203.3, 168, 153.8, 144.2, 84.1, 50.6, 33.1, 30.6, 18.2, 13.4, rest of the peaks are merged with major isomer peaks; HRMS (ESI-TOF) m/z: [M]+ calcd. For $C_{33}H_{34}N_2O_3S$: 561.2187 found 561.2188.

Spectral data of N-(3-acetyl-4-butyl-3-methyl-3H-benzo[e]isoindol-1-yl)-N-methylmethanesulfonamide (3e);

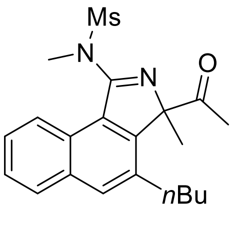

3e

The compound was purified on a silica gel column using ethyl acetate/hexane: (20: 80) as the eluent; Yellow Semi-solid (22 mg, 0.05 mmol, 33%); ^{1}H NMR (700 MHz, $CDCl_3$): δ 8.73 (d, *J* = 7 Hz, 1H), 7.87 (d, *J* = 7 Hz, 1H), 7.76 (s, 1H), 7.62 (t, *J* = 7 Hz, 1H), 7.54 (t, *J* = 7 Hz, 1H), 3.41 (s, 3H), 3.33 (s, 3H), 2.77-2.82 (m, 1H), 2.70-2.74 (m, 1H), 1.70 (s, 3H), 1.55-1.61 (m, 5H), 1.42-1.46 (m, 2H), and 0.96 (t, *J* = 7 Hz, 3H); ^{13}C NMR (175 MHz, $CDCl_3$): δ 203, 168.7, 153.8, 135.6, 134.4, 131.3, 130.5, 128.0, 127.5, 126.8, 126.0, 122.9, 84.4, 37.1, 33.2, 30.5, 23.5, 22.6, 17.8, 13.9; HRMS (ESI-TOF) m/z: [M+Na]+ calcd. For $C_{21}H_{26}N_2O_3S$: 409.1561 found 409.1559.

Spectral data of N-(3-acetyl-4-butyl-3-methyl-3H-benzo[e]isoindol-1-yl)-N-butylmethanesulfonamide (3f);

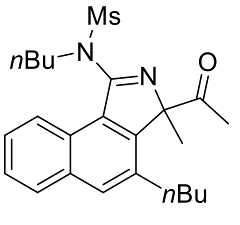

3f

The compound was purified on a silica gel column using ethyl acetate/hexane: (20: 80) as the eluent; Yellow Semi-solid (28 mg, 0.06 mmol, 43%); ^{1}H NMR (700 MHz, $CDCl_3$): ^{1}H NMR for major isomer; δ 8.87 (d, *J* = 7 Hz, 1H), 7.87 (d, *J* = 7 Hz, 1H), 7.76 (s, 1H), 7.62 (t, *J* = 7 Hz, 1H), 7.53 (d, *J* = 7 Hz, 1H), 3.91-3.95 (m, 1H), 3.76-3.80 (m, 1H), 3.25 (s, 3H), 2.76-2.78 (m, 1H), 2.70-2.74 (m, 1H), 1.70 (d, *J* = 14 Hz, 5H), 1.66 (q, *J* = 7 Hz, 2H), 1.61 (d, *J* = 2.73 Hz, 7H), 1.51-1.56 (m, 1H), 1.42-1.46 (m, 3H), 1.36 (d, *J* = 7 Hz, 1H), 1.30 (q, *J* = 7 Hz, 2H), 0.95 (t, *J* = 7 Hz, 4H), and 0.81 (t, *J* = 7 Hz, 4H); for minor isomer, δ 8.93 (d, *J* = 7Hz, 1H), rest of the peaks are merged with major isomer peaks; ^{13}C NMR (175 MHz, $CDCl_3$): for major isomer, δ 203, 168.3, 153.6, 135.2, 135.1, 134.4, 131.9, 131.1, 130.7, 127.8, 127.2, 127.1, 126.6, 126.3, 126.1, 126, 123.4, 123.4, 123.1, 123, 122.1, 121.7, 84.3, 50.6, 38.7, 37.2, 33.1, 30.6, 30.5, 30.4, 29.7, 29.7, 29.4, 28.2, 26.9, 23.7, 23.5, 22.5, 22.4, 19.9, 19.8, 18.8, 17.8, 13.8;

for minor isomer, δ 202.7, 169.1, 84.1, 51, 13.8, rest of the peaks are merged with major isomer peaks; HRMS (ESI-TOF) m/z: [M+Na] calcd. For $C_{24}H_{32}N_2NaO_3S$: 451.2031 found 451.2028.

Spectral data of N-(3-acetyl-4-butyl-3-methyl-3H-benzo[e]isoindol-1-yl)-N-benzylmethanesulfonamide (3g);

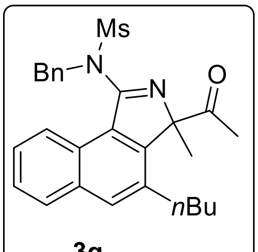

The compound was purified on a silica gel column using ethyl acetate/hexane: (20: 80) as the eluent; Yellow Semi-solid (17 mg, 0.03 mmol, 27%); ^{1}H NMR (700 MHz, $CDCl_3$): ^{1}H NMR for major isomer; δ 8.79 (d, *J* = 7 Hz, 1H), 7.82 (d, *J* = 7 Hz, 1H), 7.71 (s, 2H), 7.60-7.69 (m, 2H), 7.52 (t, *J* = 7 Hz, 2H), 7.40 (s, 1H), 2.27 (s, 2H), 7.09-7.22 (m, 5H), 5.07 (t, *J* = 14 Hz, 1H), 4.92 (d, *J* = 14 Hz, 1H), 3.06 (s, 3H), 2.74 (d, *J* = 7 Hz, 1H), 2.66 (q, *J* = 14 Hz, 2H), and 1.63 (s, 8H), 1.60 (s, 5H), 1.39-1.44 (m, 4H), 1.22-1.28 (m, 3H), and 0.94 (t, *J* = 14 Hz, 7H), ^{1}H NMR for minor isomer; δ 9.01 (d, *J* = 7 Hz, 1H), 5.16 (d, *J* = 14 Hz, 1H), 3.12 (s, 3H), 2.60 (s, 1H), and 0.85 (d, *J* = 7 Hz, 3H) other peaks are merged with major isomer; ^{13}C NMR (175 MHz, $CDCl_3$): for major isomer, δ 203.1, 167.5, 153.6, 135.4, 134.9, 134.7, 134.5, 134.4, 133.7, 132, 131.1, 129.7, 128.7, 128.5, 128.2, 128.1, 128, 127.3, 127.2, 127, 126.6, 126.5, 126.2, 125.8, 123.8, 123, 122.7, 121.7, 84.4, 54.3, 40.3, 36.7, 34.6, 33.1, 33, 31.5, 31.3, 30.5, 30.2, 29.6, 25.2, 23.5, 23.1, 22.6, 22.6, 20.6, 17.7, 13.9, for minor isomer, δ 168.43, 153.93, 84, 54.4, 17.8, 14.1, other peaks are merged with major isomer; HRMS (ESI-TOF) m/z: [M]+ calcd. For $C_{27}H_{30}N_2O_3S$: 463.2055 found 463.2056.

Spectral data of N-(3-acetyl-3,4-dimethyl-3H-benzo[e]isoindol-1-yl)-N,4-dimethylbenzenesulfonamide (3h);

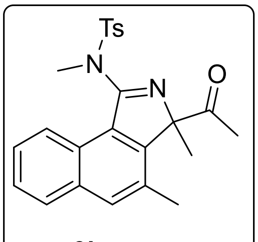

The compound was purified on a silica gel column using ethyl acetate/hexane: (20: 80) as the eluent; Yellow Semi-solid (37 mg, 0.08 mmol, 57%); ^{1}H NMR (400 MHz, $CDCl_3$): δ 8.97 (s, bs, 1H), 7.85 (d, *J* = 8 Hz, 3H), 7.65-7.73 (m, 2H), 7.55 (t, *J* = 8 Hz, 1H), 7.33 (d, *J* = 8 Hz, 2H), 3.25 (s, 3H), 2.46 (s, 3H), 2.44 (s, 3H), and 1.67 (s, 6H); ^{13}C NMR (100 MHz, $CDCl_3$): δ 202.5, 168.9, 153.6, 144.4, 134.5, 132.3, 131.6, 130.3, 129.7, 129.5, 129.1, 127.7, 127.2, 126.7, 126.5, 123.7, 84.3, 37.1, 23.6, 18.2, 16.7; HRMS (ESI-TOF) m/z: [M]+ calcd. For $C_{24}H_{24}N_2O_3S$: 443.1405 found 443.1404.

Spectral data of N-(3-acetyl-3-methyl-4-propyl-3H-benzo[e]isoindol-1-yl)-N,4-dimethylbenzenesulfonamide (3i);

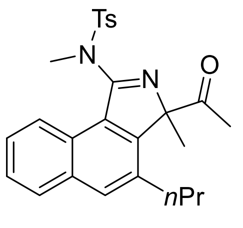

3i

The compound was purified on silica gel column using ethyl acetate/hexane: (20: 80) as the eluent; Yellow Semi-solid (36 mg, 0.08 mmol, 57%); ^{1}H NMR (700 MHz, $CDCl_3$): δ 8.99 (s, bs, 1H), 7.87 (d, *J* = 7 Hz, 2H), 7.75 (s, 2H), 7.65-7.68 (m, 1H), 7.55 (t, *J* = 7 Hz, 1H), 7.33 (d, *J* = 7 Hz, 2H), 3.24 (s, 3H), 2.74-2.79 (m, 1H), 2.67-2.71 (m, 1H), 2.43 (s, 3H), 1.69-1.75 (m, 2H), 1.63-1.68 (m, 4H), 1.59 (s, 2H) and 1.02 (t, *J* = 7 Hz, 3H); ^{13}C NMR (100 MHz, $CDCl_3$): δ 203.1, 168.8, 144.4, 135.1, 134.6, 131.7, 131.1, 129.5, 129.1, 127.9, 127.2, 126.6, 126.4, 123.7, 84.2, 37, 32.8, 24.2, 23.6, 21.6, 17.9, 14; HRMS (ESI-TOF) m/z: [M]+ calcd. For $C_{26}H_{28}N_2O_3S$: 471.1718 found 471.1724.

Spectral data of N-(3-acetyl-4-isopropyl-3-methyl-3H-benzo[e]isoindol-1-yl)-N,4-dimethylbenzenesulfonamide (3j);

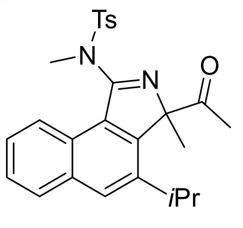

3j

The compound was purified on a silica gel column using ethyl acetate/hexane: (20: 80) as the eluent; MP 172-174 °C, White solid (38 mg, 0.08 mmol, 60%); ^{1}H NMR (700 MHz, $CDCl_3$):

δ 8.98 (s, bs, 1H), 7.90 (d, *J* = 7 Hz, 2H), 7.84 (s, 2H), 7.67 (t, *J* = 7 Hz, 1H), 7.55 (t, *J* = 7 Hz, 1H), 7.33 (d, *J* = 14 Hz, 2H), 3.24 (s, 3H), 3.19 (t, *J* = 7 Hz, 1H), 2.44 (s, 3H), 1.66 (s, 3H), 1.56 (s, 3H), 1.33 (d, *J* = 7 Hz, 3H) and 1.22 (d, *J* = 14 Hz, 3H); ^{13}C NMR (175 MHz, $CDCl_3$): δ 203.4, 168.7, 152.8, 144.4, 142.1, 134.9, 131.4, 129.4, 129.1, 128.5, 128, 127.2, 126.6, 123.7, 84.1, 37, 28.6, 25, 24.1, 21.6, 18.4; HRMS (ESI-TOF) m/z: [M+Na]+ calcd. For $C_{26}H_{28}N_2NaO_3S$: 471.1718 found 471.1718.

Spectral data of N-(3-acetyl-4-hexyl-3-methyl-3H-benzo[e]isoindol-1-yl)-N,4-dimethylbenzenesulfonamide (3k);

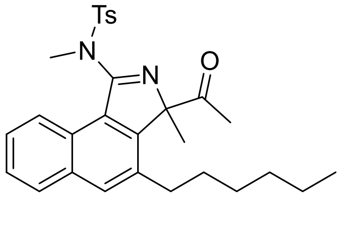

3k

The compound was purified on a silica gel column using ethyl acetate/hexane: (20: 80) as the eluent; White solid (30 mg, 0.06 mmol, 48%); ^{1}H NMR (700 MHz, $CDCl_3$): δ 8.98 (s, bs, 1H), 7.88 (d, *J* = 7 Hz, 2H), 7.77-7.82 (m, 2H), 7.66 (q, *J* = 7 Hz, 1H), 7.55 (t, *J* = 7 Hz, 1H), 7.33 (d, *J* = 14 Hz, 2H), 3.24 (s, 3H), 2.75-2.80 (m, 1H), 2.69-2.72 (m, 1H), 2.44 (s, 3H), 1.64-1.68 (m, 6H), 1.41 (d, *J* = 7 Hz, 3H) 1.32 (q, *J* = 7 Hz, 3H) and 0.89 (t, *J* = 7 Hz, 3H); ^{13}C NMR (100 MHz, $CDCl_3$): δ 203, 168.8, 153.5, 144.4, 135.4, 134.6, 131.7, 131.1, 129.5, 129.1, 127.9, 127.2, 126.6, 126.4, 123.7, 84.2, 37, 31.7, 31.1, 30.9, 29.3, 22.6, 21.6, 17.9, 14; HRMS (ESI-TOF) m/z: [M]+ calcd. For $C_{29}H_{34}N_2NaO_3S$: 513.2187 found 513.2186.

Spectral data of N-(3-acetyl-4-cyclohexyl-3-methyl-3H-benzo[e]isoindol-1-yl)-N,4-dimethylbenzenesulfonamide (3l);

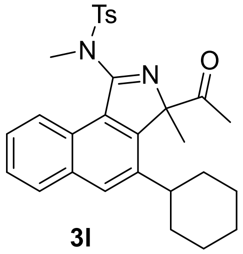

3l

The compound was purified on a silica gel column using ethyl acetate/hexane: (20: 80) as the eluent; MP 180-182 °C, White solid (26 mg, 0.05 mmol, 41%); ^{1}H NMR (700 MHz, $CDCl_3$): δ 8.98 (s, bs, 1H), 7.88 (d, *J* = 7 Hz, 2H), 7.80 (s, 2H), 7.65 (t, *J* = 7 Hz, 1H), 7.54 (t, *J* = 7 Hz, 1H), 7.33 (d, *J* = 7 Hz, 2H), 3.23 (s, 3H), 2.81 (s, 1H), 2.43 (s, 3H), 1.78-1.86 (m, 5H), 1.65 (s,

4H), 1.55 (t, *J* = 14 Hz, 3H), 1.38-1.46 (m, 3H) and 1.31 (d, *J* = 14 Hz, 1H); ^{13}C NMR (100 MHz, $CDCl_3$): δ 203.2, 168.7, 153, 144.4, 141, 134.8, 131.4, 129.5, 129.2, 129.1, 128.1, 128, 127.2, 126.6, 126.5, 123.7, 84.2, 39.2, 37.1, 35.4, 34.6, 26.7, 26, 21.6, 18.5; HRMS (ESI-TOF) m/z: [M]+ calcd. For $C_{29}H_{32}N_2O_3S$: 511.2031 found 511.2026.

Spectral data of N-(3-acetyl-4-butyl-3,8-dimethyl-3H-benzo[e]isoindol-1-yl)-N,4-dimethylbenzenesulfonamide (3m);

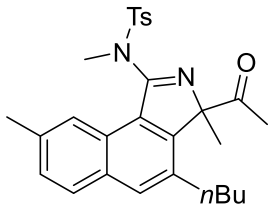

3m

The compound was purified on a silica gel column using ethyl acetate/hexane: (20: 80) as the eluent; Yellow Semi-solid (35 mg, 0.07 mmol, 56%); ^{1}H NMR (700 MHz, $CDCl_3$): δ 8.72 (s, bs, 1H), 7.72-7.90 (m, 4H), 7.38 (d, *J* = 7 Hz, 1H), 7.34 (d, *J* = 7 Hz, 2H), 3.25 (s, 3H), 2.75-2.79 (m, 1H), 2.68-2.72 (m, 1H), 2.62 (s, 3H), 2.45 (s, 3H), 1.60-1.63 (m, 3H), 1.42-1.48 (m, 3H), 1.26-1.31 (m, 1H), 1.22-1.24 (m, 1H), 0.97 (t, *J* = 7 Hz, 3H) and 0.87 (t, *J* = 7 Hz, 2H); ^{13}C NMR (175 MHz, $CDCl_3$): δ 203.2, 168.8, 153.4, 144.3, 137.1, 134.4, 132.9, 130.9, 129.5, 129.0, 128.8, 127.7, 126.6, 122.8, 84.1, 36.9, 33.2, 30.5, 22.7, 22.3, 21.6, 17.9, 13.9; HRMS (ESI-TOF) m/z: [M+Na]+ calcd. For $C_{28}H_{32}NaN_2O_3S$: 499.2031 found 499.2028.

Spectral data of N-(3-acetyl-4-butyl-8-chloro-3-methyl-3H-benzo[e]isoindol-1-yl)-N,4-dimethylbenzenesulfonamide (3n);

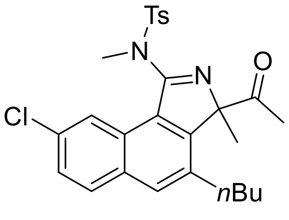

3n

The compound was purified on a silica gel column using ethyl acetate/hexane: (20: 80) as the eluent; MP 194-196 °C, white solid (29 mg, 0.05 mmol, 47%); ^{1}H NMR (400 MHz, $CDCl_3$): δ 8.96 (s, bs, 1H), 7.80 (d, *J* = 8 Hz, 2H), 7.72 (s, 2H), 7.48 (q, *J* = 8 Hz, 1H), 7.34 (d, *J* = 8 Hz, 2H), 3.23 (s, 3H), 2.70-2.79 (m, 2H), 2.44 (s, 3H), 1.55-1.66 (m, 7H) 1.41- 1.49 (m, 3H) and 0.9 (t, *J* = 8 Hz, 3H); ^{13}C NMR (100 MHz, $CDCl_3$): δ 202.6, 168.2, 154.3, 144.6, 135.8, 133.3, 132.8, 131, 130.8, 129.5, 129.3, 129, 127.6, 127, 123, 84.3, 37.1, 33.1, 30.5, 29.6, 23.5, 22.6,

21.6, 17.9, 13.9; HRMS (ESI-TOF) m/z: [M+Na] calcd. For $C_{27}H_{29}ClN_2NaO_3S$: 519.1485 found 519.1483.

Spectral data of N-(3-acetyl-4-butyl-7-chloro-3-methyl-3H-benzo[e]isoindol-1-yl)-N,4-dimethylbenzenesulfonamide (3o);

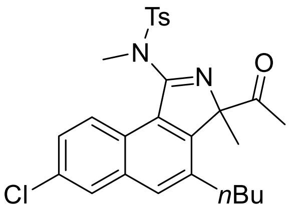

3o

The compound was purified on a silica gel column using ethyl acetate/hexane: (20: 80) as the eluent; MP 182-184 °C, White solid (33 mg, 0.06 mmol, 52%); ^{1}H NMR (400 MHz, $CDCl_3$): δ 8.96 (s, bs, 1H), 7.75-7.85 (m, 4H), 7.58 (q, J = 4 Hz, 1H), 7.33 (d, J = 8 Hz, 2H), 3.22 (s, 3H), 2.70-2.79 (m, 2H), 2.43 (s, 3H), 1.59-1.67 (m, 7H) 1.41- 1.47 (m, 3H) and 0.96 (t, J = 8 Hz, 3H);^{13}C NMR (100 MHz, $CDCl_3$): δ 202.5, 168.5, 153.5, 144.6, 136.7, 135.4, 132.5, 131.9, 130, 129.5, 129, 127.9, 126.5, 125.7, 124.7, 64.4, 37.1, 33.1, 30.6, 23.7, 22.6, 21.6, 17.9, 13.9; HRMS (ESI-TOF) m/z: [M+Na]+ calcd. For $C_{27}H_{29}ClN_2NaO_3S$: 519.1485 found 519.1482.

Spectral data of N-(3-acetyl-7-bromo-4-butyl-3-methyl-3H-benzo[e]isoindol-1-yl)-N,4-dimethylbenzenesulfonamide (3p);

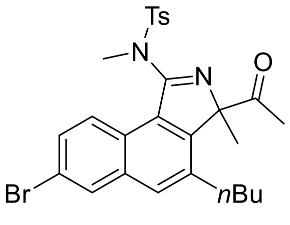

3p

The compound was purified on a silica gel column using ethyl acetate/hexane: (20: 80) as the eluent; Yellow Semi-solid (18 mg, 0.03 mmol, 30%); ^{1}H NMR (400 MHz, $CDCl_3$): δ 8.91 (s, bs, 1H), 8.05 (s, 1H), 7.61-7.85 (m, 4H), 7.33 (d, J = 4 Hz, 2H), 3.22 (s, 3H), 2.76-2.81 (m, 1H), 2.69-2.73 (m, 1H), 2.45 (s, 3H), 1.66 (s, 4H), 1.61 (t, J = 4 Hz, 4H), 1.44 (q, J = 7 Hz, 3H) and 0.97 (t, J = 8 Hz, 3H); ^{13}C NMR (175 MHz, $CDCl_3$): δ 202.4, 168.7, 153.8, 144.6, 136.6, 135.7, 134, 130.3, 129.9, 129.8, 129.5, 129, 124.9, 120.8, 84.4, 37.1, 33.1, 30.5, 22.6, 21.6, 17.8, 13.9; HRMS (ESI-TOF) m/z: [M+Na]+ calcd. For $C_{27}H_{29}BrN_2NaO_3S$: 563.0980 found 563.0978.

Spectral data of N-(4-butyl-3-methyl-3-propionyl-3H-benzo[e]isoindol-1-yl)-N-ethyl-4-methylbenzenesulfonamide (3r);

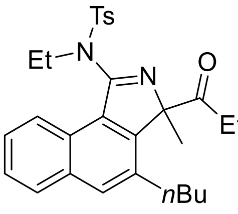

3r

The compound was purified on a silica gel column using ethyl acetate/hexane: (20: 80) as the eluent; Yellow oil (26 mg, 0.05 mmol, 40%); ^{1}H NMR (400 MHz, $CDCl_3$): for major isomer δ 9.14 (bs, 1H), 7.87 (d, J = 8 Hz, 2H), 7.74 (s, 1H), 7.64-7.68 (m, 2H), 7.52-7.56 (m, 1H), 7.30 (d, J = 4 Hz, 2H), 3.88 (d, J = 32 Hz, 2H), 2.70-2.77 (m, 2H), 2.42 (s, 3H), 1.87 (s, 1H), 1.64-1.72 (m, 2H), 1.58 (s, 3H), 1.42-1.50 (m, 2H), 1.22 (d, J = 4 Hz, 3H), 1.06 (s, 2H), 0.98 (t, J = 8 Hz, 3H), 0.95 (s, 2H) for minor isomer δ 9.07 (bs, 1H) and 3.62 (d, J = 32 Hz, 2H) rest of the peaks are merged with major isomer peaks; ^{13}C NMR (100 MHz, $CDCl_3$) for major isomer: δ 206.1, 168.4, 154.1, 144.2, 136.2, 135.8, 135.2, 134.6, 134.1, 132.9, 131.0, 129.5, 128.7, 128.5, 127.9, 127.2, 126.5, 124.0, 123.5, 84.2, 45.8, 33.3, 30.6, 29.6, 29.1, 24.6, 22.7, 21.6, 18.6, 18.2, 13.9, 12.9, 7.8, for minor isomer; 205.7, 167.4, 153.4 rest of the peaks are merged with major isomer peaks; HRMS (ESI-TOF) m/z: [M+Na]+ calcd. For $C_{29}H_{34}N_2NaO_3S$: 513.2187 found 513.2187.

Spectral data of N-(4-butyl-3-methyl-3-propionyl-3H-benzo[e]isoindol-1-yl)-N,4-dimethylbenzenesulfonamide;

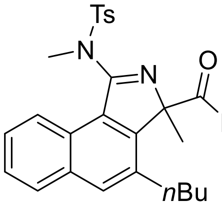

4b

The compound was purified on a silica gel column using ethyl acetate/hexane: (20: 80) as the eluent; Yellow Semi-solid (21 mg, 0.044 mmol, 31%); ^{1}H NMR (400 MHz, $CDCl_3$): δ 8.99 (s, bs, 1H), 7.74-7.88 (m, 4H), 7.64-7.68 (m, 1H), 7.52-7.56 (m, 1H), 7.32 (d, J = 8 Hz, 2H), 3.23 (s, 3H), 2.67-2.81 (m, 2H), 2.43 (s, 3H), 1.63-1.67 (m, 4H), 1.56-1.62 (m, 3H), 1.42-1.47 (m, 2H), 0.96 (t, J = 8 Hz, 3H), 0.86 (t, J = 8 Hz, 3H); ^{13}C NMR (100 MHz, $CDCl_3$): δ 205.8, 168.5, 153.6, 144.4, 135.4, 134.5, 131.5, 131, 129.4, 129, 127.9, 127.1, 126.6, 126.4, 123.7, 84.1,

37.1, 33.3, 31.5, 30.6, 28.8, 22.7, 21.6, 18.2, 14, 13.9, 7.9; HRMS (ESI-TOF) m/z: [M+Na]+ calcd. For $C_{28}H_{32}N_2NaO_3S$: 499.2031 found 499.2038.

Spectral data of N-(4-butyl-3-butyryl-3-methyl-3H-benzo[e]isoindol-1-yl)-N,4-dimethylbenzenesulfonamide (4c);

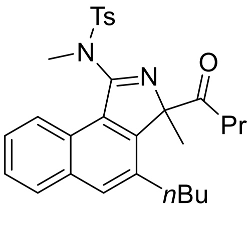

4c

The compound was purified on a silica gel column using ethyl acetate/hexane: (20: 80) as the eluent; Yellow Semi-solid (18 mg, 0.036 mmol, 27%); ^{1}H NMR (400 MHz, $CDCl_3$): δ 9.00 (s, bs, 1H), 7.75-7.89 (m, 4H), 7.67 (t, *J* = 4 Hz, 1H), 7.55 (q, *J*= 8 Hz, 1H), 7.32 (d, *J* = 8 Hz, 2H), 3.24 (t, *J* = 4 Hz, 3H), 2.75-2.82 (m, 1H), 2.65-2.73 (m, 1H), 2.43 (s, 3H), 1.62 (m, 6H), 1.26-1.50 (m, 2H), 1.28 (d, *J* = 16 Hz, 2H), 0.97 (t, *J* = 8 Hz, 4H) and 0.67 (s, 3H); ^{13}C NMR (100 MHz, $CDCl_3$): δ 205.2, 168.6, 153.6, 144.4, 135.4, 134.6, 131.7, 131.0, 129.8, 129.4, 129.1, 127.9, 127.4, 127.1, 126.6, 124.0, 84.2, 37.1, 33.3, 30.6, 25.8, 22.7, 21.9, 21.6, 18.2, 14.0, 13.7; HRMS (ESI-TOF) m/z: [M+Na]+ calcd. For $C_{29}H_{34}N_2NaO_3S$: 513.2187 found 513.2184.

Spectral data of N-(4-butyl-3-methyl-3-pentanoyl-3H-benzo[e]isoindol-1-yl)-N,4-dimethylbenzenesulfonamide (4d);

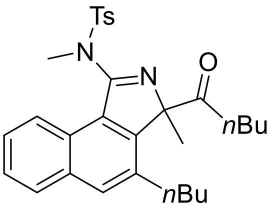

4d

The compound was purified on a silica gel column using ethyl acetate/hexane: (20: 80) as the eluent; MP 182-184 °C, White solid (21 mg, 0.041 mmol, 31%); ^{1}H NMR (700 MHz, $CDCl_3$): δ 9.00 (s, bs, 1H), 7.87 (d, *J* = 7 Hz, 3H), 7.76 (s, 1H), 7.68 (t, *J* = 7 Hz, 1H), 7.54 (t, *J*= 7 Hz, 1H), 7.33 (d, *J* = 7 Hz, 2H), 3.25 (s, 3H), 2.77-2.81 (m, 1H), 2.67-2.72 (m, 1H), 2.45 (s, 3H), 1.63-1.69 (m, 4H), 1.58-1.63 (m, 10H), 1.44-1.49 (m, 2H), and 0.97 (t, *J* = 7 Hz, 3H); ^{13}C NMR (175 MHz, $CDCl_3$): δ 205.1, 168.5, 153.5, 144.3, 135.3, 134.5, 131.6, 130.9, 129.7, 129.3, 129.0, 127.8, 127.3, 127.0, 126.5, 126.4, 123.9, 84.2, 37.0, 33.2, 30.5, 25.7, 22.6, 21.9, 21.5,

18.1, 13.9, 13.6; HRMS (ESI-TOF) m/z: [M+Na]+ calcd. For $C_{30}H_{36}N_2NaO_3S$: 527.2344 found 527.2348.

Spectral data of N-(4-butyl-3-methyl-3-(4-methylpentanoyl)-3H-benzo[e]isoindol-1-yl)-N,4-dimethylbenzenesulfonamide (4e);

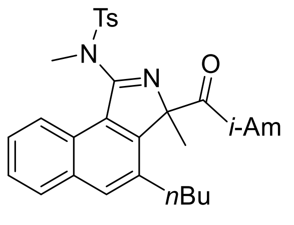

4e

The compound was purified on a silica gel column using ethyl acetate/hexane: (20: 80) as the eluent; Yellow Semi-solid (18 mg, 0.04 mmol, 30%);^{1}H NMR (400 MHz, $CDCl_3$): δ 8.99 (s, bs, 1H), 7.74-7.89 (m, 4H), 7.66 (s, 1H), 7.55 (d, *J*= 4 Hz, 1H), 7.32 (d, *J* = 8 Hz, 2H), 3.24 (s, 3H), 2.77-2.81 (m, 1H), 2.69-2.75 (m, 1H), 2.43 (s, 3H), 1.67 (s, 6H), 1.43-1.48 (s, 3H), 1.24 (s, 4H), 0.97 (t, *J* = 4 Hz, 4H), 0.84 (t, *J* = 8 Hz, 1H) and 0.73 (t, *J* = 4 Hz, 3H); ^{13}C NMR (100 MHz, $CDCl_3$): δ 205.4, 168.6, 153.6, 144.3, 135.4, 134.5, 131.6, 131, 129.8, 129.4, 129.1, 127.9, 127.4, 127.1, 126.6, 126.4, 84.3, 37, 33.3, 32.5, 31, 30.6, 29.6, 27.3, 23.3, 22.7, 22.3, 22, 21.6, 13.9, 13.7; HRMS (ESI-TOF) m/z: [M+Na]+ calcd. For $C_{31}H_{38}N_2NaO_3S$: 541.2500 found 541.2505.

Spectral data of N-(3-acetyl-4-butyl-3-propyl-3H-benzo[e]isoindol-1-yl)-N,4-dimethylbenzenesulfonamide;

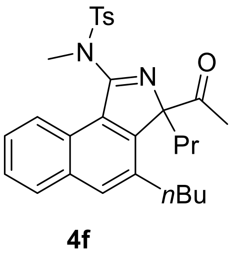

4f

The compound was purified on a silica gel column using ethyl acetate/hexane: (20: 80) as the eluent; Yellow Semi-solid (18 mg, 0.36 mmol, 26%); ^{1}H NMR (400 MHz, $CDCl_3$): δ 9.01 (s, bs, 1H), 7.75-7.89 (m, 4H), 7.67-7.73 (m, 1H), 7.52-7.56 (m, 1H), 7.32 (d, *J* = 8 Hz, 2H), 3.24 (s, 3H), 2.75-2.80 (m, 1H), 2.69-2.75 (m, 1H), 2.43 (s, 3H), 1.63-1.72 (m, 6H), 1.56-1.61 (m, 3H), 0.97 (t, *J* = 8 Hz, 4H) and 0.67 (s, 4H); ^{13}C NMR (100 MHz, $CDCl_3$): δ 205.3, 168.6, 153.6, 144.4, 135.4, 134.5, 131.7, 131, 130.5, 129.4, 129.1, 128.9, 128.2, 127.9, 127.1, 126.6,

126.4, 123.9, 84.2, 37.1, 35.2, 33.3, 30.6, 27.5, 25.7, 22.7, 21.9, 21.6, 18.2, 13.9, 13.7; HRMS (ESI-TOF) m/z: [M+Na]+ calcd. For $C_{29}H_{34}N_2NaO_3S$: 513.2187 found 513.2184.

Spectral data of N-(4-butyl-3-ethyl-3-propionyl-3H-benzo[e]isoindol-1-yl)-N,4-dimethylbenzenesulfonamide;

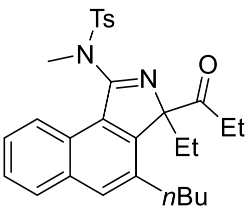

4g

The compound was purified on a silica gel column using ethyl acetate/hexane: (20: 80) as the eluent; Yellow Semi-solid (31 mg, 0.63 mmol, 46%); ^{1}H NMR (400 MHz, $CDCl_3$): δ 8.95 (d, *J* = 8 Hz, 1H), 7.88 (*J* = 8Hz, 3H), 7.73 (s, 1H), 7.65 (q, *J* = 8 Hz, 1H), 7.55 (q, *J* = 4 Hz, 1H), 7.33 (d, *J* = 8 Hz, 2H), 3.26 (s, 3H), 2.68-2.79 (m, 2H), 2.61 (t, *J* = 8 Hz, 1H), 2.44 (s, 3H), 2.36 (s, 1H), 1.57-1.64 (m, 6H), 1.42-1.51 (m, 3H), 0.96 (t, *J* = 8 Hz, 3H), and 0.85-0.90 (m, 3H); ^{13}C NMR (100 MHz, $CDCl_3$): δ 205.9, 168.8, 151.8, 144.2, 135.8, 134.5, 132.2, 130.9, 129.5, 129, 127.9, 127.4, 127.2, 126.5, 126.2, 123.4, 88, 37.1, 33, 30.4, 29.6, 24.8, 22.7, 21.6, 13.9, 13.6, 7.9; HRMS (ESI-TOF) m/z: [M+]+ calcd. For $C_{29}H_{34}N_2O_3S$: 491.2368 found 491.2369.

Spectral data of 1-(4-butyl-3-methyl-1-(methylamino)-3H-benzo[e]isoindol-3-yl)ethan-1-one (5a);

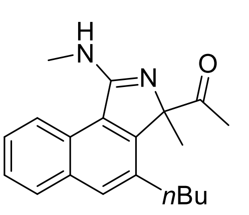

5a

The compound was purified on a silica gel column using Methanol/DCM: (10: 90) as the eluent; MP 160-162 °C, white solid (6.5 mg, 0.021 mmol, 32%); ^{1}H NMR (700 MHz, $CDCl_3$): δ 8.53 (d, *J* = 14 Hz, 1H), 7.96 (s, 1H), 7.92 (d, *J* = 7 Hz, 1H), 7.79 (t, *J* = 7 Hz, 1H), 7.61 (t, *J* = 7 Hz, 1H), 3.47 (s, 3H), 2.74-2.79 (m, 1H), 2.65-2.69 (m, 1H), 1.86 (s, 6H), 1.61-1.66 (m, 3H), 1.40-1.45 (m, 2H), and 0.94 (t, *J* = 7 Hz, 3H); ^{13}C NMR (175 MHz, $CDCl_3$): δ 201.4, 164.5, 148.1, 135.7, 135.3, 134.2, 129.5, 129, 127.7, 126.2, 122.6, 122, 76.2, 33.2, 31.5, 30,

23.2, 22.5, 19.2, 13.8; HRMS (ESI-TOF) m/z: [M]+ calcd. For $C_{20}H_{24}N_2O_1$: 309.1966 found 309.1967.

Spectral data of N-(4-acetyl-3-(2-(hex-1-yn-1-yl)phenyl)-5-methyl-1H-pyrrol-2-yl)-N,4-dimethylbenzenesulfonamide (6a);

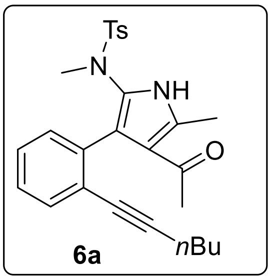

The compound was purified on a silica gel column using ethyl acetate/hexane: (20: 80) as the eluent; Yellow Semi-solid (46 mg, 0.09 mmol, 72%); ^{1}H NMR (400 MHz, $CDCl_3$): δ 9.17 (s, 1H), 7.43 (d, *J* = 8 Hz, 2H), 7.19-7.25 (m, 3H), 7.05-7.09 (m, 1H), 6.71-6.75 (m, 1H), 6.00 (d, *J* = 4 Hz, 1H), 2.87 (s, 3H), 2.49 (s, 3H), 2.42 (s, 3H), 2.16 (t, *J* = 8 Hz, 2H), 1.70 (s, 3H), 1.23 (q, *J* = 8 Hz, 2H), 1.12 (q, *J* = 8 Hz, 2H), and 0.77 (t, *J* = 8 Hz, 3H); ^{13}C NMR (100 MHz, $CDCl_3$): δ 195.6, 143.8, 137.1, 134.5, 133.2, 131.6, 130.1, 129.6, 127.7, 127.2, 126.8, 125.5, 122.8, 120.4, 119.3, 94.5, 79.6, 37.8, 30.3, 29.7, 21.6, 21.4, 18.9, 14.4, 13.5; HRMS (ESI-TOF) m/z: [M]+ calcd. For $C_{27}H_{30}N_2O_3S$: 463.2055 found 463.2055.

Spectral data of N-(4-acetyl-5-methyl-3-(2-(3-methylbut-1-yn-1-yl)phenyl)-1H-pyrrol-2-yl)-N,4-dimethylbenzenesulfonamide (6b);

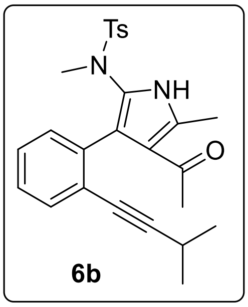

The compound was purified on a silica gel column using ethyl acetate/hexane: (20: 80) as the eluent; MP 190-192 °C, white solid (42 mg, 0.09 mmol, 66%); ^{1}H NMR (400 MHz, $CDCl_3$): δ 8.68 (s, 1H), 7.46 (d, *J* = 8 Hz, 2H), 7.25 (d, *J* = 8 Hz, 3H), 7.09 (t, *J* = 8 Hz, 1H), 6.74 (t, *J* = 8 Hz, 1H), 5.93 (d, *J* = 8 Hz, 1H), 2.84 (s, 3H), 2.51 (s, 3H), 2.48 (s, 1H), 2.46 (s, 3H), 1.70 (s, 3H), and 0.98 (d, *J* = 8 Hz, 6H); ^{13}C NMR (100 MHz, $CDCl_3$): δ 195.6, 143.9, 137.4, 134.4, 133.1, 131.2, 129.9, 129.6, 127.7, 127.3, 126.9, 125.5, 122.8, 120.5, 119.4, 100.2, 78.7, 37.7,

29.6, 22.6, 22.5, 21.6, 21, 14.4; HRMS (ESI-TOF) m/z: [M]+ calcd. For $C_{26}H_{28}N_2O_3S$: 449.1898 found 449.1897.

Spectral data of N-ethyl-N-(3-(2-(hex-1-yn-1-yl)phenyl)-5-methyl-4-propionyl-1H-pyrrol-2-yl)-4-methylbenzenesulfonamide (6c);

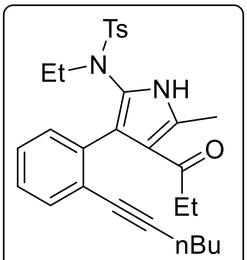

The compound was purified on a silica gel column using ethyl acetate/hexane: (20: 80) as the eluent; yellow oil (30 mg, 0.06 mmol, 46%); ^{1}H NMR (400 MHz, $CDCl_3$): δ 8.49 (s, 1H), 7.48 (d, *J* = 12 Hz, 2H), 7.22-7.26 (m, 3H), 7.08-7.12 (m, 1H), 6.73-6.77 (m, 1H), 5.98 (t, *J* = 8 Hz, 1H), 3.53-3.62 (m,1H), 2.90-2.97 (m, 1H), 2.51 (s, 3H), 2.45 (s, 3H), 2.15-2.18 (m, 2H), 2.0-2.06 (m, 1H), 1.81-1.89 (m, 1H), 1.26-1.32 (m, 2H), 1.02-1.07 (m, 3H), 0.87-0.92 (m, 2H), 0.80 (t, *J* = 8 Hz, 3H) and 0.73 (t, *J* = 8 Hz, 3H); ^{13}C NMR (100 MHz, $CDCl_3$): δ 198.3, 143.67, 136.9, 136.2, 132.8, 131.6, 129.9, 129.5, 127.5, 127.2, 126.9, 125.2, 120.8, 120.4, 120.3, 94.6, 79.4, 46.1, 41.1, 31.3, 30.3, 23.8, 22.3, 21.5, 18.9, 14.9, 14.5, 13.8, 13.5; HRMS (ESI-TOF) m/z: [M+Na]+ calcd. For $C_{29}H_{34}N_2NaO_3S$: 513.2187 found 513.2189.

(7) X-ray crystallographic data of compounds:

7.1 X-ray crystallographic structure and data of compound (3d):

Ellipsoid contour % probability level = 50%

Experimental: The sample was dissolved in the appropriate amount of ethyl acetate and added pentane to furnish a saturated solution. Afterward, the mixture was allowed to stand at room temperature to form the crystals.

Crystal Measurement:

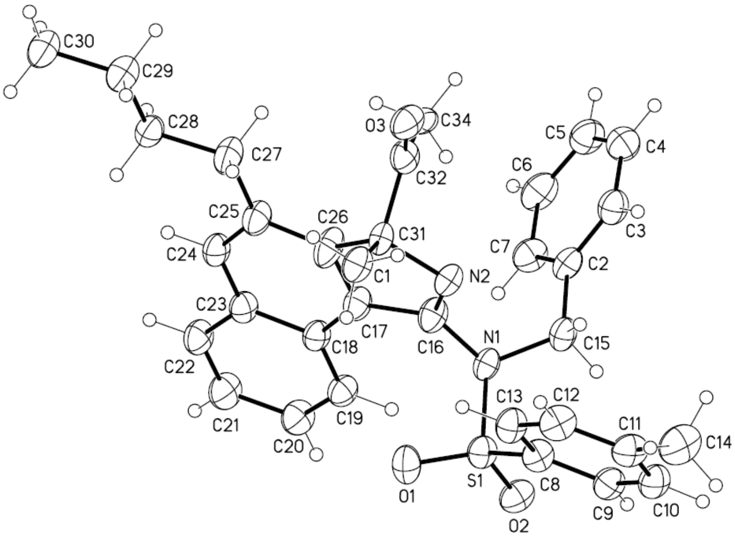

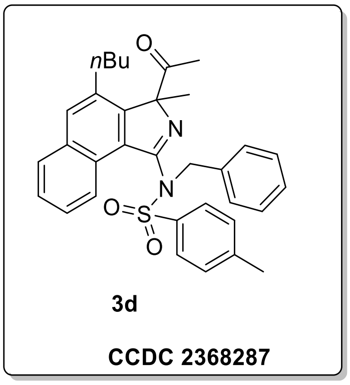

230762lt2_auto

Table S1 Crystal data and structure refinement for 230762lt2_auto.

Identification code	230762lt2_auto
Empirical formula	$C_{66}H_{68}N_4O_6S_2$
Formula weight	1077.36
Temperature/K	99.99(10)
Crystal system	triclinic
Space group	P-1
a/Å	9.9311(7)
b/Å	11.6958(7)
c/Å	14.2929(6)
α/°	67.541(5)
β/°	83.233(5)
γ/°	66.372(6)
Volume/Å^3	1404.39(16)
Z	1
ρ_{calc}g/cm^3	1.274
μ/mm^{-1}	1.313
F(000)	572.0
Crystal size/mm^3	0.19 × 0.02 × 0.02
Radiation	Cu Kα (λ = 1.54184)
2Θ range for data collection/°	6.698 to 149.688
Index ranges	-12 ≤ h ≤ 11, -14 ≤ k ≤ 14, -17 ≤ l ≤ 17
Reflections collected	16354
Independent reflections	5405 [R_{int} = 0.0538, R_{sigma} = 0.0525]
Data/restraints/parameters	5405/0/414
Goodness-of-fit on F^2	1.070
Final R indexes [I>=2σ (I)]	R_1 = 0.0706, wR_2 = 0.1924
Final R indexes [all data]	R_1 = 0.0818, wR_2 = 0.1999
Largest diff. peak/hole / e Å^{-3}	0.63/-0.61

7.2 X-ray crystallographic structure and data of compound (5a):

Ellipsoid contour % probability level = 50%

Experimental: The sample was dissolved in the appropriate amount of DCM Afterward; the mixture was allowed to stand at room temperature to form the crystals.

Crystal Measurement:

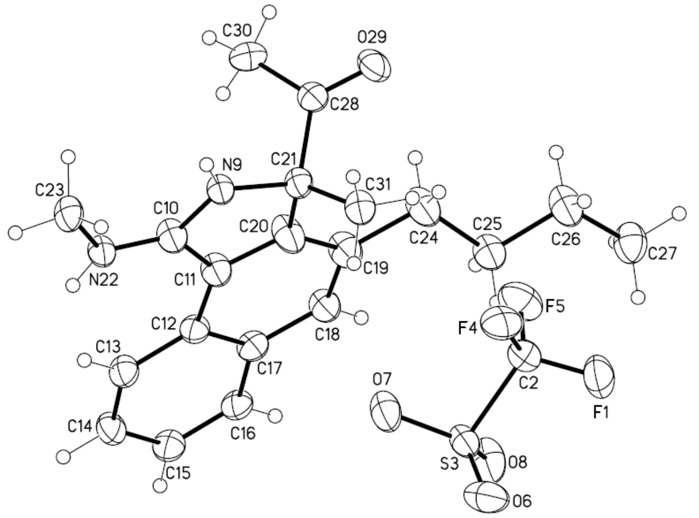

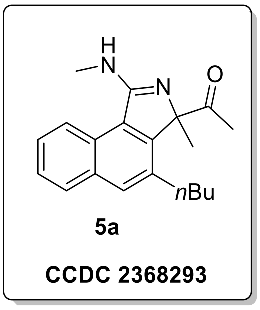

240543lt2_auto

Table S1 Crystal data and structure refinement for 240543lt2_auto.

Identification code	240543lt2_auto
Empirical formula	$C_{21}H_{25}F_3N_2O_4S$
Formula weight	458.49
Temperature/K	100.01(10)
Crystal system	monoclinic
Space group	$P2_1/c$
a/Å	8.6058(6)
b/Å	14.5871(10)
c/Å	17.0489(12)
α/°	90
β/°	90.434(2)
γ/°	90
Volume/Å^3	2140.1(3)
Z	4
ρ_{calc}g/cm^3	1.423
μ/mm^{-1}	1.856
F(000)	960.0
Crystal size/mm^3	0.14 × 0.11 × 0.04
Radiation	Cu Kα (λ = 1.54184)

2Θ range for data collection/°	7.976 to 134.138
Index ranges	$-10 \le h \le 10$, $-17 \le k \le 16$, $-20 \le l \le 20$
Reflections collected	11189
Independent reflections	3792 [R_{int} = 0.0332, R_{sigma} = 0.0362]
Data/restraints/parameters	3792/185/341
Goodness-of-fit on F^2	1.177
Final R indexes [I>=2σ (I)]	R_1 = 0.0595, wR_2 = 0.1476
Final R indexes [all data]	R_1 = 0.0649, wR_2 = 0.1503
Largest diff. peak/hole / e Å^{-3}	0.43/-0.26

7.3 X-ray crystallographic structure and data of compound (6b):

Ellipsoid contour % probability level = 50%

Experimental: The sample was dissolved in the appropriate amount of Ethyl acetate, afterward; the mixture was allowed to stand at room temperature to form the crystals.

Crystal Measurement:

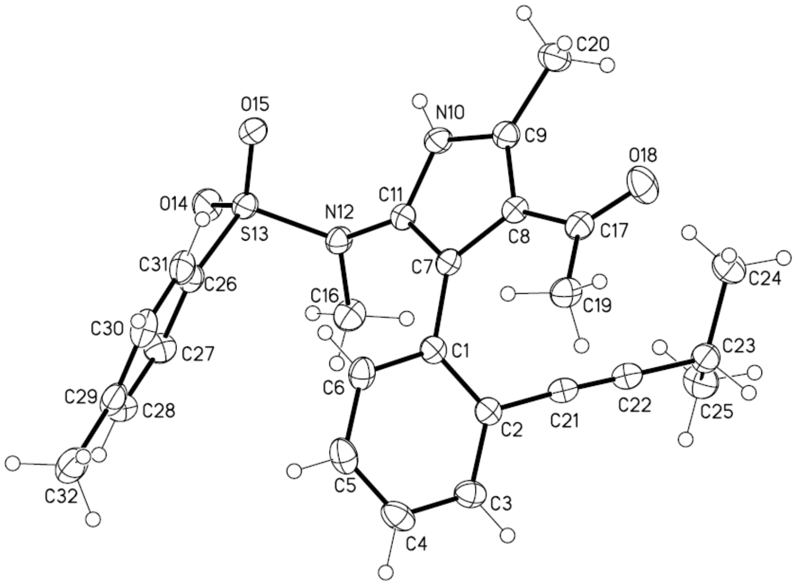

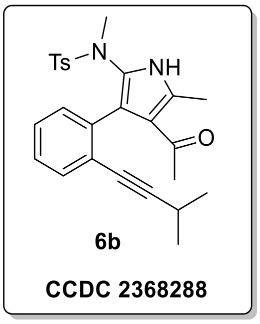

240220lt_auto

Table S1 Crystal data and structure refinement for 240220lt_auto.

Identification code	240220lt_auto
Empirical formula	$C_{26}H_{28}N_2O_3S$
Formula weight	448.56

Temperature/K	100.01(10)
Crystal system	triclinic
Space group	P-1
a/Å	10.10748(17)
b/Å	10.6583(2)
c/Å	12.4712(3)
α/°	110.189(2)
β/°	94.6826(15)
γ/°	108.5163(18)
Volume/Å^3	1167.76(4)
Z	2
ρ_{calc}g/cm^3	1.276
μ/mm^{-1}	1.470
F(000)	476.0
Crystal size/mm^3	0.17 × 0.13 × 0.13
Radiation	Cu Kα (λ = 1.54184)
2Θ range for data collection/°	7.734 to 148.618
Index ranges	$-12 \le h \le 10$, $-12 \le k \le 13$, $-15 \le l \le 15$
Reflections collected	15203
Independent reflections	4508 [R_{int} = 0.0148, R_{sigma} = 0.0153]
Data/restraints/parameters	4508/0/296
Goodness-of-fit on F^2	1.060
Final R indexes [I>=2σ (I)]	R_1 = 0.0313, wR_2 = 0.0826
Final R indexes [all data]	R_1 = 0.0326, wR_2 = 0.0835
Largest diff. peak/hole / e Å^{-3}	0.32/-0.34

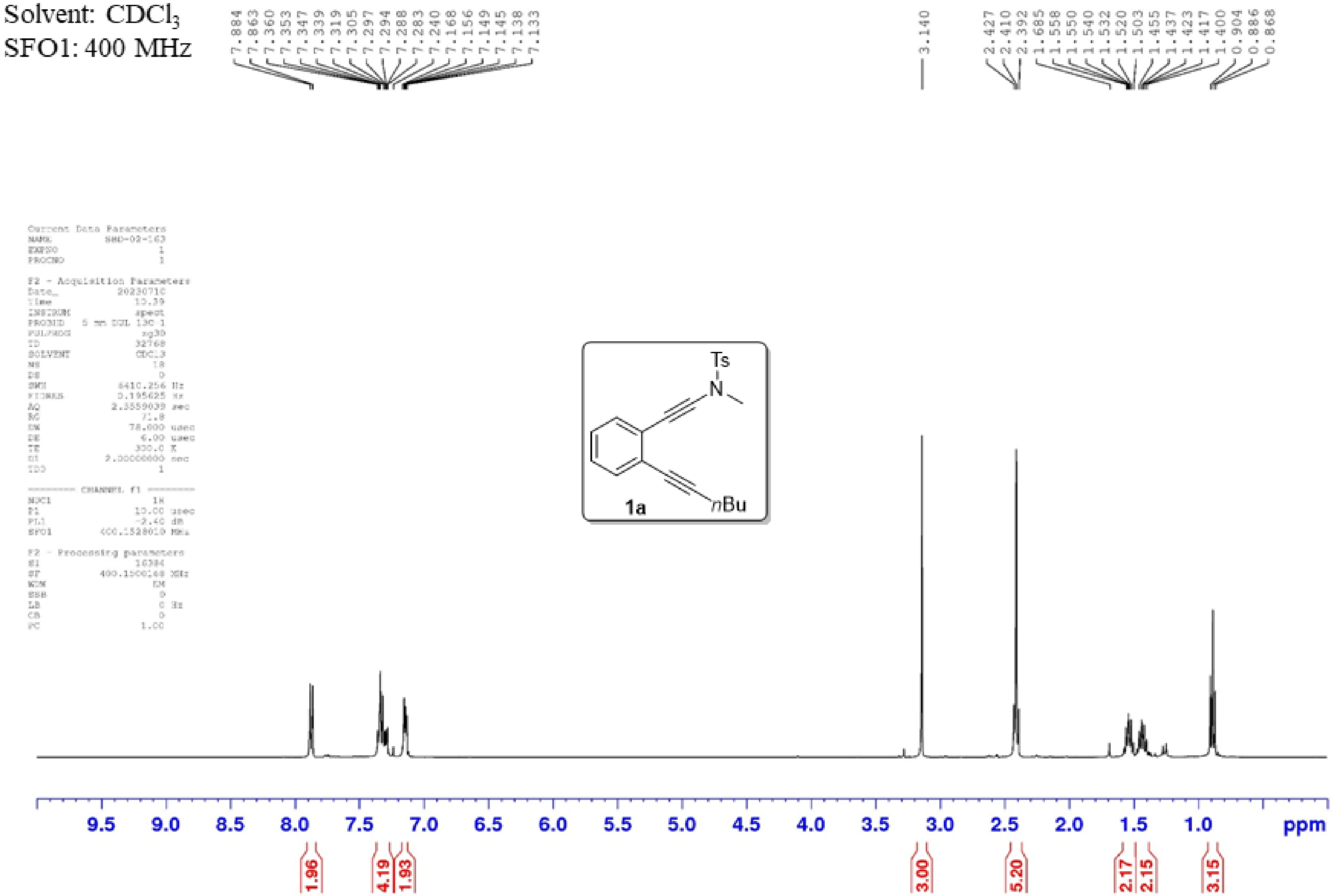
Solvent: CDCl3
SFO1: 400 MHz
7.884 7.863 7.360 7.353 7.347 7.339 7.319 7.305 7.297 7.294 7.288 7.283 7.240 7.168 7.156 7.149 7.145 7.138 7.133
3.140
2.427 2.410 2.392 1.685 1.558 1.550 1.540 1.532 1.520 1.503 1.455 1.437 1.423 1.417 1.400 0.904 0.886 0.868
Ts
N
1a
nBu
9.5 9.0 8.5 8.0 7.5 7.0 6.5 6.0 5.5 5.0 4.5 4.0 3.5 3.0 2.5 2.0 1.5 1.0 ppm
1.96 4.19 1.93 3.00 5.20 2.17 2.15 3.15

Solvent: $CDCl_3$
SFO1: 100 MHz

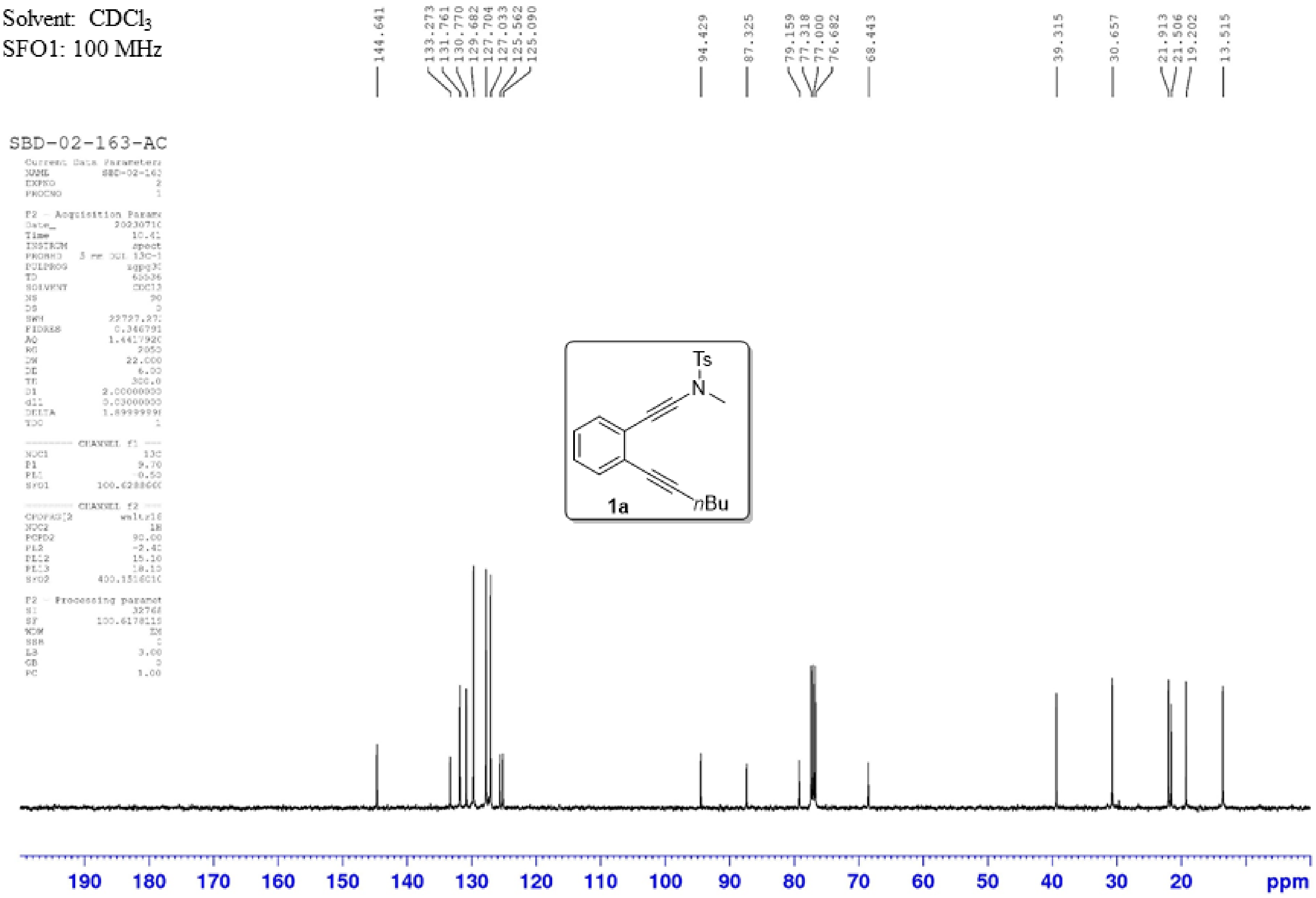

Solvent: $CDCl_3$
SFO1: 400 MHz

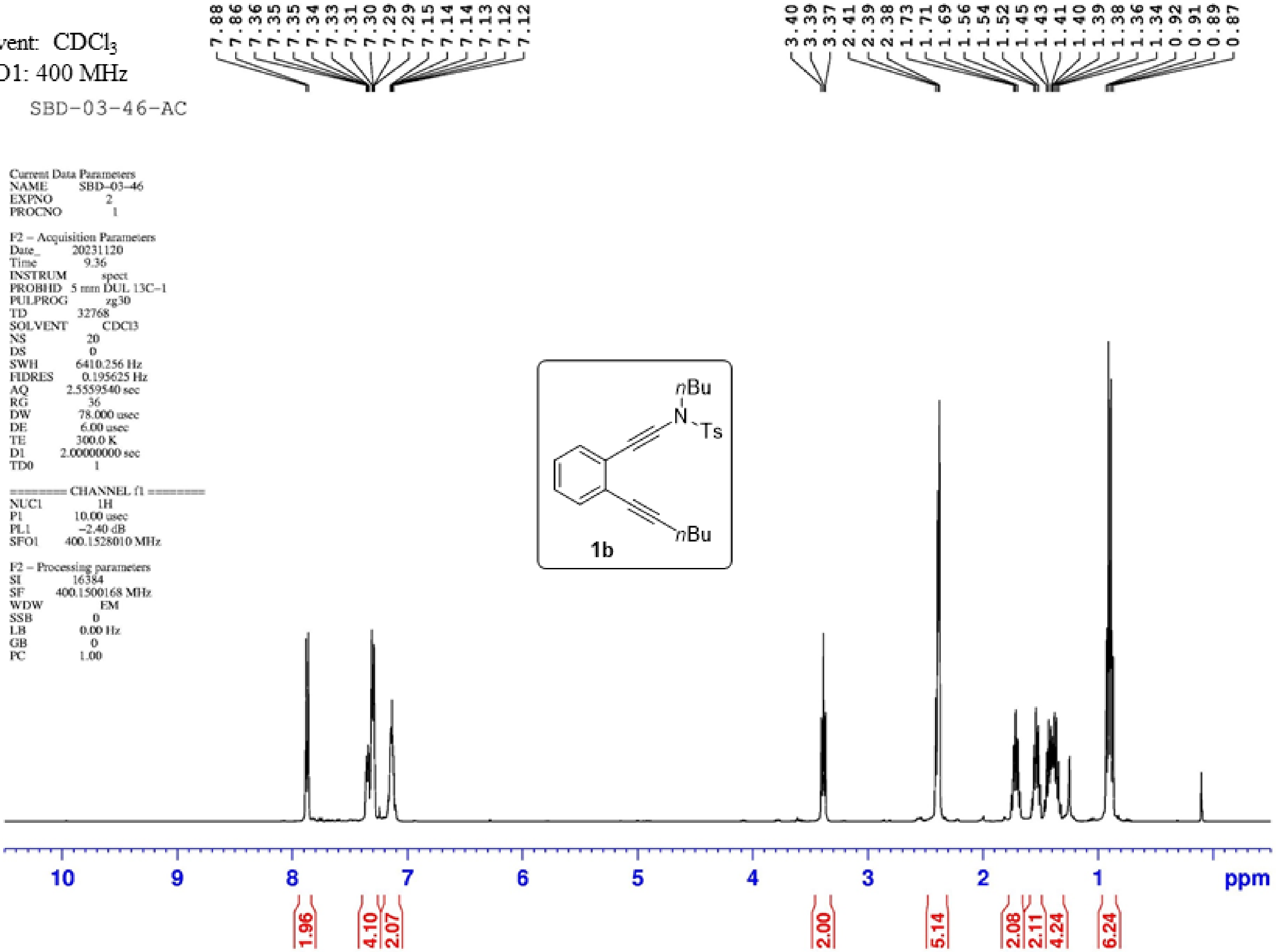

Solvent: $CDCl_3$
SFO1: 100 MHz

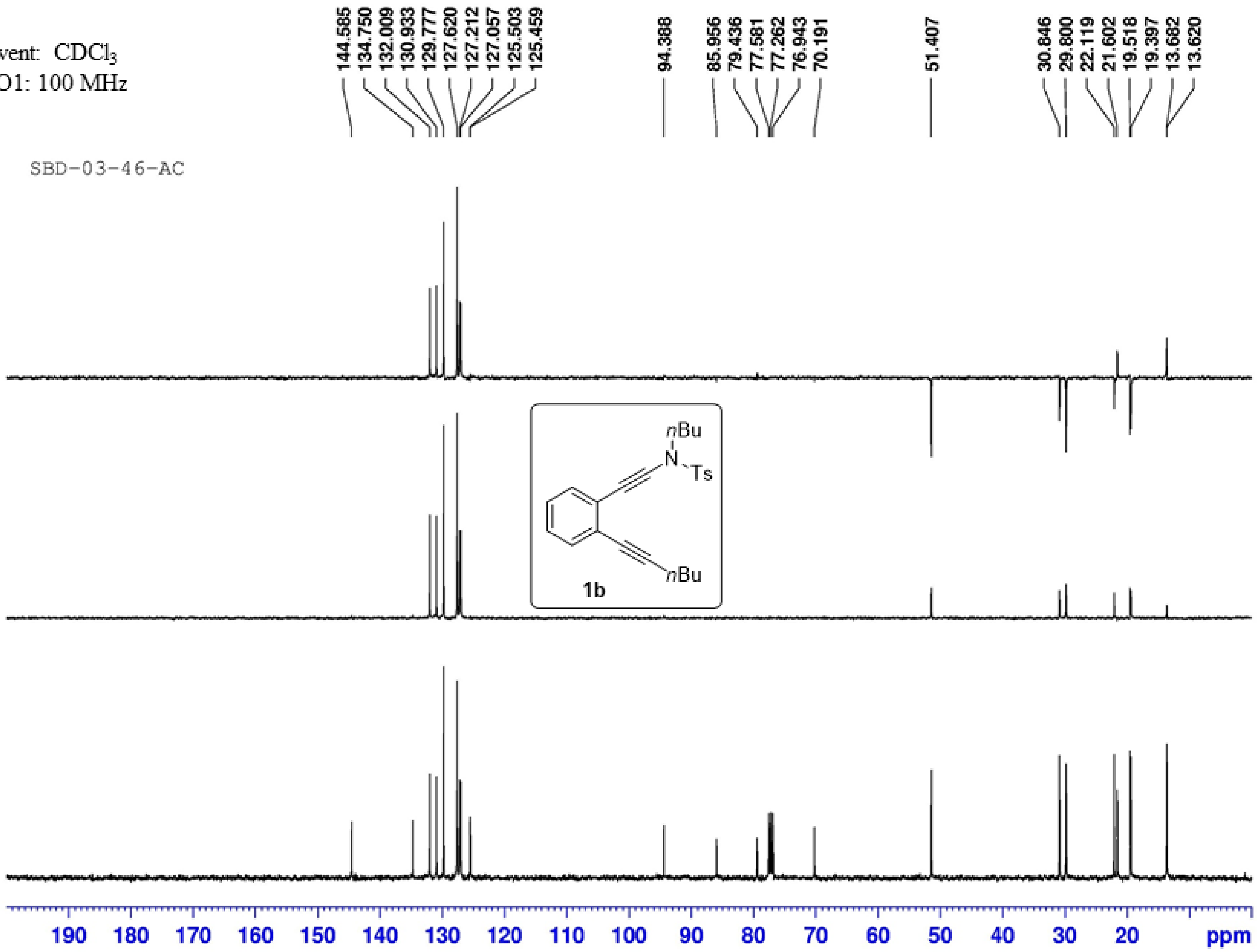

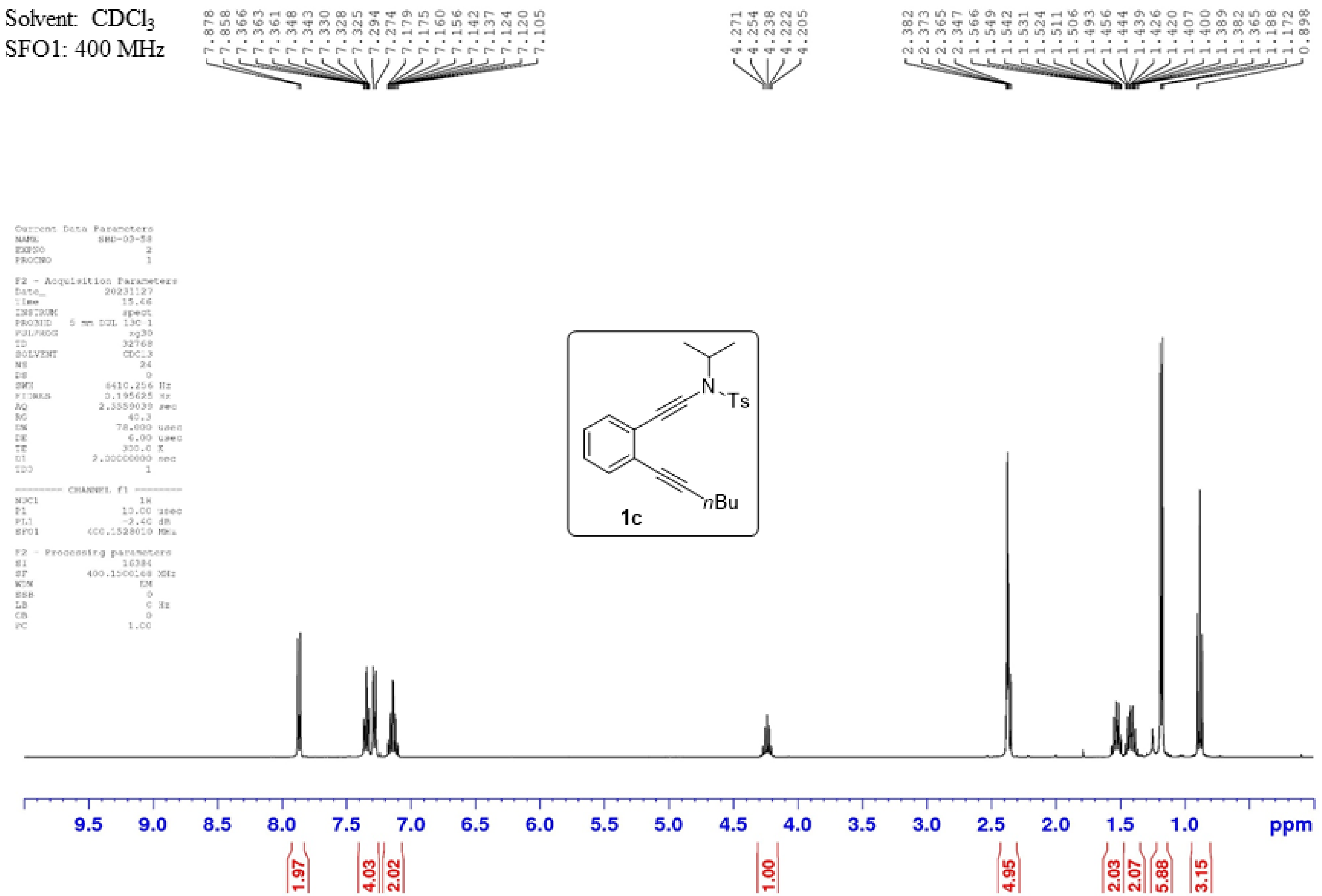

Solvent: CDCl3
SFO1: 400 MHz
7.878 7.858 7.366 7.363 7.361 7.348 7.343 7.330 7.328 7.325 7.294 7.274 7.179 7.175 7.160 7.156 7.142 7.137 7.124 7.120 7.105
4.271 4.254 4.238 4.222 4.205
2.382 2.373 2.365 2.347 1.566 1.549 1.542 1.531 1.524 1.511 1.506 1.493 1.456 1.444 1.439 1.426 1.420 1.407 1.400 1.389 1.382 1.365 1.188 1.172 0.898
N
Ts
nBu
1c
9.5 9.0 8.5 8.0 7.5 7.0 6.5 6.0 5.5 5.0 4.5 4.0 3.5 3.0 2.5 2.0 1.5 1.0 ppm
1.97 4.03 2.02 1.00 4.95 2.03 2.07 5.88 3.15

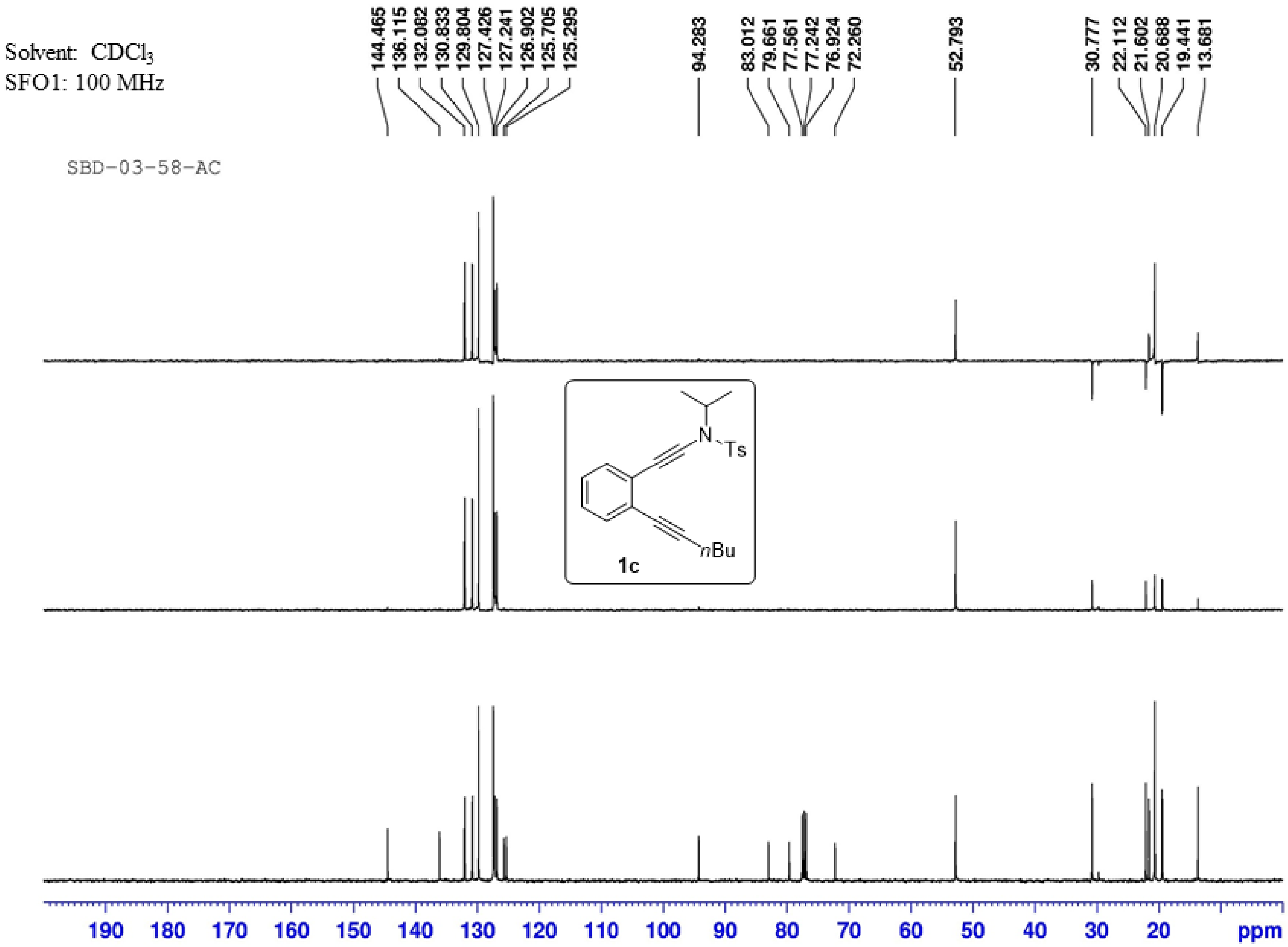
Solvent: $CDCl_3$
SFO1: 100 MHz
SBD-03-58-AC
144.465
136.115
132.082
130.833
129.804
127.426
127.241
126.902
125.705
125.295
94.283
83.012
79.661
77.561
77.242
76.924
72.260
52.793
30.777
22.112
21.602
20.688
19.441
13.681
N
Ts
nBu
1c
190 180 170 160 150 140 130 120 110 100 90 80 70 60 50 40 30 20 ppm

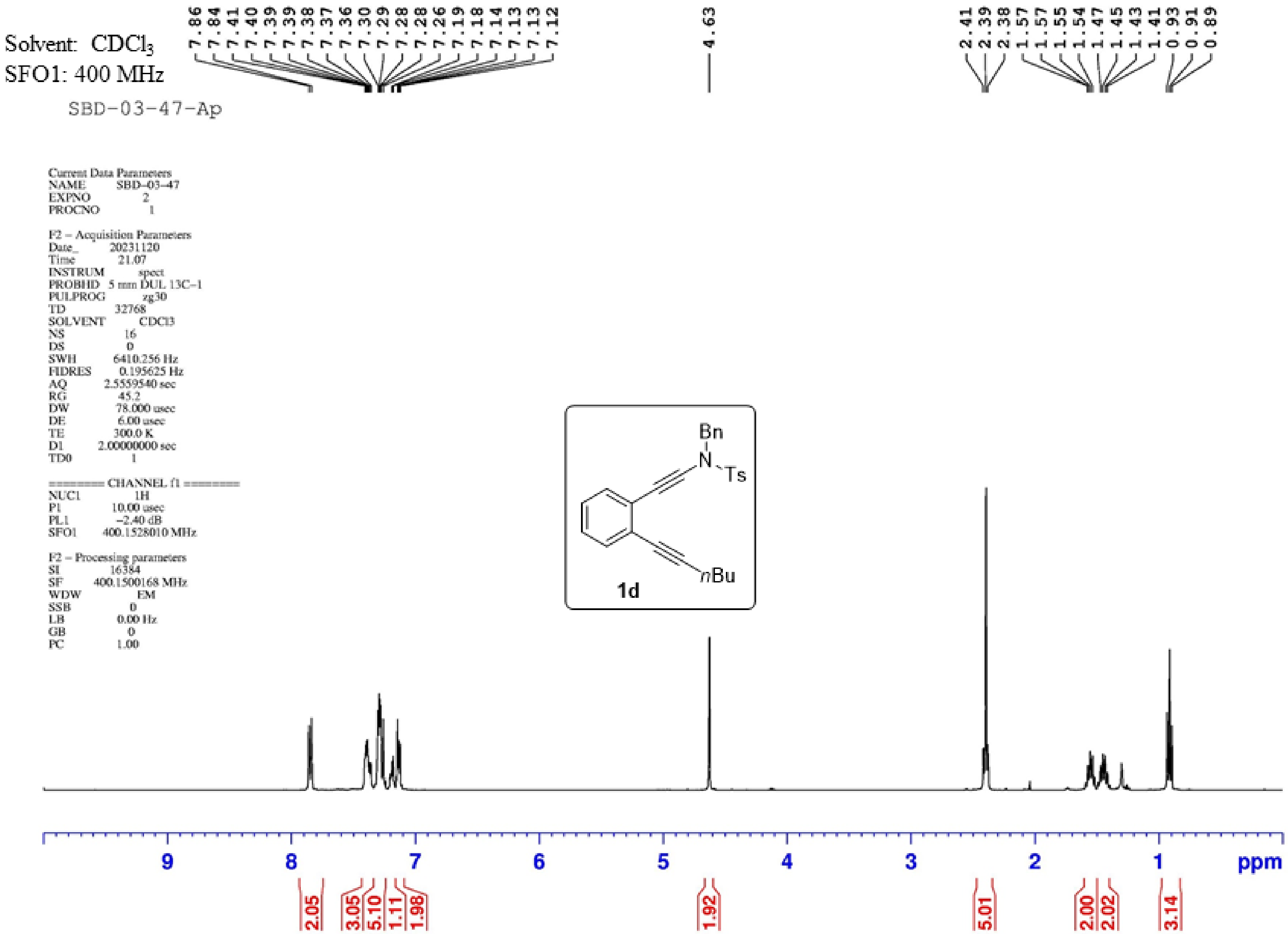
Solvent: $CDCl_3$
SFO1: 400 MHz
SBD-03-47-Ap
7.86 7.84 7.41 7.40 7.39 7.39 7.38 7.37 7.36 7.30 7.29 7.28 7.26 7.19 7.18 7.14 7.13 7.13 7.12
4.63
2.41 2.39 2.38 1.57 1.57 1.55 1.54 1.47 1.45 1.43 1.41 0.93 0.91 0.89
Current Data Parameters
NAME SBD-03-47
EXPNO 2
PROCNO 1
F2 - Acquisition Parameters
Date_ 20231120
Time 21.07
INSTRUM spect
PROBHD 5 mm DUL 13C-1
PULPROG zg30
TD 32768
SOLVENT CDCl3
NS 16
DS 0
SWH 6410.256 Hz
FIDRES 0.195625 Hz
AQ 2.5559540 sec
RG 45.2
DW 78.000 usec
DE 6.00 usec
TE 300.0 K
D1 2.00000000 sec
TD0 1
======== CHANNEL f1 ========
NUC1 1H
P1 10.00 usec
PL1 -2.40 dB
SFO1 400.1528010 MHz
F2 - Processing parameters
SI 16384
SF 400.1500168 MHz
WDW EM
SSB 0
LB 0.00 Hz
GB 0
PC 1.00
Bn
N
Ts
nBu
1d
9 8 7 6 5 4 3 2 1 ppm
2.05 3.05 5.10 1.11 1.98 1.92 5.01 2.00 2.02 3.14

Solvent: $CDCl_3$
SFO1: 100 MHz

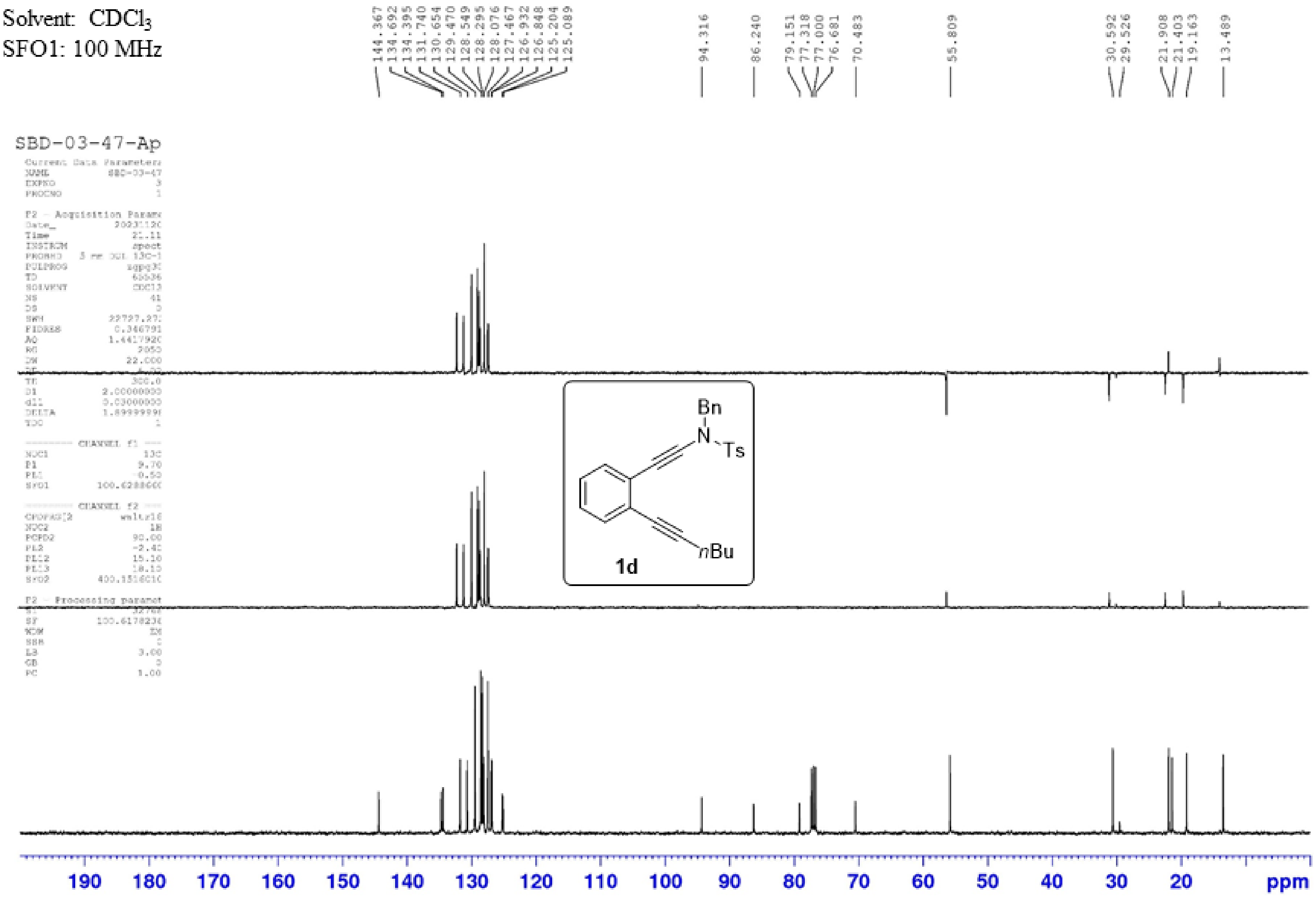

Solvent: $CDCl_3$
SFO1: 400 MHz

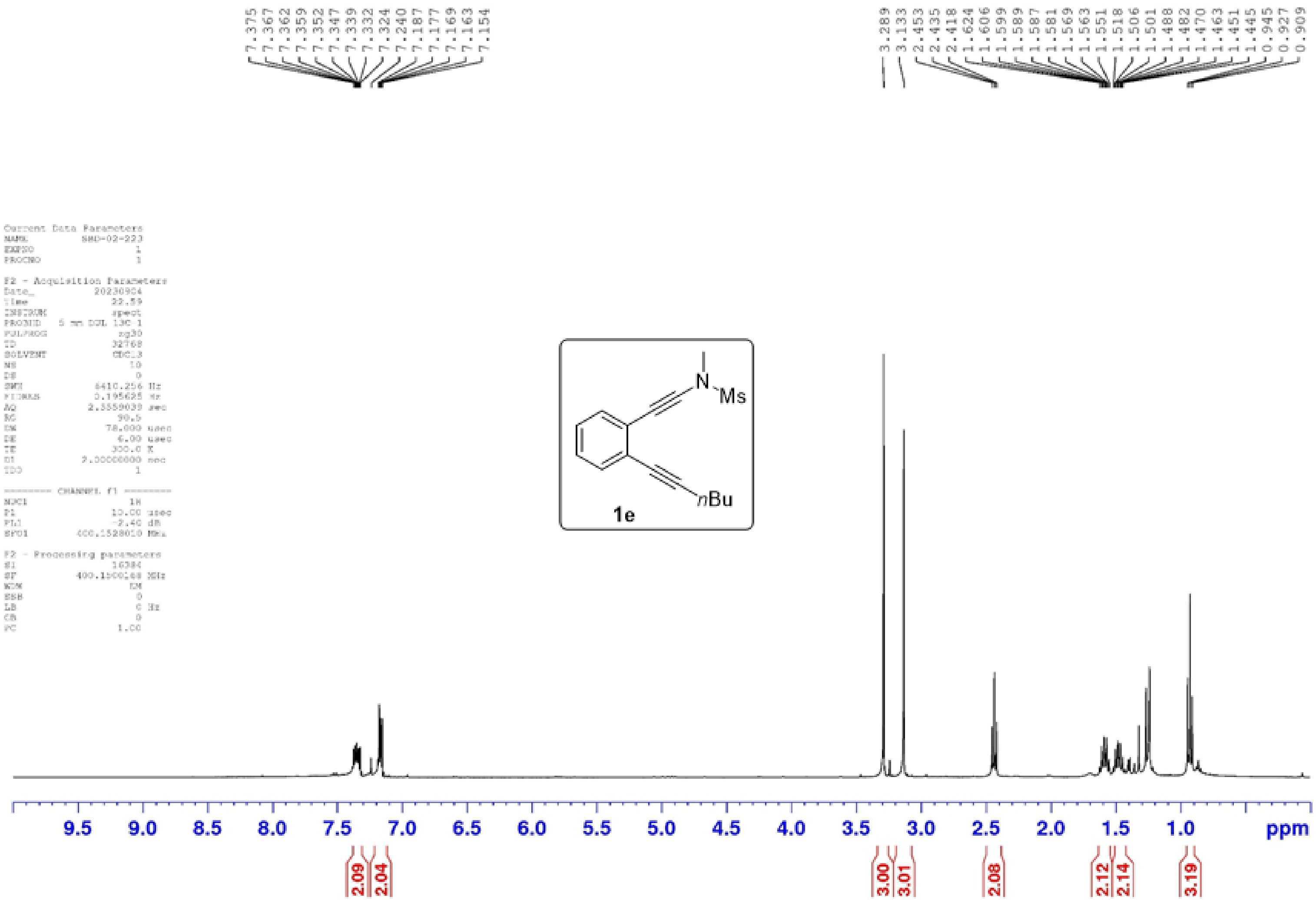

Solvent: $CDCl_3$
SFO1: 100 MHz

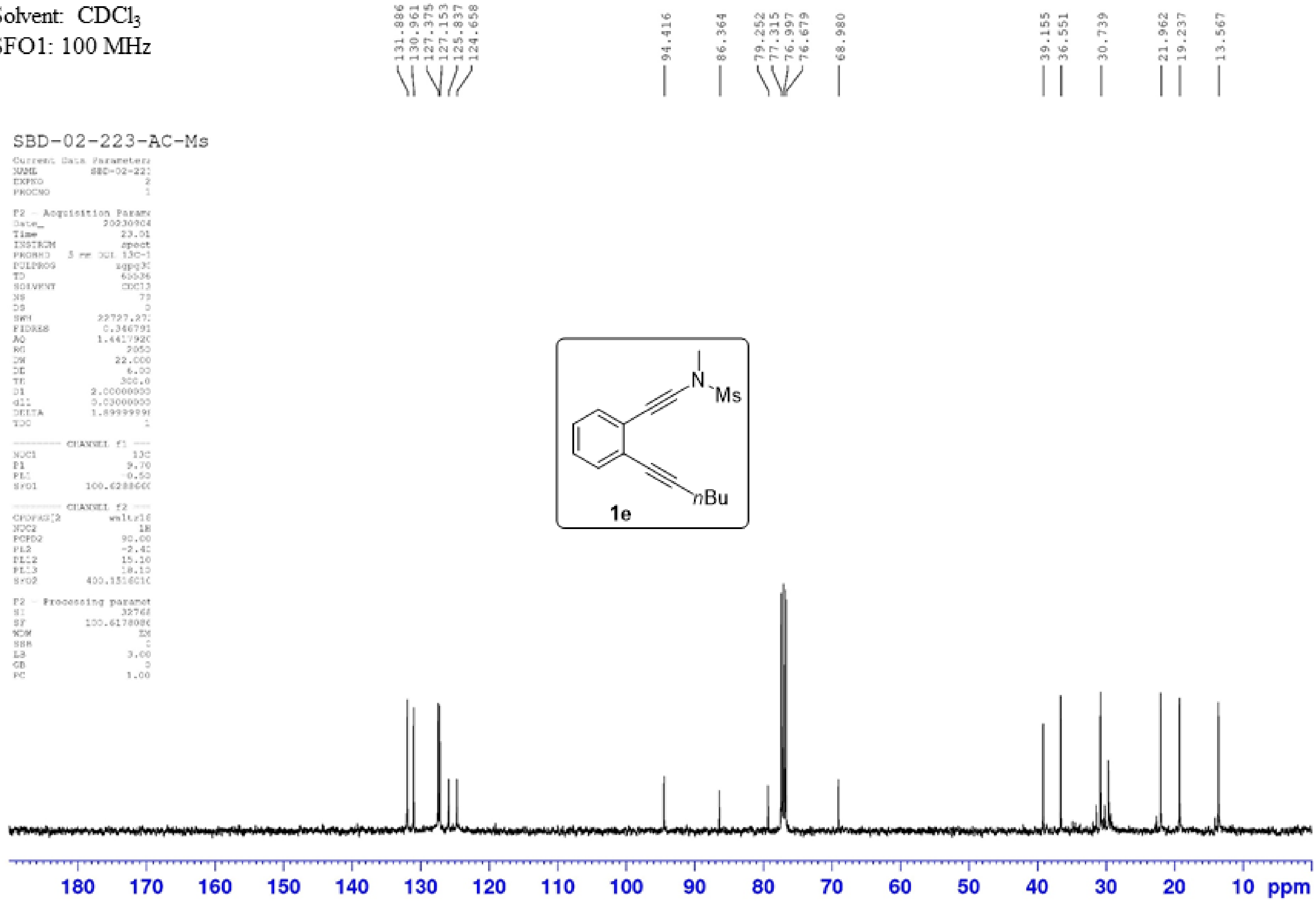

1e

Solvent: $CDCl_3$
SFO1: 400 MHz

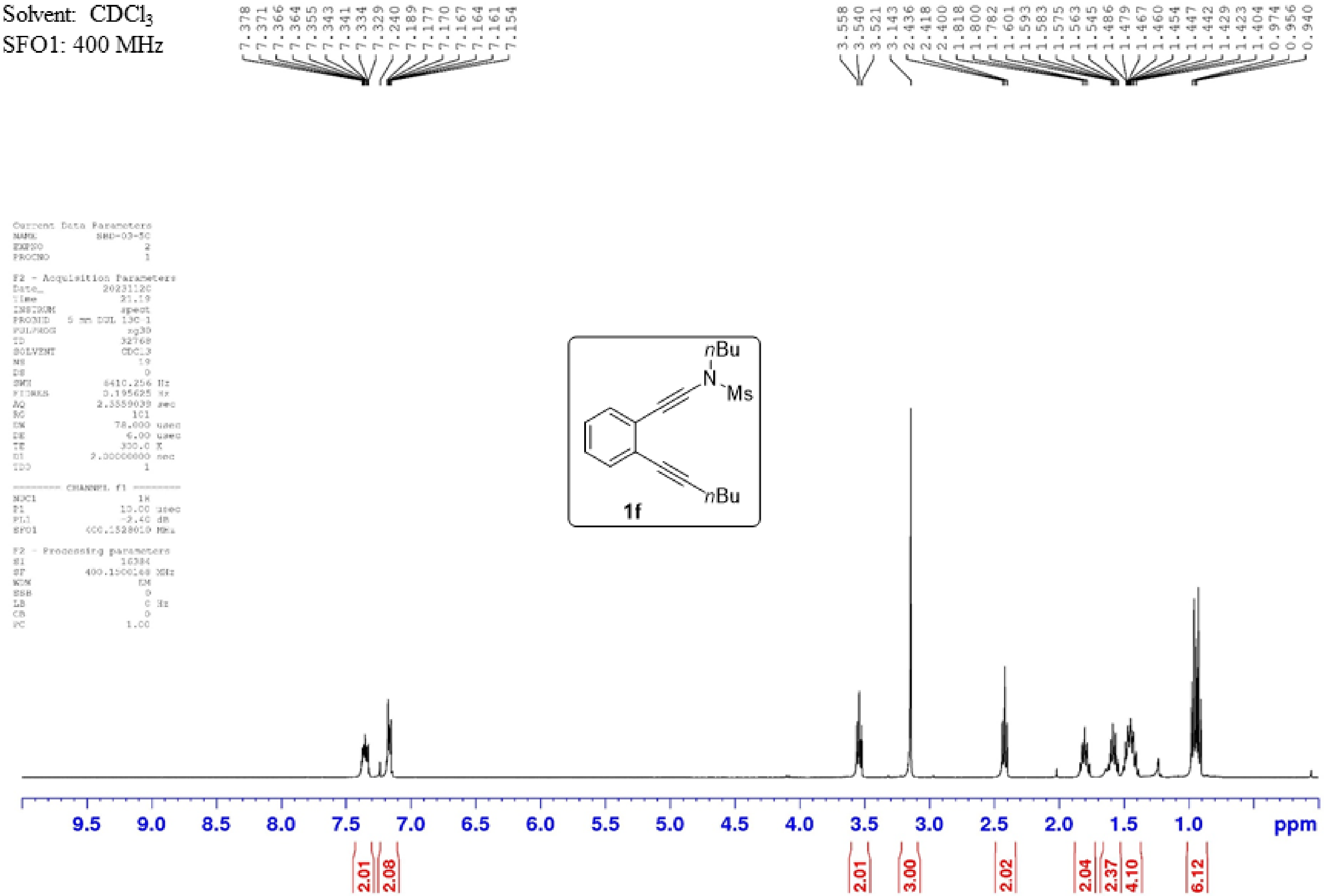

Solvent: $CDCl_3$
SFO1: 100 MHz

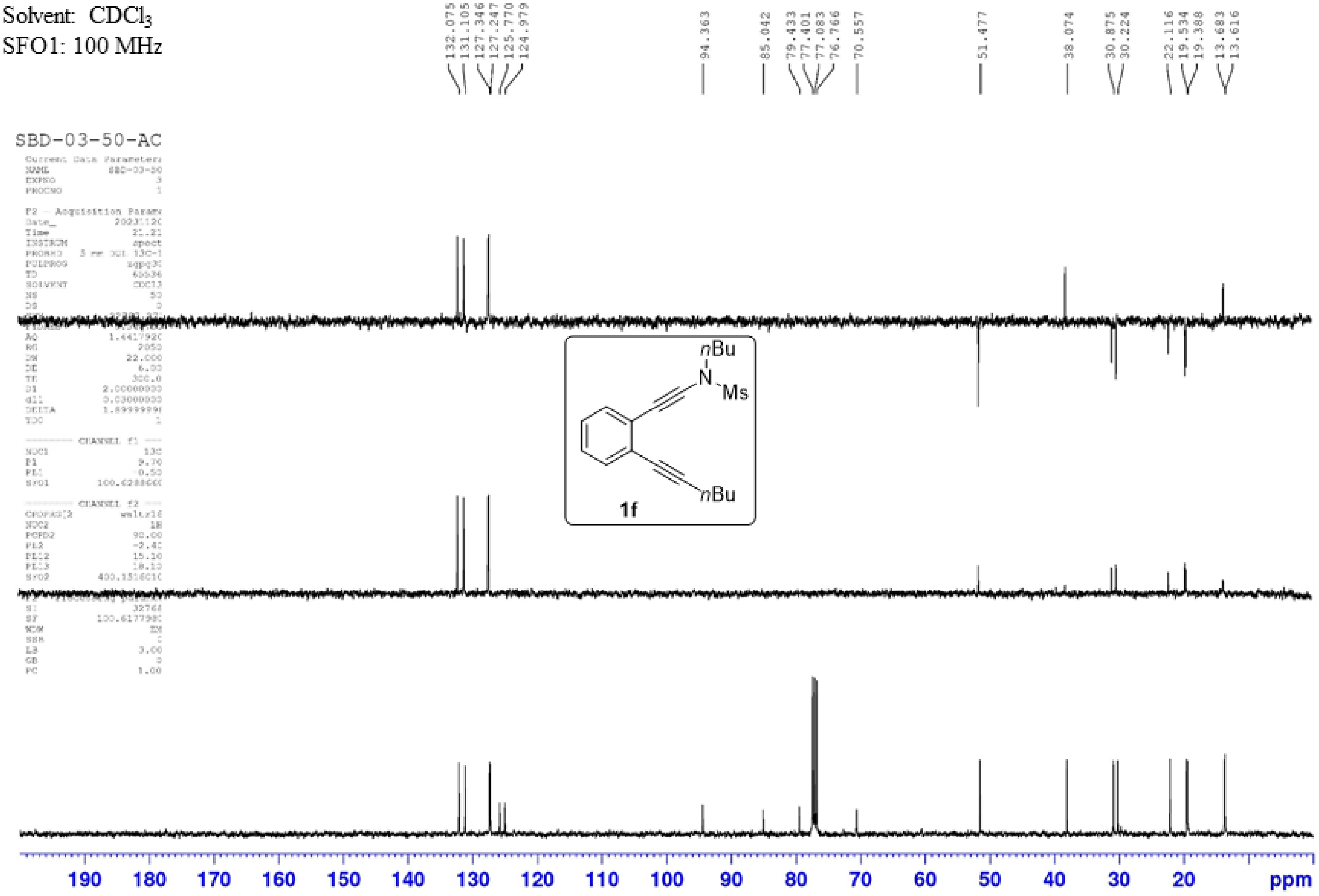

Solvent: $CDCl_3$
SFO1: 400 MHz

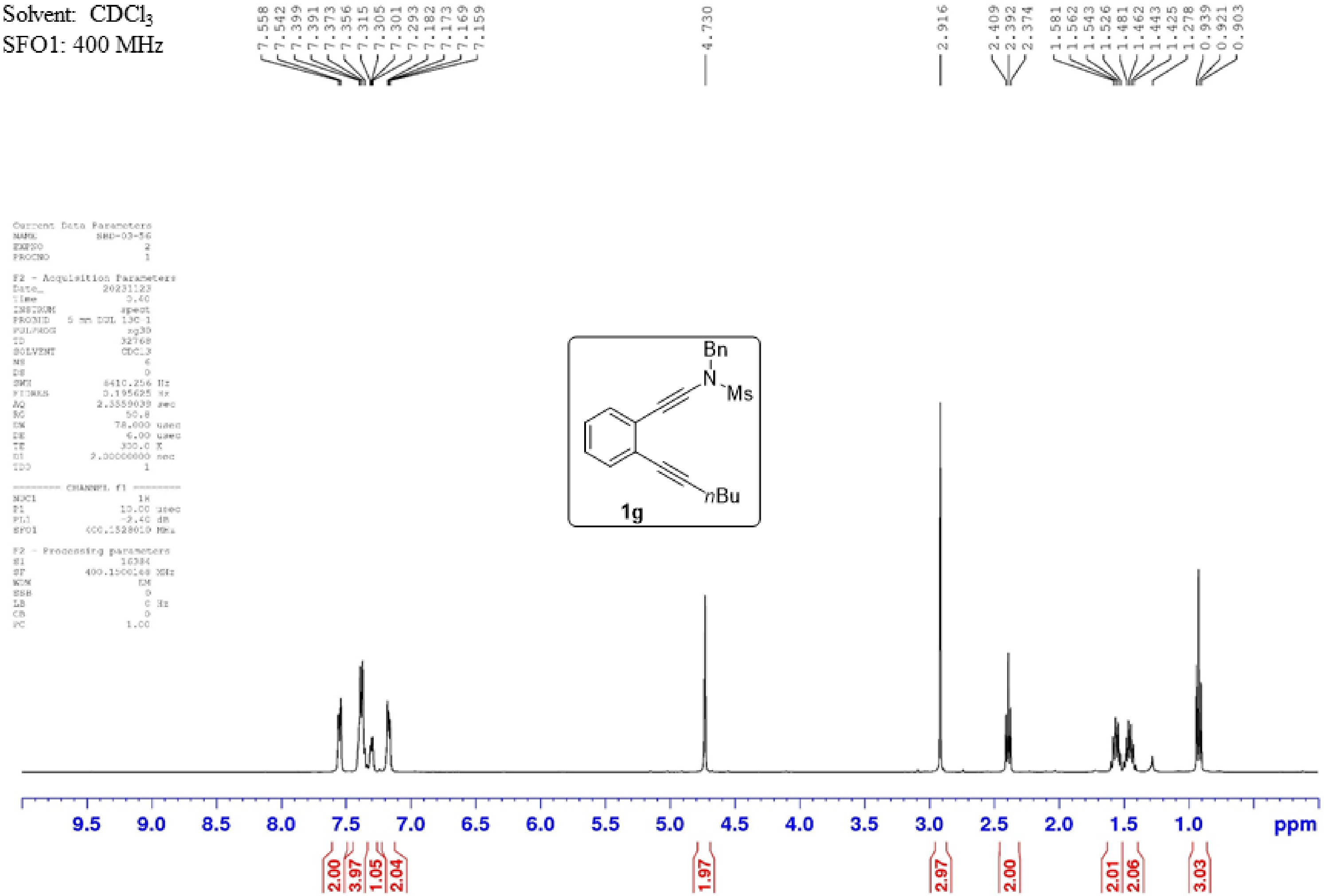

Solvent: $CDCl_3$
SFO1: 100 MHz

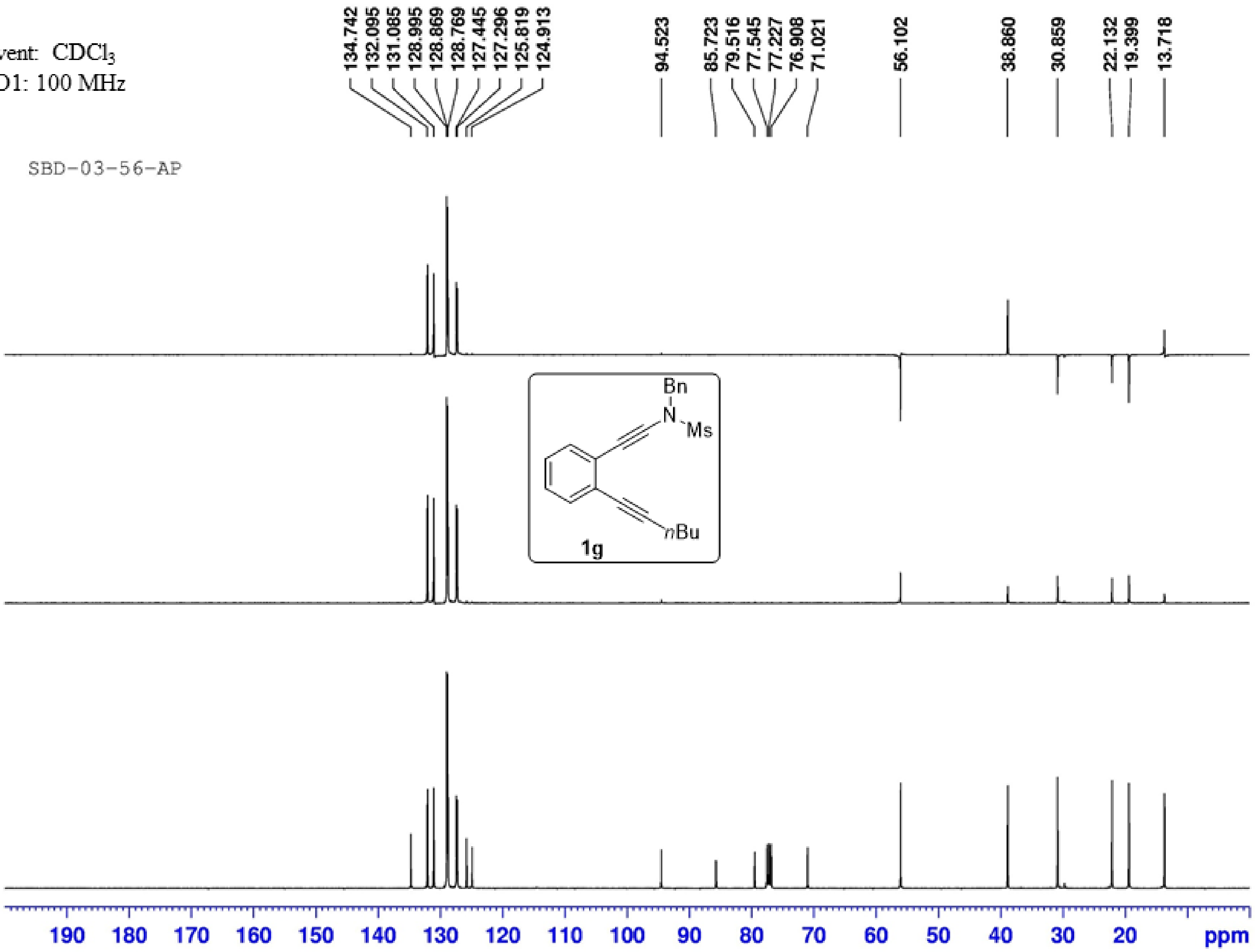

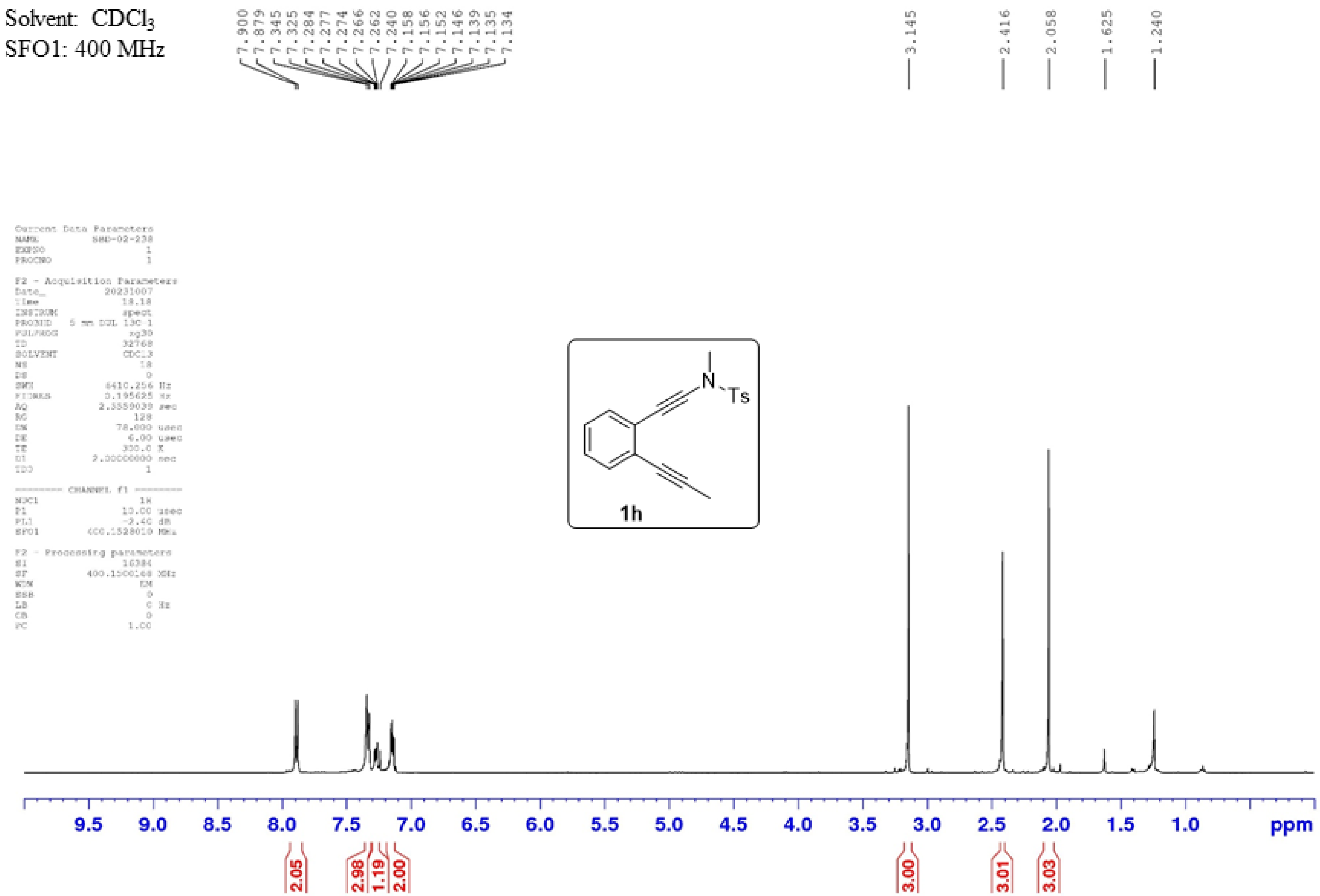
Solvent: CDCl3
SFO1: 400 MHz
7.900 7.879 7.345 7.325 7.284 7.277 7.274 7.266 7.262 7.240 7.158 7.156 7.152 7.146 7.139 7.135 7.134
3.145
2.416
2.058
1.625
1.240
N
Ts
1h
9.5 9.0 8.5 8.0 7.5 7.0 6.5 6.0 5.5 5.0 4.5 4.0 3.5 3.0 2.5 2.0 1.5 1.0 ppm
2.05
2.98
1.19
2.00
3.00
3.01
3.03

Solvent: $CDCl_3$
SFO1: 100 MHz

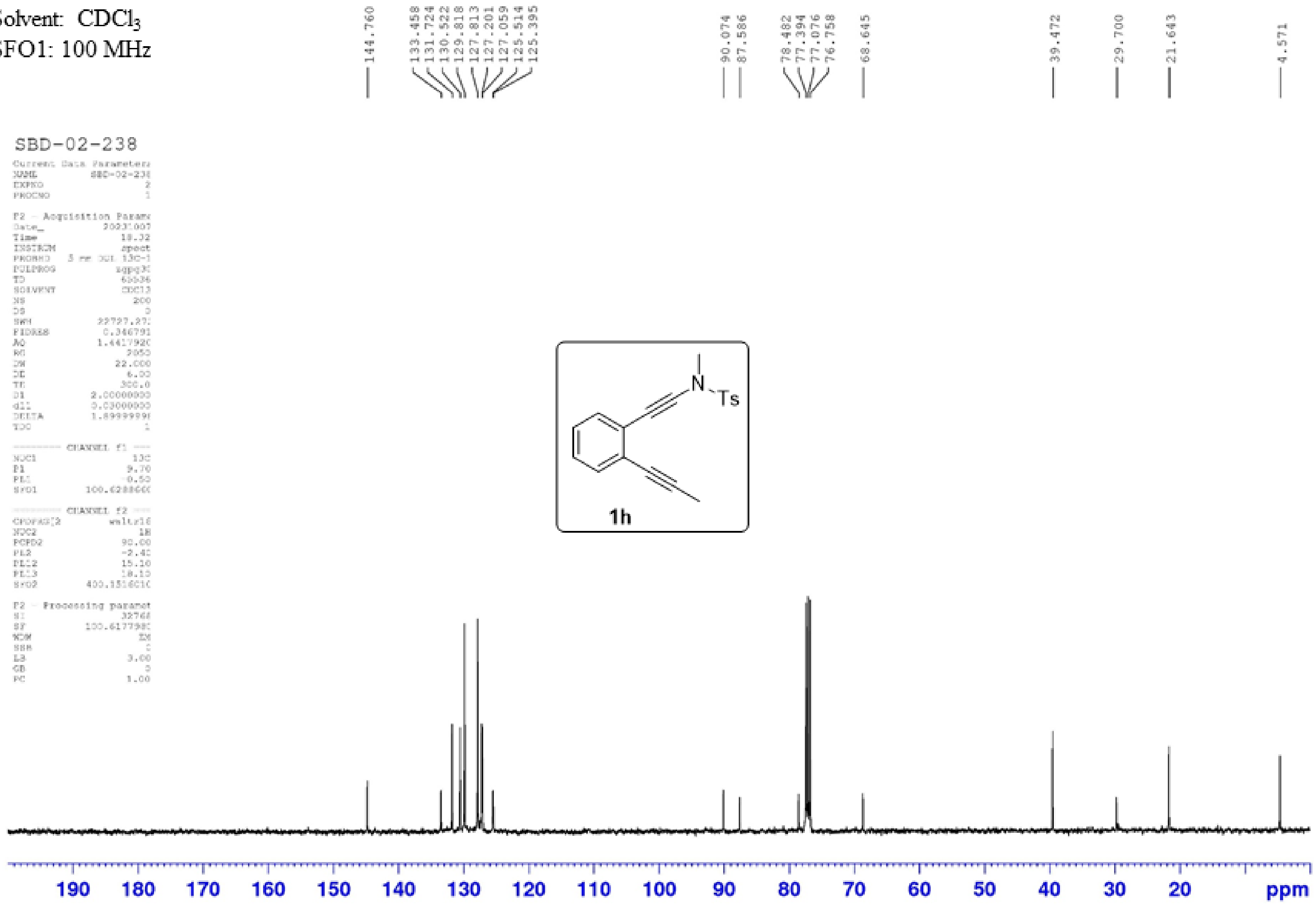

Solvent: $CDCl_3$
SFO1: 400 MHz

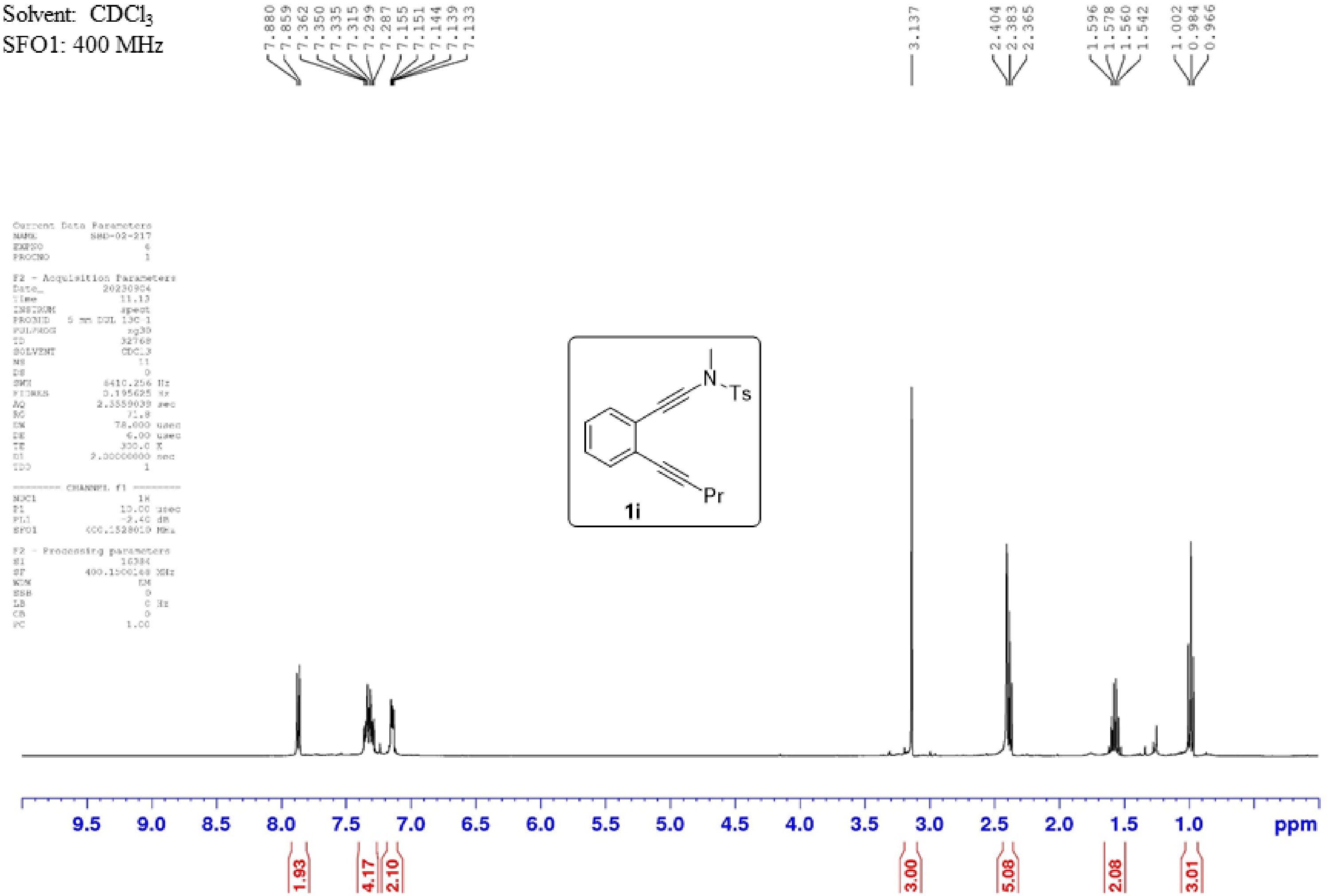

Solvent: $CDCl_3$
SFO1: 100 MHz

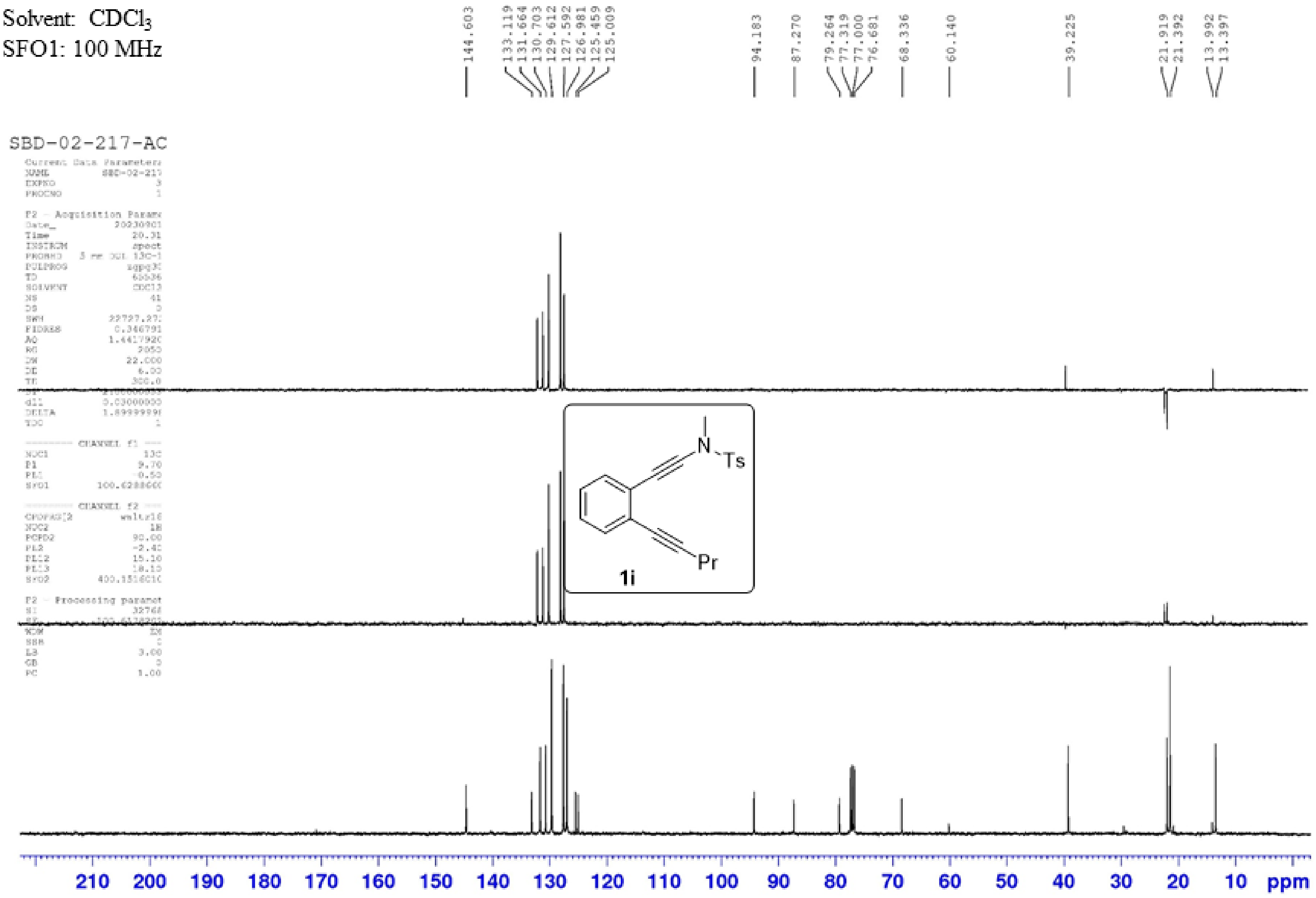

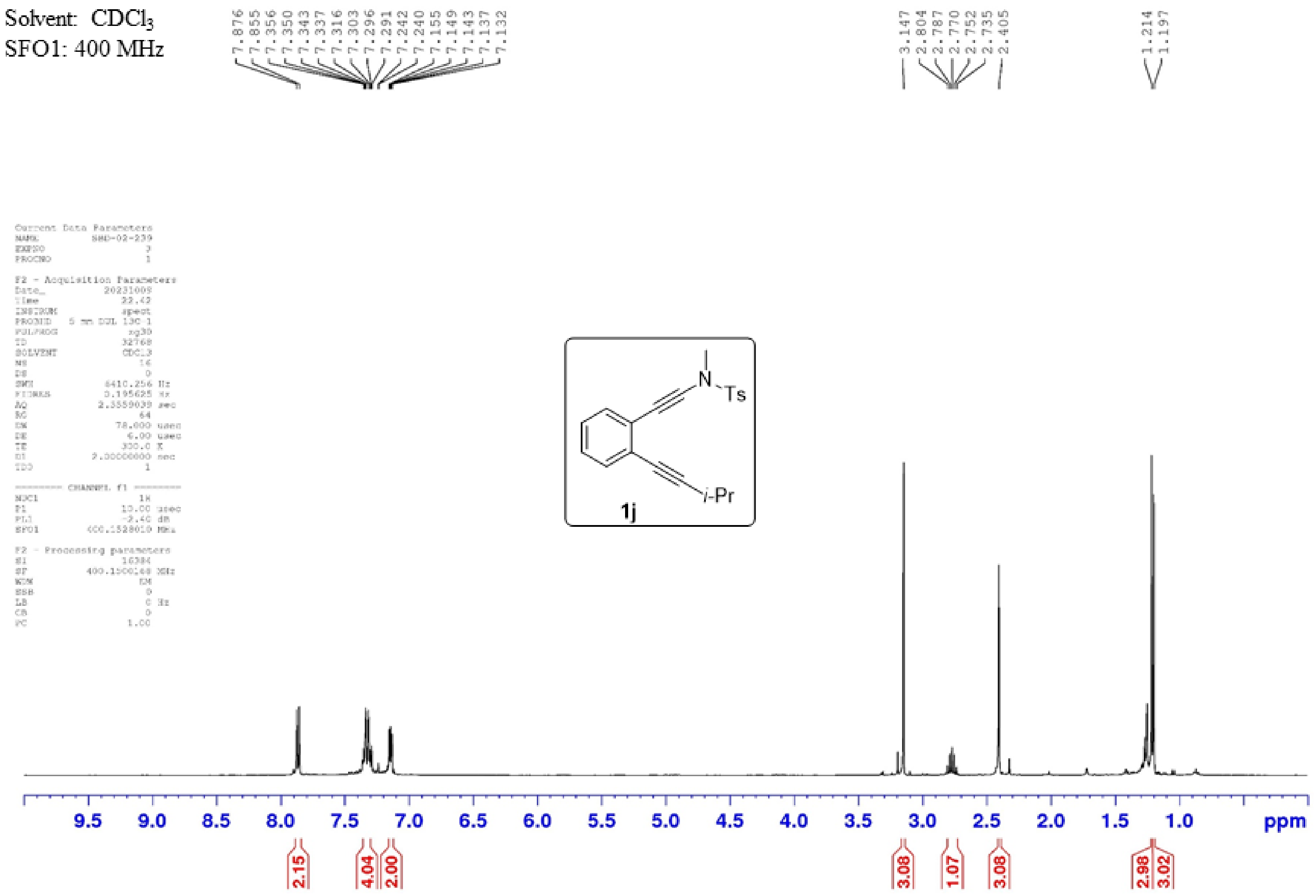
Solvent: CDCl3
SFO1: 400 MHz
7.876
7.855
7.356
7.350
7.343
7.337
7.316
7.303
7.296
7.291
7.242
7.240
7.155
7.149
7.143
7.137
7.132
3.147
2.804
2.787
2.770
2.752
2.735
2.405
1.214
1.197
N
Ts
i-Pr
1j
9.5
9.0
8.5
8.0
7.5
7.0
6.5
6.0
5.5
5.0
4.5
4.0
3.5
3.0
2.5
2.0
1.5
1.0
ppm
2.15
4.04
2.00
3.08
1.07
3.08
2.98
3.02

Solvent: $CDCl_3$
SFO1: 100 MHz

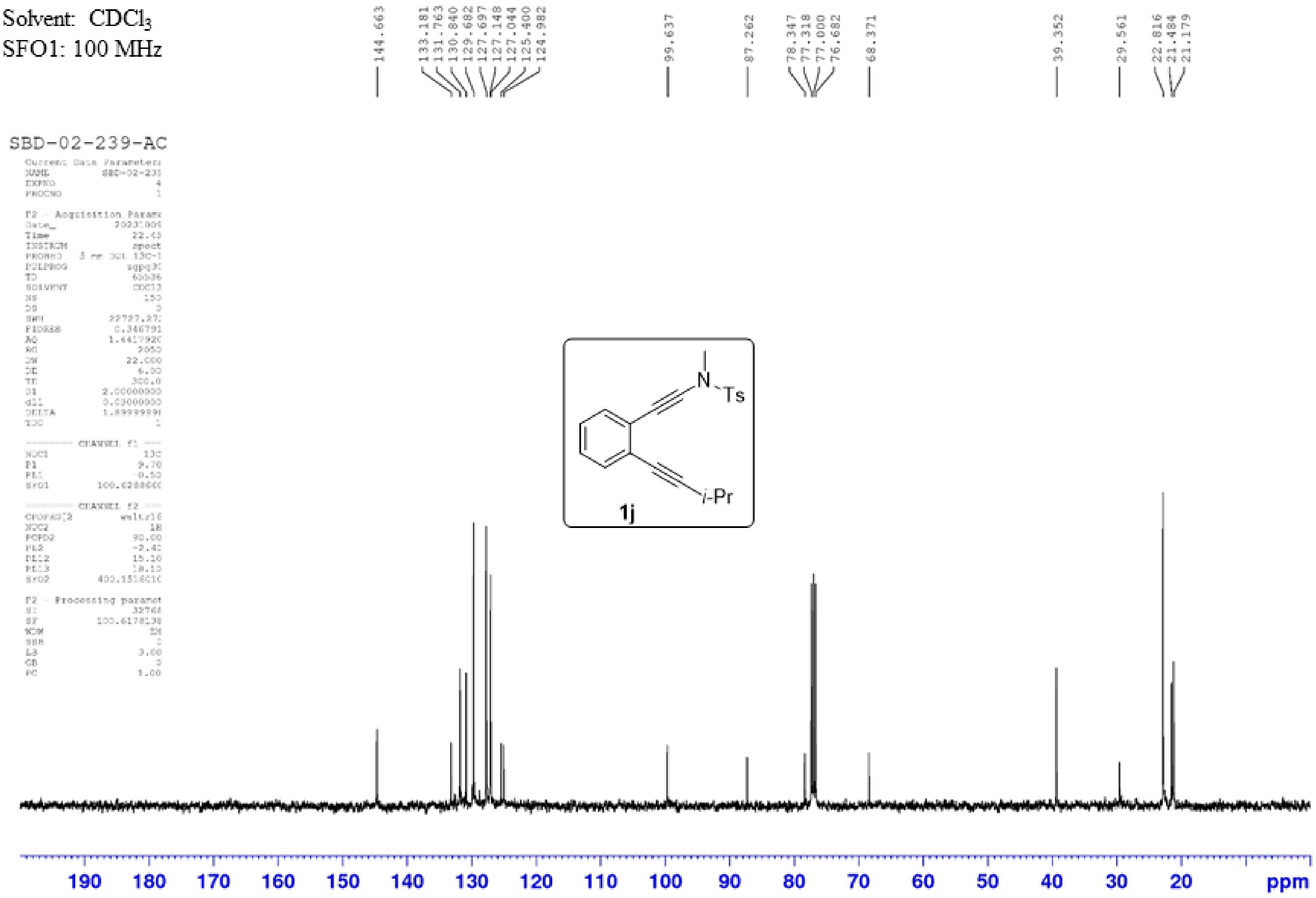

Solvent: $CDCl_3$
SFO1: 400 MHz

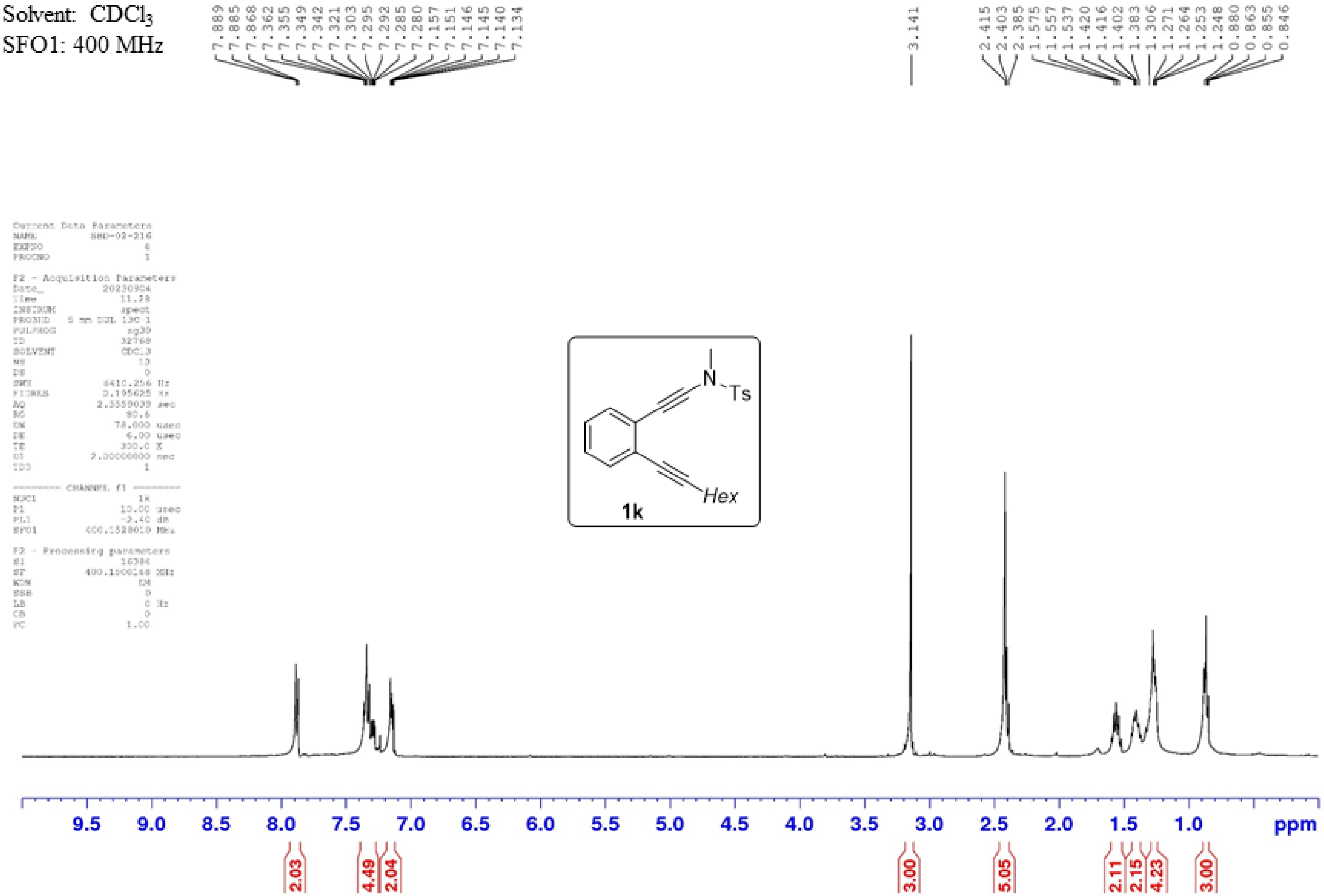

Solvent: $CDCl_3$
SFO1: 100 MHz

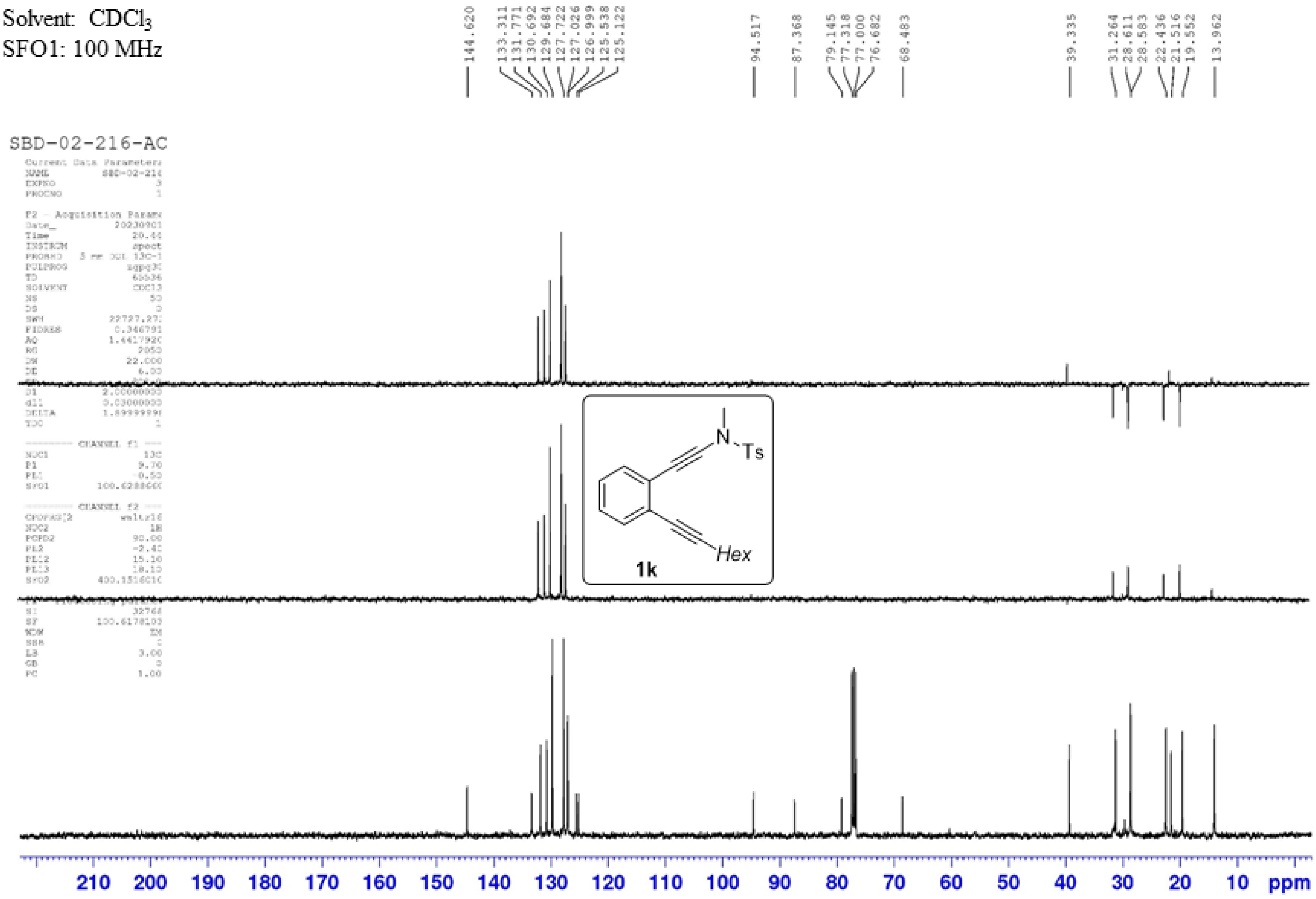

Solvent: $CDCl_3$
SFO1: 400 MHz

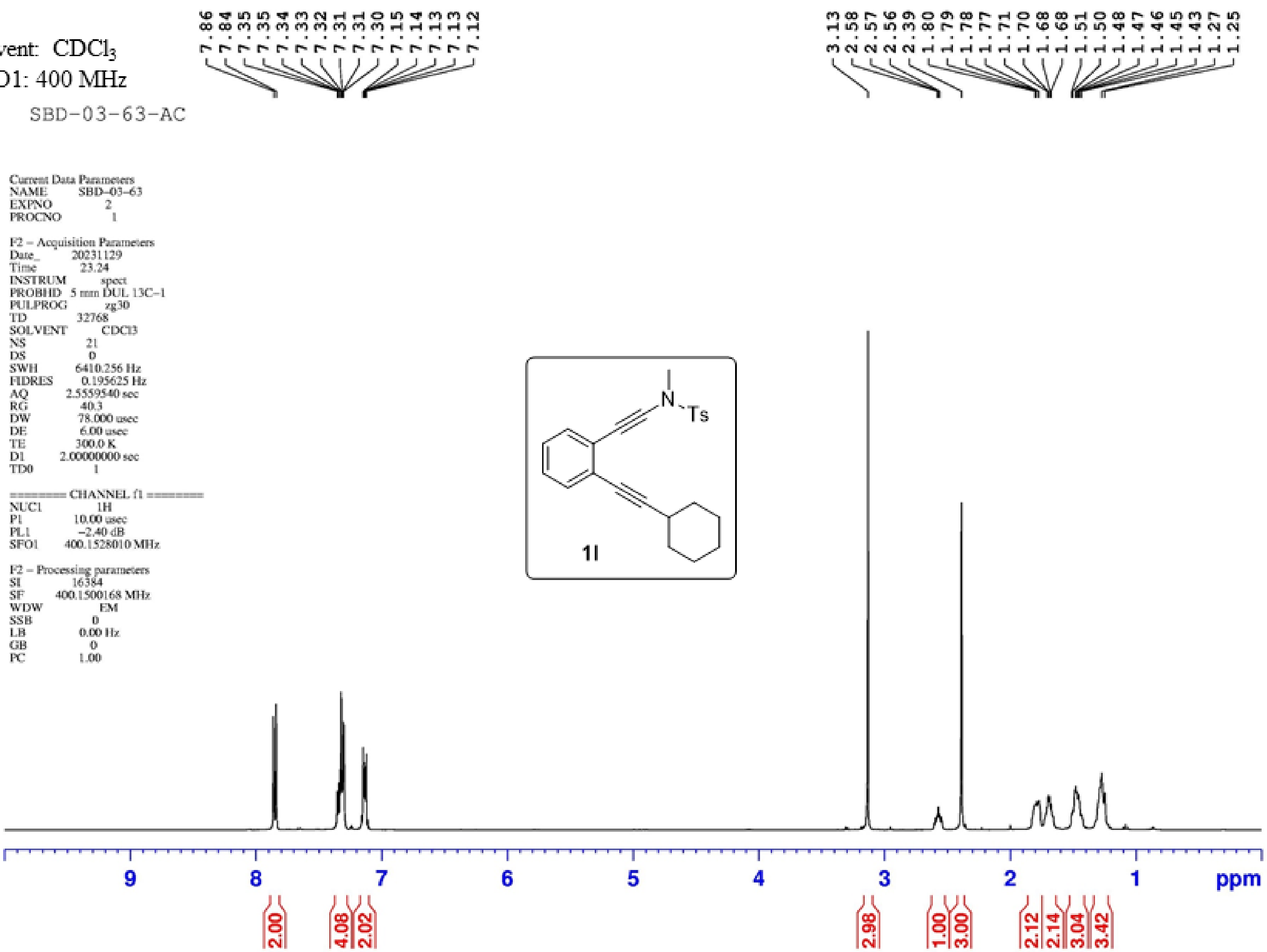

Solvent: $CDCl_3$
SFO1: 100 MHz

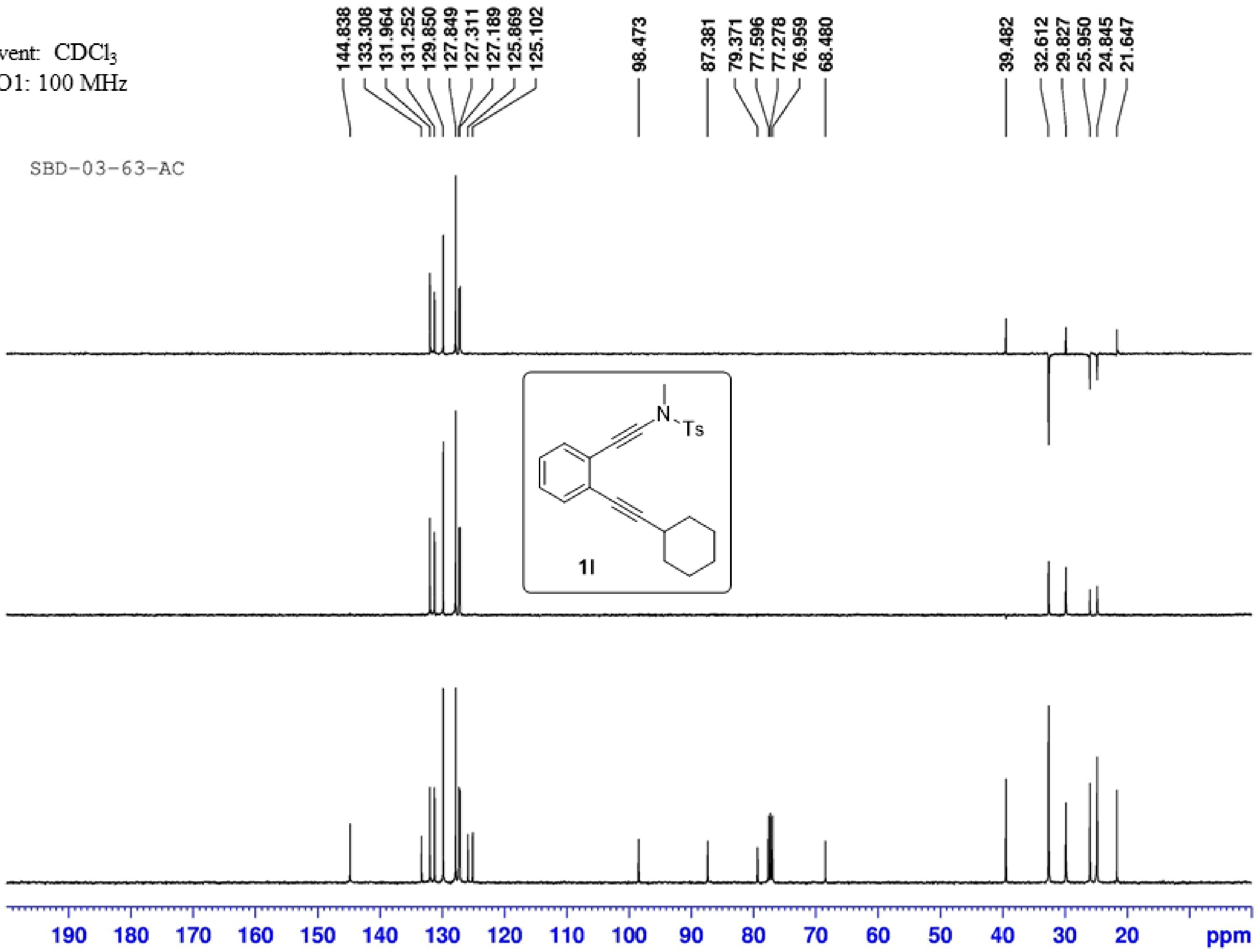

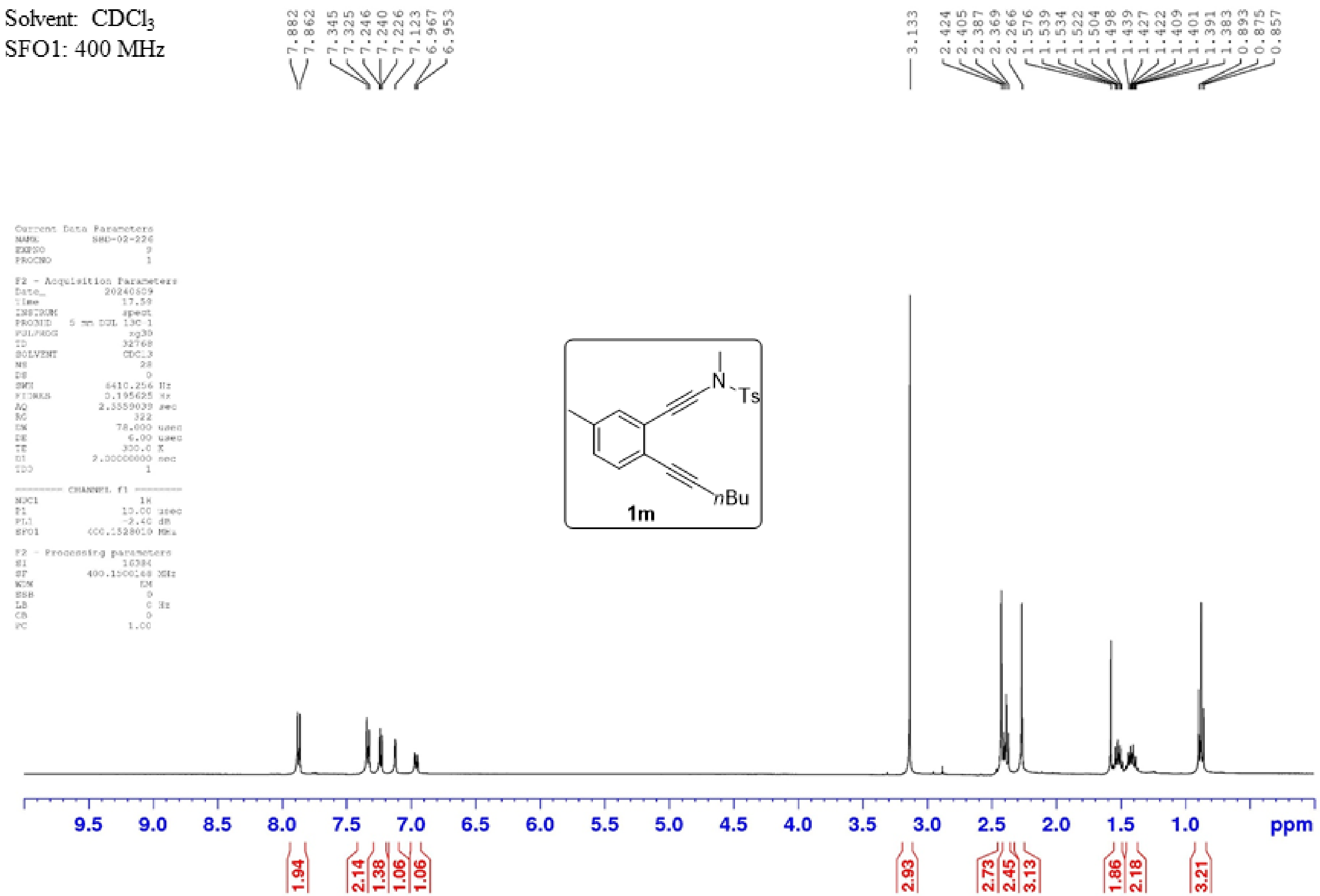
Solvent: $CDCl_3$
SFO1: 400 MHz
7.882
7.862
7.345
7.325
7.246
7.240
7.226
7.123
6.967
6.953
3.133
2.424
2.405
2.387
2.369
2.266
1.576
1.539
1.534
1.522
1.504
1.498
1.439
1.427
1.422
1.409
1.401
1.391
1.383
0.893
0.875
0.857
N
Ts
nBu
1m
9.5
9.0
8.5
8.0
7.5
7.0
6.5
6.0
5.5
5.0
4.5
4.0
3.5
3.0
2.5
2.0
1.5
1.0
ppm
1.94
2.14
1.38
1.06
1.06
2.93
2.73
2.45
3.13
1.86
2.18
3.21

Solvent: $CDCl_3$
SFO1: 100 MHz

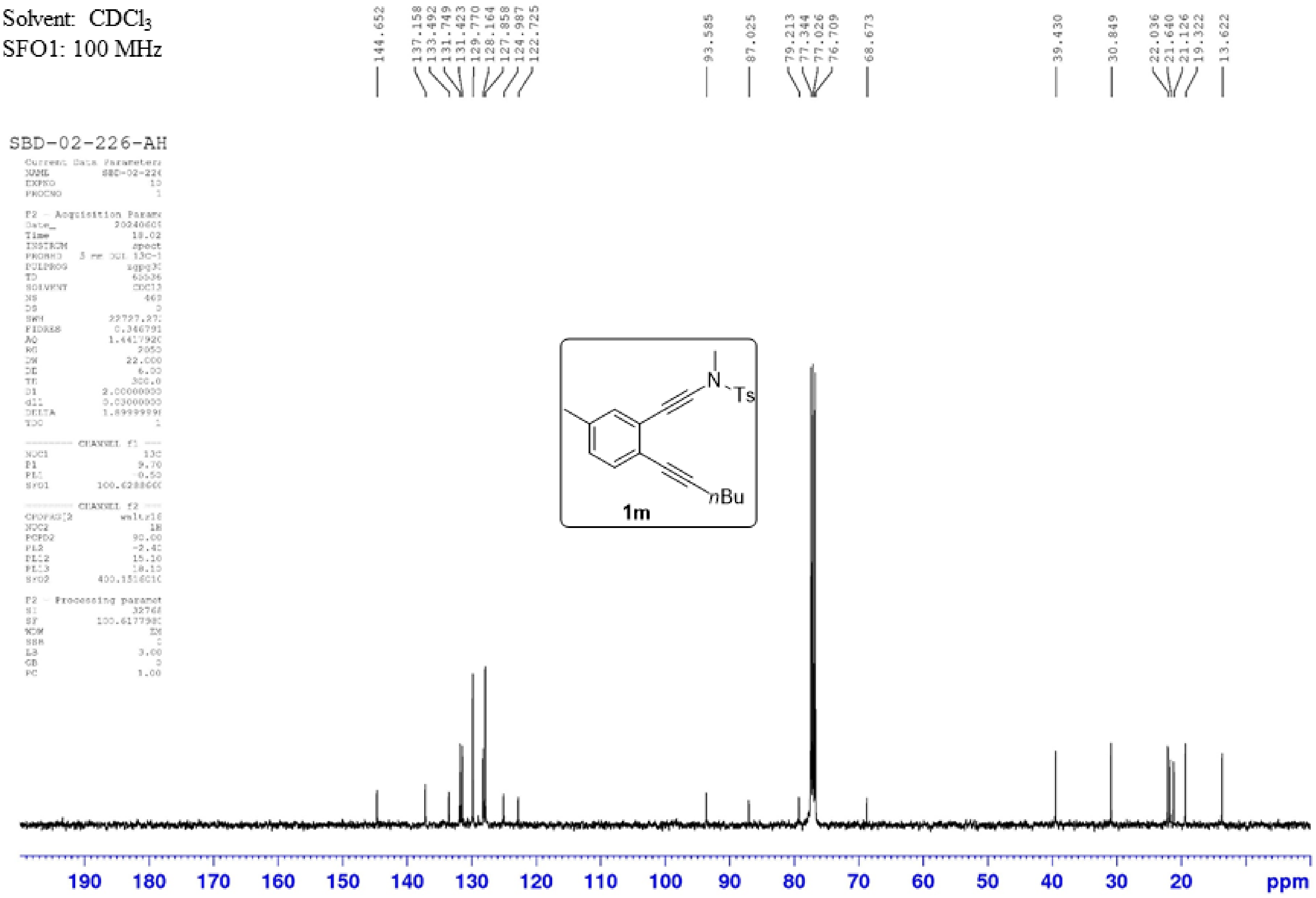

Solvent: $CDCl_3$
SFO1: 400 MHz

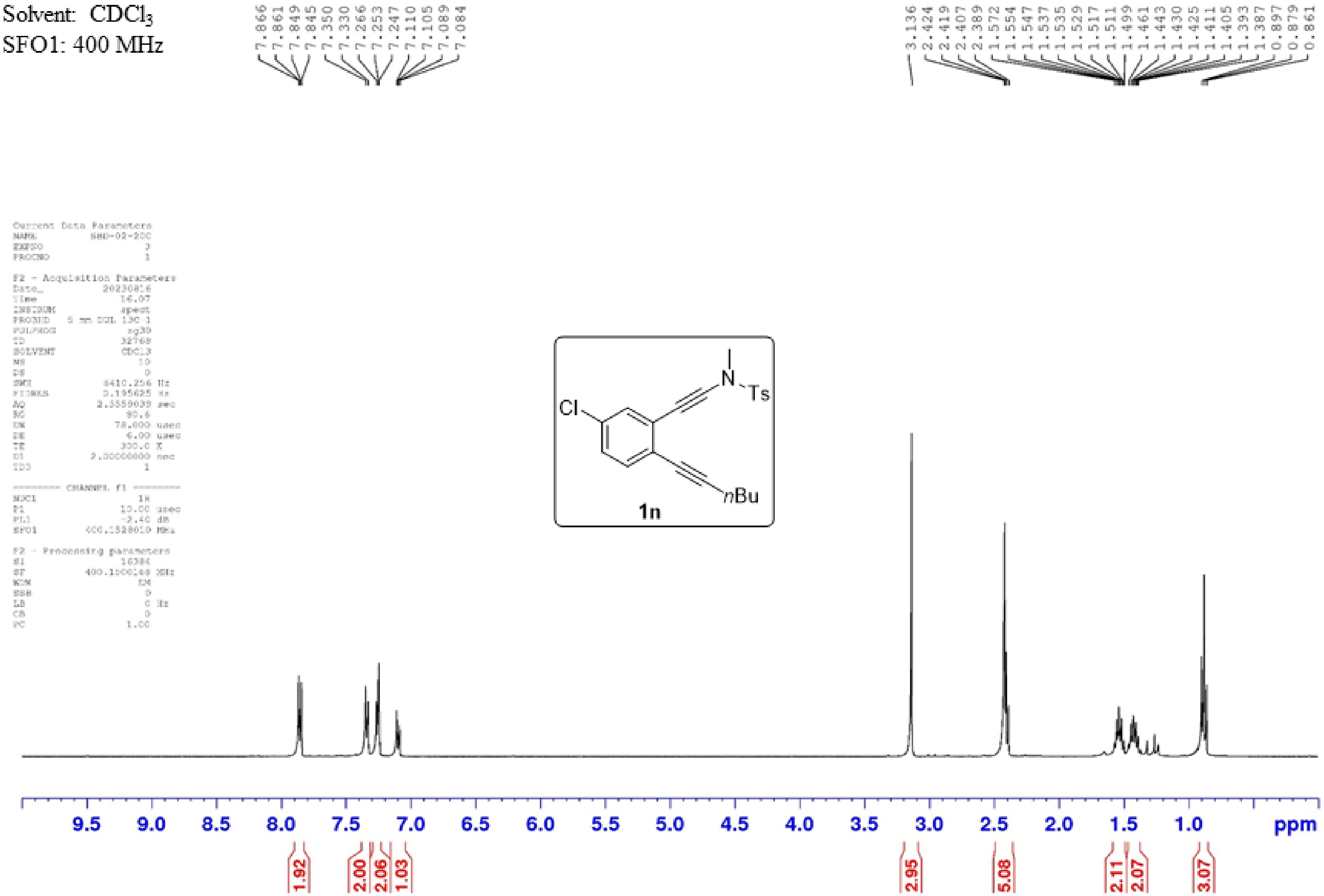

Solvent: $CDCl_3$
SFO1: 100 MHz

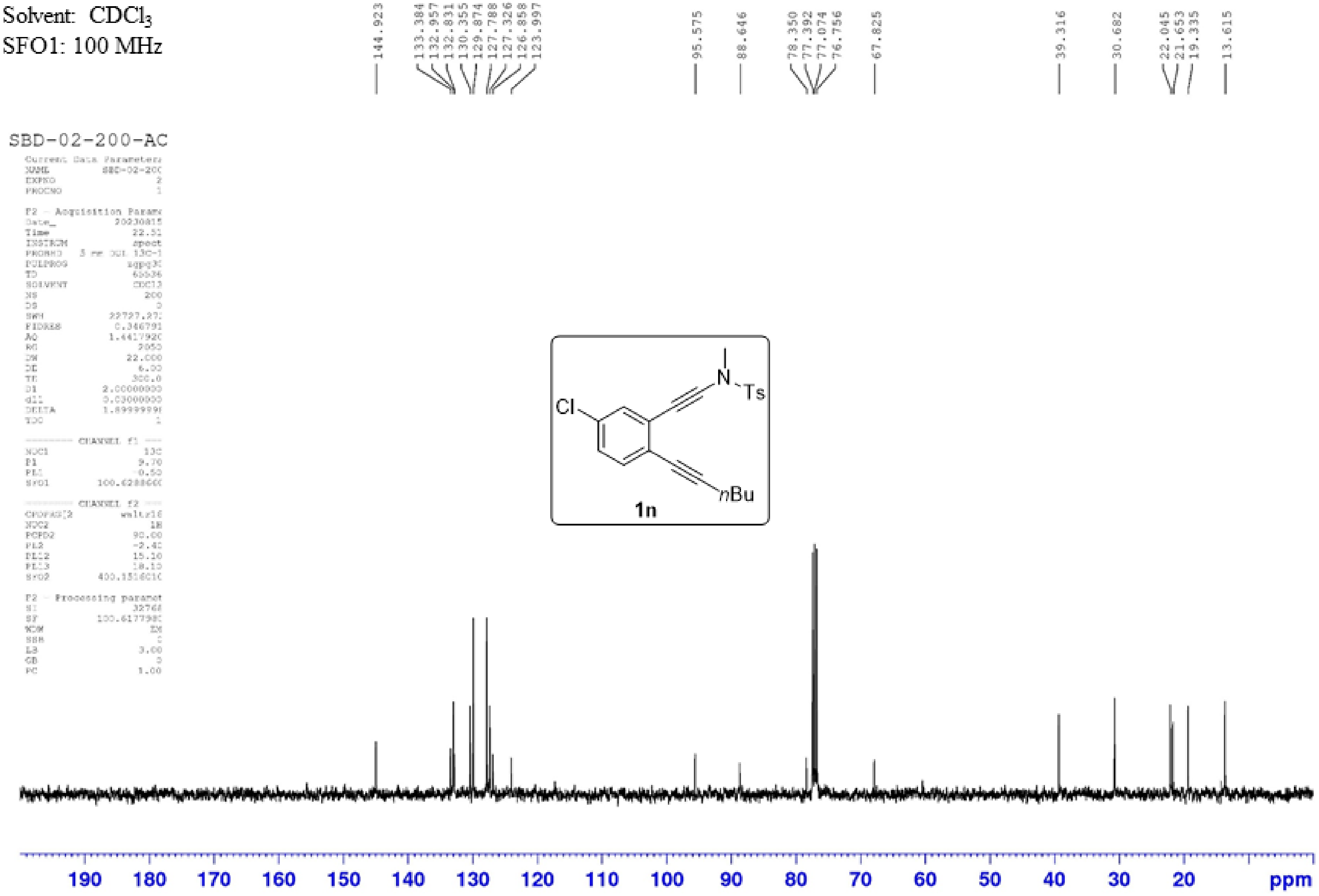

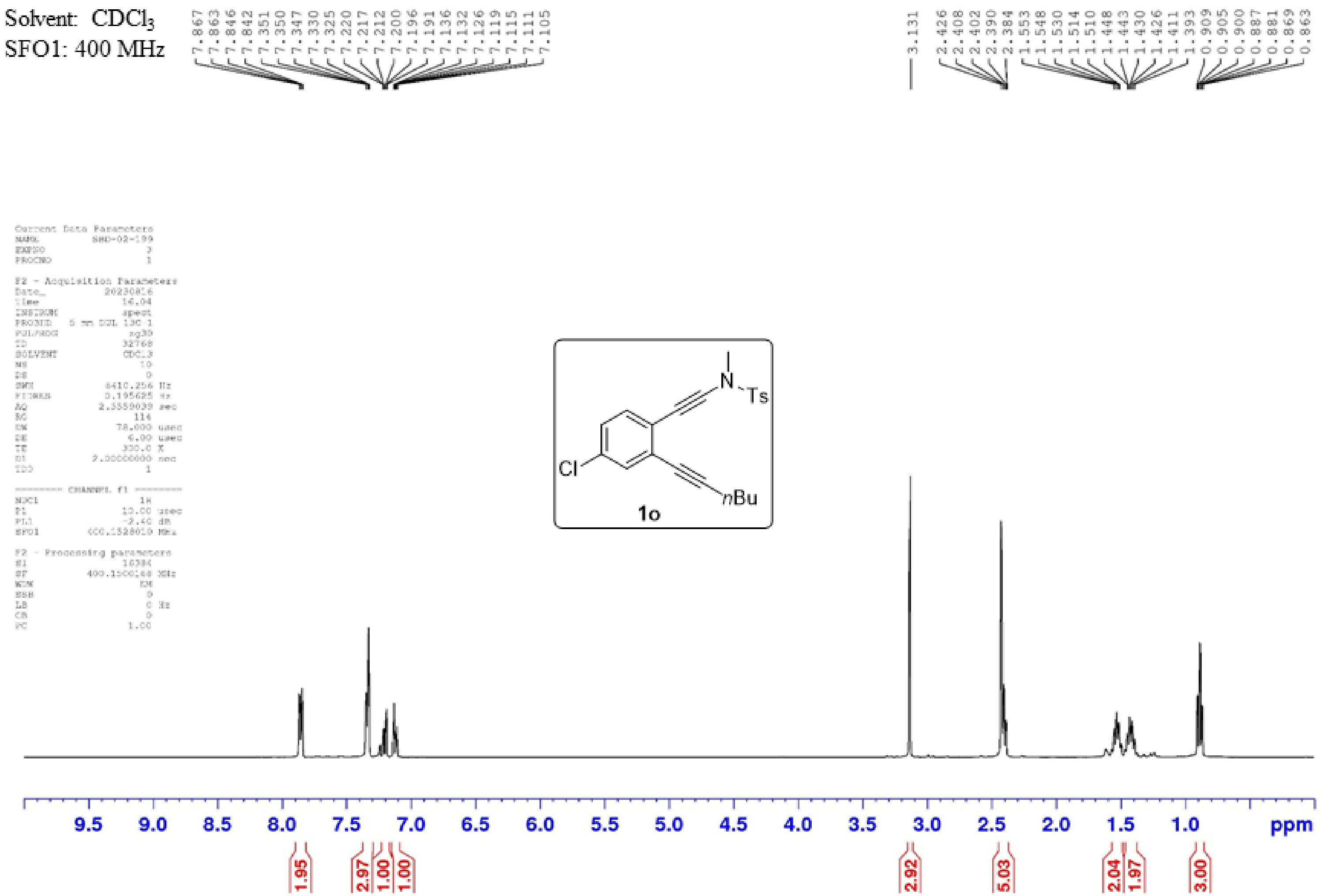
Solvent: CDCl3
SFO1: 400 MHz
7.867 7.863 7.846 7.842 7.351 7.350 7.347 7.330 7.325 7.220 7.217 7.212 7.200 7.196 7.191 7.136 7.132 7.126 7.119 7.115 7.111 7.105
3.131
2.426 2.408 2.402 2.390 2.384 1.553 1.548 1.530 1.514 1.510 1.448 1.443 1.430 1.426 1.411 1.393 0.909 0.905 0.900 0.887 0.881 0.869 0.863
N
Ts
Cl
nBu
1o
9.5 9.0 8.5 8.0 7.5 7.0 6.5 6.0 5.5 5.0 4.5 4.0 3.5 3.0 2.5 2.0 1.5 1.0 ppm
1.95 2.97 1.00 1.00 2.92 5.03 2.04 1.97 3.00

Solvent: $CDCl_3$
SFO1: 100 MHz

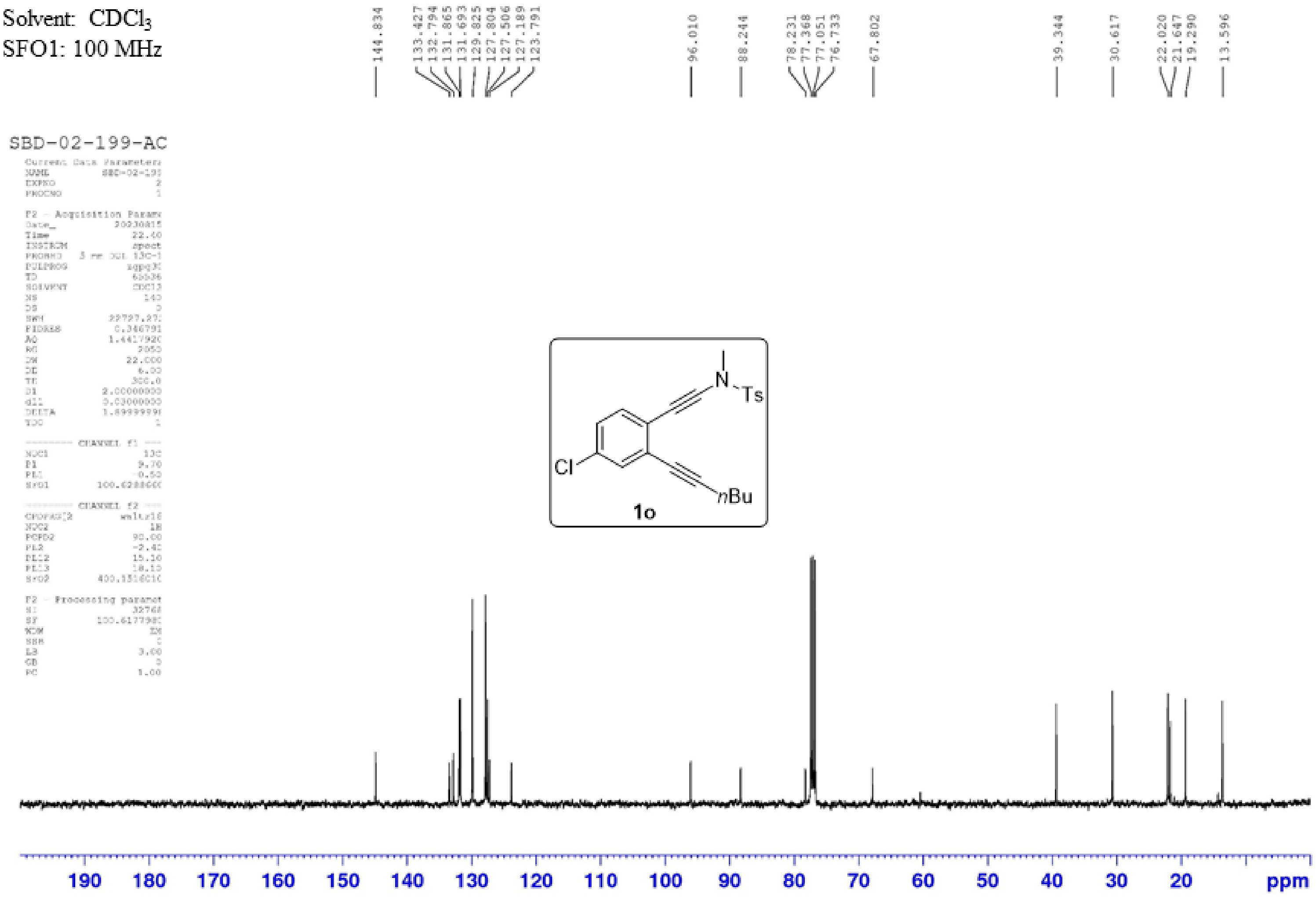

Solvent: $CDCl_3$
SFO1: 500 MHz

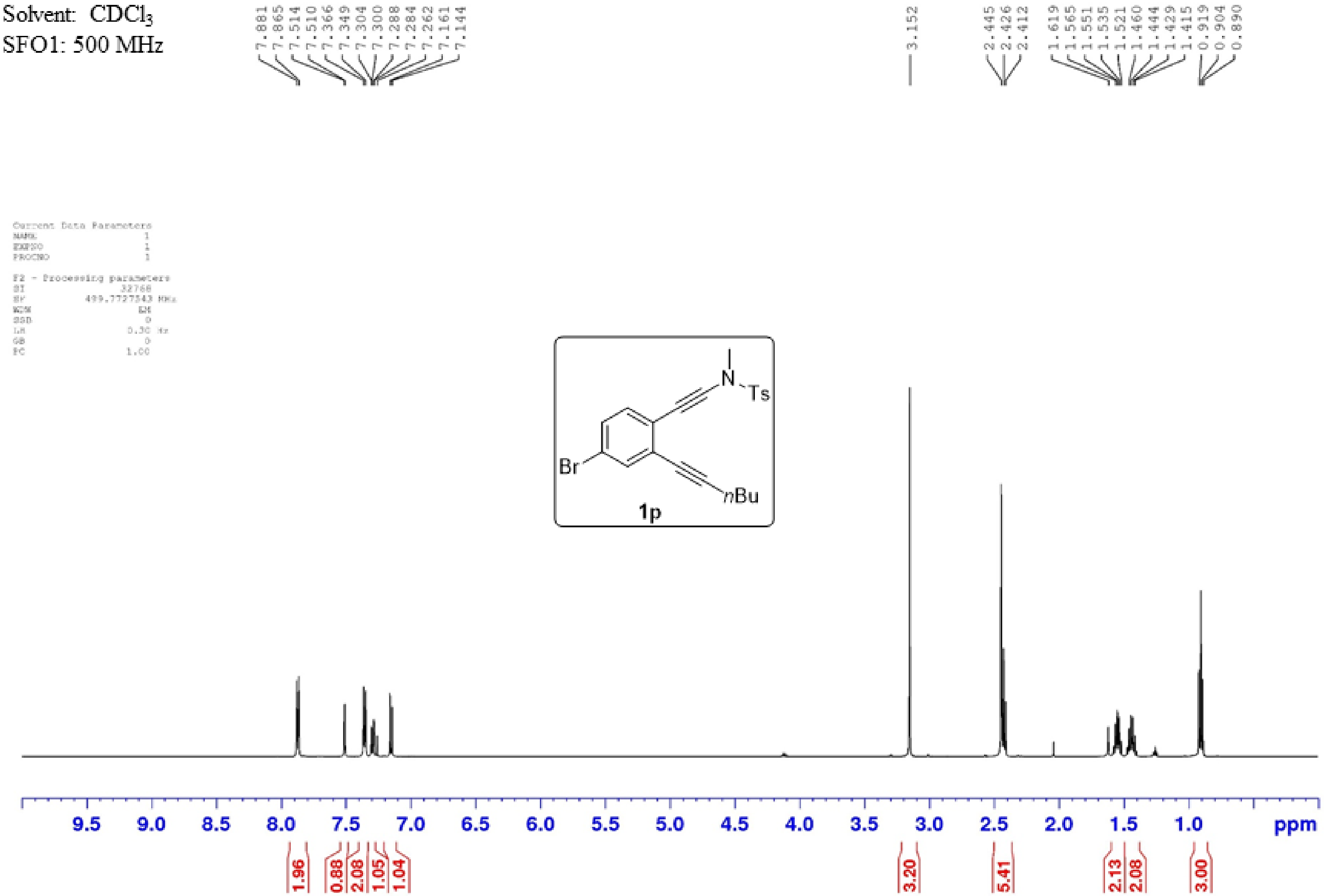

Solvent: $CDCl_3$
SFO1: 100 MHz

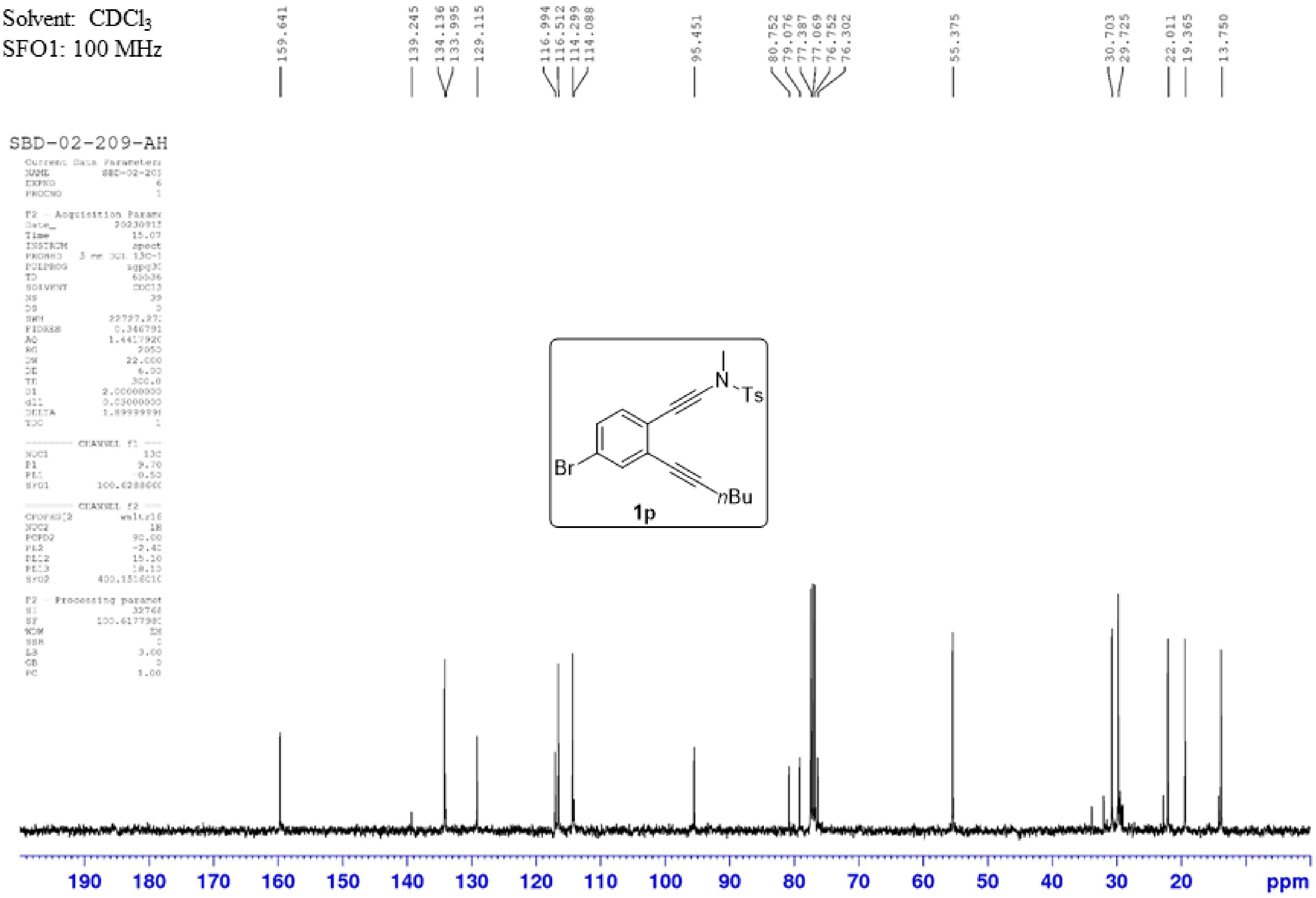

Solvent: $CDCl_3$
SFO1: 400 MHz

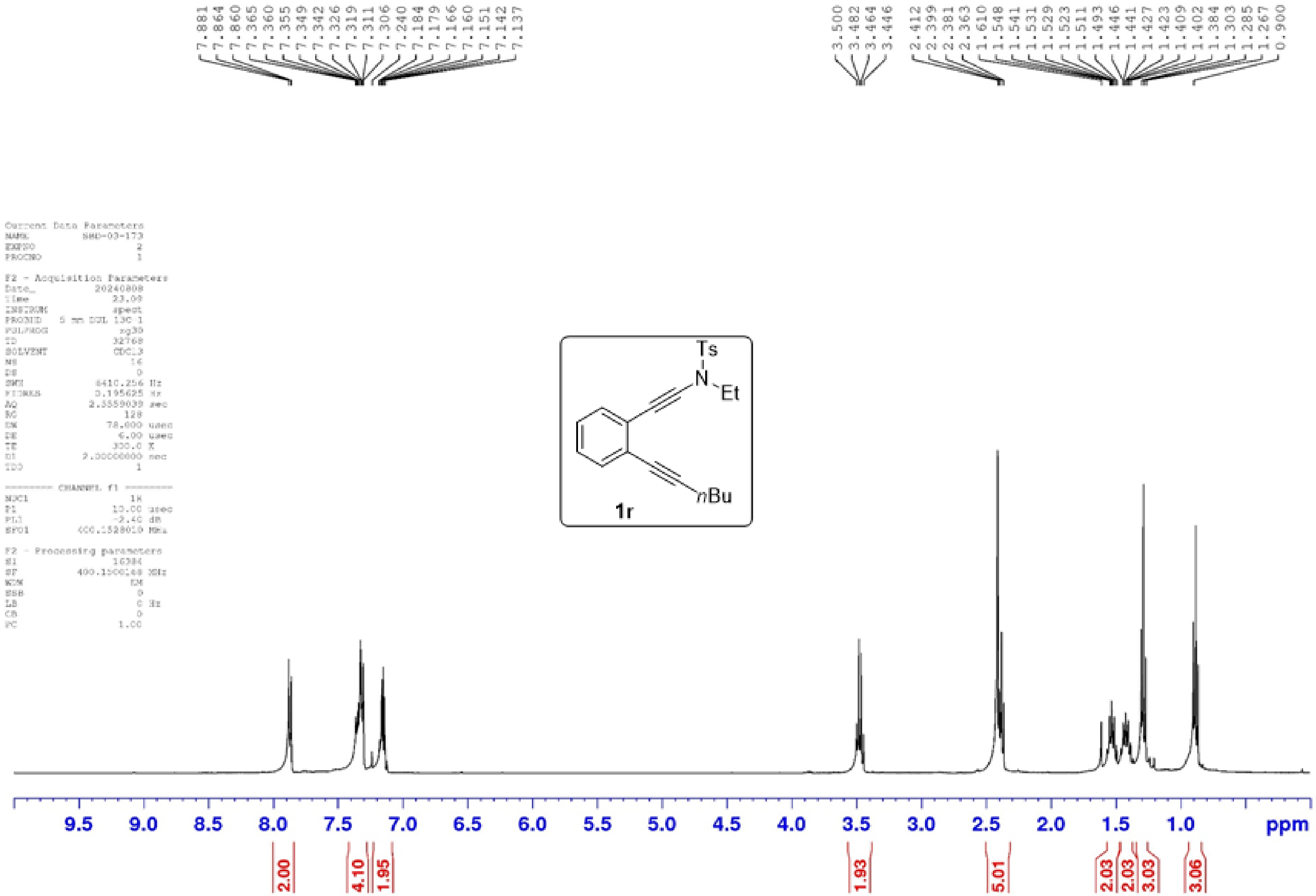

Solvent: $CDCl_3$
SFO1: 100 MHz

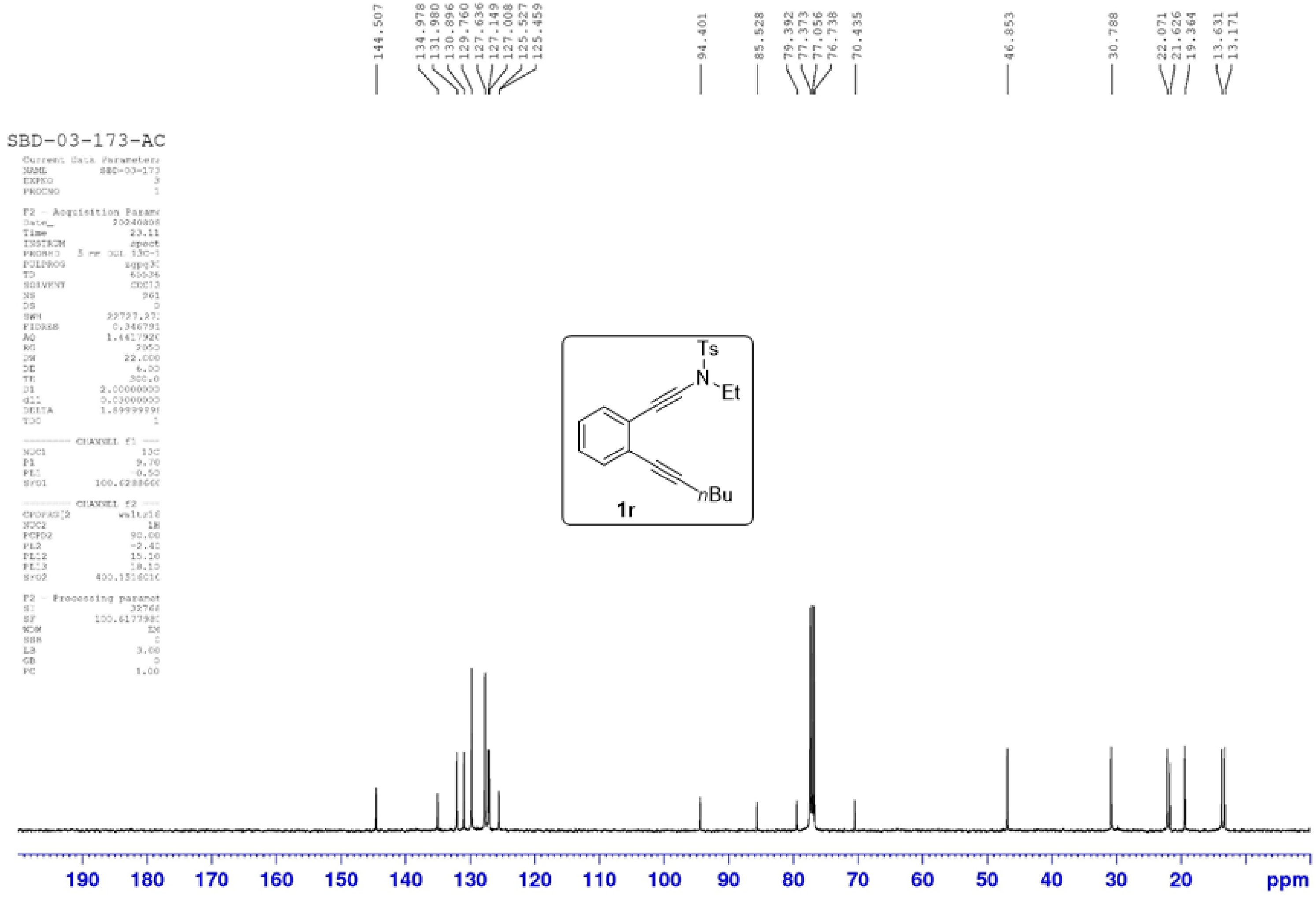

Solvent: $CDCl_3$
SFO1: 400 MHz

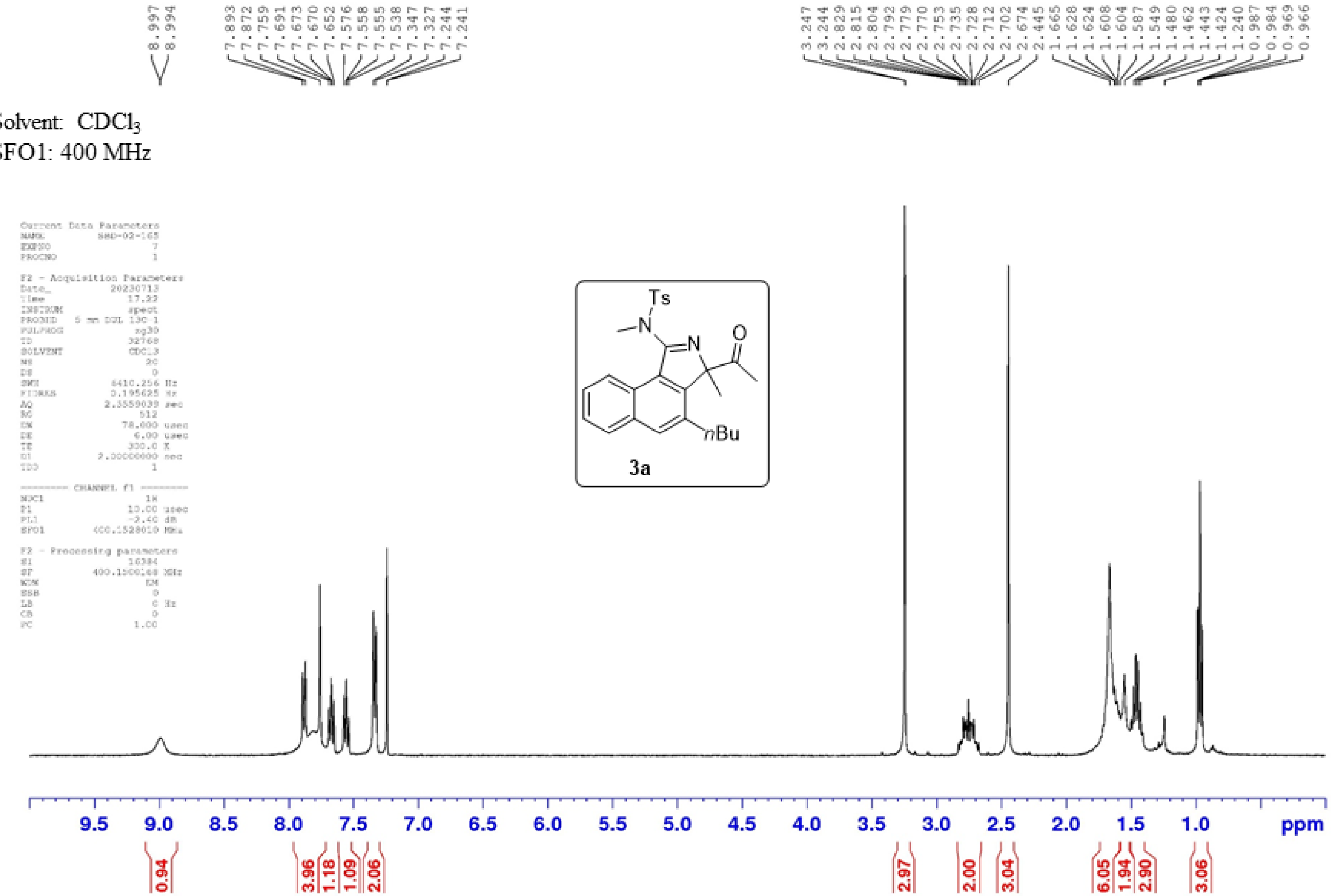

Solvent: $CDCl_3$
SFO1: 100 MHz

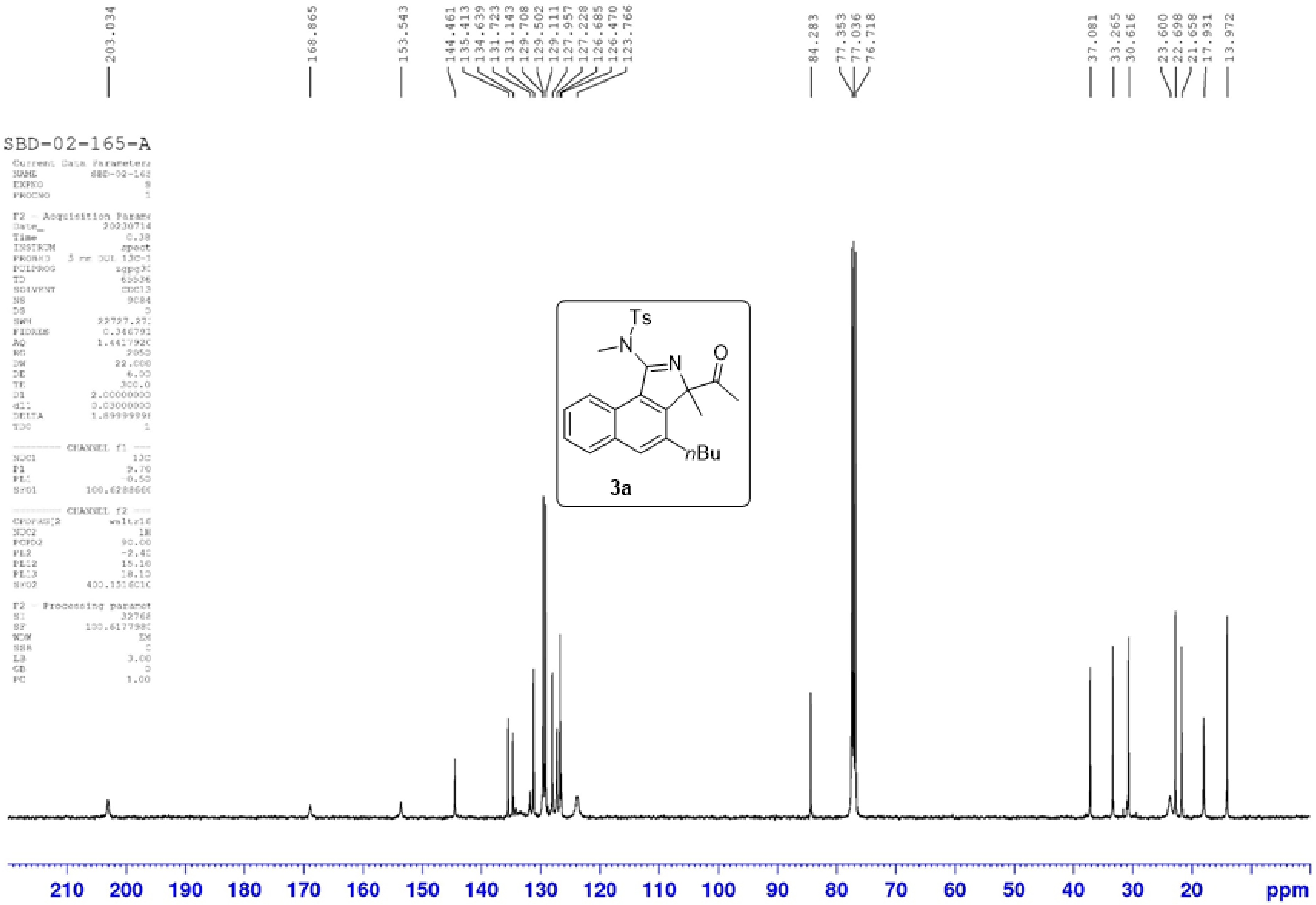

Solvent: $CDCl_3$
SFO1: 700 MHz

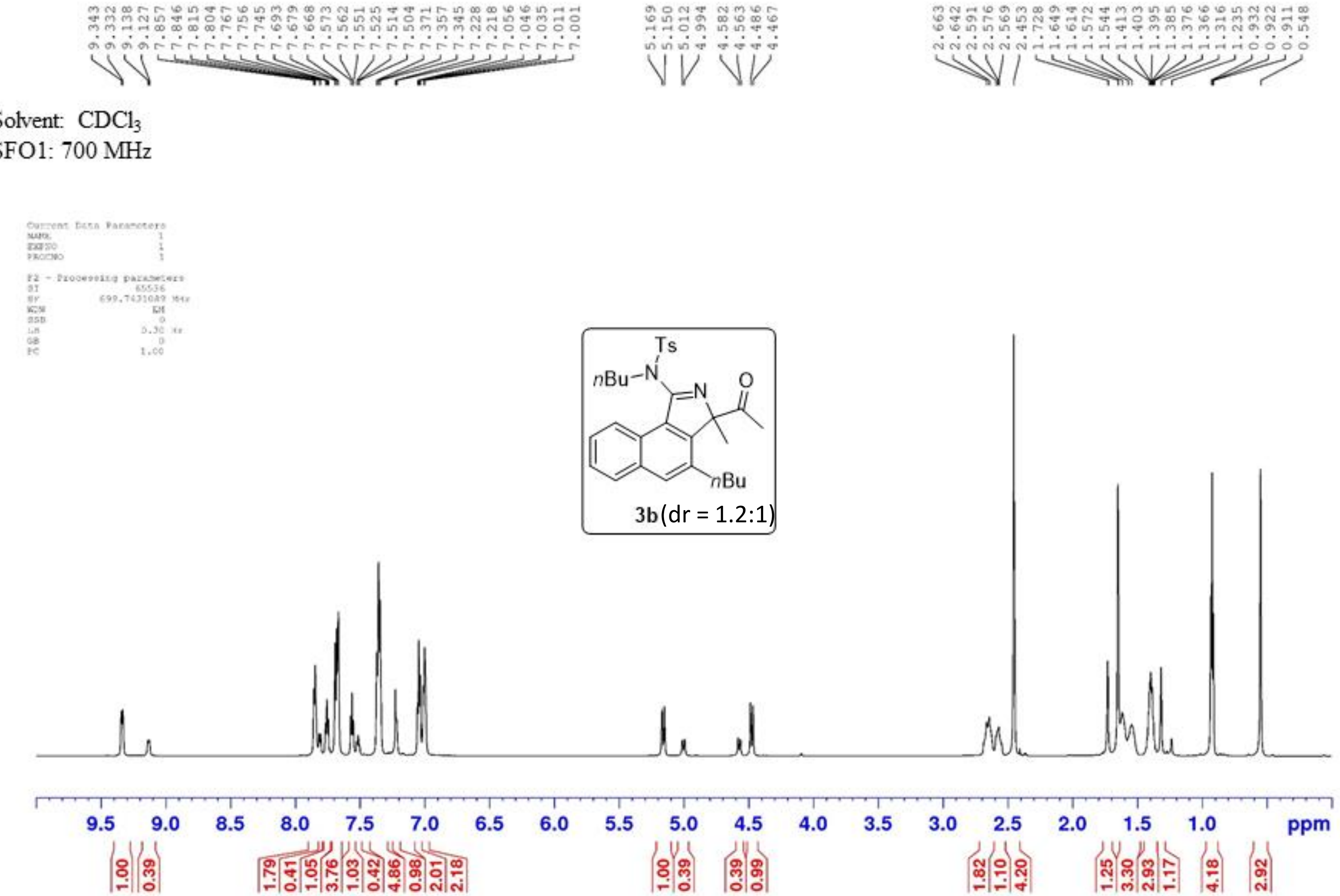

Solvent: $CDCl_3$
SFO1: 175 MHz

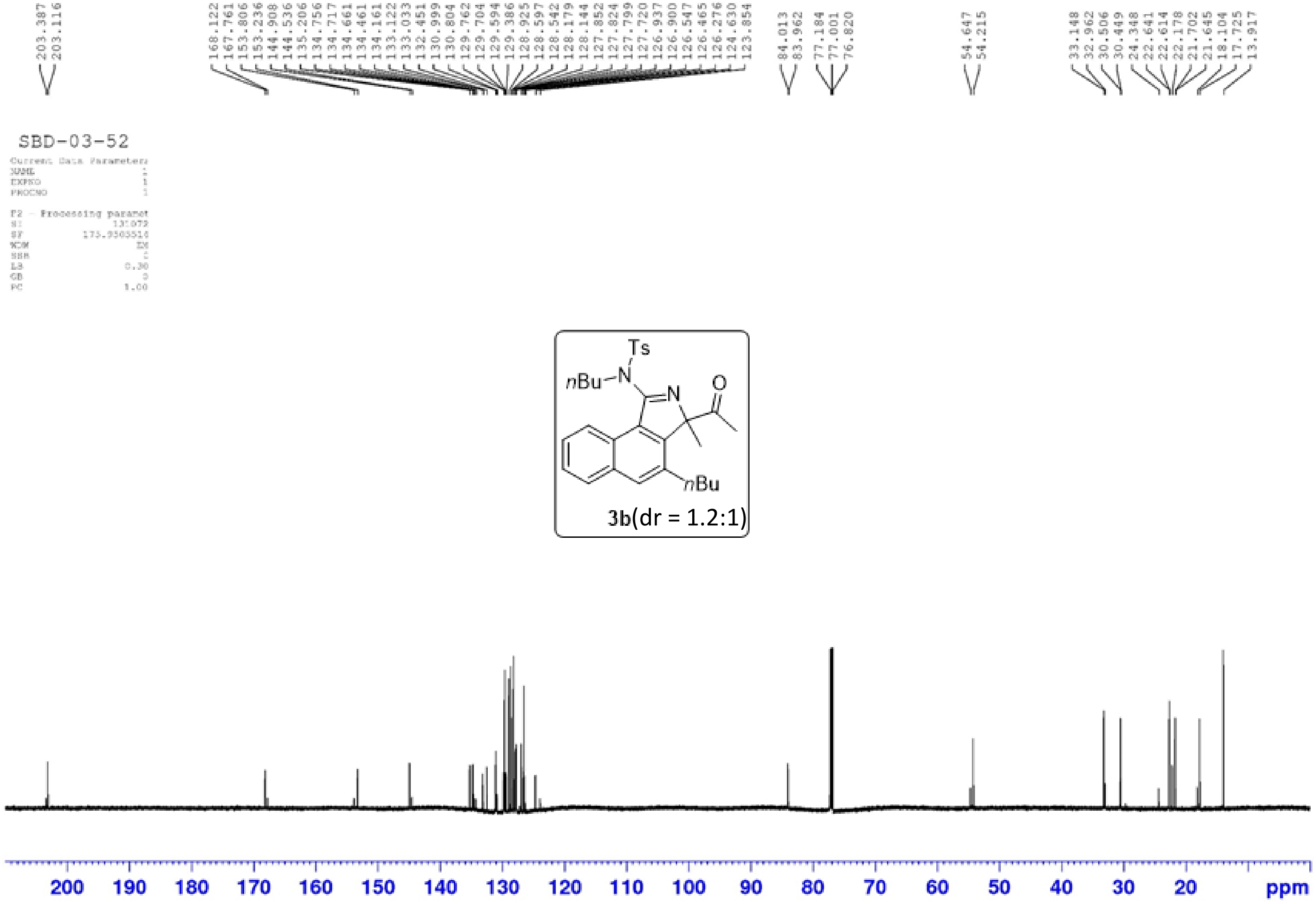

3b(dr = 1.2:1)

Solvent: $CDCl_3$
SFO1: 400 MHz

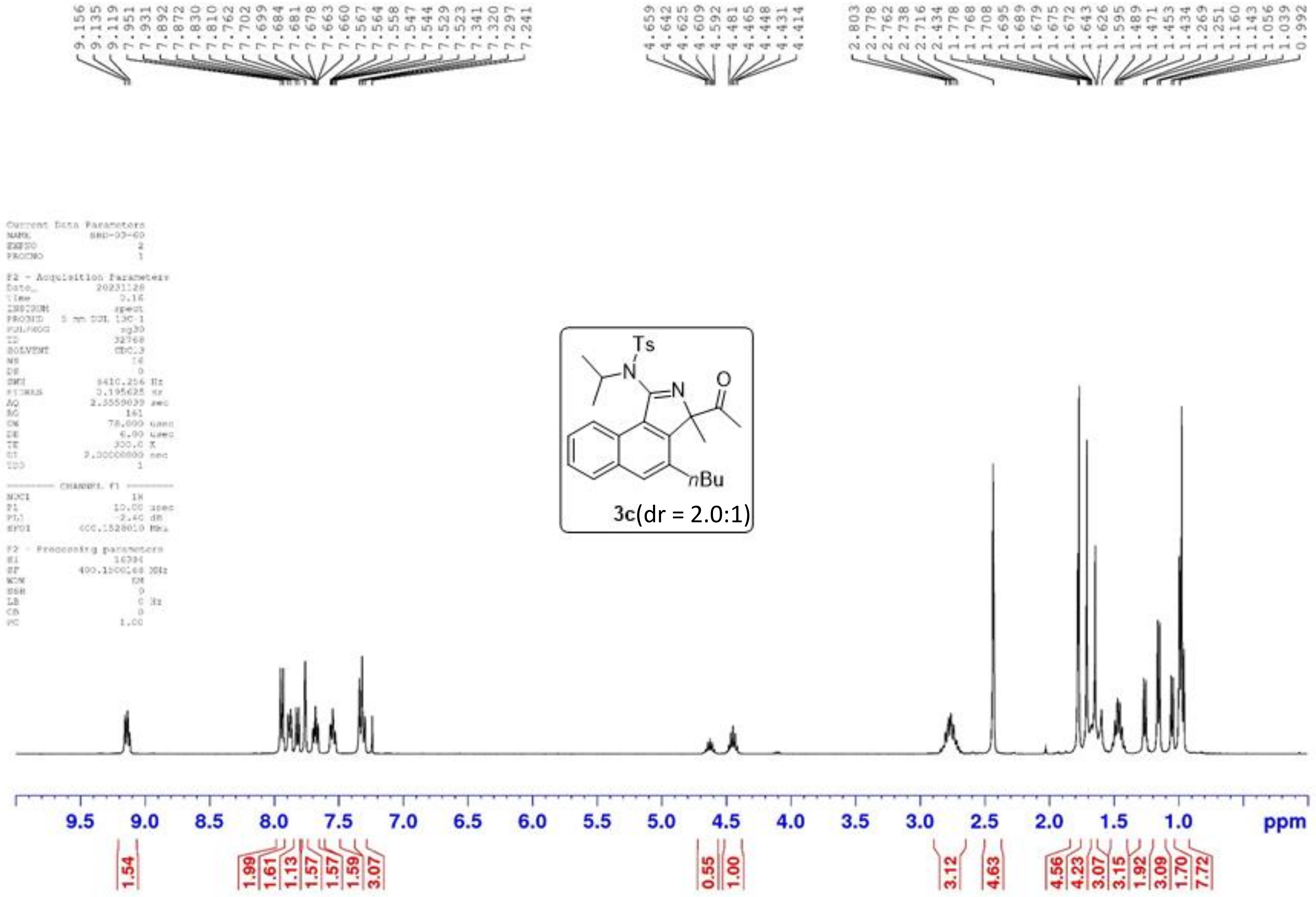

Solvent: $CDCl_3$
SFO1: 100 MHz

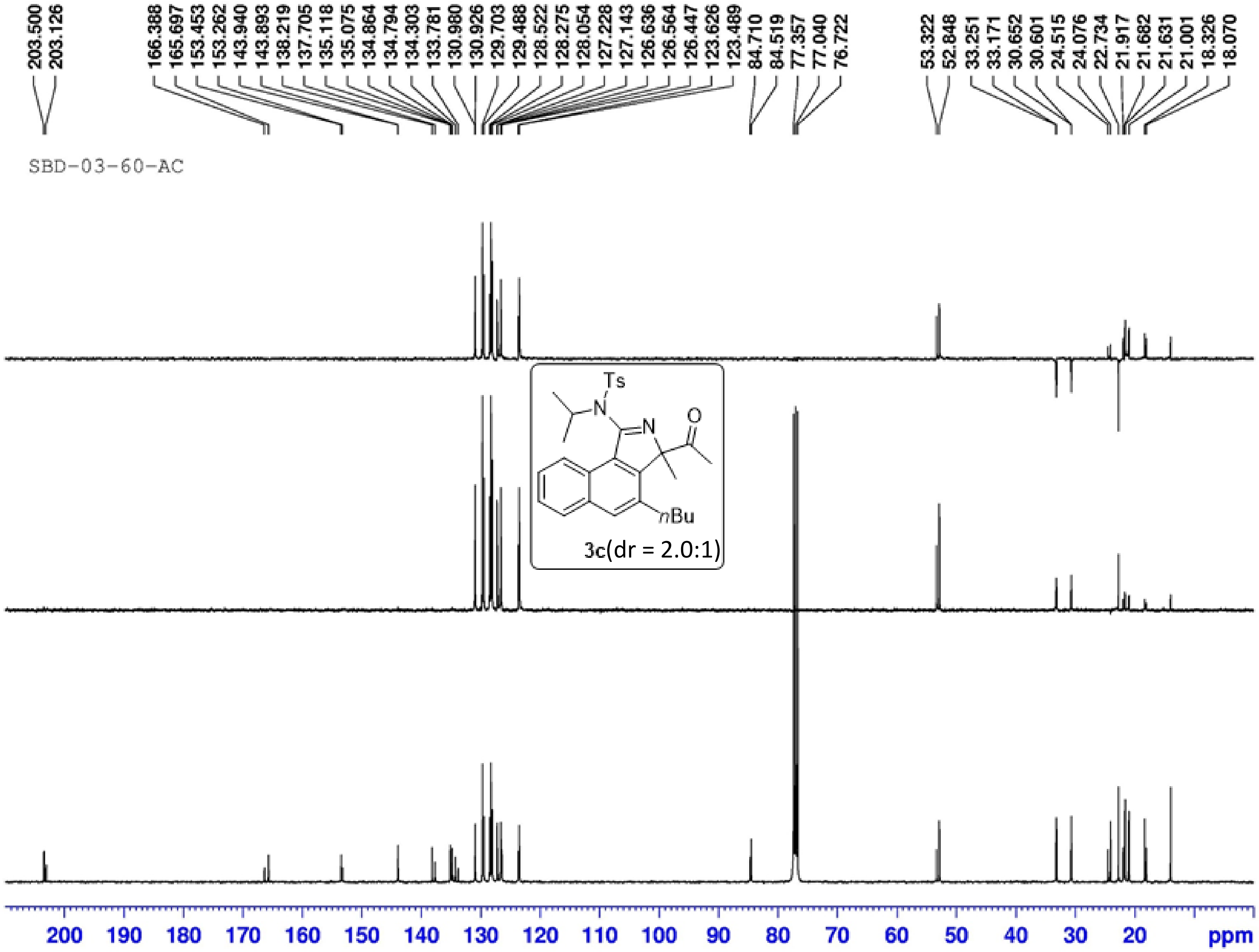

Solvent: $CDCl_3$
SFO1: 700 MHz

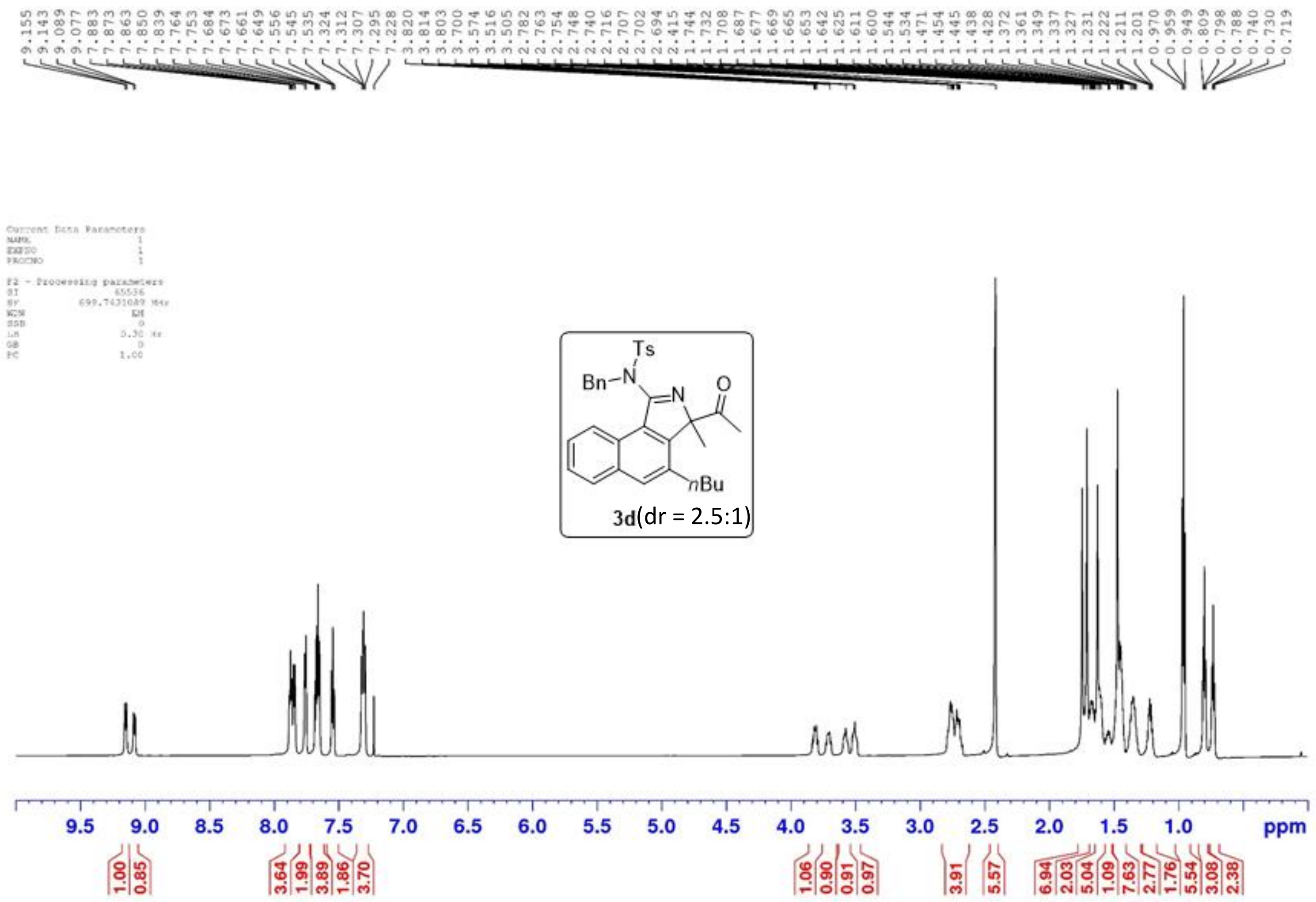

Solvent: $CDCl_3$
SFO1: 175 MHz

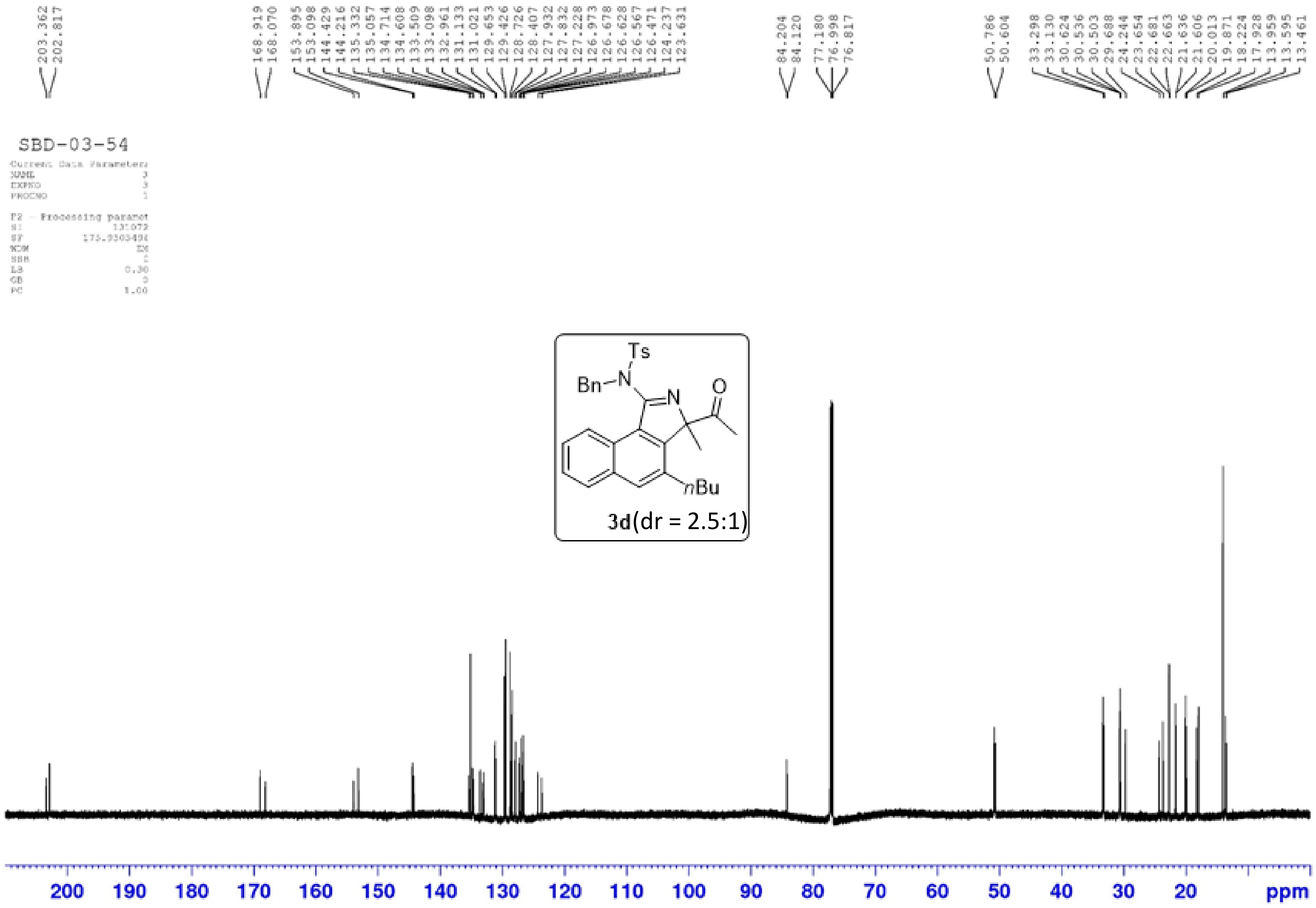

Solvent: $CDCl_3$
SFO1: 700 MHz

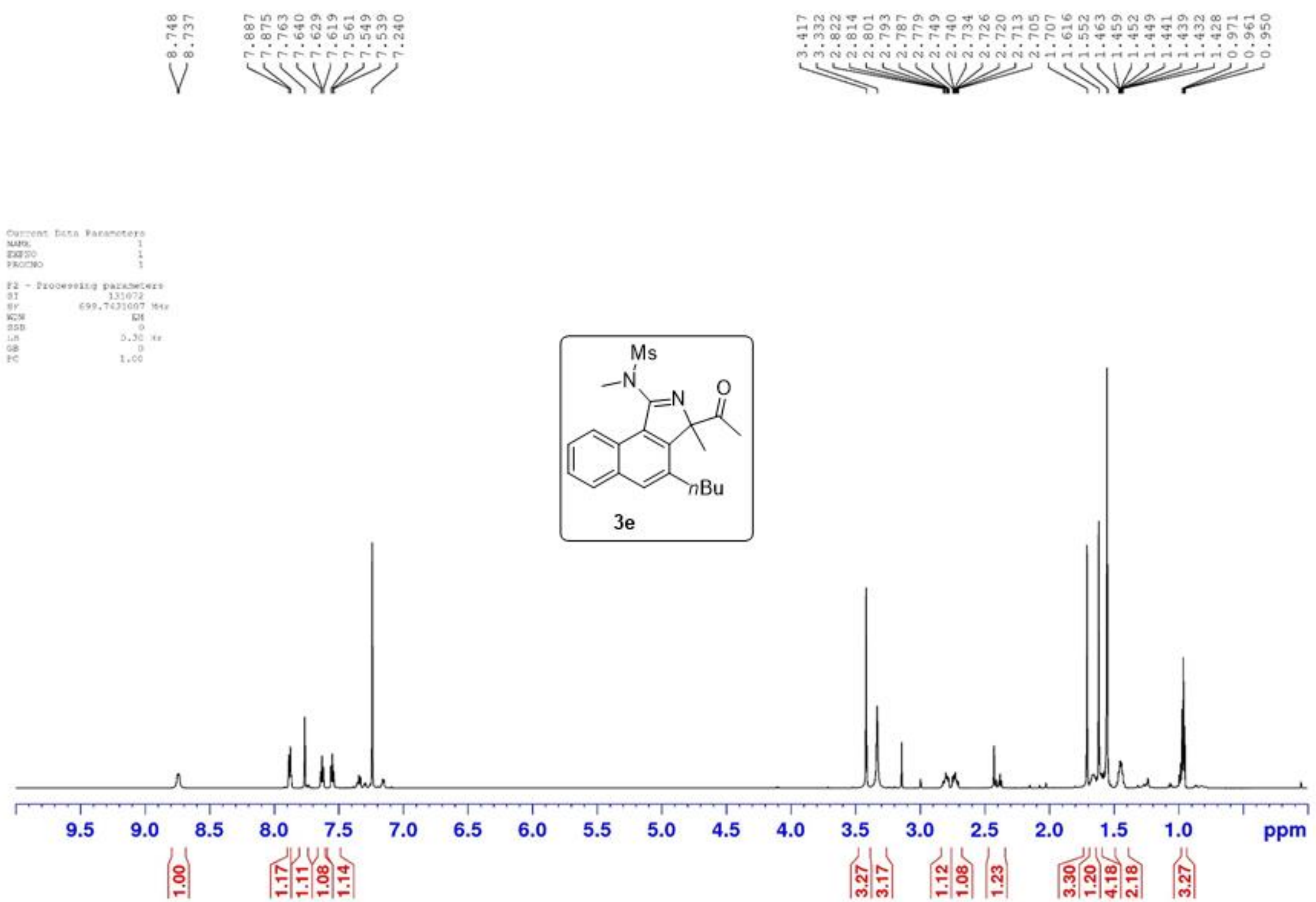

Solvent: $CDCl_3$
SFO1: 100 MHz

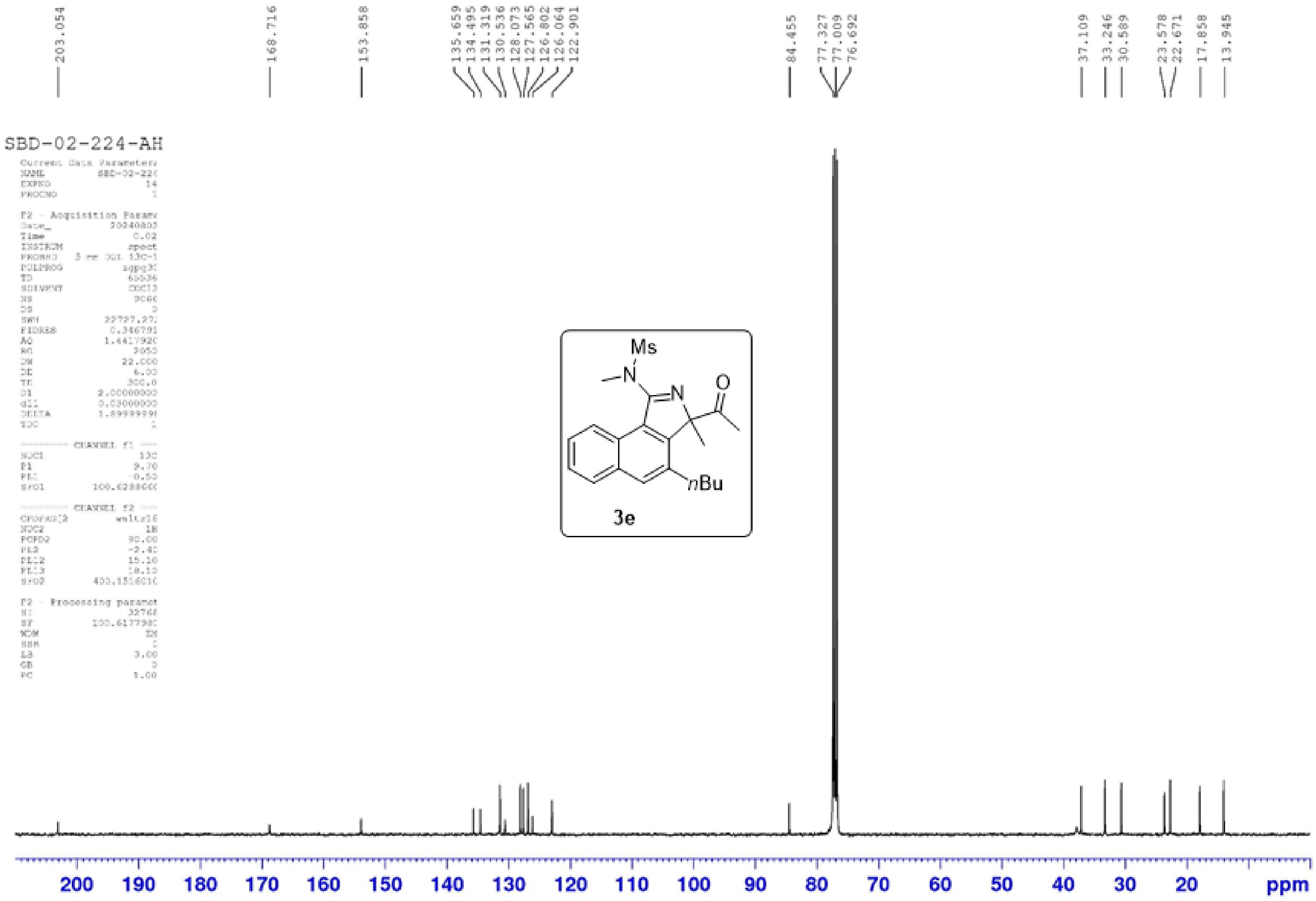

Solvent: $CDCl_3$
SFO1: 700 MHz

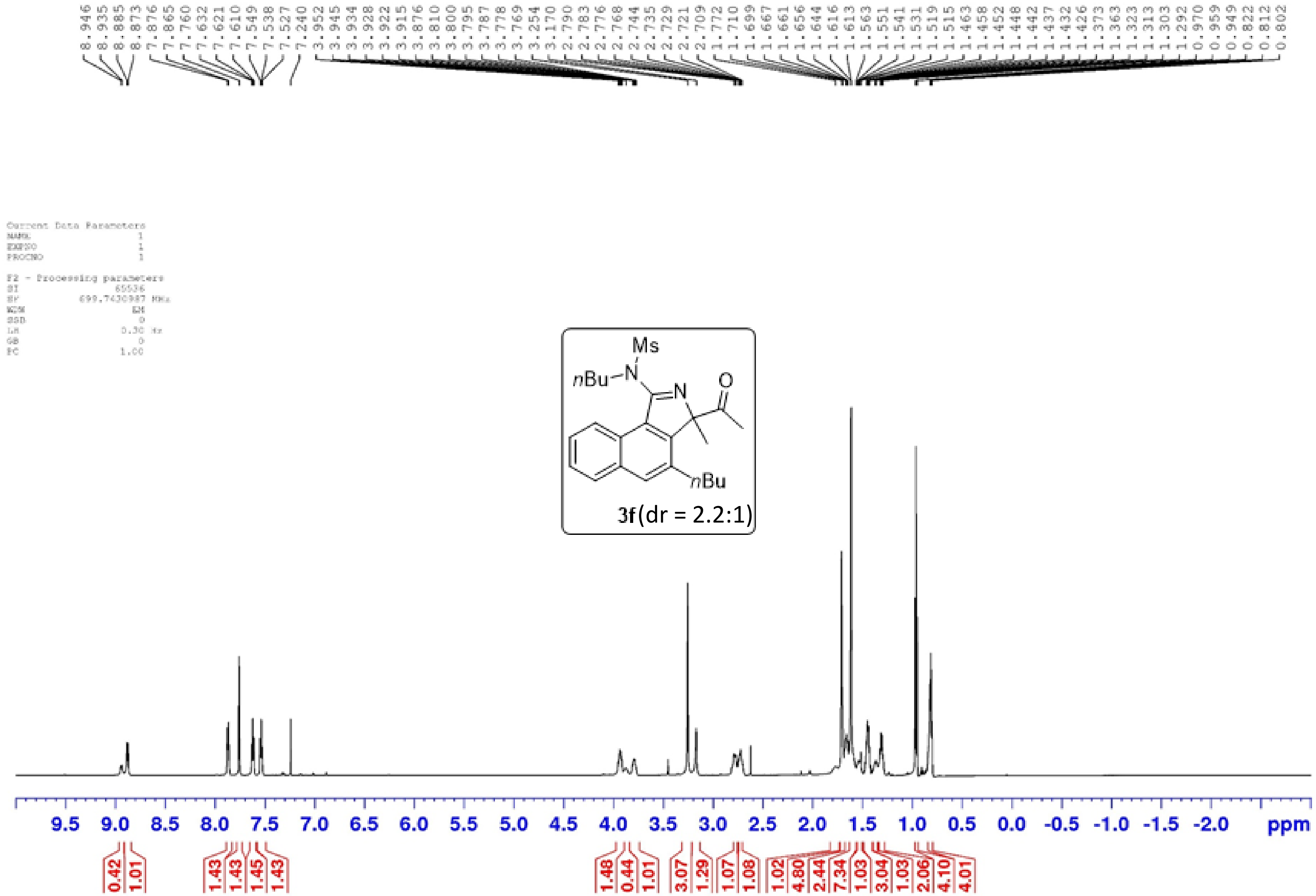

Solvent: $CDCl_3$
SFO1: 175 MHz

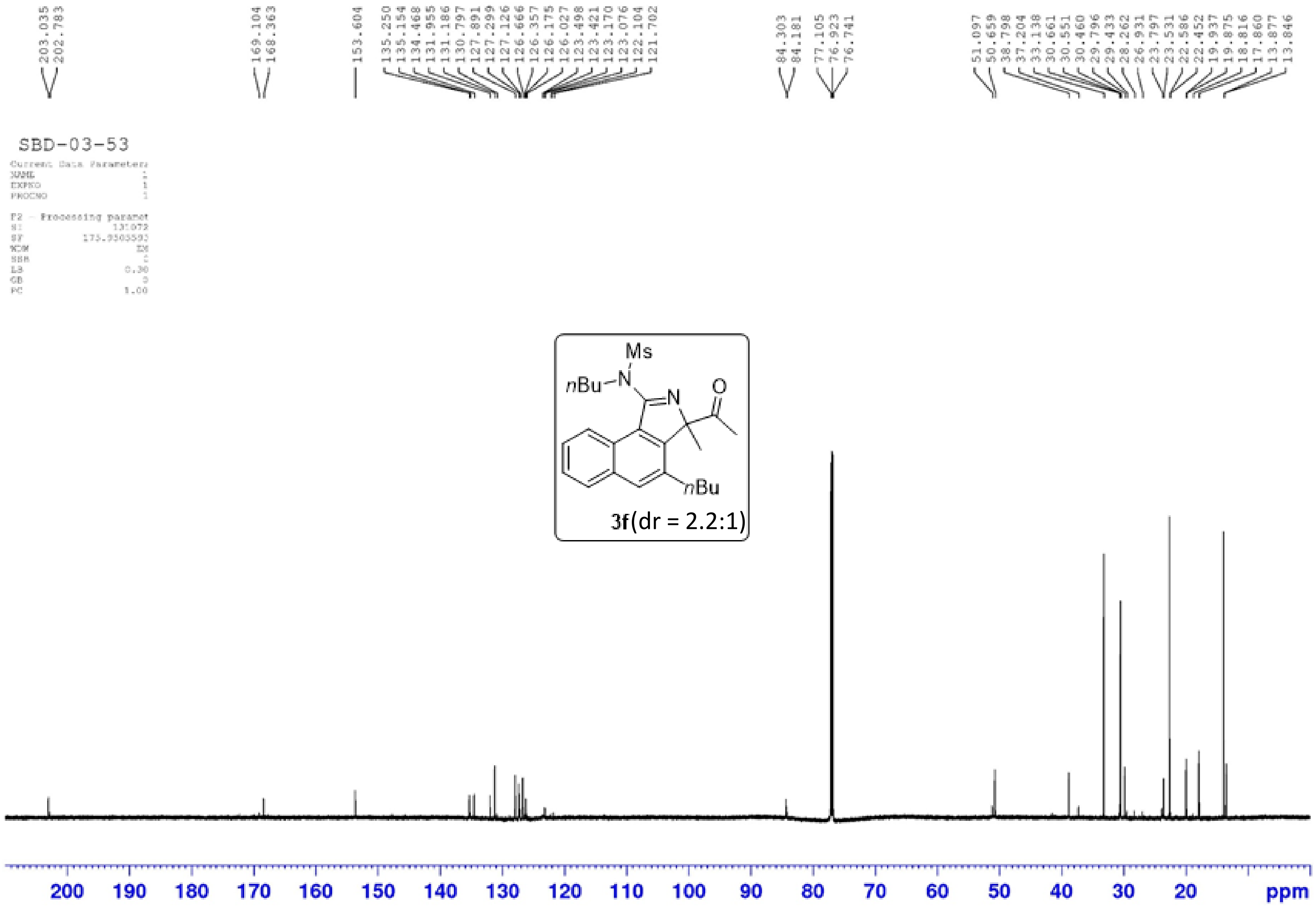

3f(dr = 2.2:1)

Solvent: $CDCl_3$
SFO1: 700 MHz

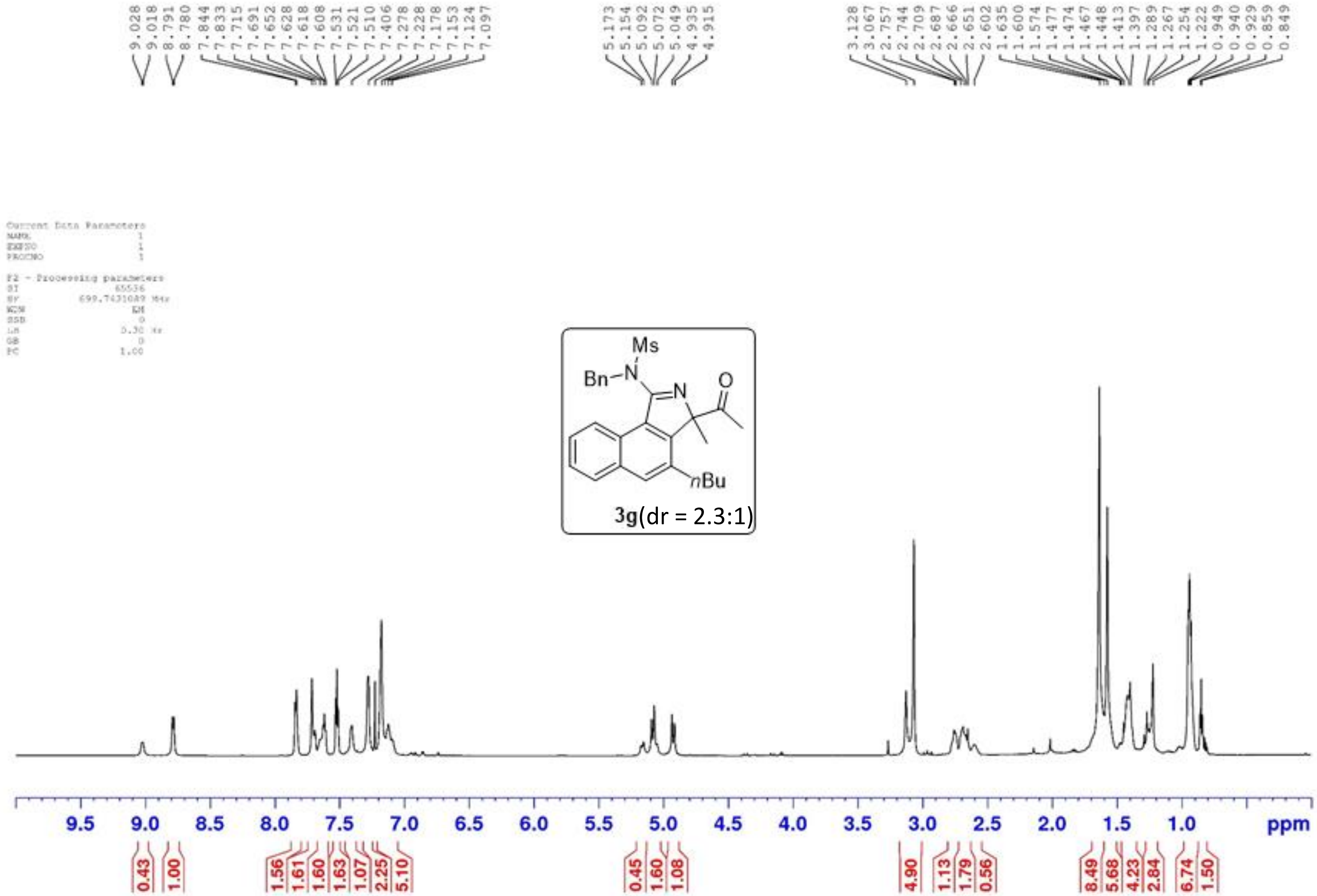

Solvent: $CDCl_3$
SFO1: 175 MHz

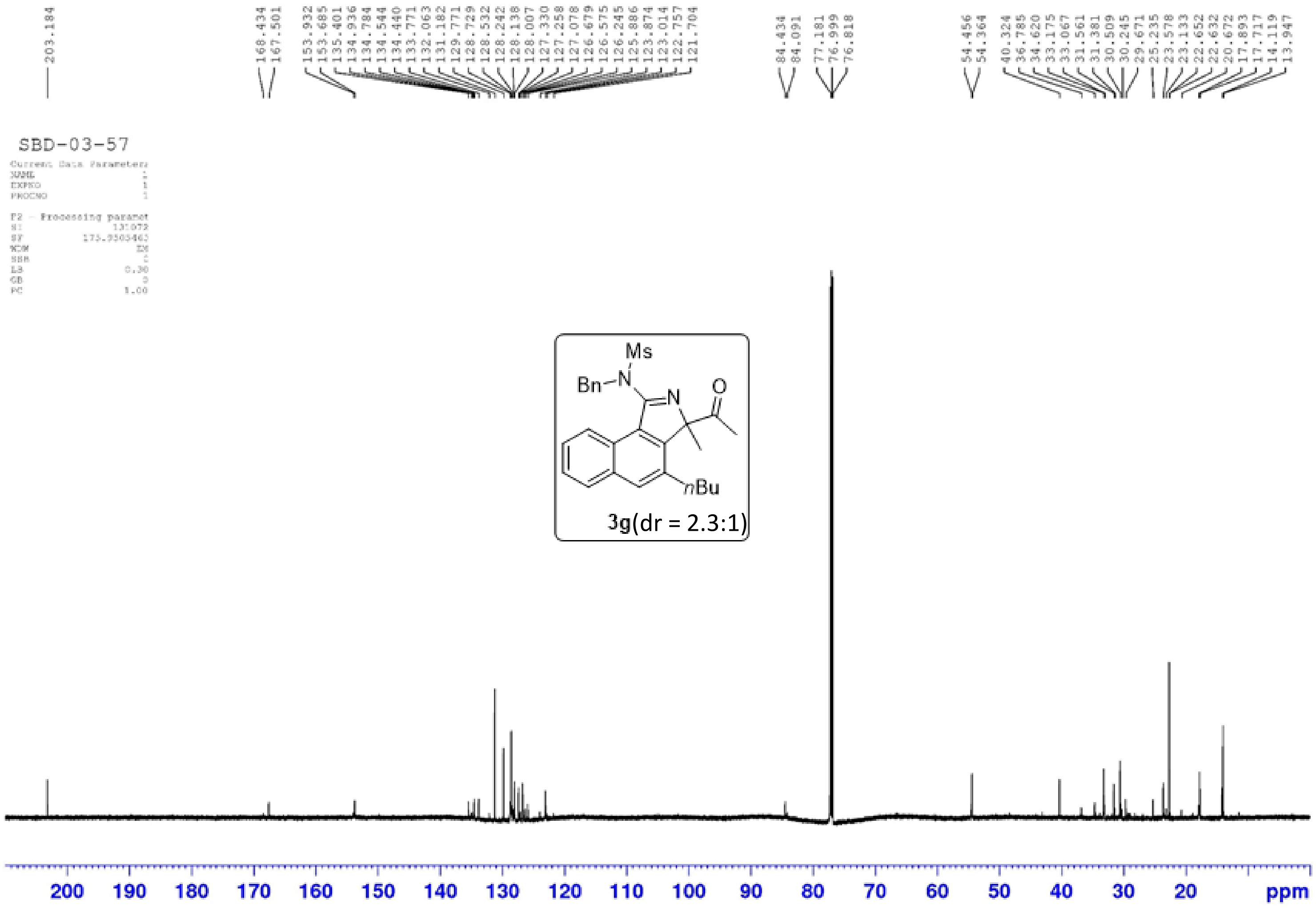

Solvent: $CDCl_3$
SFO1: 400 MHz

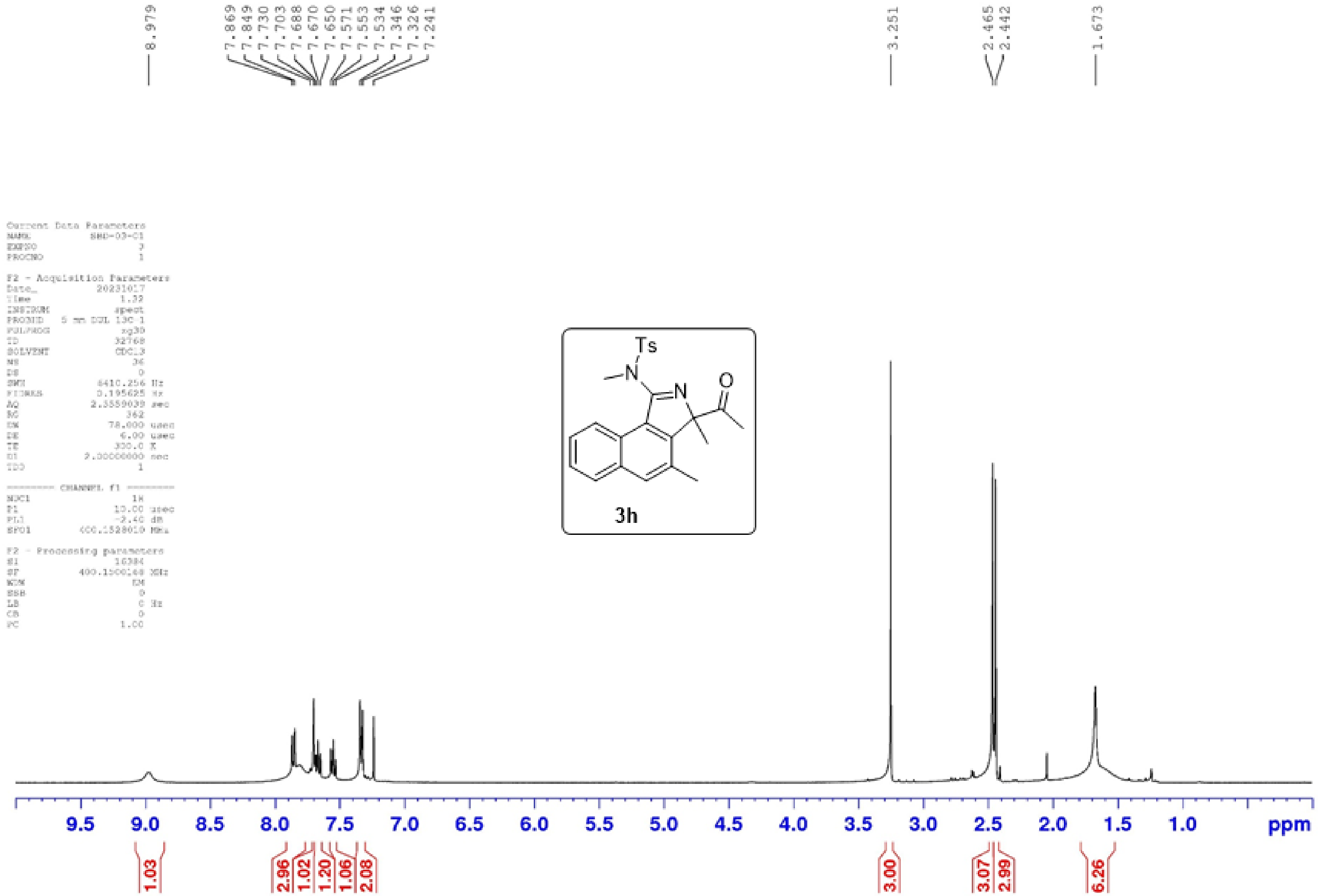

Solvent: $CDCl_3$
SFO1: 100 MHz

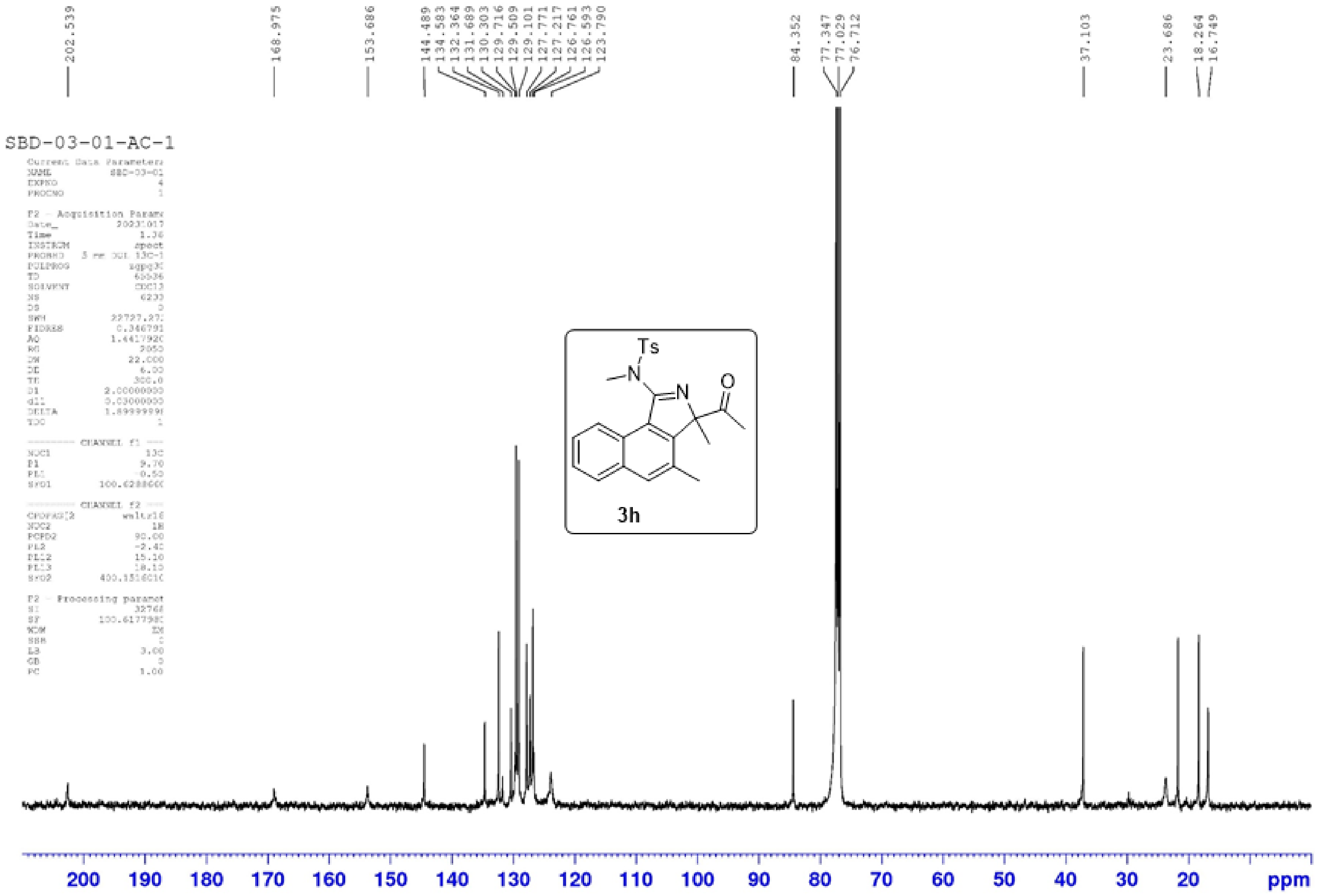

Solvent: $CDCl_3$
SFO1: 700 MHz

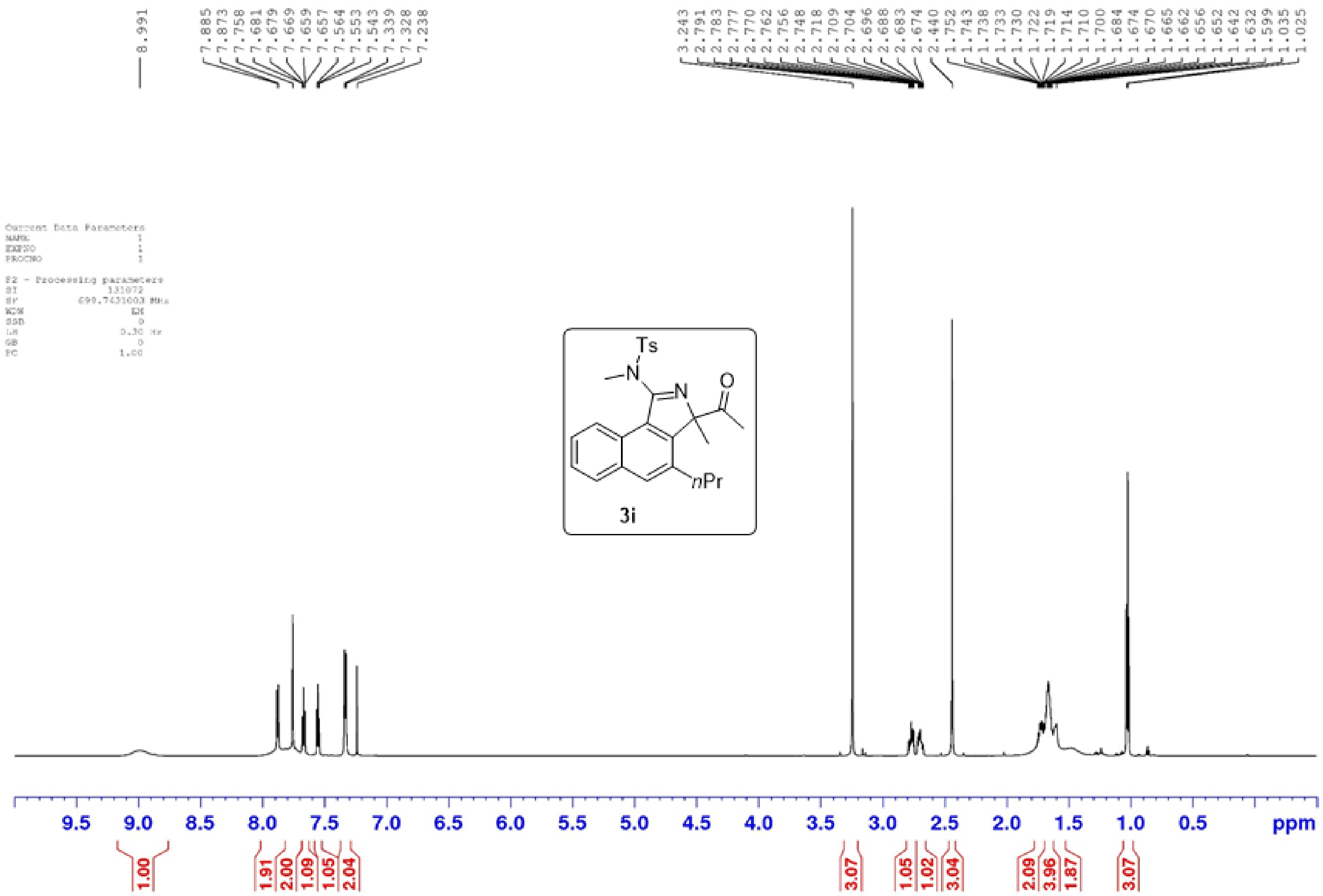

Solvent: $CDCl_3$
SFO1: 100 MHz

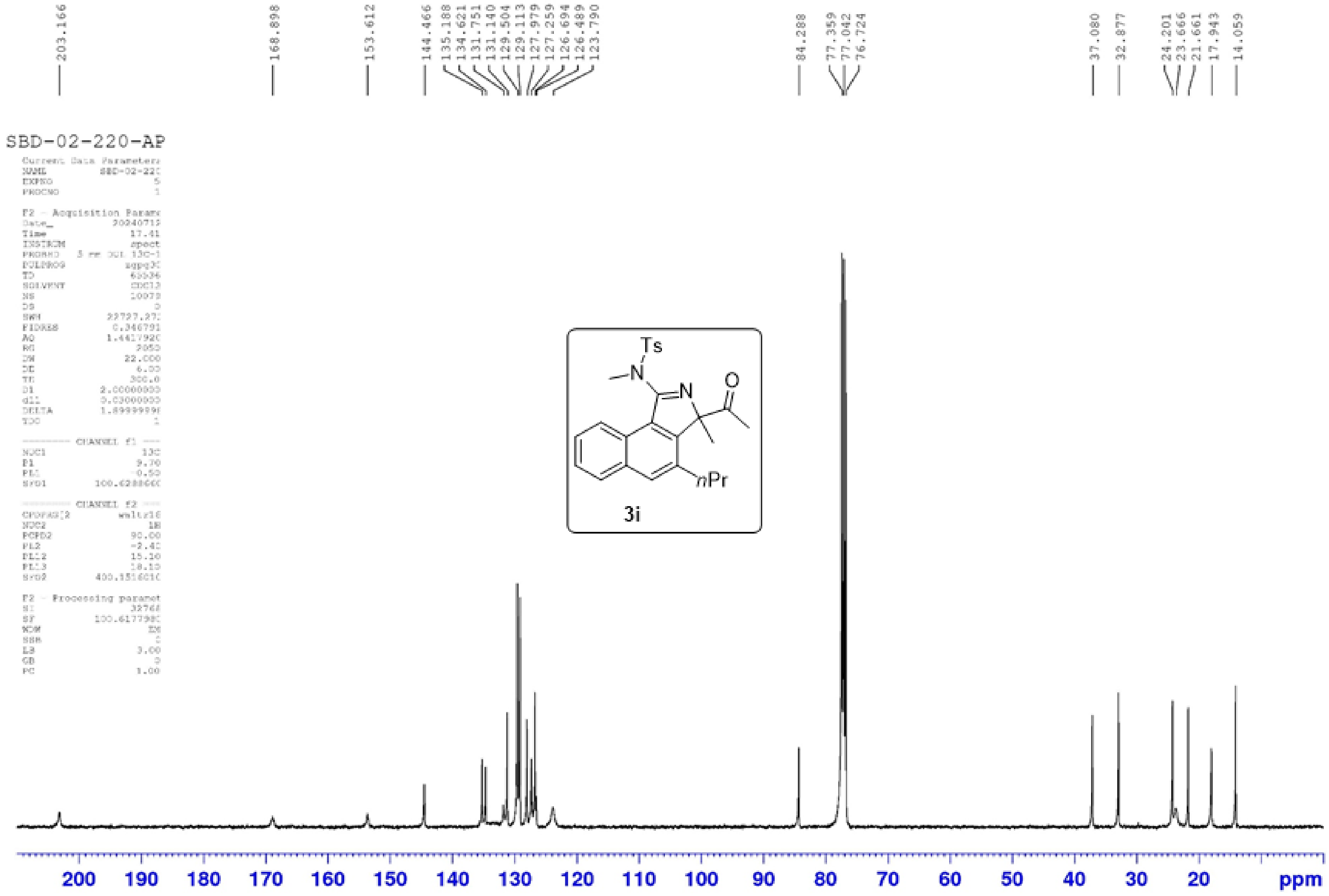

Solvent: $CDCl_3$
SFO1: 400 MHz

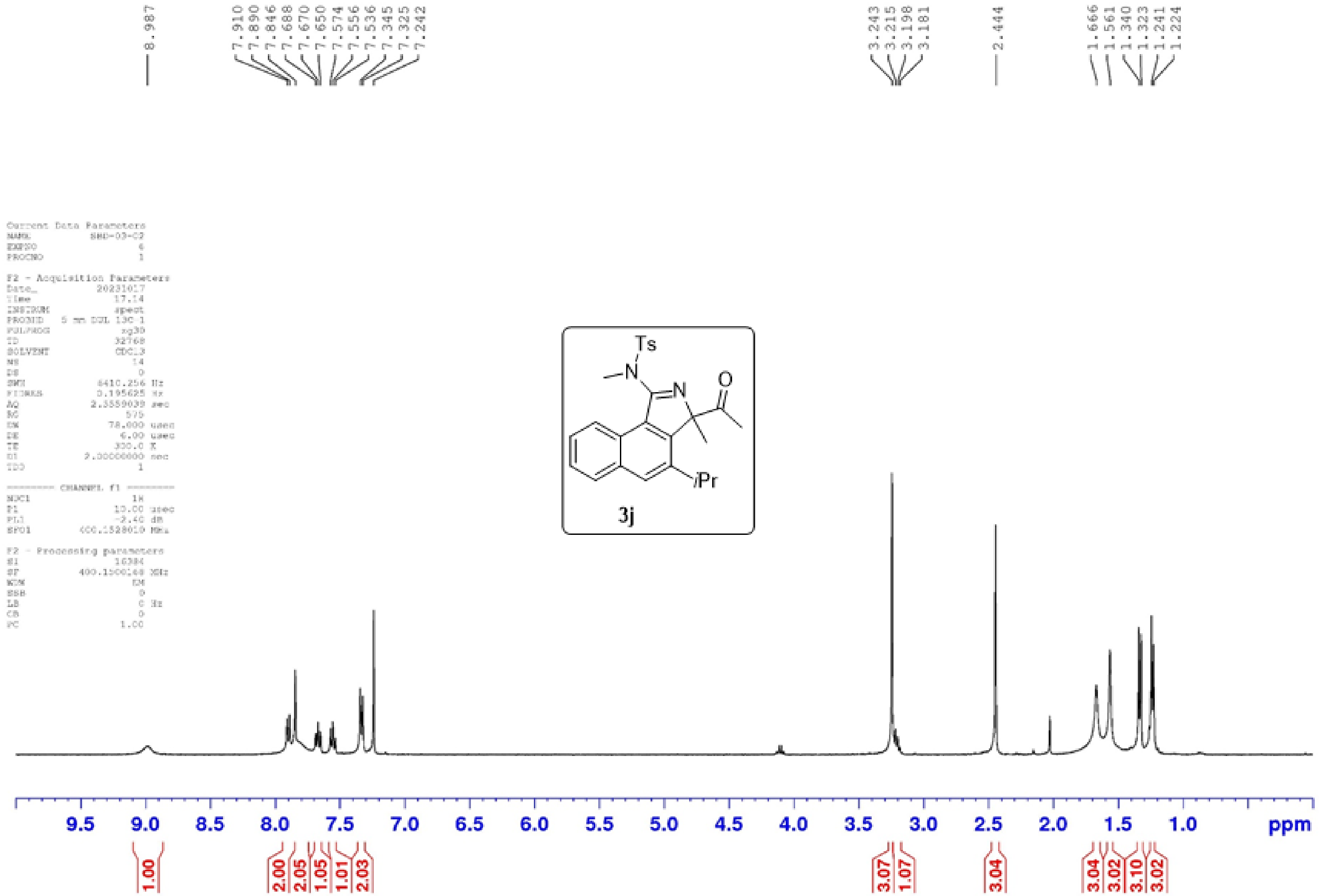

Solvent: $CDCl_3$
SFO1: 100 MHz

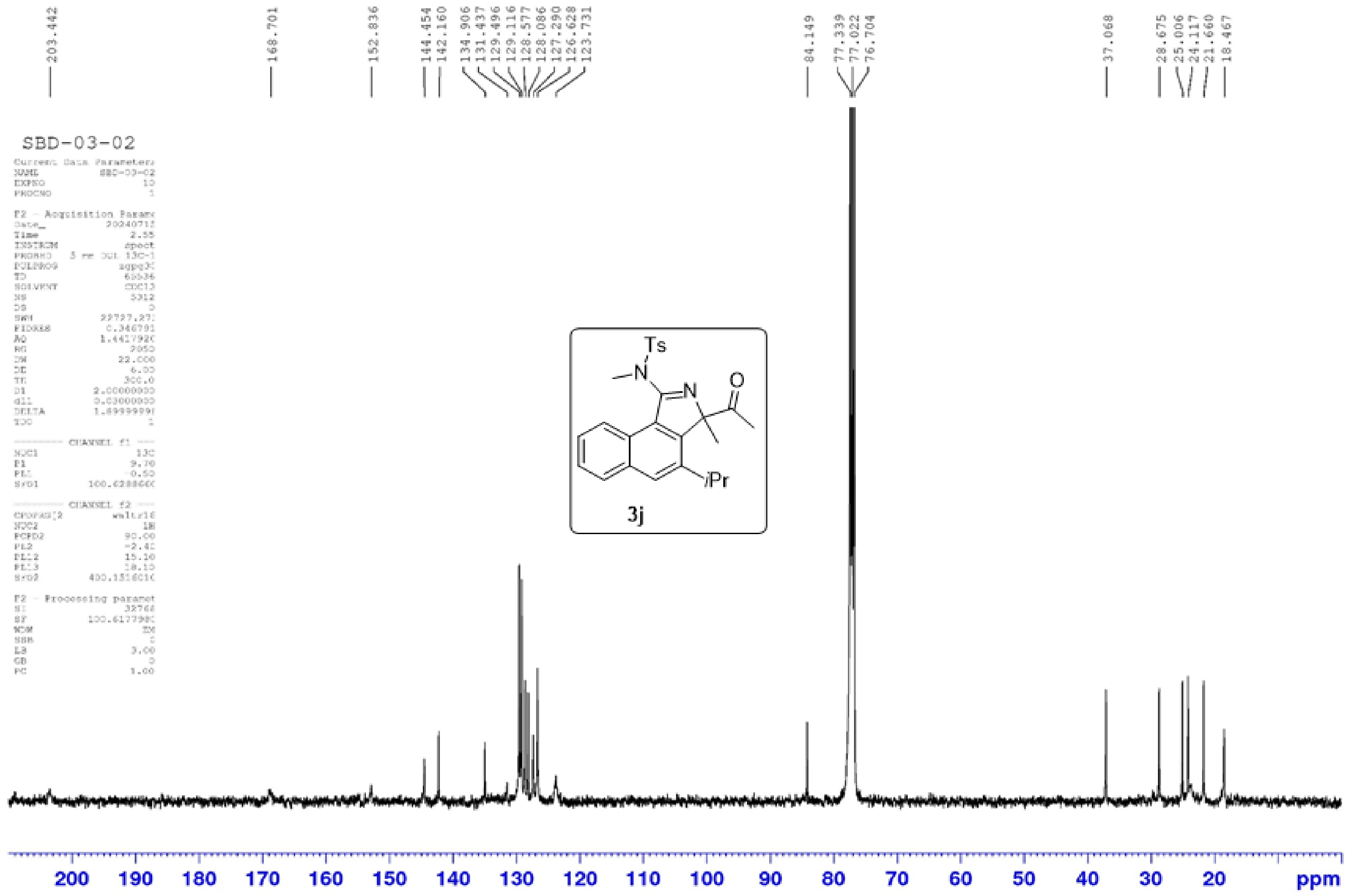

Solvent: $CDCl_3$
SFO1: 700 MHz

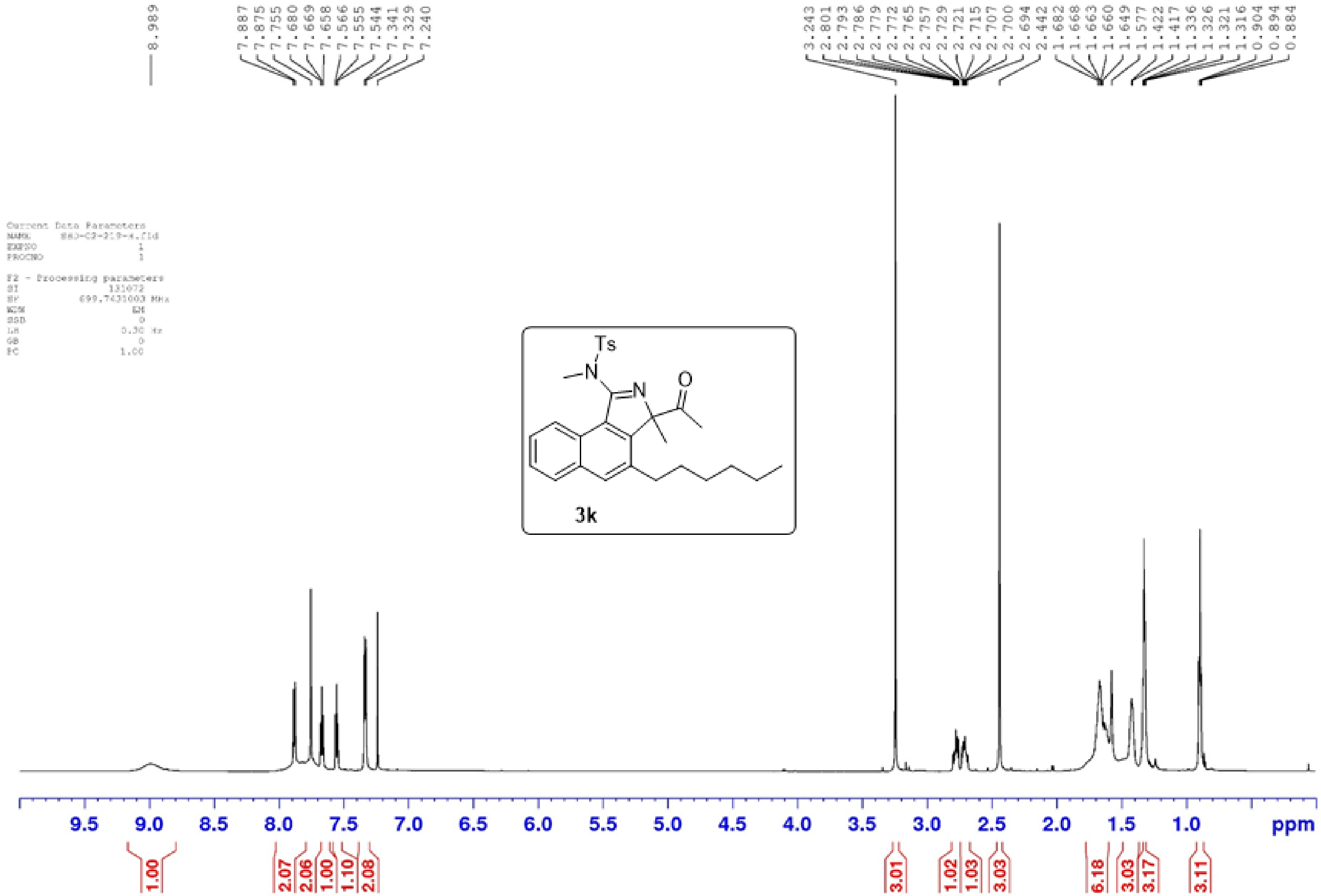

Solvent: $CDCl_3$
SFO1: 100 MHz

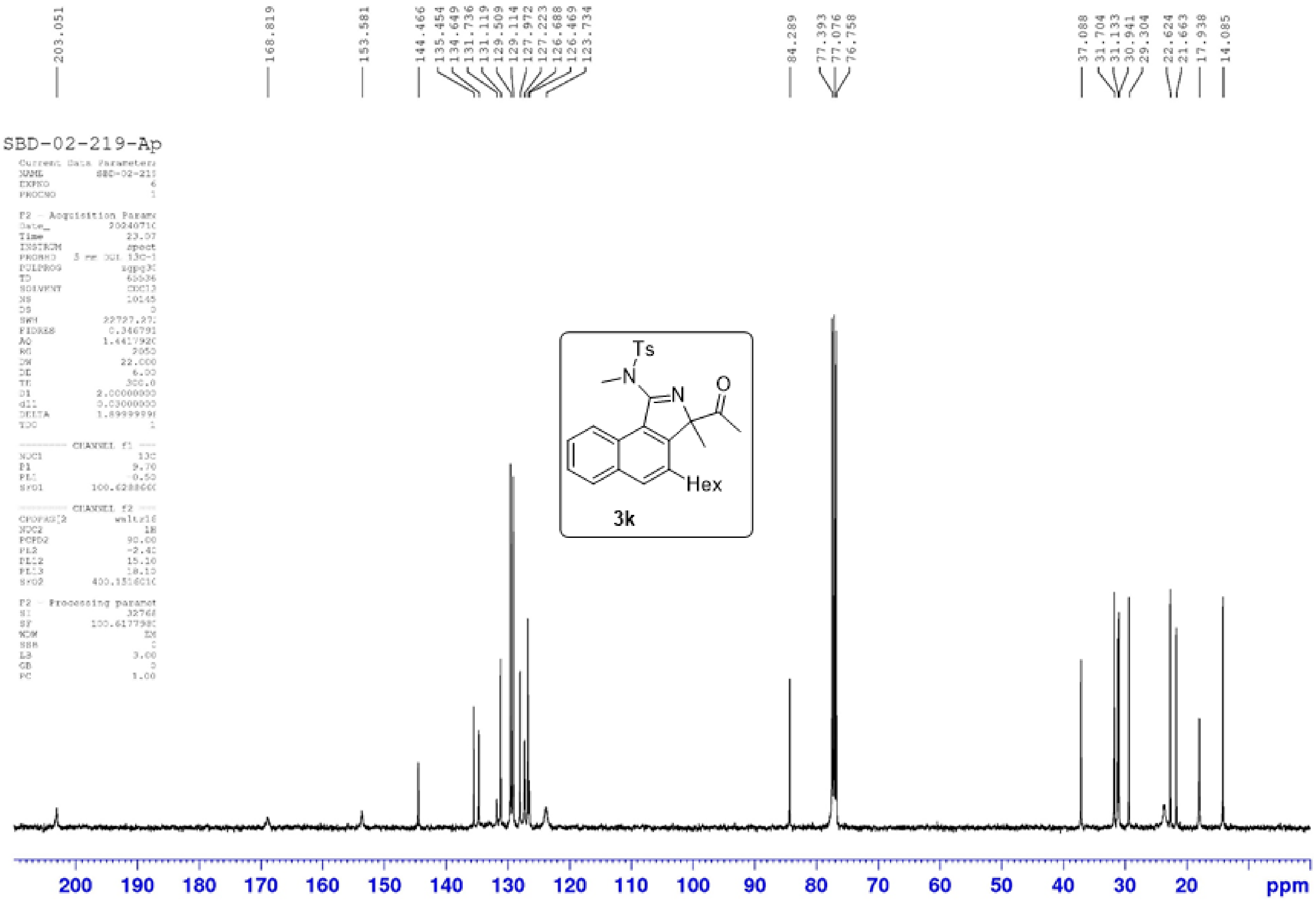

Solvent: $CDCl_3$
SFO1: 700 MHz

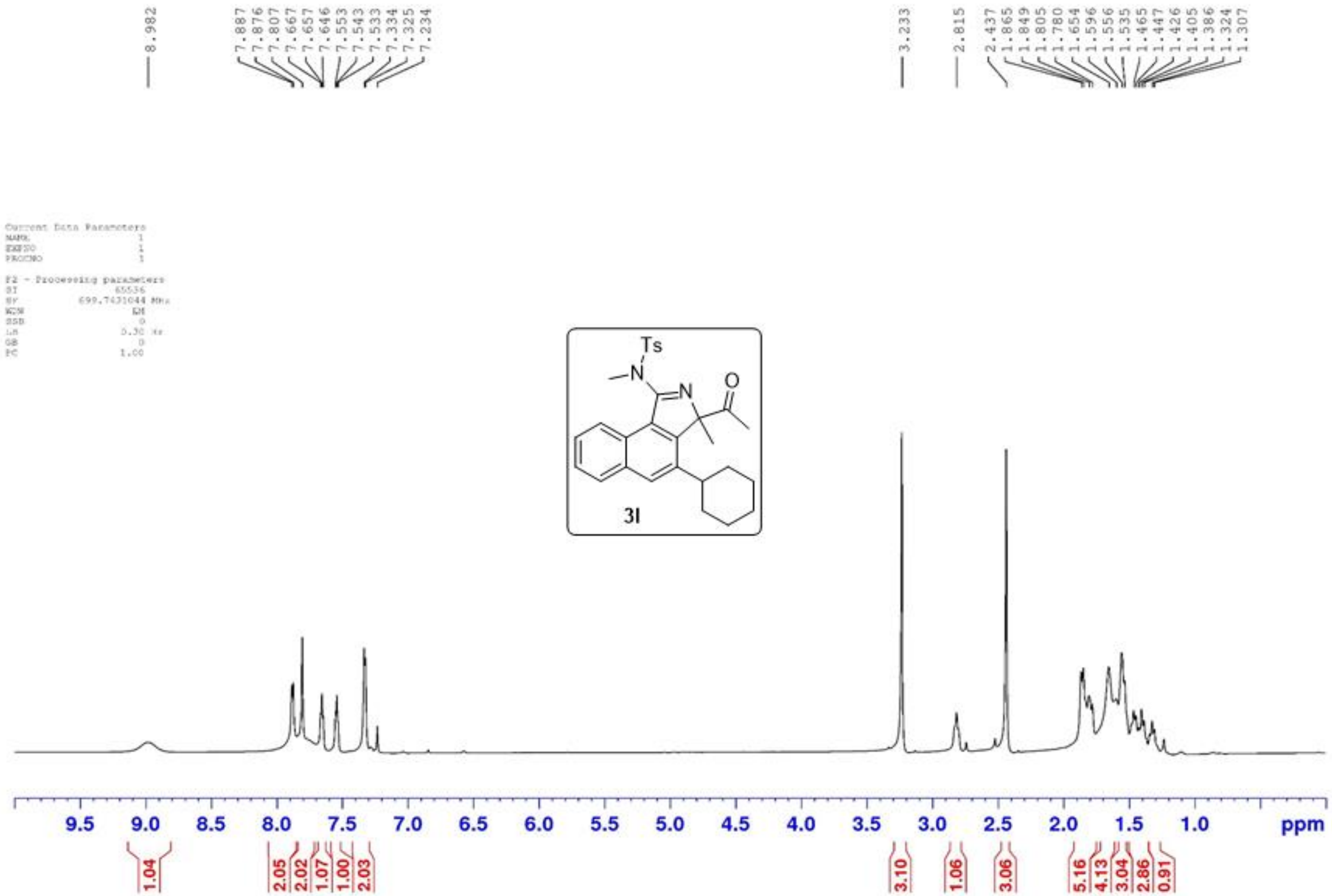

Solvent: $CDCl_3$
SFO1: 100 MHz

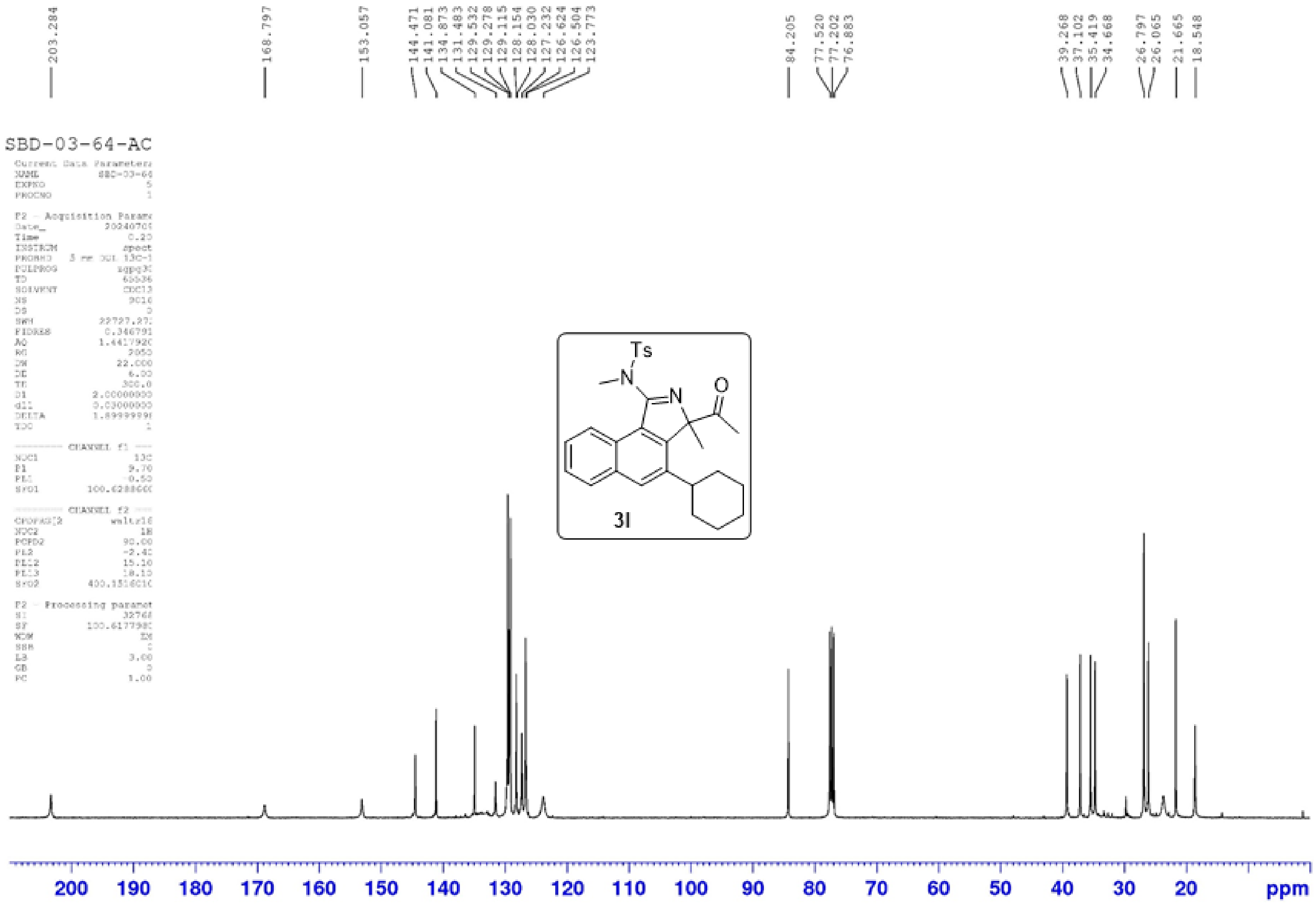

Solvent: $CDCl_3$
SFO1: 700 MHz

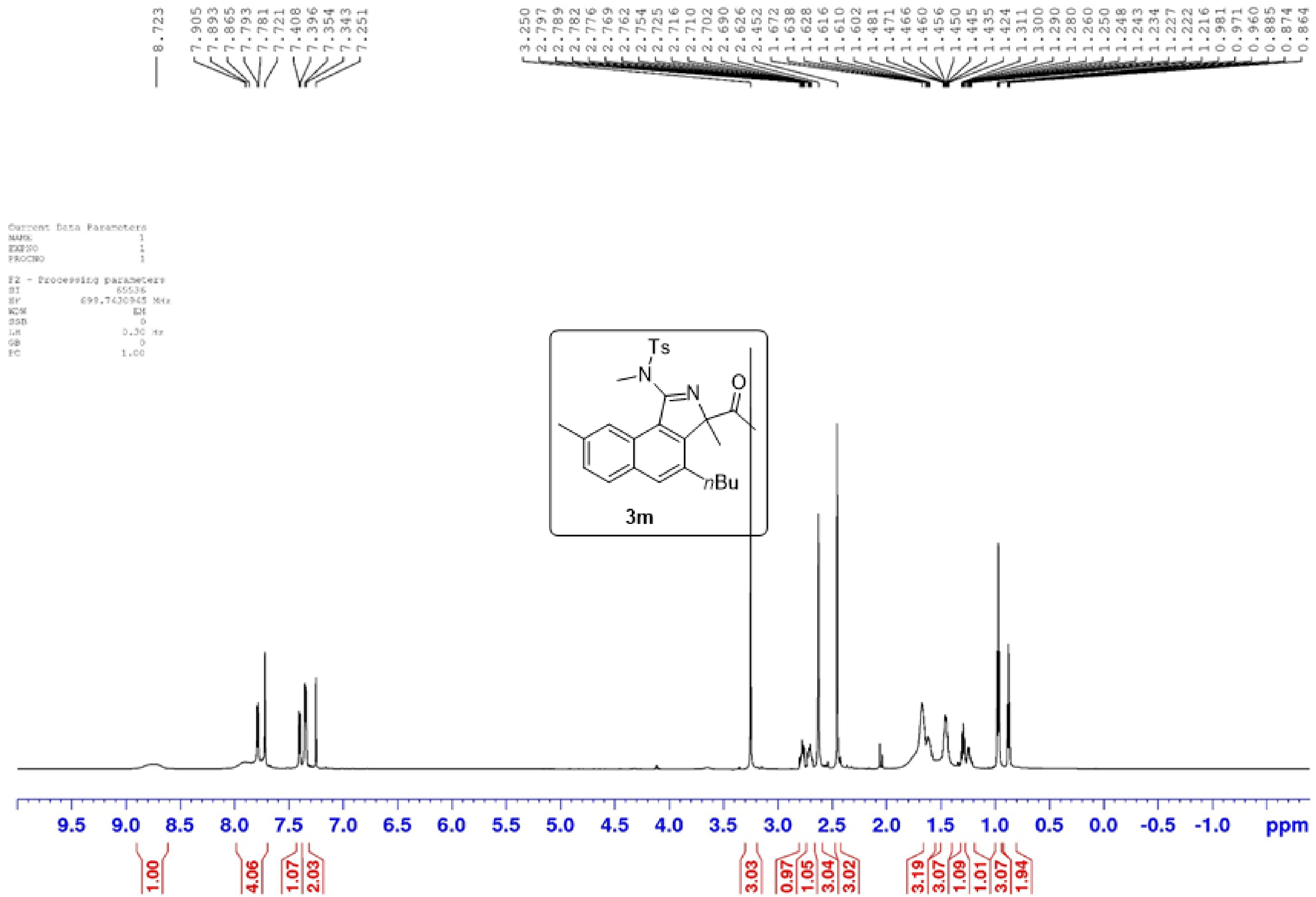

Solvent: $CDCl_3$
SFO1: 100 MHz

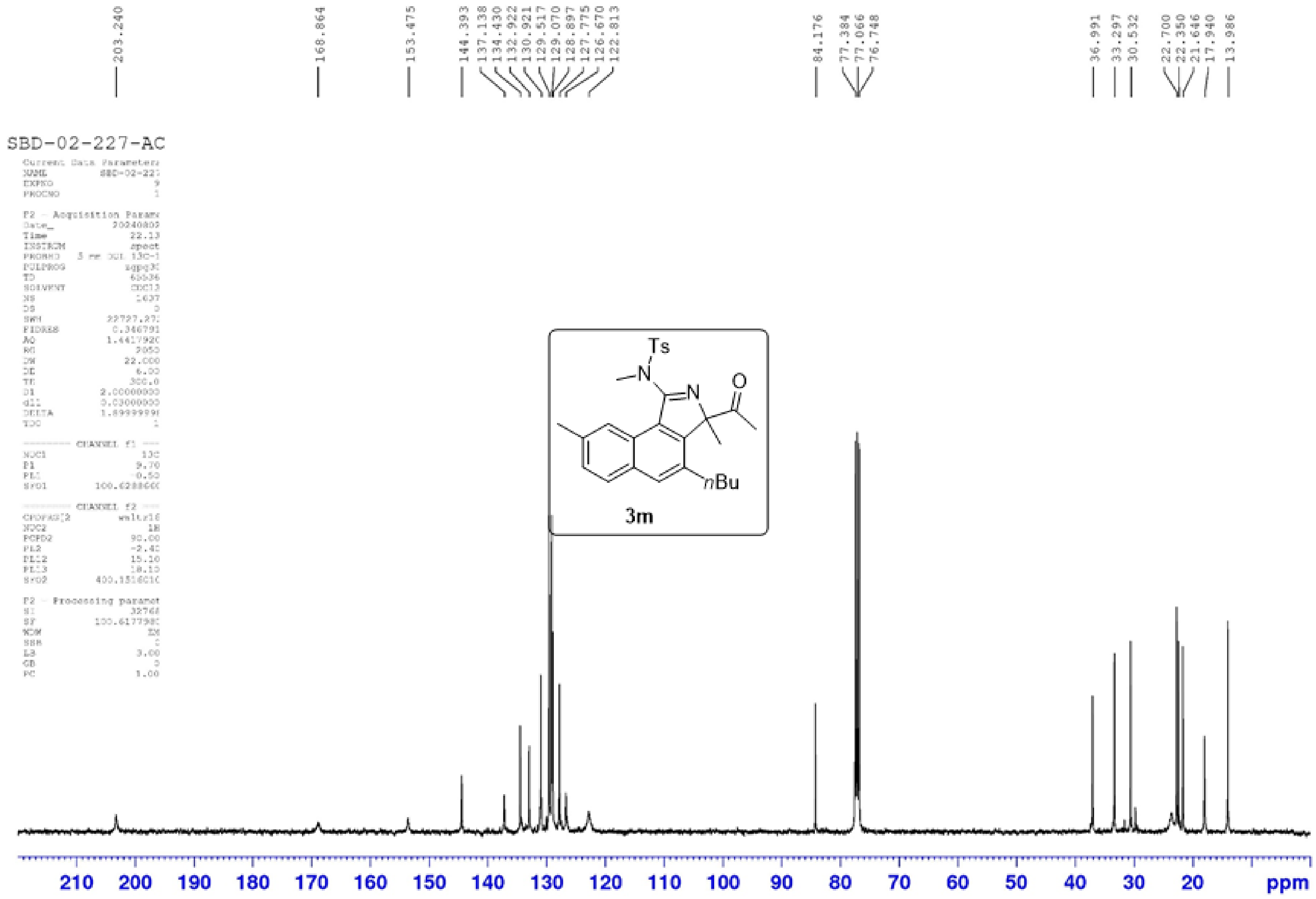

Solvent: $CDCl_3$
SFO1: 400 MHz

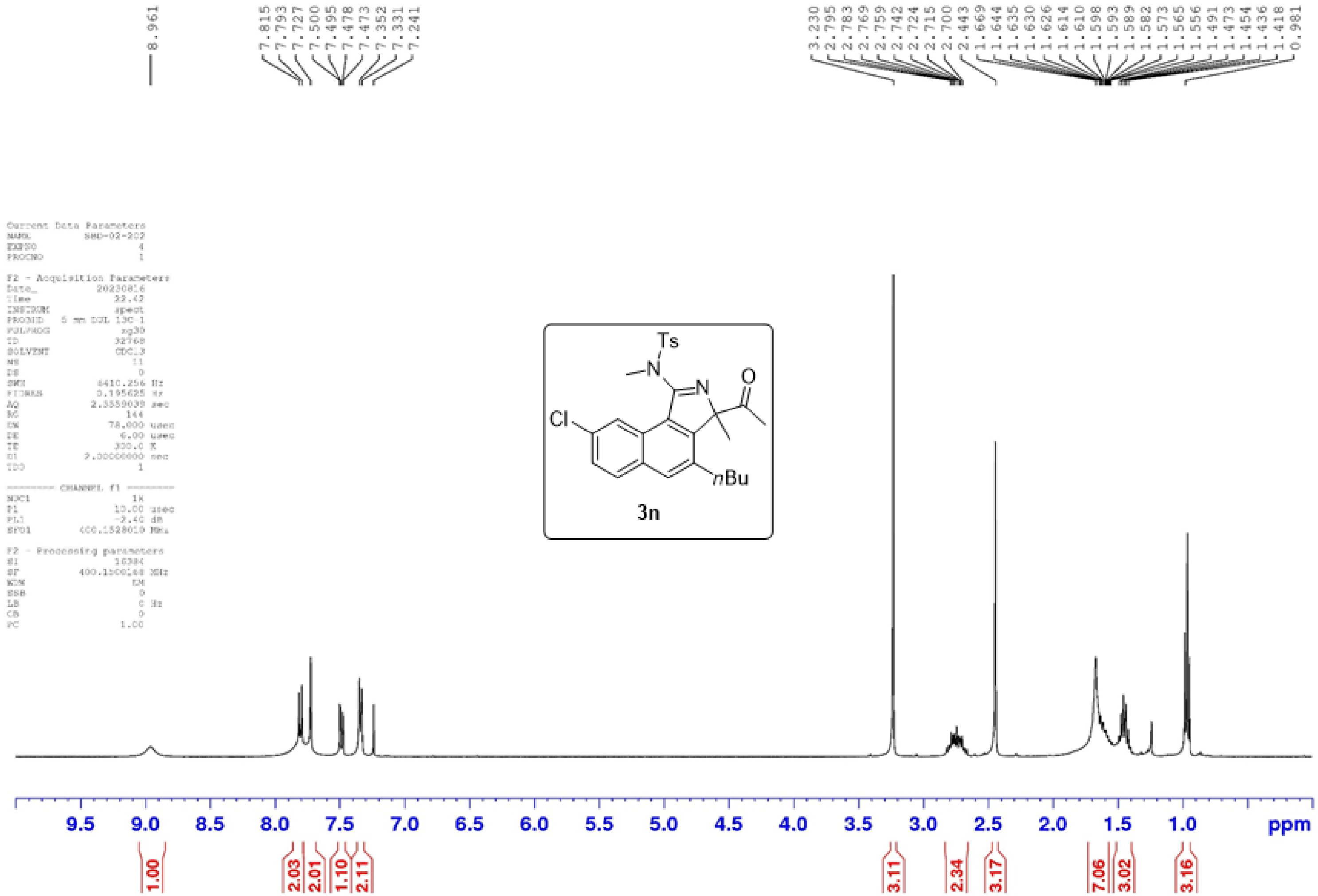

Solvent: $CDCl_3$
SFO1: 100 MHz

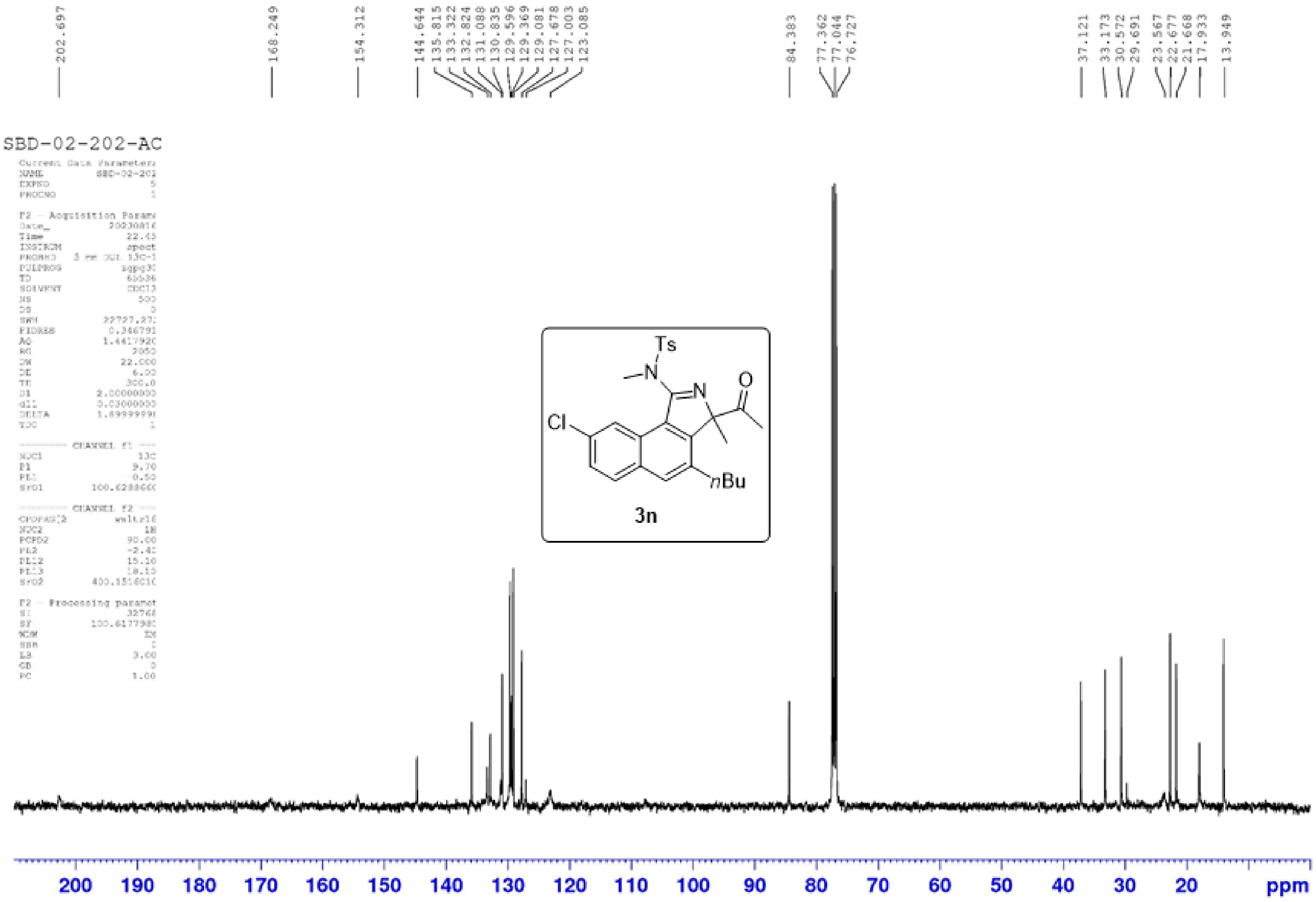

Solvent: $CDCl_3$
SFO1: 400 MHz

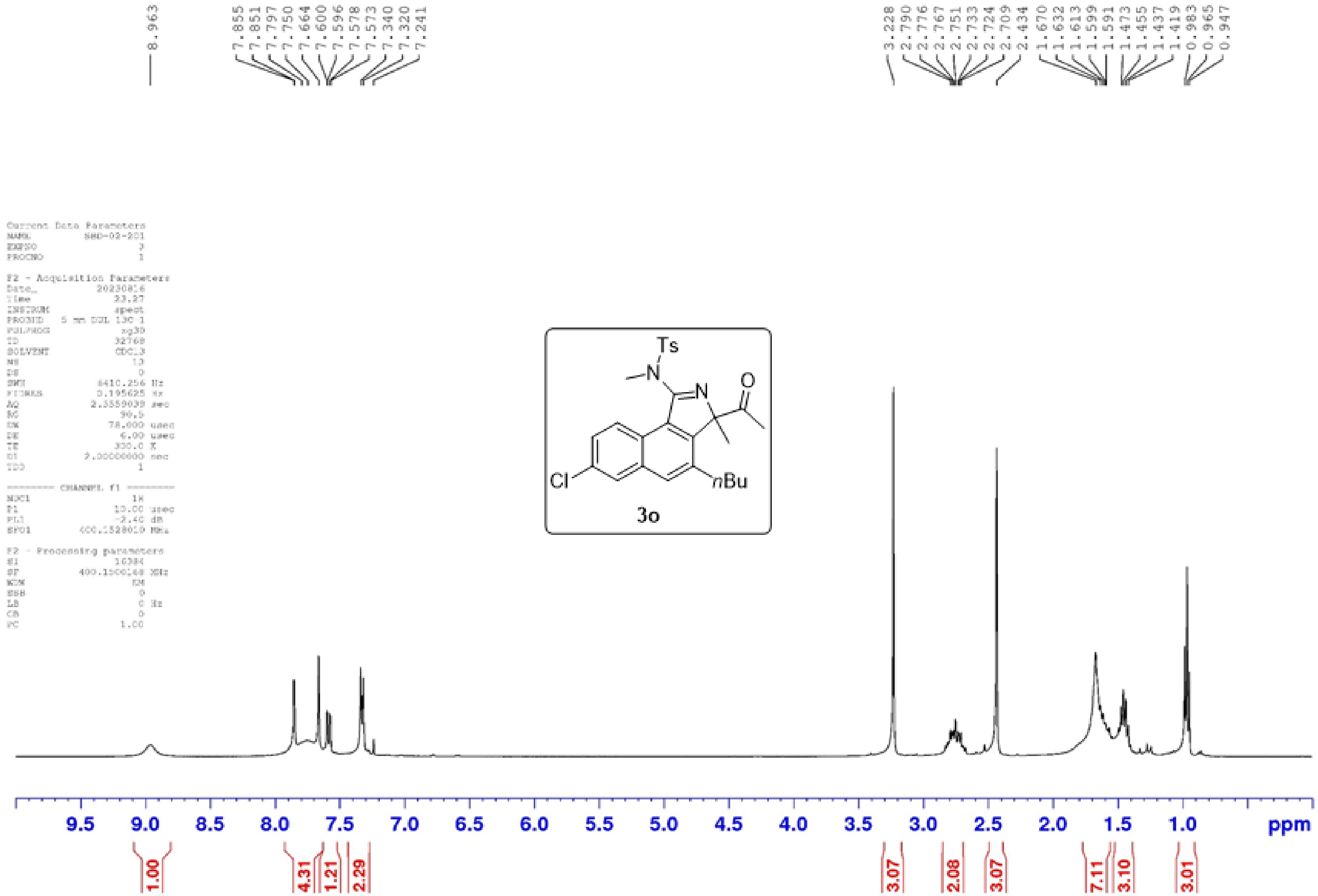

Solvent: $CDCl_3$
SFO1: 100 MHz

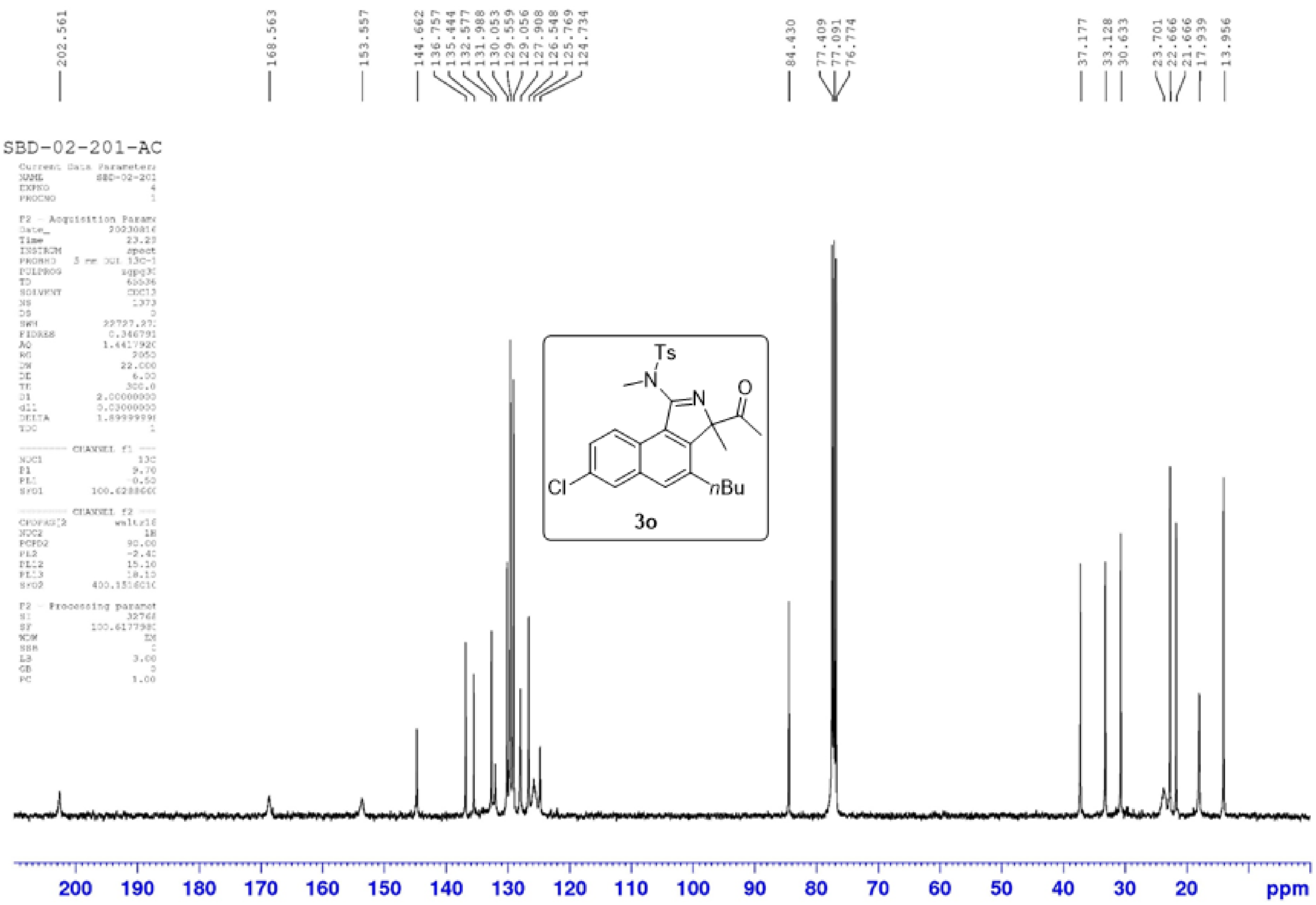

Solvent: $CDCl_3$
SFO1: 700 MHz

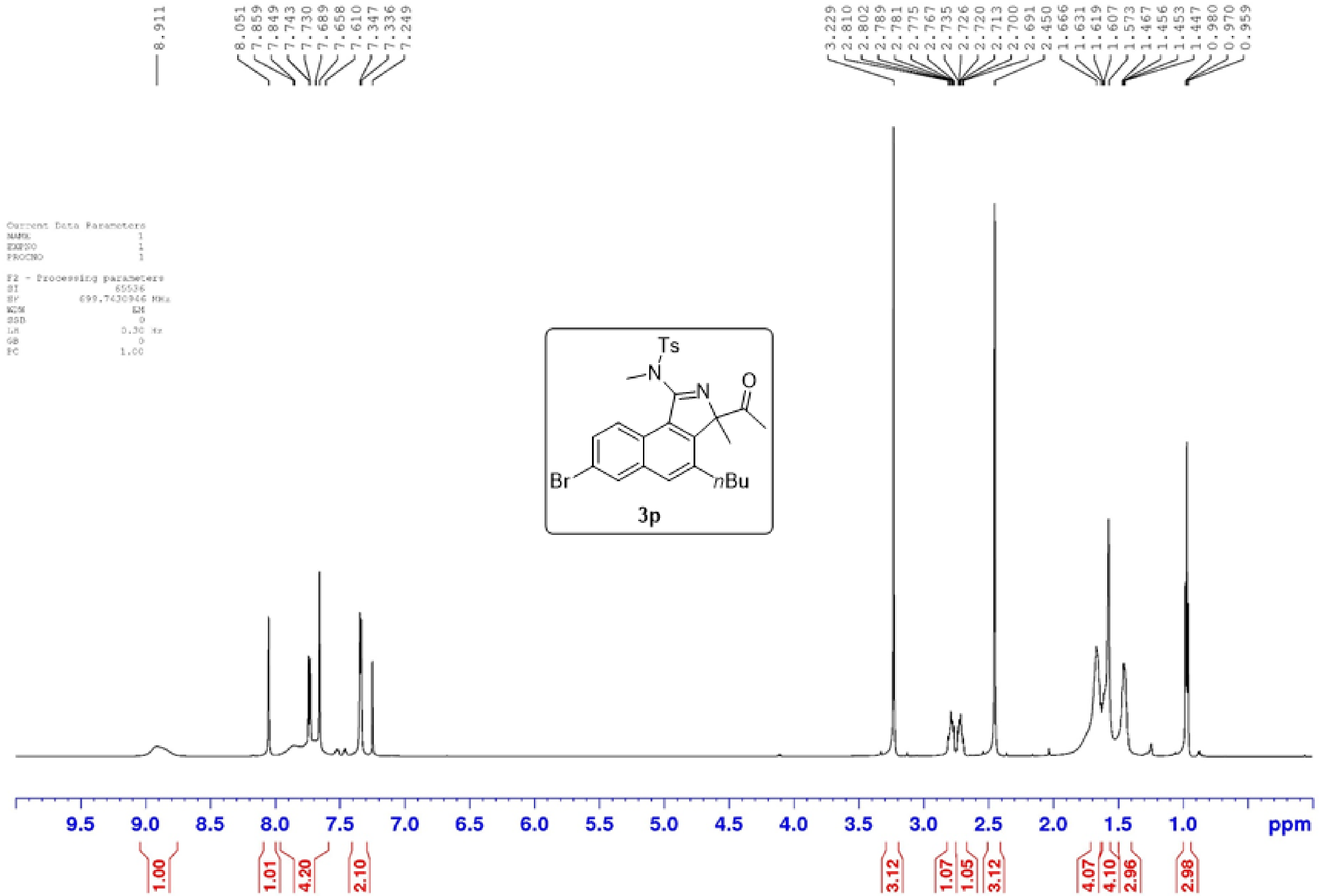

Solvent: $CDCl_3$
SFO1: 400 MHz

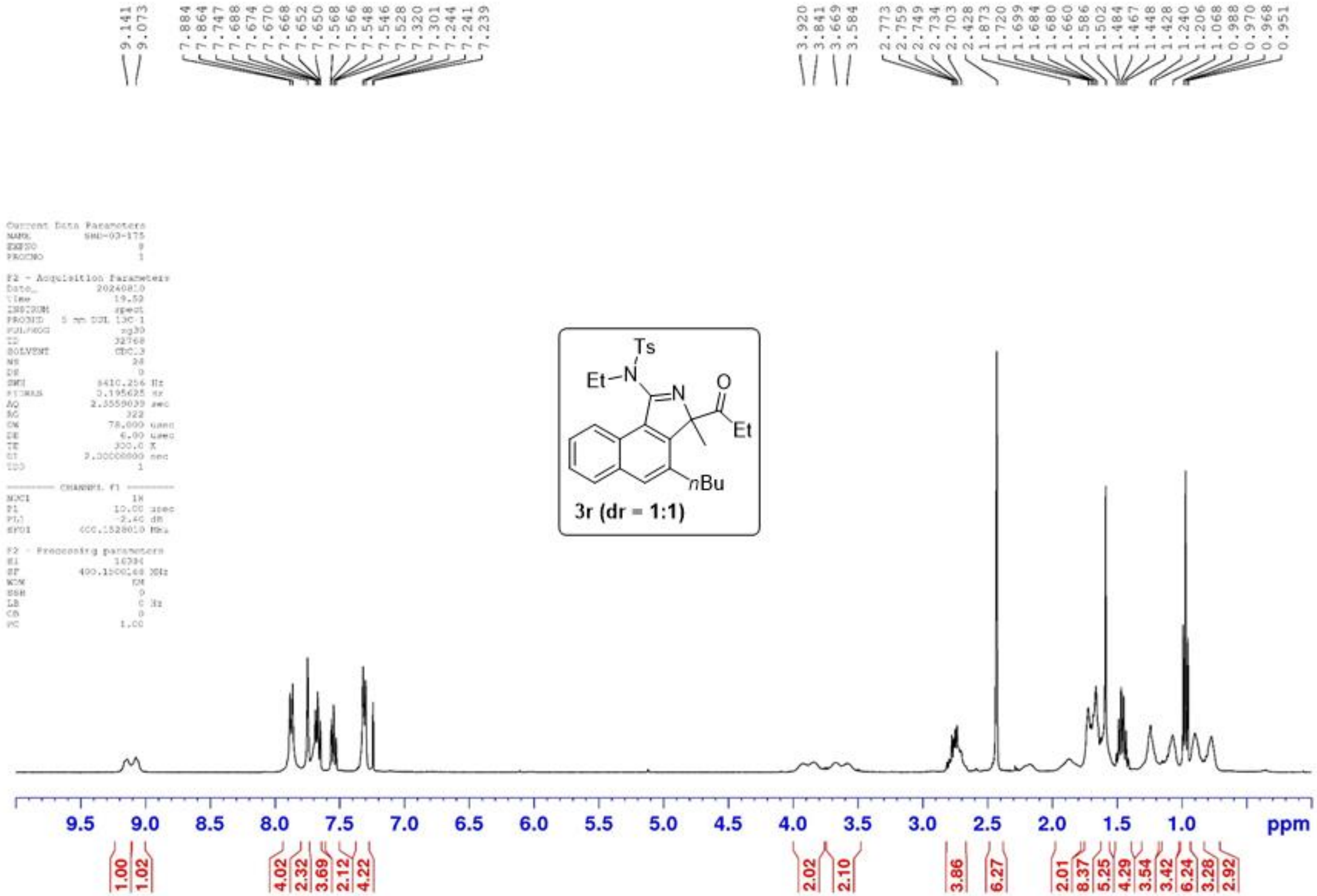

Solvent: $CDCl_3$
SFO1: 100 MHz

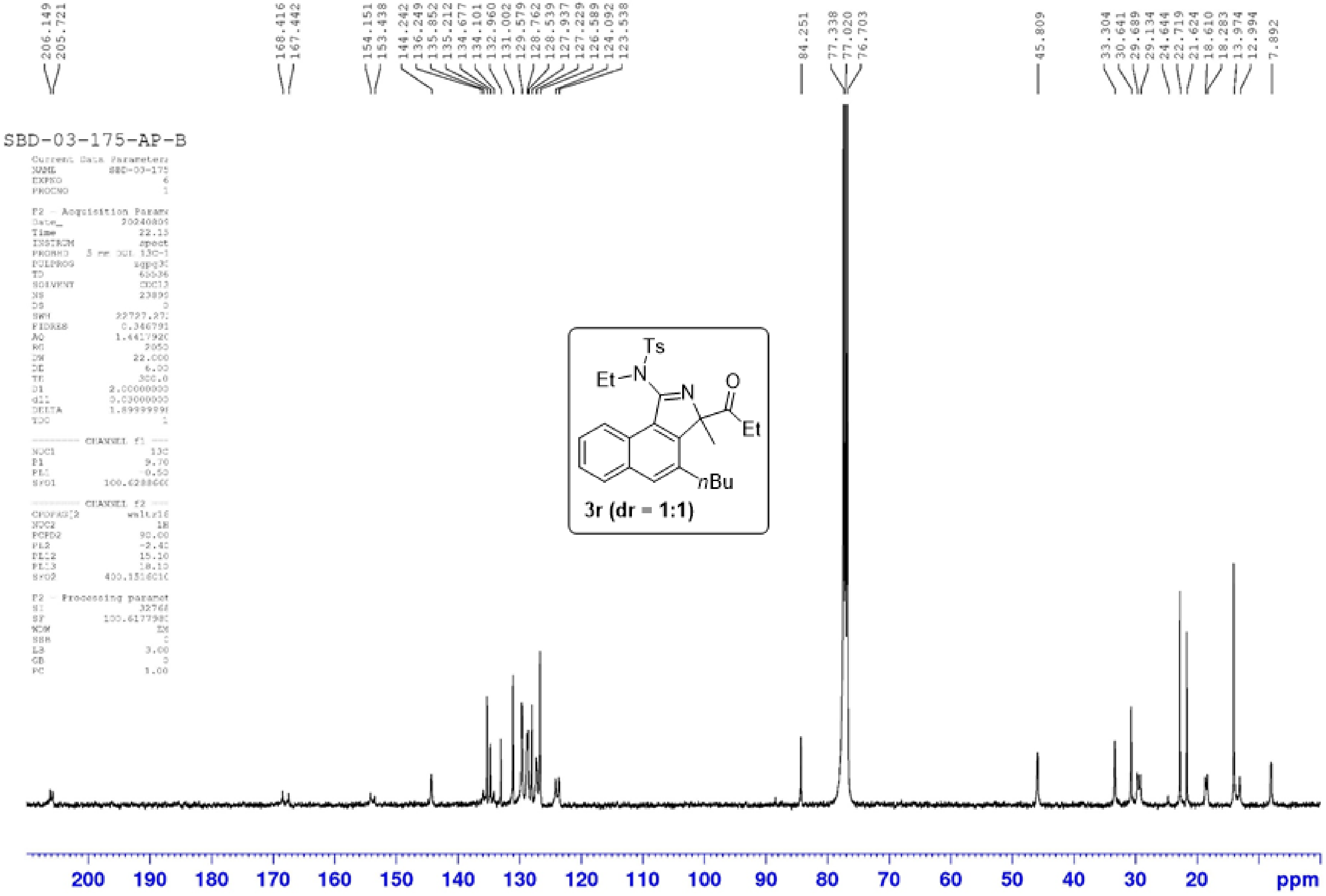

Solvent: $CDCl_3$
SFO1: 175 MHz

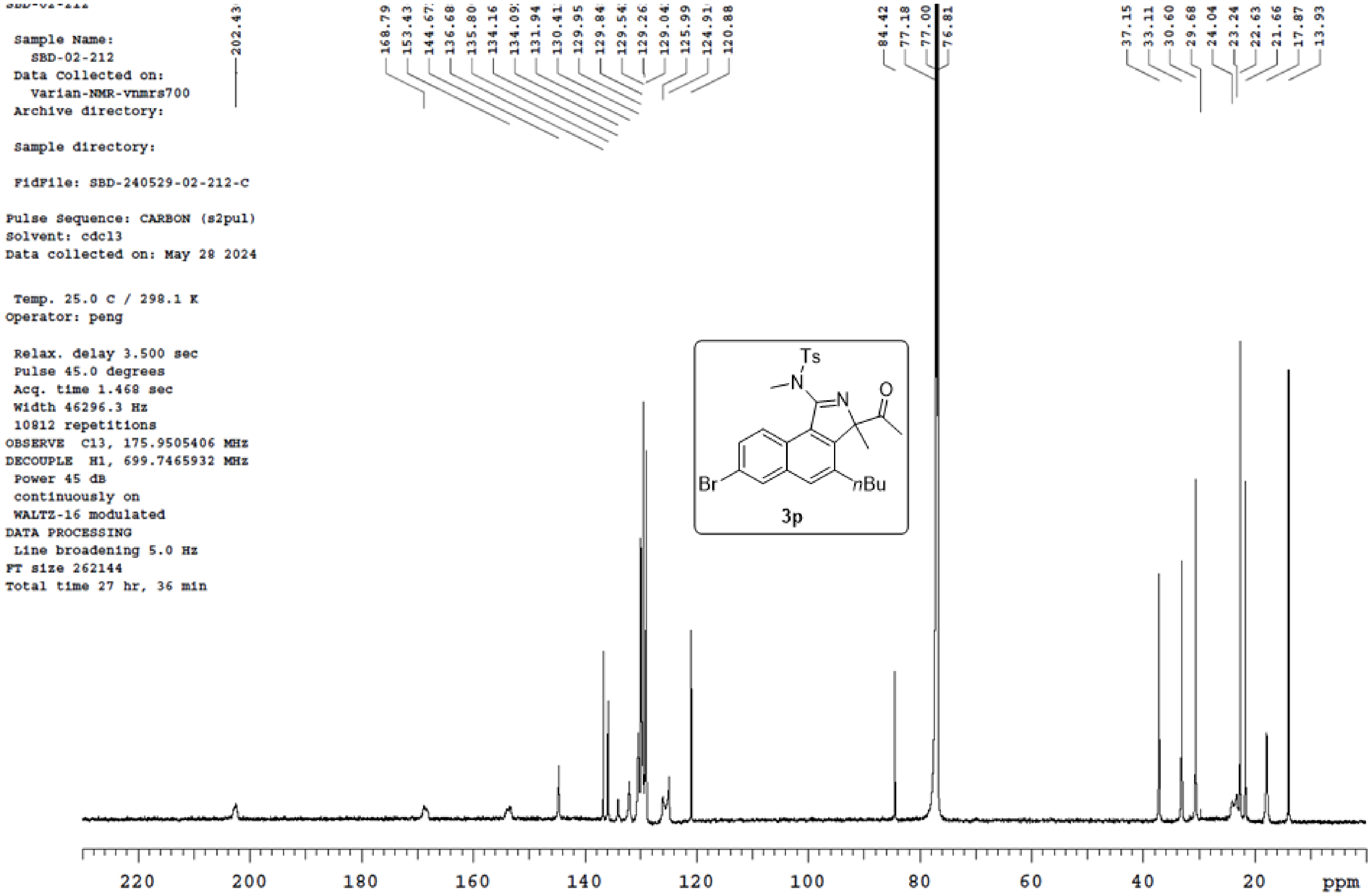

Solvent: $CDCl_3$
SFO1: 400 MHz

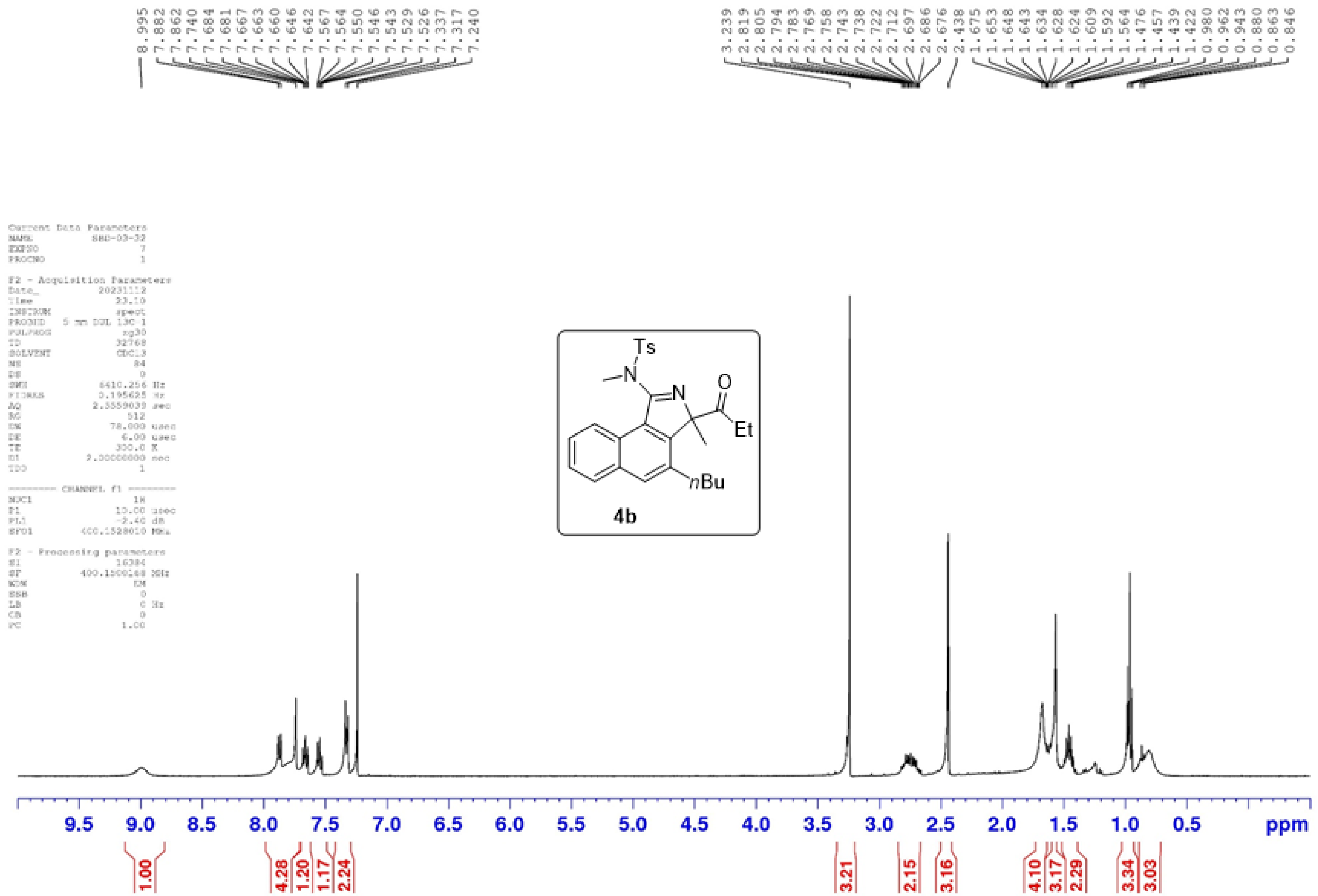

Solvent: $CDCl_3$
SFO1: 100 MHz

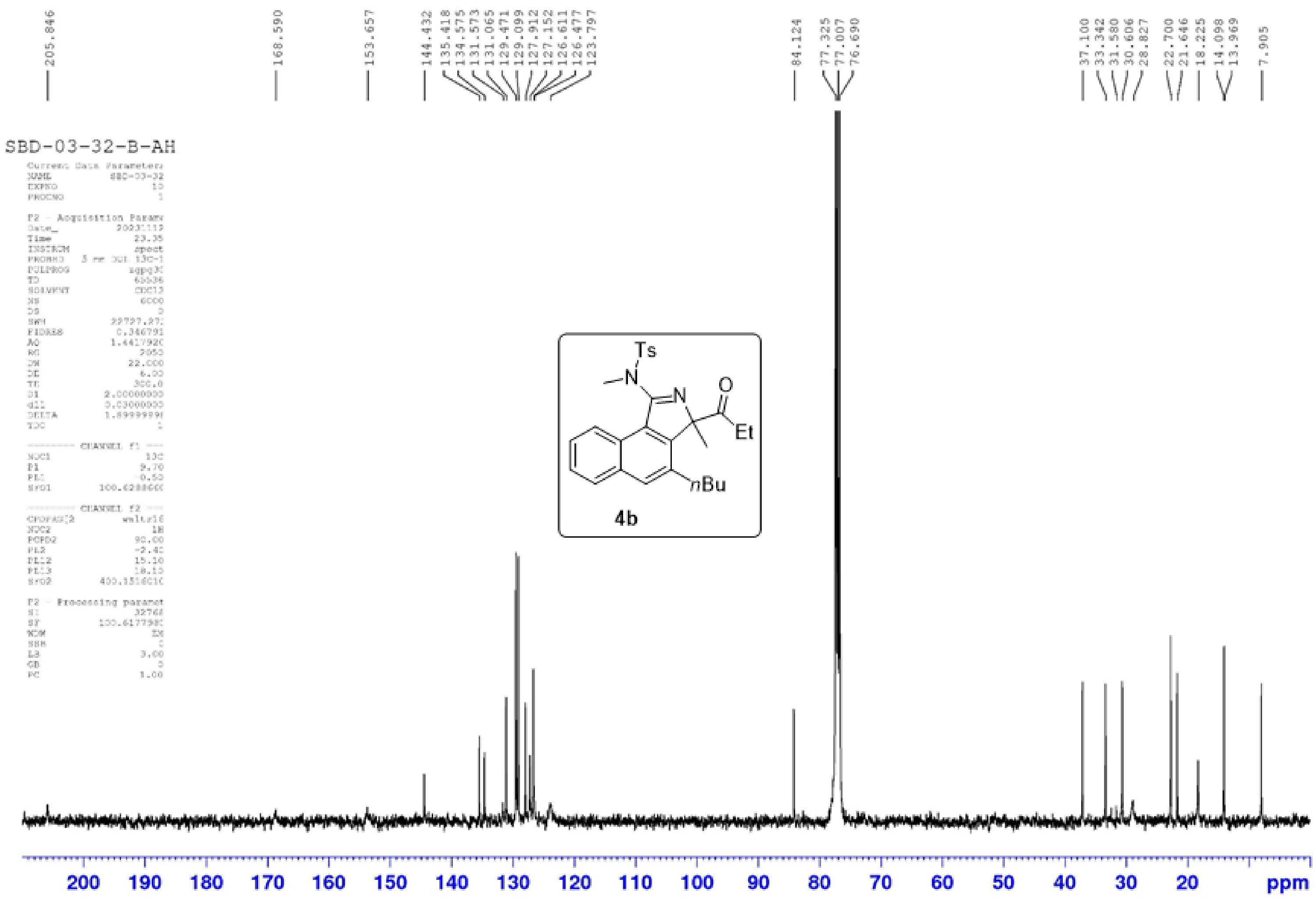

Solvent: $CDCl_3$
SFO1: 400 MHz

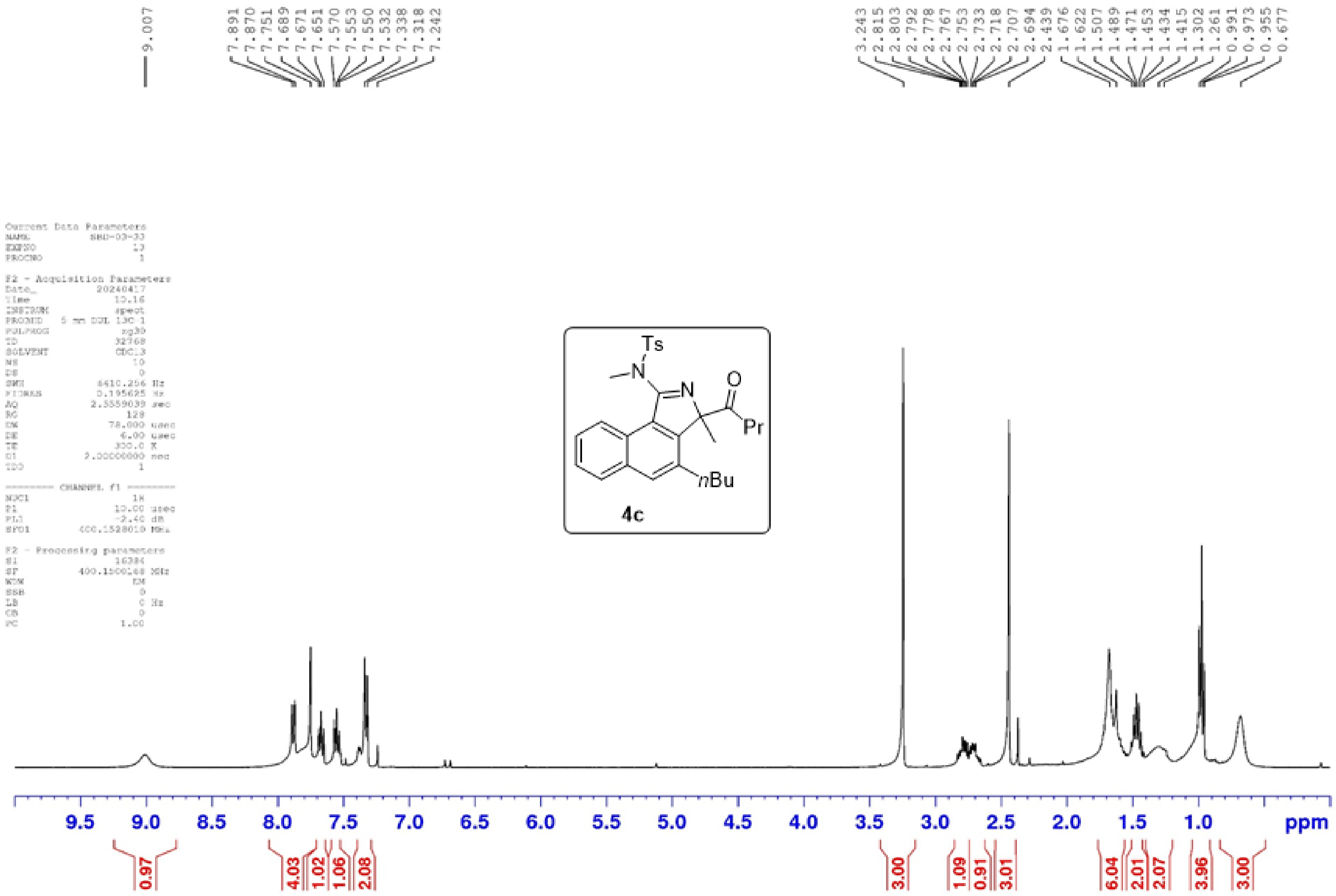

Solvent: $CDCl_3$
SFO1: 100 MHz

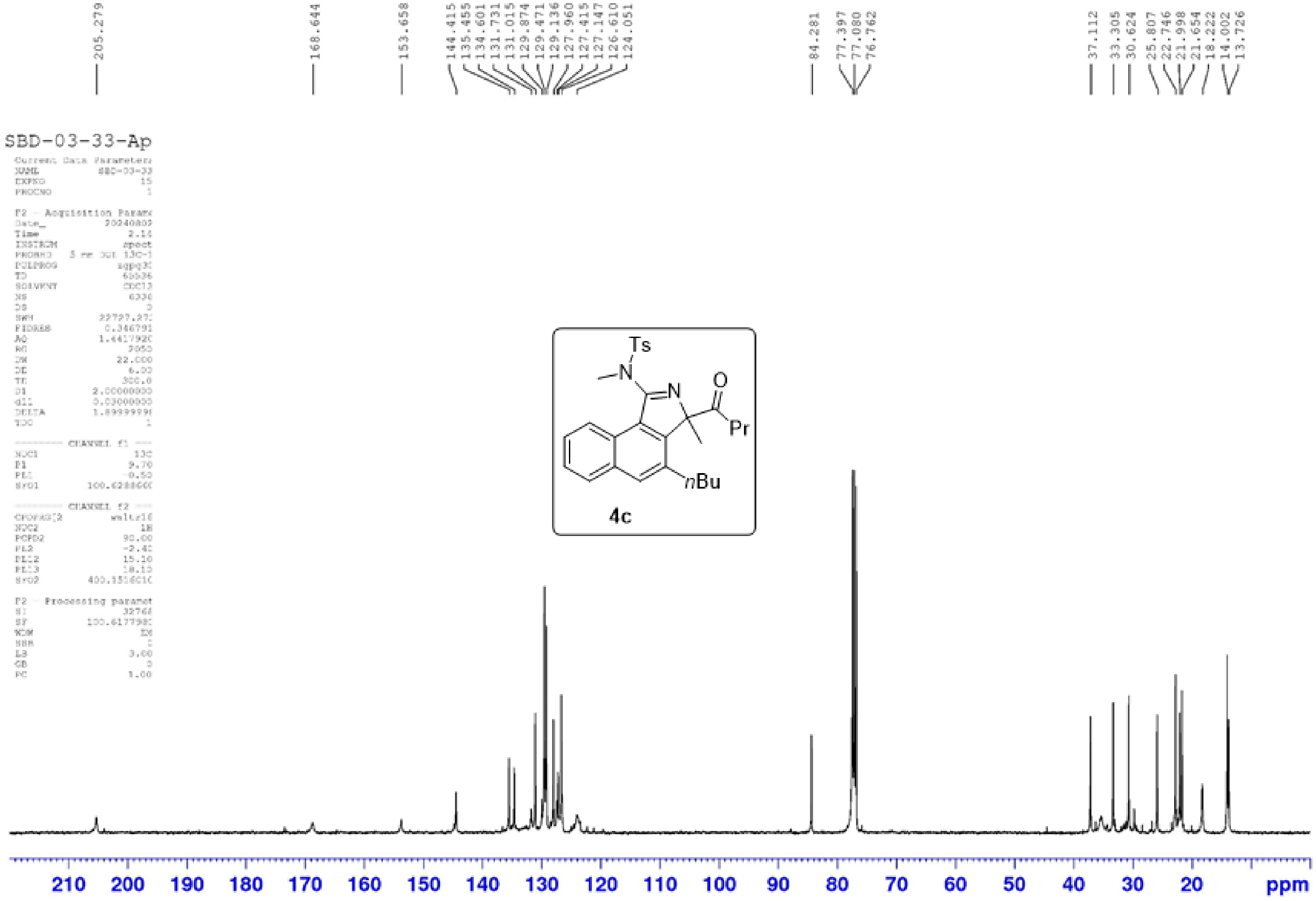

Solvent: $CDCl_3$
SFO1: 700 MHz

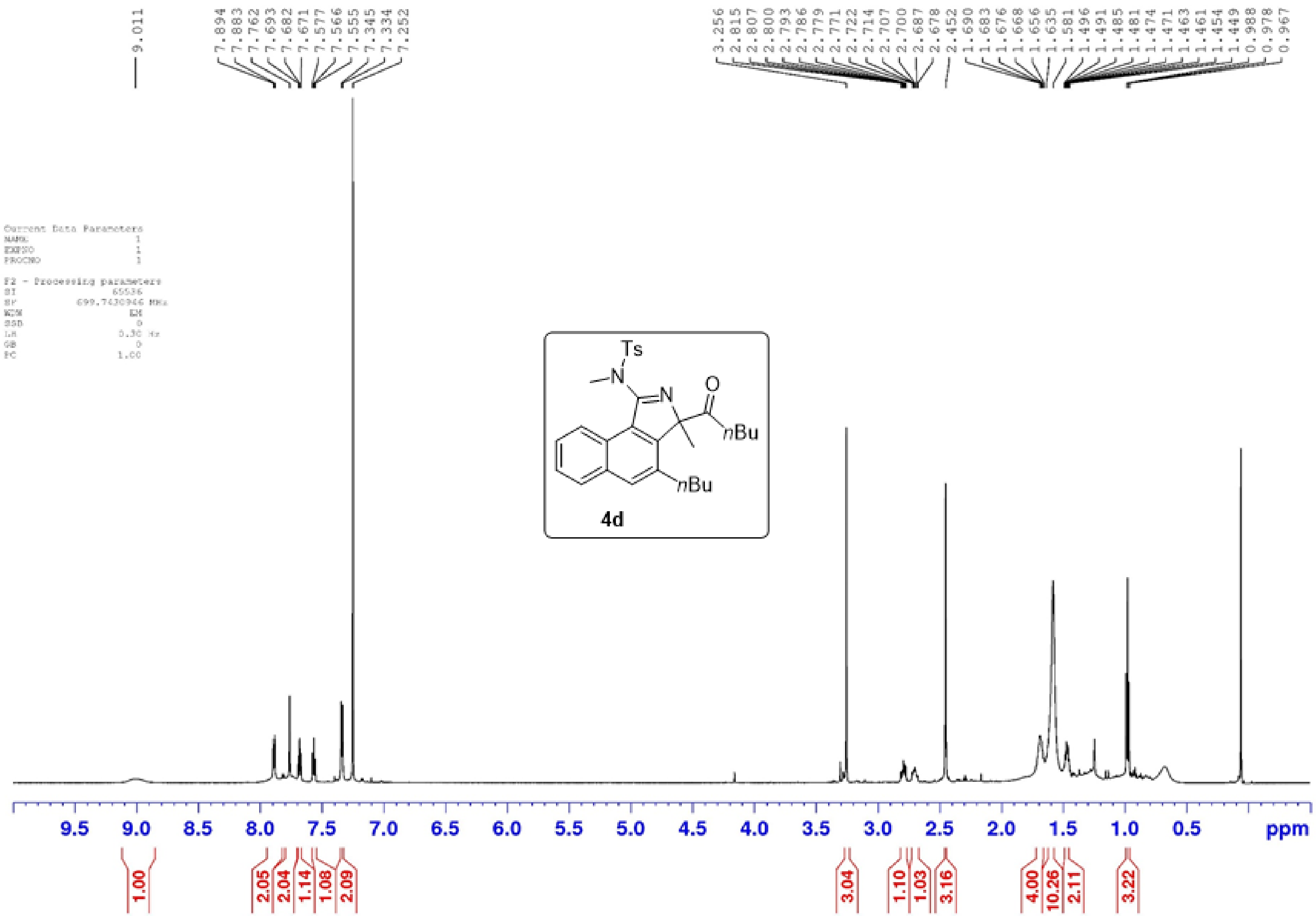

Solvent: $CDCl_3$
SFO1: 100 MHz

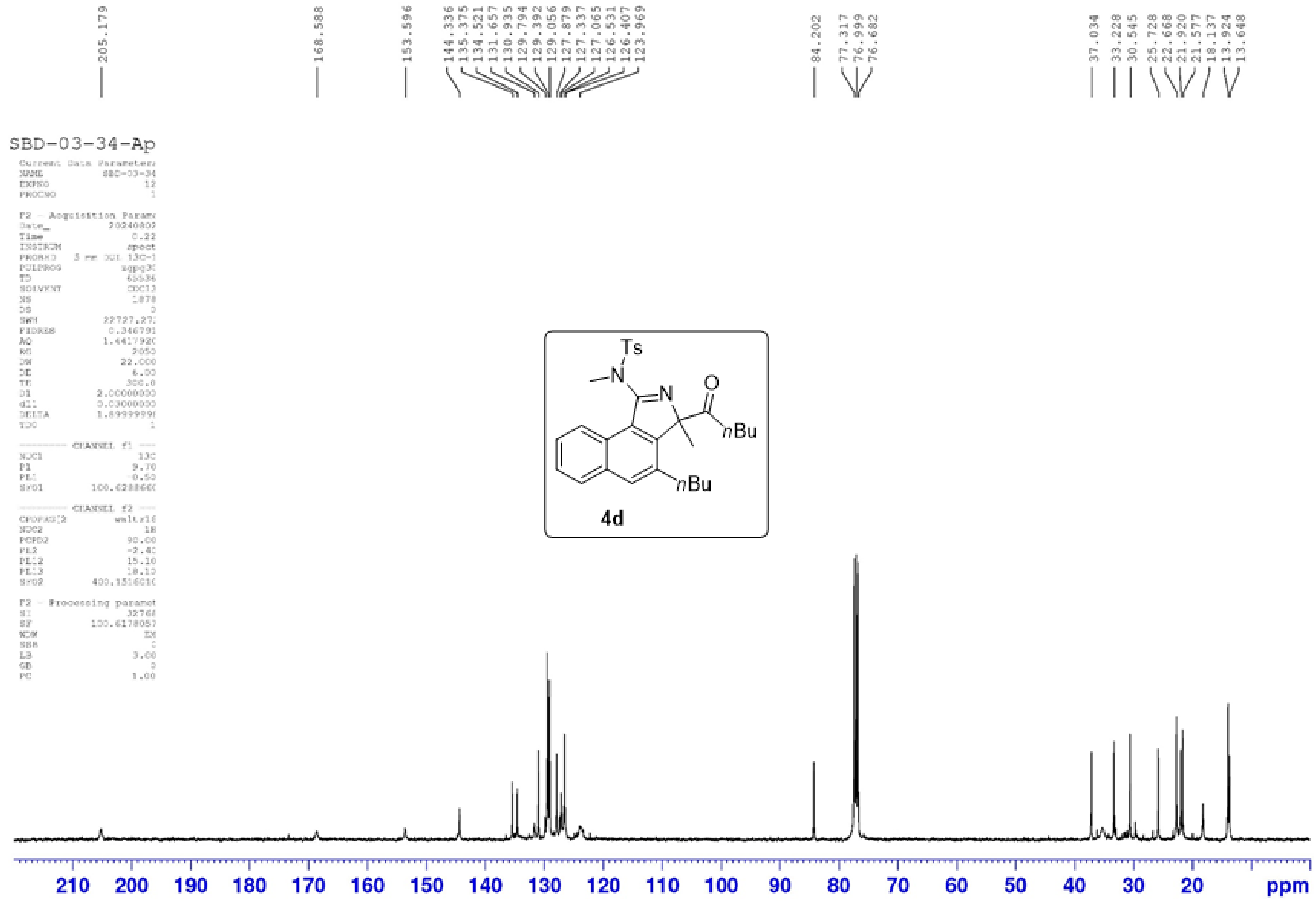

Solvent: $CDCl_3$
SFO1: 400 MHz

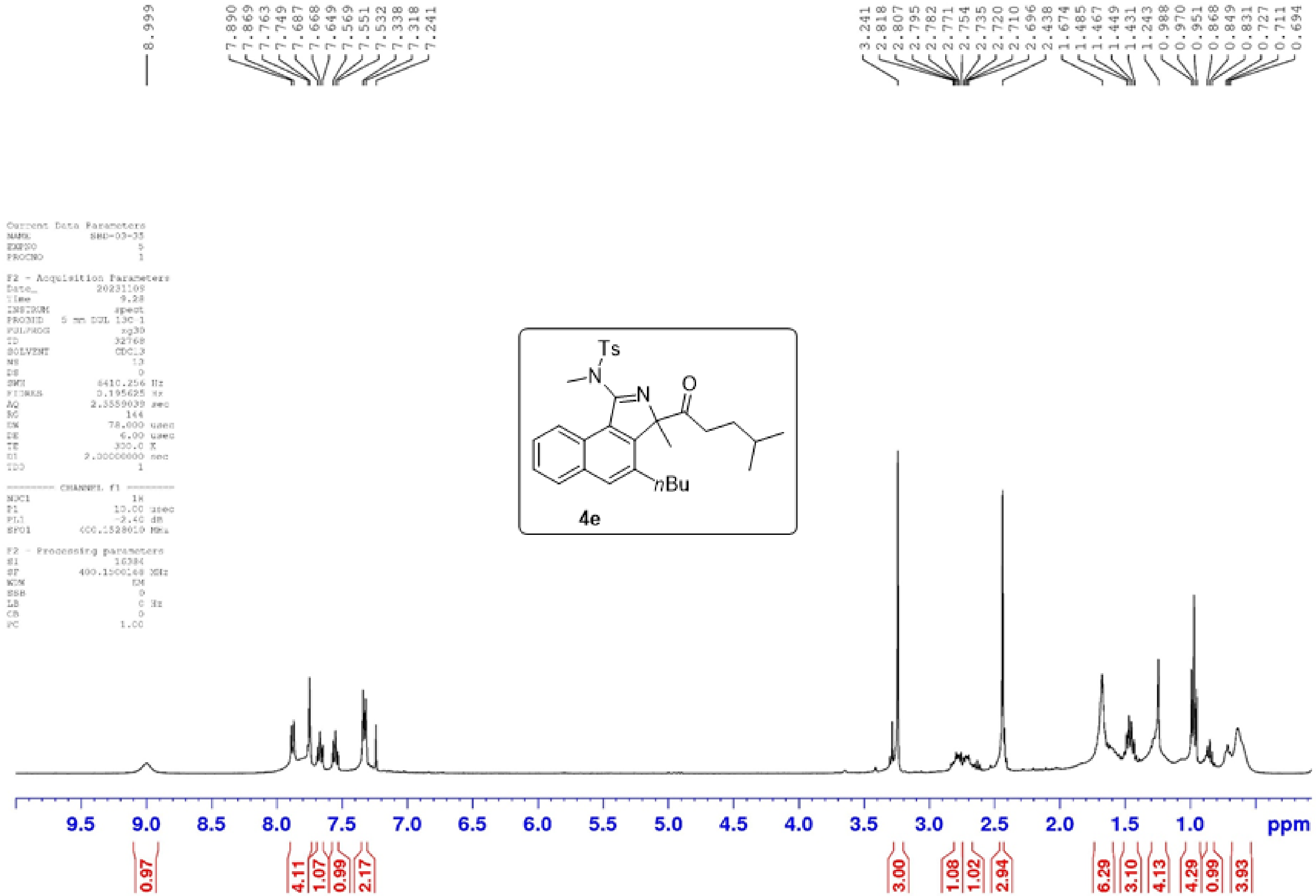

Solvent: $CDCl_3$
SFO1: 100 MHz

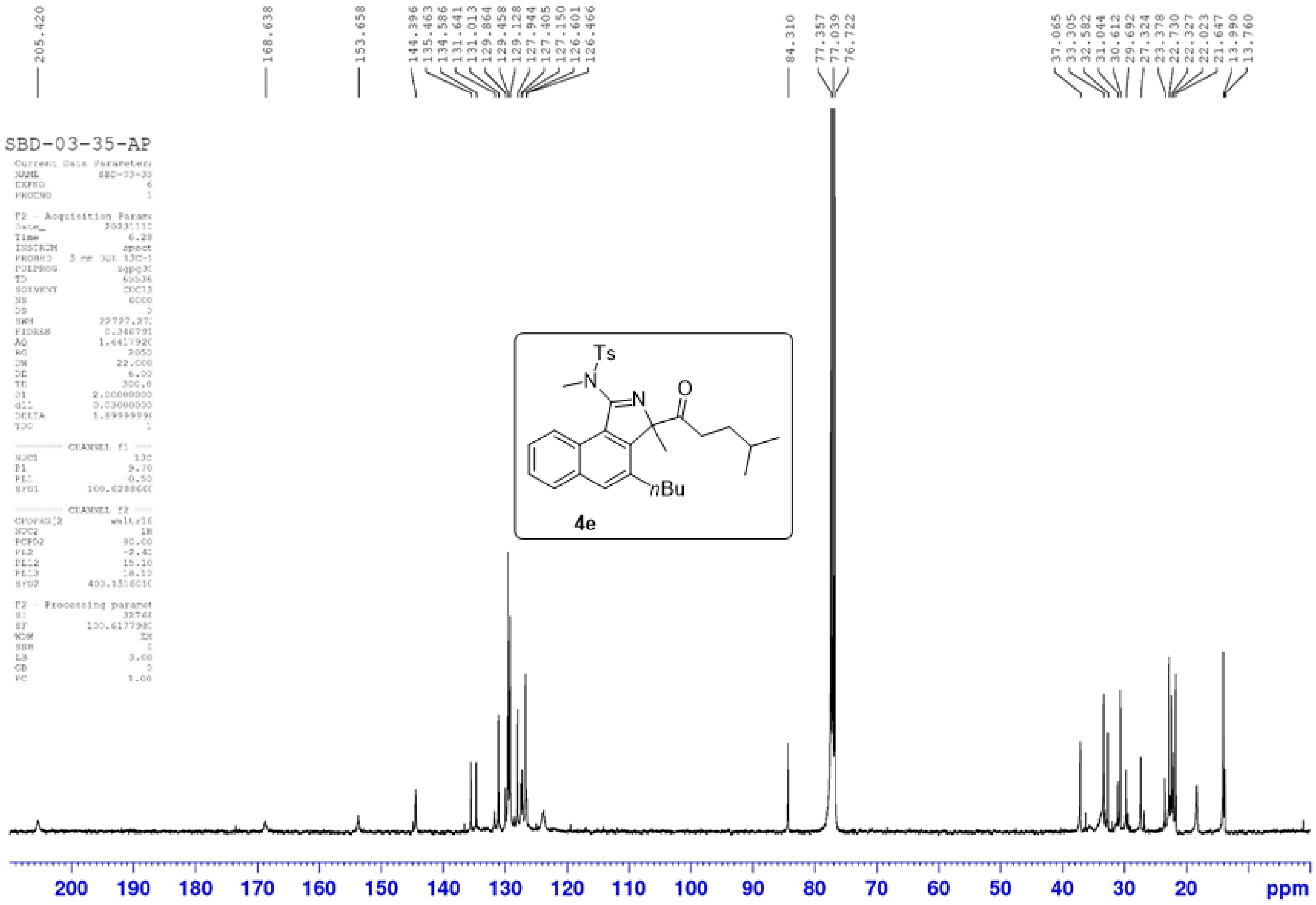

Solvent: $CDCl_3$
SFO1: 400 MHz

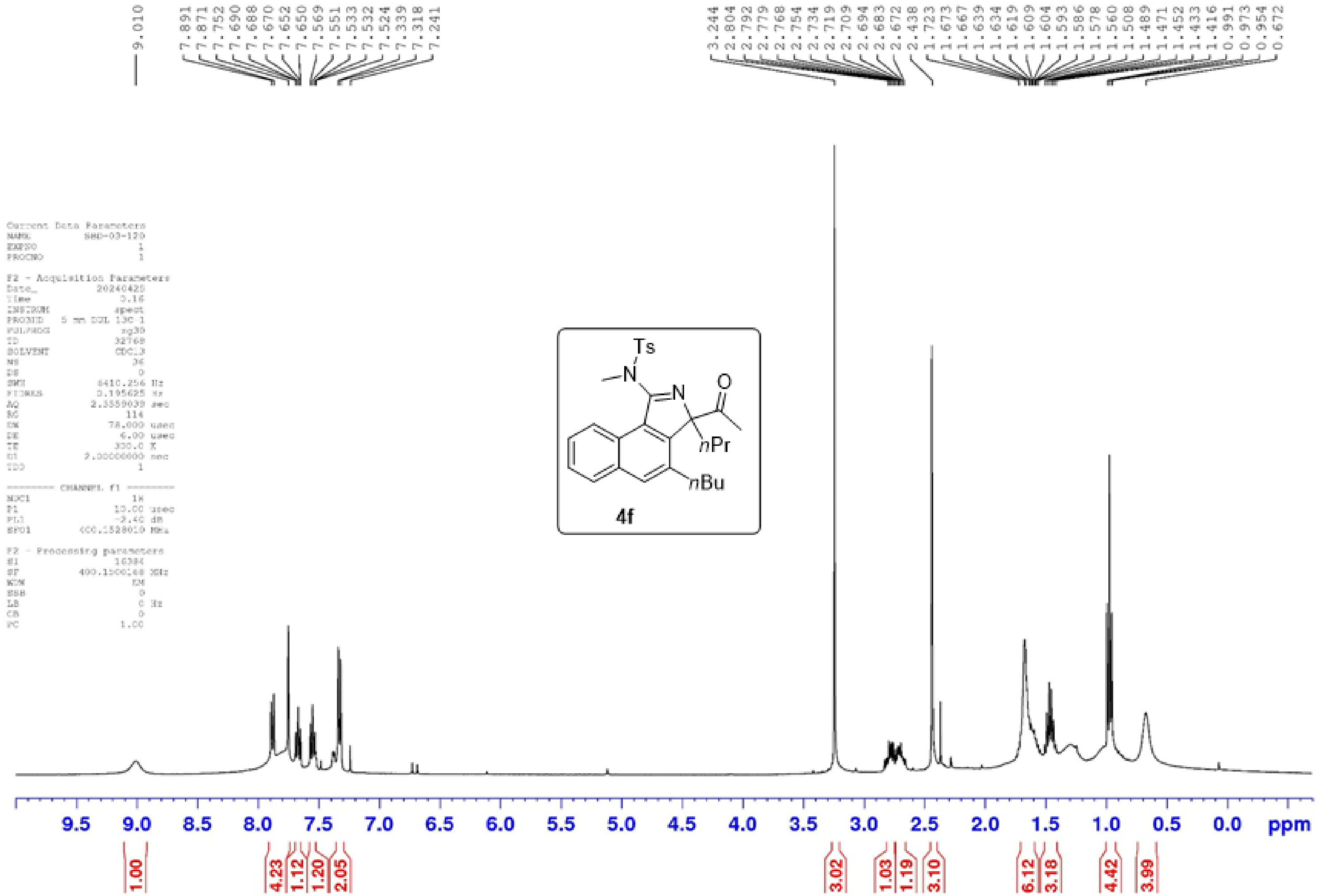

Solvent: $CDCl_3$
SFO1: 100 MHz

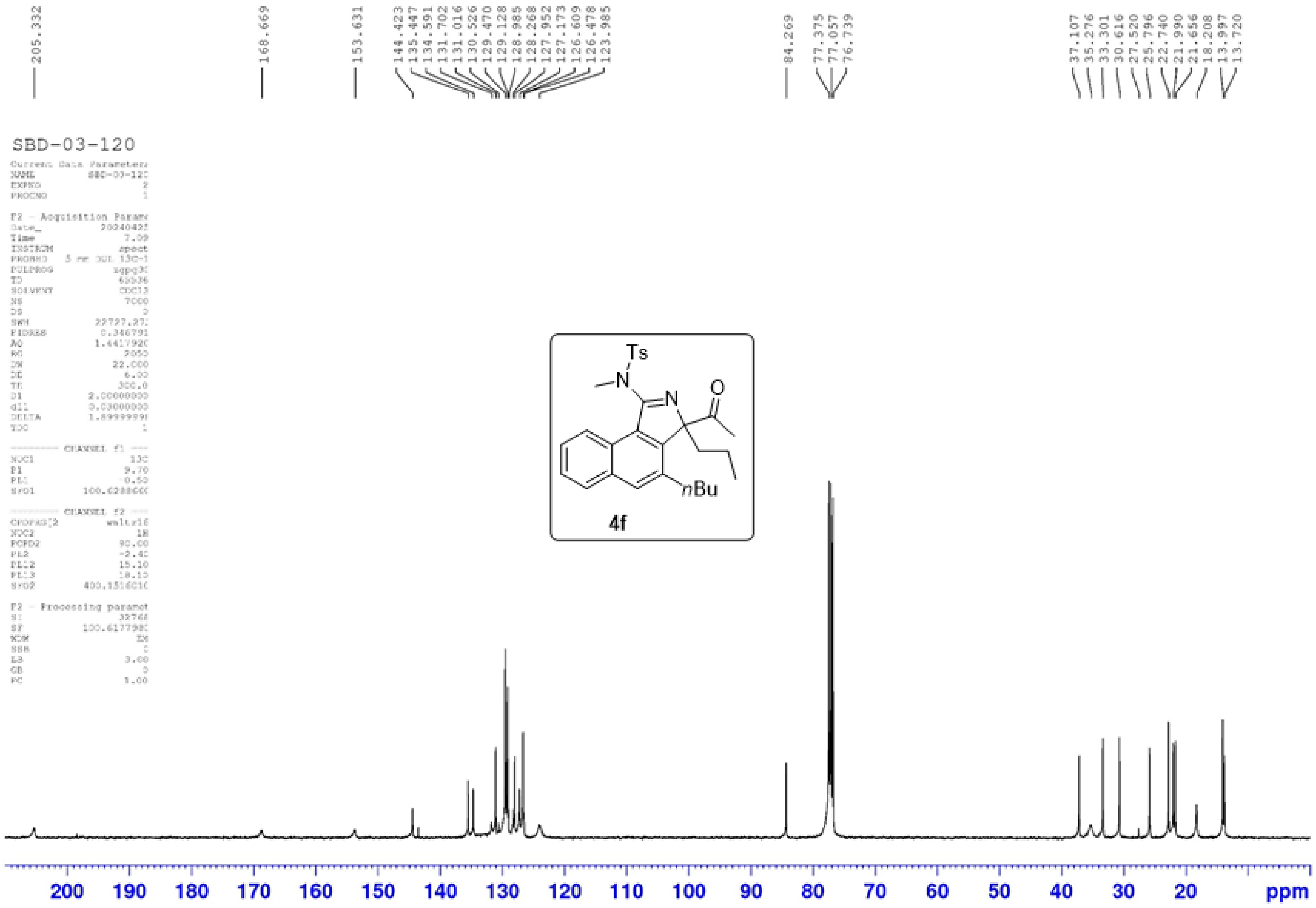

Solvent: $CDCl_3$
SFO1: 400 MHz

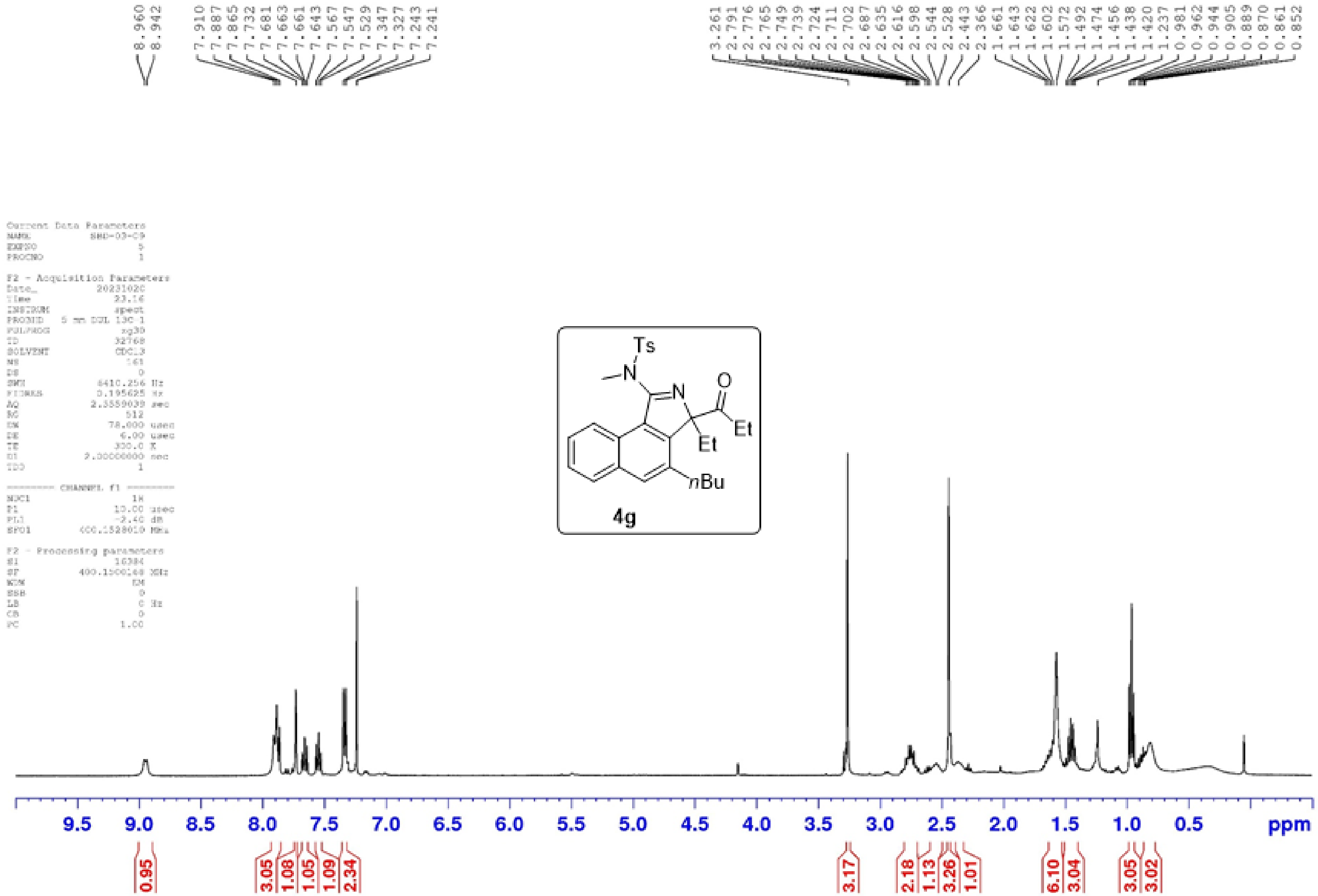

Solvent: $CDCl_3$
SFO1: 100 MHz

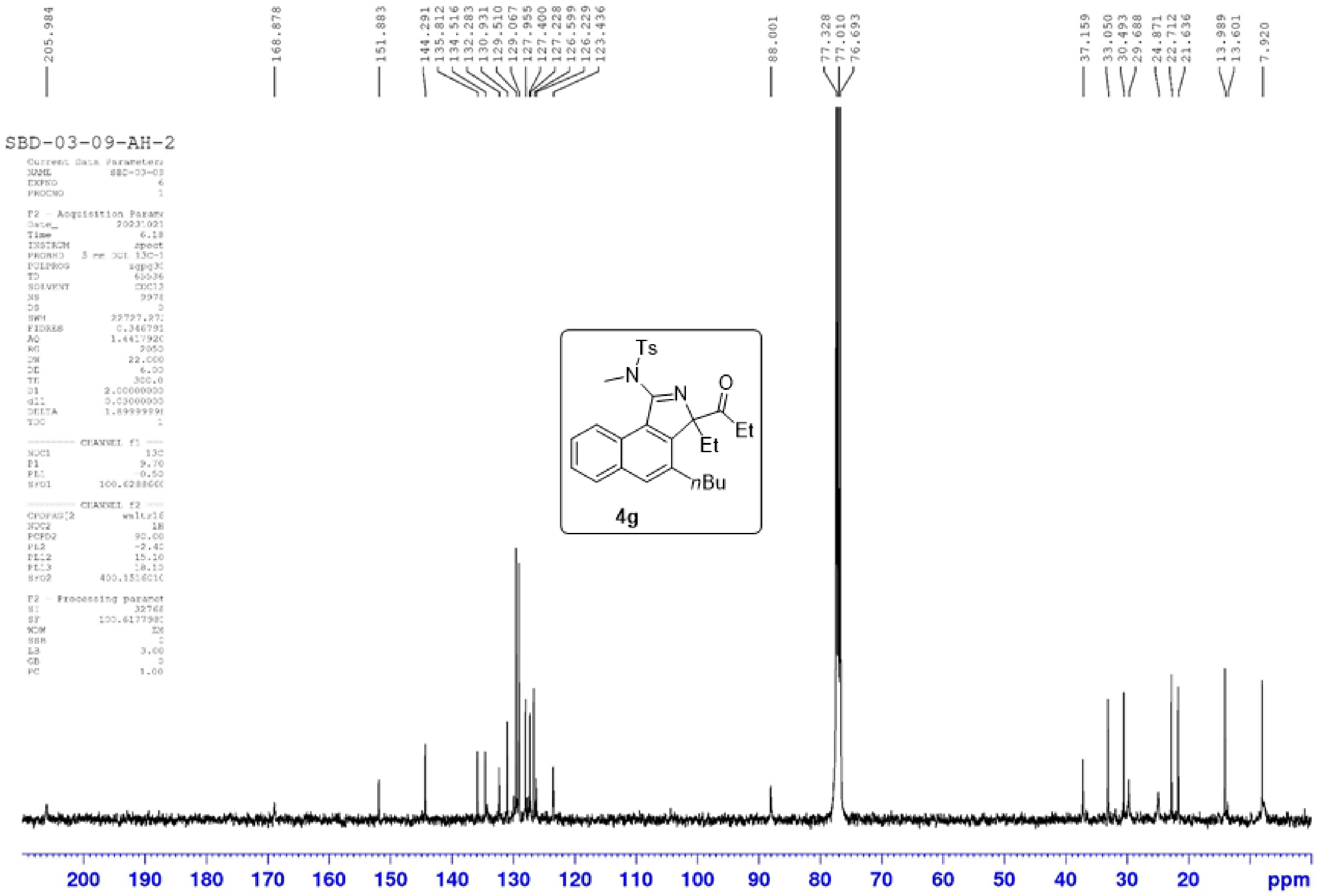

Solvent: $CDCl_3$
SFO1: 700 MHz

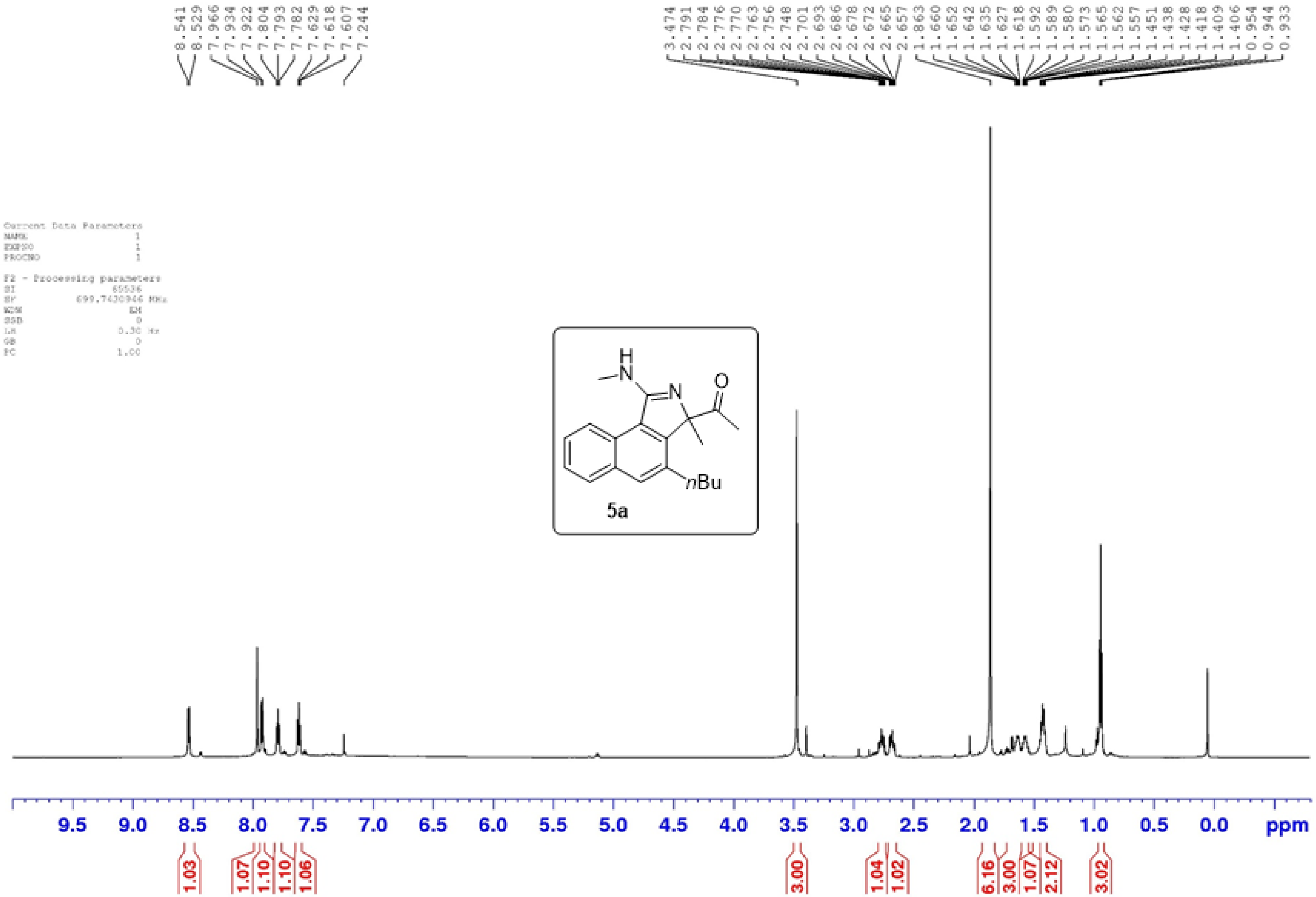

Solvent: $CDCl_3$
SFO1: 175 MHz

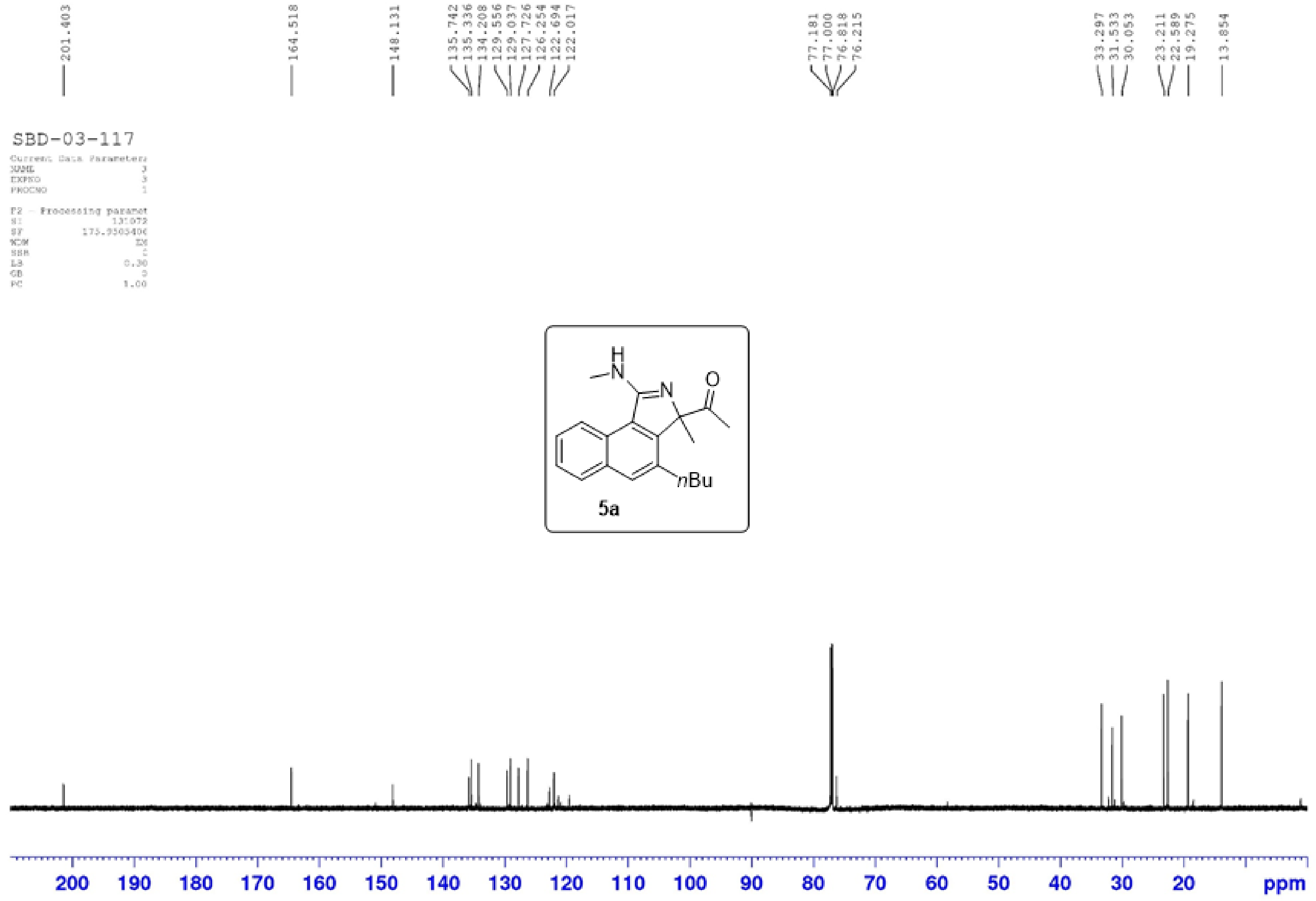

Solvent: $CDCl_3$
SFO1: 400 MHz

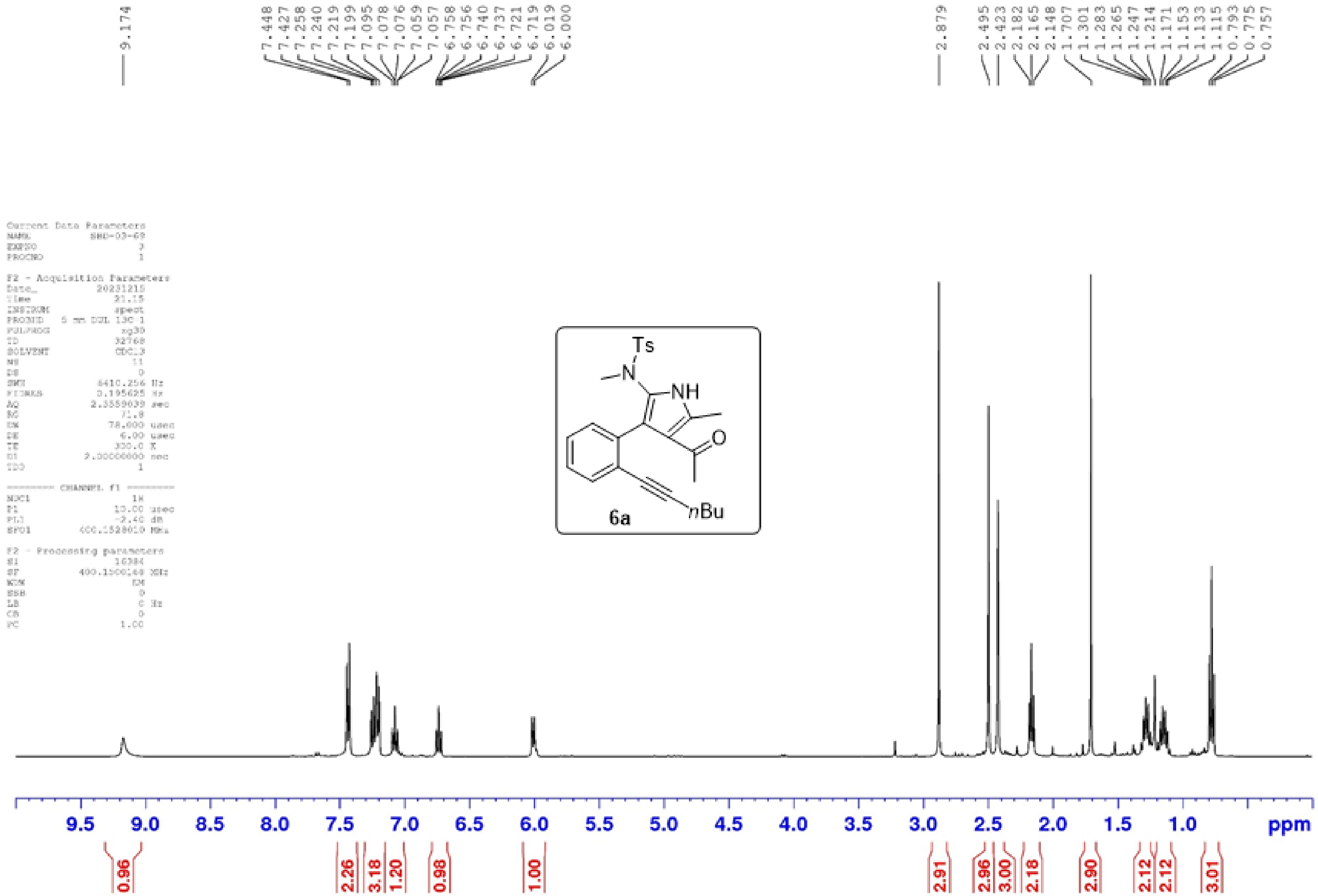

Solvent: $CDCl_3$
SFO1: 100 MHz

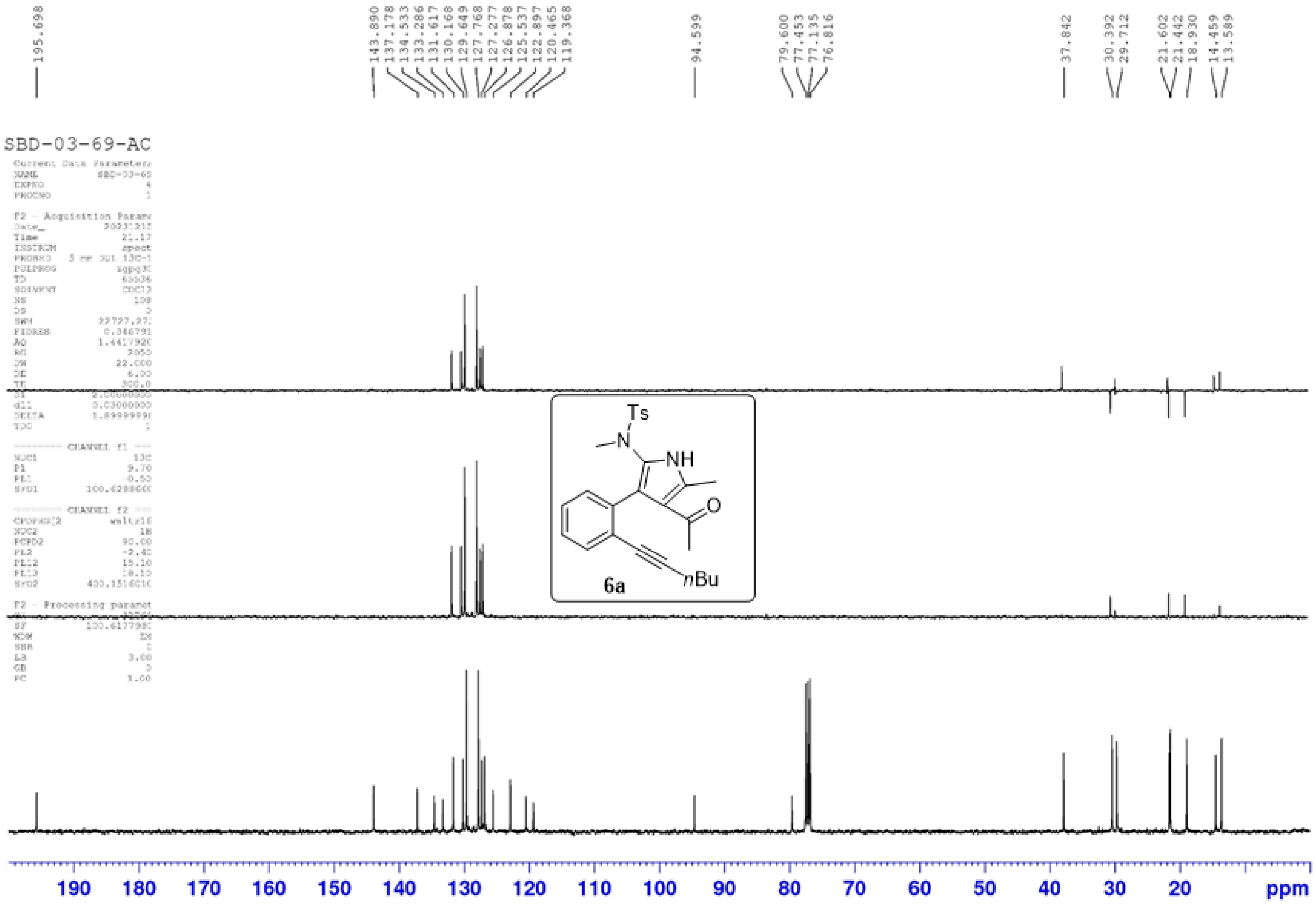

Solvent: $CDCl_3$
SFO1: 400 MHz

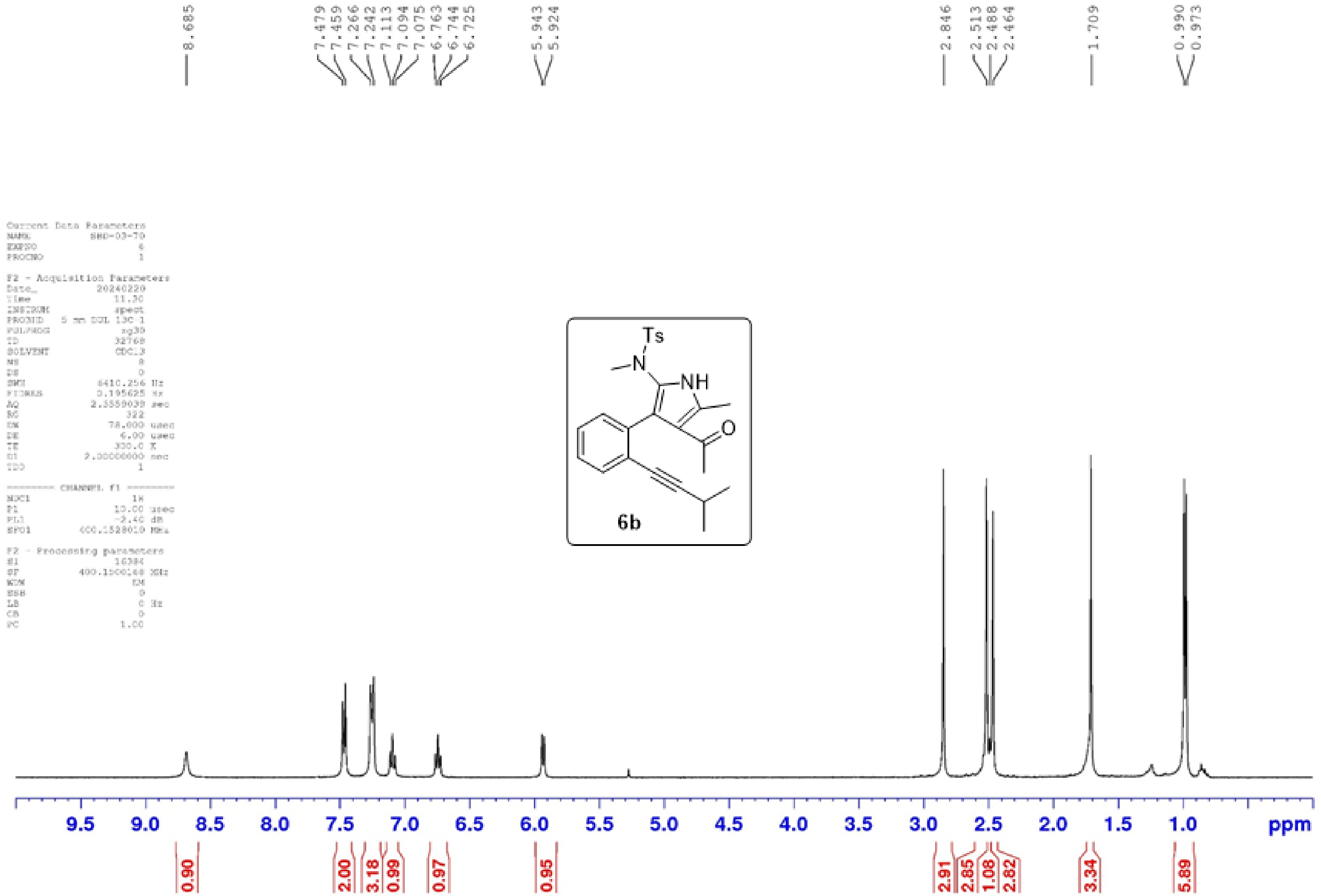

Solvent: $CDCl_3$
SFO1: 100 MHz

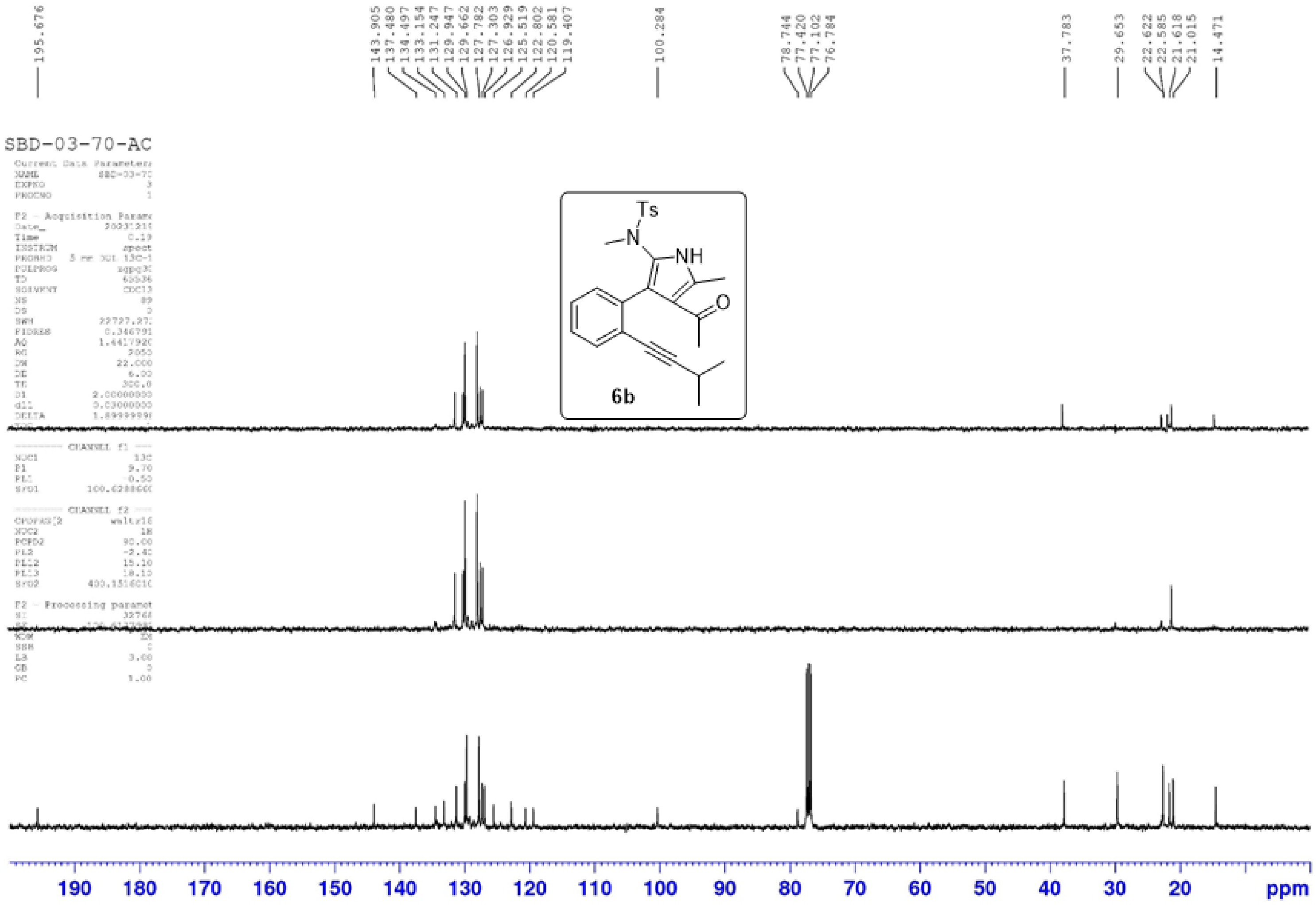

Solvent: $CDCl_3$
SFO1: 400 MHz

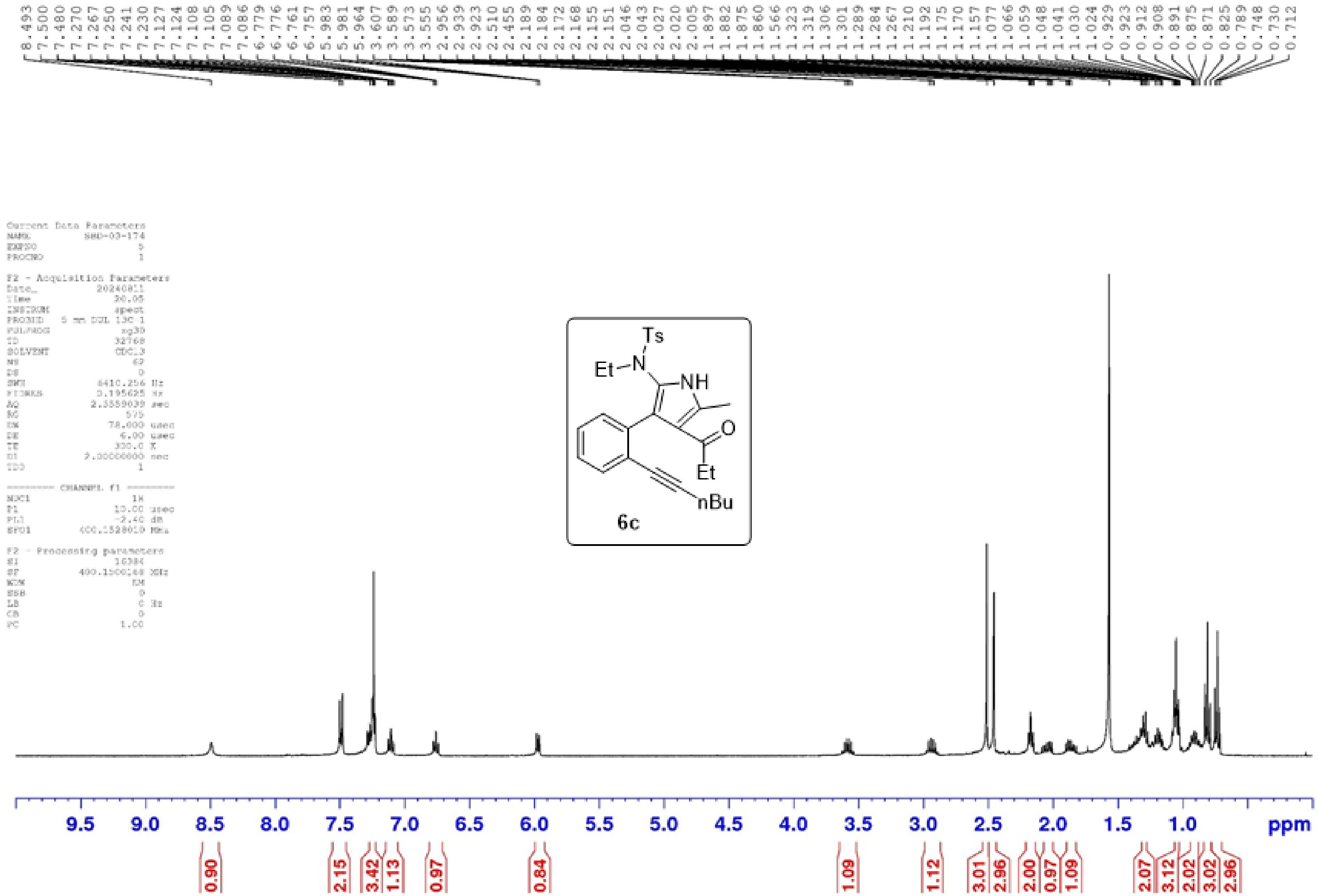

Solvent: $CDCl_3$
SFO1: 100 MHz

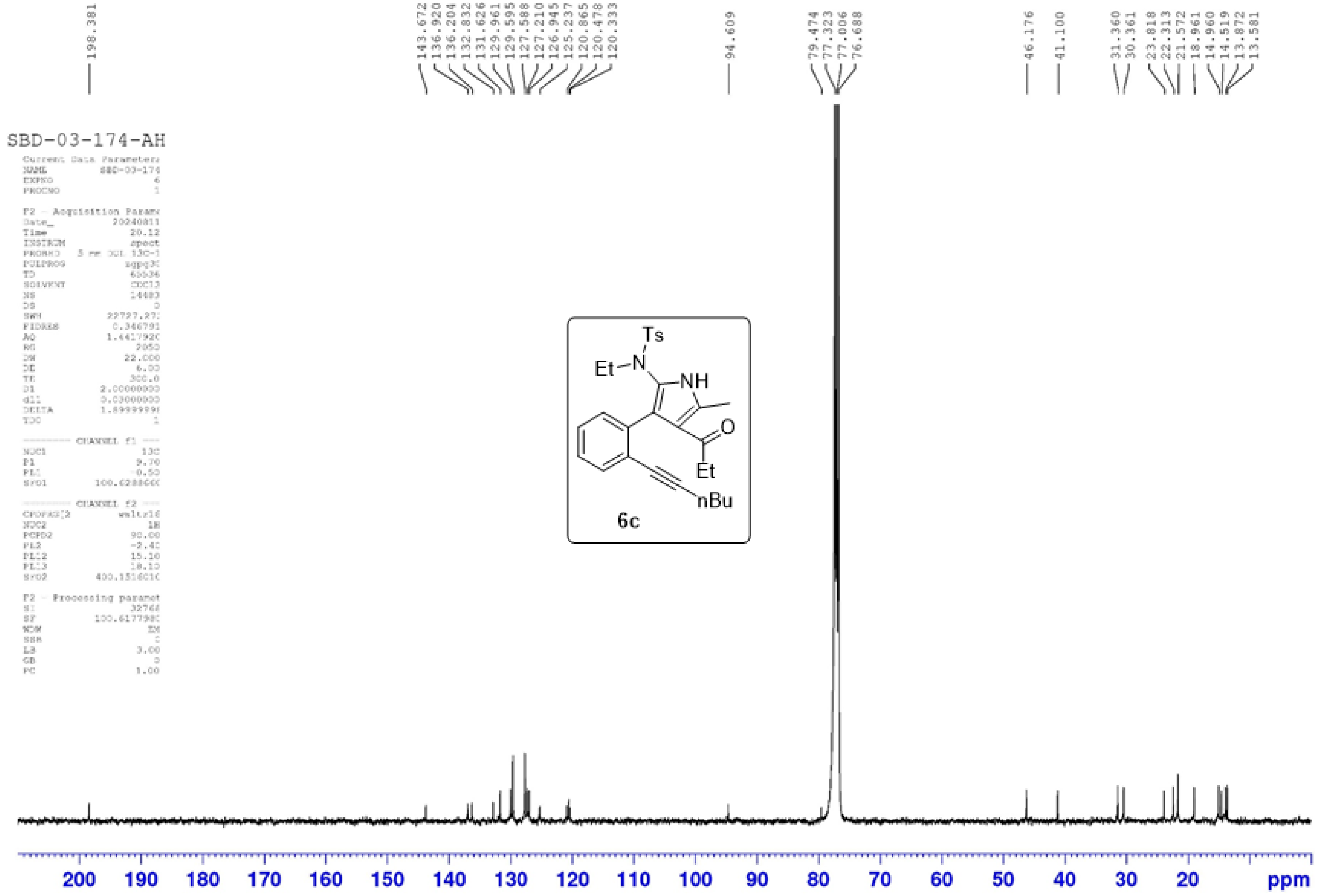

6c
